# Catalytic Amidation of Carboxylic Acids Using *ortho*-Aminophenylboronic Acids

**DOI:** 10.1021/acsorginorgau.6c00021

**Published:** 2026-05-12

**Authors:** Veera K. Bruce-Salmenkivi, Mikko Passiniemi, Petri M. Pihko

**Affiliations:** † Department of Chemistry and NanoScience Center, University of Jyväskylä, P.O. Box 35, Jyväskylä FI-40014, Finland; ‡ Orion Pharma, P. O. Box 65, Orionintie 1, FI-02200 Espoo, Finland

**Keywords:** catalysis, dehydrative amidation, organoboron catalyst, aminoarylboronic acid, catalyst screening

## Abstract

*ortho*-Substituted boronic acids have demonstrated significant potential as catalysts for direct amidation reactions, providing a sustainable alternative to conventional coupling reagents. We report the screening of *ortho*-functionalized arylboronic acids, including four literature benchmarks, as catalysts for the direct condensation of amines and carboxylic acids. The commercially available *ortho*-aminophenylboronic acids, specifically (2-(piperidin-1-yl)­phenyl)­boronic acid (2-PPBA), emerge as the optimal catalyst, providing amides from a range of carboxylic acids and amines.

## Graphical abstract

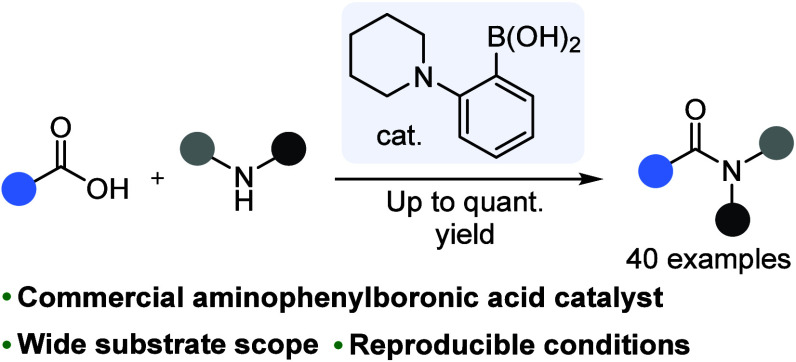

## Introduction

The formation of amides is one of the most frequently performed transformations in medicinal chemistry.^1^ Conventional methods for amide synthesis, however, typically rely on stoichiometric quantities of coupling reagents. Their use can pose challenges in terms of atom economy, environmental sustainability, and toxicity, and amide (peptide) coupling reagents have also raised concerns as sensitizing agents.^2–4^ The critical role of amide formation, along with the associated problems, has also prompted the ACS Green Chemistry Institute to recognize amidation reactions avoiding poor atom economy reagents as a key research area.^5^

In response to these challenges, more atom economic and sustainable amidation protocols have been developed in recent years.^6–9^ Among the possible alternatives, boronic acid catalysts and their derivatives are highly attractive, as they provide greener and safer access to amides under relatively mild conditions. Since Yamamoto et al.’s introduction of 3,4,5-trifluorophenylboronic acid as amidation catalysts in 1996 (Scheme 1), numerous attempts have been made to optimize the electronic and steric properties of boronic acid catalysts to achieve catalytic activity that results in yields comparable to those obtained with the conventional coupling reagents.^10–28^

**Scheme 1:**
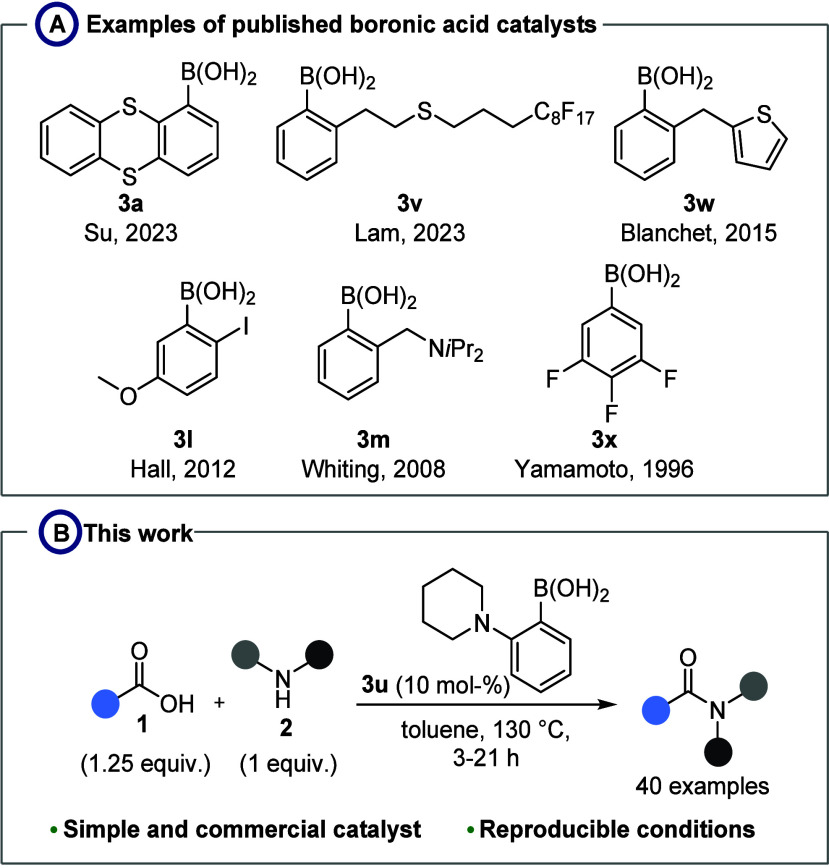
(A) Examples of Known Boronic Acid Catalysts (**3a**, **3v**, **3w**, **3l**, **3m**, **3x**). (B) (2-(Piperidin-1-yl)­phenyl)­boronic Acid (2-PPBA, **3u**) Catalyzed Amidation Protocol

In particular, *ortho*-substituted aryl boronic acids frequently feature in successful catalyst designs (Scheme 1a). In 2008, Whiting and co-workers introduced *N*,*N*-di-isopropylbenzylamine-2-boronic acid (**3m**) and its derivatives as effective catalysts for direct amidations.^14^ Later, in 2012, Hall et al. reported 5-methoxy-2-iodophenylboronic acid (**3l**, MIBA) as an active catalyst that operates in lower temperatures (in the presence of drying agents).^15^ During the last 10 years, sulfur-containing *o*-substituted boronic acids (**3a**–**3w**) have raised interest and led to noteworthy results.^13,16,17^

Herein, we report that (2-(piperidin-1-yl)­phenyl)­boronic acid (2-PPBA, **3u**) emerges as a robust, structurally simple, and commercially available catalyst for the direct amidation of carboxylic acids and amines (Scheme 1b). A wide range of aromatic and aliphatic carboxylic acids and amines were tolerated, providing amides up to quantitative yields. The method is operationally straightforward and provides reproducible results without the need for additional molecular sieves, which are often tedious to activate. Moreover, the amidation reactions exhibited good tolerance to racemization in the case of amino amides, while dipeptides were afforded in varying yields and diastereomeric purities.

## Results and Discussion

The study was initiated by identifying optimal and reproducible reaction conditions for catalyst screening experiments. The purpose of the optimization was to ensure that the differences in catalytic activity would not originate from disparities in the water-removal efficiency in small scale reactions. A model reaction between benzoic acid (**1i**) and benzyl amine (**2a**), catalyzed by thianthren-1-ylboronic acid (**3a**),^13^ was selected (Table 1), and the conversions were recorded with LC–MS using 1,3,5-trimethoxybenzene as an internal standard. In response to the low initial conversions (Table 1, entry 1), the catalyst loading was raised from 10 to 15 mol % (Table 1, entry 2) and the excess of **1i** from 1.1 to 1.25 equiv (Table 1, entry 3). These modifications proved critical in ensuring that the model reaction gave consistently reproducible conversions. In addition, temperatures between 110 and 130 °C were evaluated (Table 1, entries 4–6), confirming that refluxing at 130 °C (heater block temperature) is required to ensure efficient reaction progress. Alternative high-boiling solvents, including DMSO, xylene, anisole, and 1,2-dichlorobenzene (Table 1, entries 7–10), were screened to see whether elevated temperatures would enhance reaction rates. However, toluene as the solvent consistently provided the highest conversion under the tested conditions. Dichloromethane (DCM) in combination with molecular sieves (commonly employed solvent system) was deliberately avoided in this study due to sustainability considerations^29^ and the practical challenges associated with the activation of molecular sieves.

**Table 1:**
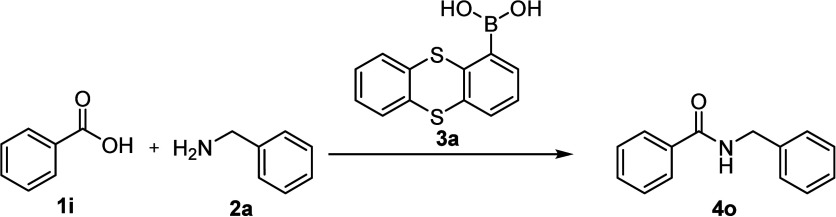
Optimization of the Reaction Conditions Using **3a** as the Catalyst

entry	3a (mol %)	temperature (°C)^a^	solvent	1i (equiv)	time (h)	4o (%)^b^
1	10	130	toluene	1.1	7	69%
2	15	130	toluene	1.1	7	78%
3	15	130	toluene	1.25	10	80%
4	15	120	toluene	1.25	7	70%
5	15	110	toluene	1.25	12	2%
6	15	115	toluene	1.25	7	55%
7	15	130	DMSO	1.25	7	0%
8	15	130	anisole	1.25	4	0%
9	15	130	xylene	1.25	7	51%
10	10	130	*o*-DCB	1.1	16	47%

a The temperature corresponds to the aluminum heater block temperature.

b Yield determined with quantitative LC–MS.

Next, a broad range of boronic acids were evaluated in the model reaction between **1i** and **2a** (**3** 15 mol % in toluene at 130 °C). Reaction progress was carefully monitored with quantitative LC–MS to assess differences in reaction rates (Scheme 2, for more details, see the Supporting Information), and catalysts that exhibited poor activity were rechecked under identical conditions to confirm the reliability of the observed trends. To check whether the observed differences in the activities were due to moisture, we also carried out a control experiment with water added. In this experiment, 50 mol % water was initially added to the reaction catalyzed by **3a** (15 mol %) between **1i** and **2a** (in toluene at 130 °C), yet no change in reaction progress was observed, indicating that the initial presence of water does not reduce the catalytic activity under reflux conditions.

**Scheme 2:**
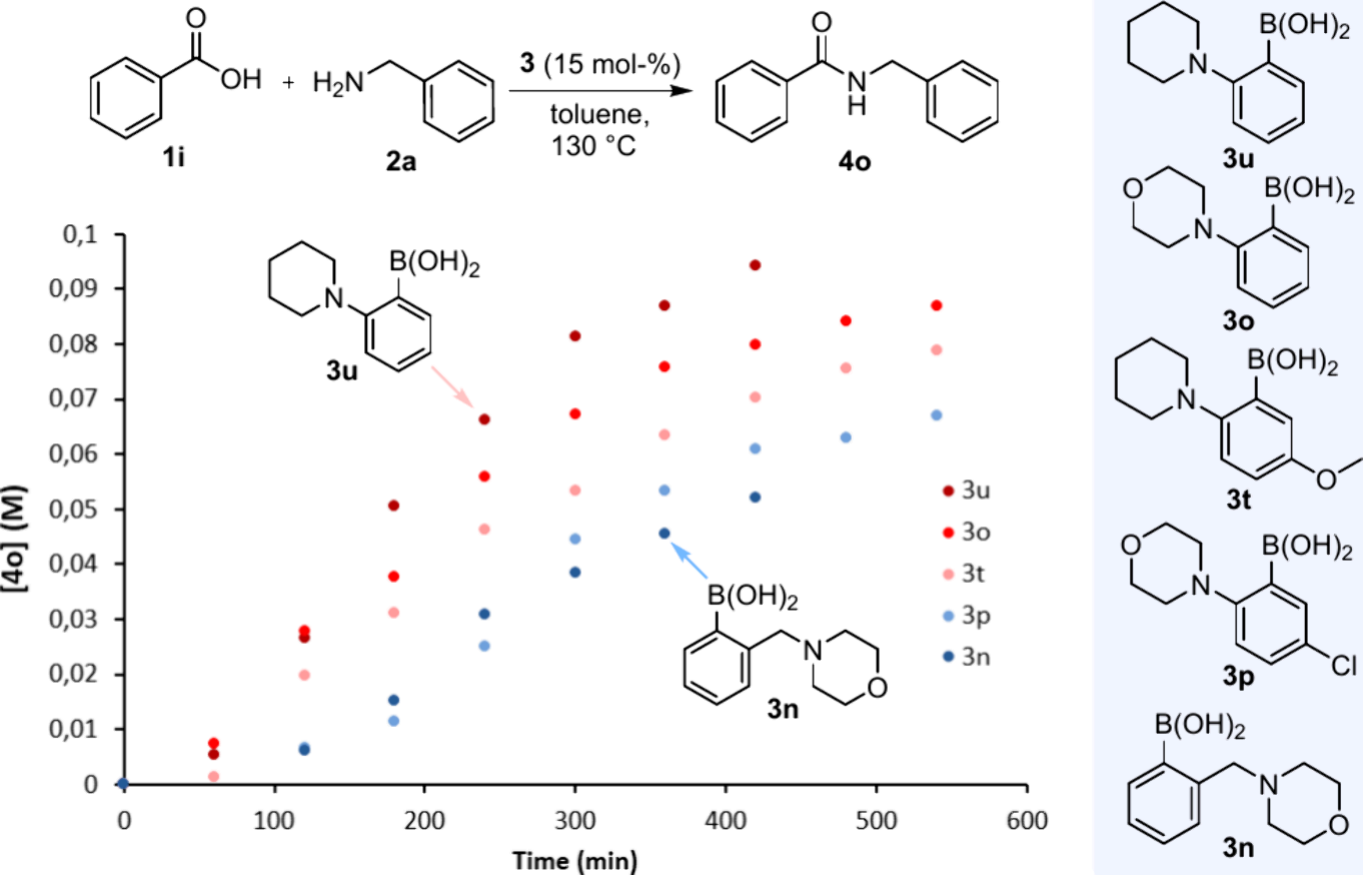
Reaction Progress Curves from the Reaction between **1i** and **2a** to Afford **4o** with Selected Tertiary Amine Boronic Acid Catalysts (**3n**, **3o**, **3p**, **3t**, **3u**)

Initially, sulfur- and oxygen-containing boronic acids (**3c**–**3f**) were examined, but only **3c** delivered satisfactory results (84%) (Scheme 3). Consequently, a series of heterocyclic boronic acids (**3g**–**3k**) were designed to achieve improved activity, but conversions with these catalysts remained only moderate (≤52%). As *ortho*-substituted boronic acids (**3c**, **3d**) afforded the highest conversions, a focused set of *ortho*-substituted, amino-functionalized phenylboronic acids (**3o**–**3u**) were selected for further investigation (Scheme 3).

**Scheme 3:**
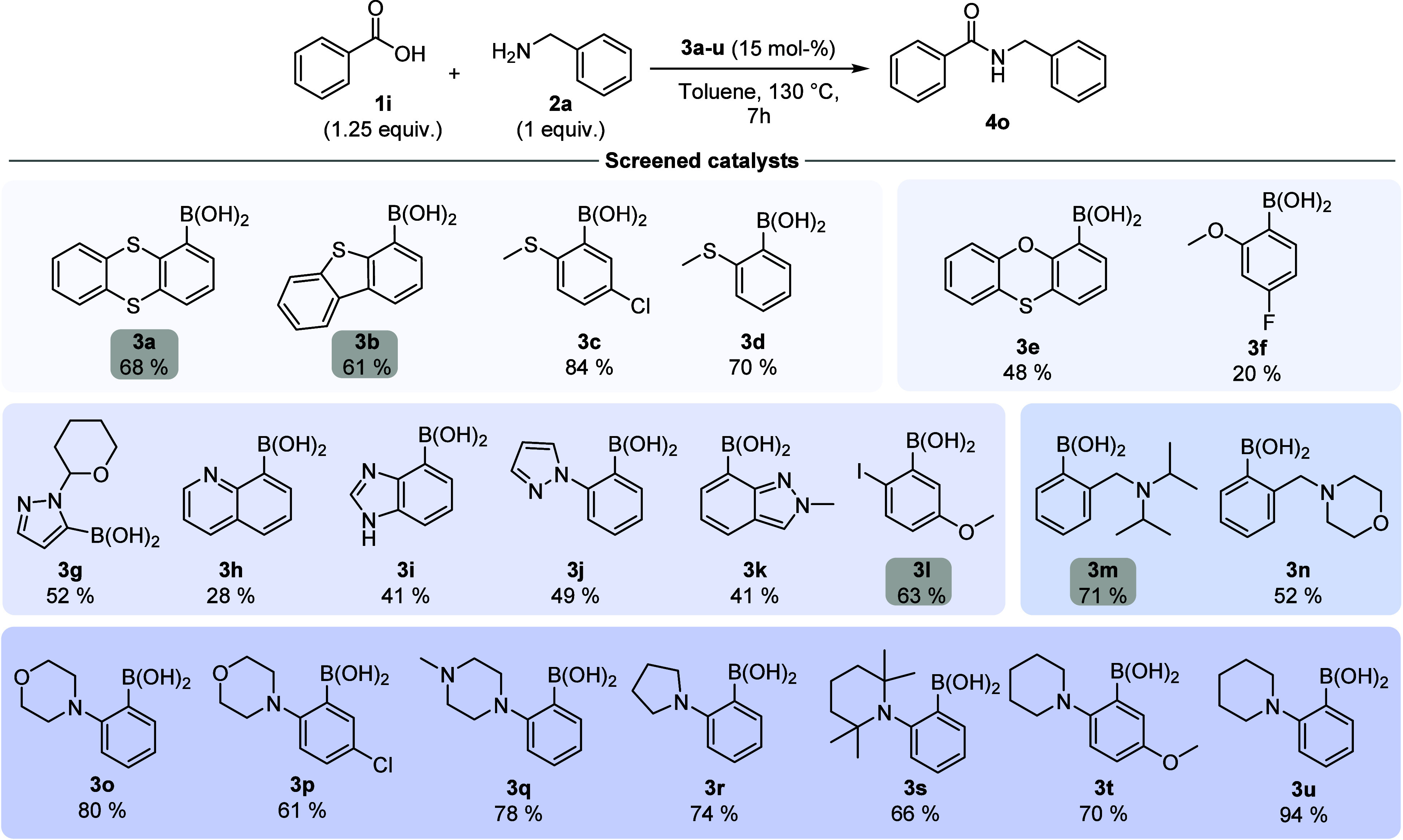
Set of Screened Boronic Acids with the Reaction between **1i** and **2a** ^a^

a The catalysts are divided into groups according to their structure. Known boronic acid catalysts (**3a**, **3b**, **3l**, **3m**) are highlighted in gray. Reported yields of product **4o** are recorded after 7 h.

Morpholinyl (**3o**) and 4-methylpiperazin-1-yl (**3q**) boronic acids delivered promising results, affording yields of up to 80%. Introduction of a piperidinyl moiety (**3u**) further increased the yield of **4o** to 94%, whereas the more basic pyrrolidinyl-substituted catalyst (**3r**) did not lead to a higher conversion. In addition, the catalytic activity decreased when an extra methylene unit was introduced between the nitrogen atom and the aromatic ring (**3n**, 52%). Similarly, introduction of either electron donating (**3t**) or withdrawing (**3p**) substituents at the *meta*-position led to a pronounced suppression of catalytic activity (70%, 61%). This was particularly surprising given that Hall’s catalyst (**3l**), featuring an electron donating methoxy group at the *meta*-position and a bulky iodide substituent at the *ortho*-position, has been reported to exhibit good activity.^15^ The introduction of the sterically hindered 2,2,6,6-tetramethylpiperidin-1-yl (**3s**) moiety did not increase the yields (66%).

Based on these results, **3u** was selected as the optimal catalyst for subsequent substrate scope studies. To benchmark its performance against established boronic acid catalysts, **3a**, **3b**, **3l**, and **3m** were subjected to the same model reaction between **1i** and **2a**.^13–15^ Under these conditions, **3u** afforded the highest yield and the fastest reaction rate (see Scheme S4 in the Supporting Information). However, note that the optimized conditions employed in this study differ from those reported in the original papers. Nevertheless, based on the literature yields for product **4o** (90%^13^, 50%^14^) and similar compounds,^15^ the benchmarked catalysts are not likely to be superior to **3u**.

After securing the optimal catalyst **3u**, reaction conditions were re-evaluated with the reaction between **1i** and **2a**. The aim was to identify a single set of reaction conditions applicable to both aliphatic and aromatic substrates. The re-evaluation revealed that catalyst loading could be lowered from 15 to 10 mol % without a change in the reaction rate or yield. However, reducing the amount of carboxylic acid **1i** from 1.25 equiv to 1.1 equiv led to diminished yield (79%). Investigation of the addition order showed that premixing **1i** with catalyst **3u** for a couple of minutes improved the reproducibility of the reaction, leading to more consistent conversions. Finally, increasing the concentration to 0.5 M further enhanced the reaction rate and afforded near-quantitative yield (99%).

Having identified the optimal catalyst and reaction conditions, we explored amidations between a broad range of aliphatic or aromatic carboxylic acids and amines (Scheme 4). In general, aliphatic carboxylic acids displayed higher reactivity than aromatic examples. Phenyl (**4a**), 4-chlorophenyl (**4b**), and 4-methoxyphenyl (**4c**) substrates afforded comparable results, leading up to 94% conversion. Surprisingly, cyclohexanecarboxylic acid (**1e**) gave **4e** in only 76% yield, while with cyclopentanecarboxylic acid (**1f**), the reaction proceeded cleanly to give a quantitative yield of **4f**. Aliphatic chains in the acid and amine side as well as secondary amines were well tolerated (**4d**, **4g**–**4l**). The reaction with **1g** and **2a** gave an excellent yield (**4m**, 94%), but the enantiomeric ratio remained moderate (*er* 82:18). Bifunctional amine, 2-aminopyridine (**2h**), gave moderate yield (**4n** 46%) when coupled with 2-(4-(trifluoromethyl)­phenyl)­acetic acid (**1h**). Furthermore, the reaction between 2-phenylacetic acid (**1a**) and 1-benzylpiperazine (**2d**) was run on a 6.61 mmol (1 g) scale with a Dean–Stark apparatus, leading to quantitative yield (**4i**), highlighting the potential for preparative applications.

**Scheme 4:**
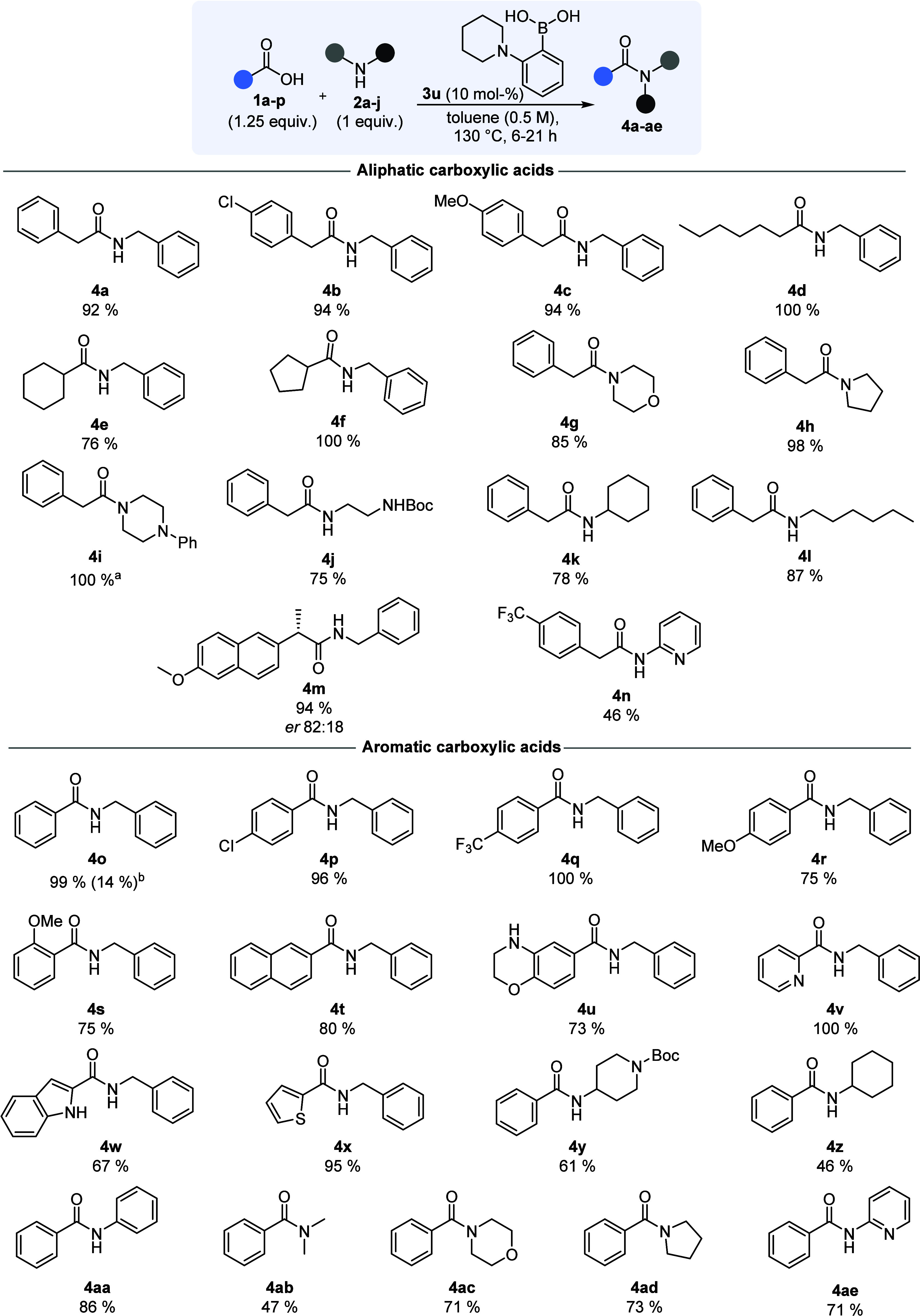
**3u** Catalyzed Amidation of Aliphatic and Aromatic Carboxylic Acids with Amines^a^,^b^

a Reaction done on a 1 g scale with Dean–Stark apparatus.
b Reaction without **3u** for 24 h.

The electron withdrawing groups (Cl, CF_3_) in the *para*-position of benzoic acids (**4p**, **4q**) resulted in almost quantitative yields, while the introduction of an electron donating methoxy-group both in the *para*- and *ortho*-positions (**4r**, **4s**) led to 75% yield. Nitrogen containing heterocycles exhibited varying reactivity: *N*-benzylpicolinamide (**4v**) was obtained in quantitative yield, while *N*-benzyl-1H-indole-2-carboxamide (**4w**) reached only a 67% yield. In general, modifications in the amine counterpart led to a slight decrease in the yields (**4y**–**4ae**). Surprisingly, aniline (**2j**) and 2-aminopyridine (**2h**) were well tolerated (**4aa** 86%, **4ae** 71%), whereas cyclohexyl amine led to only 46% of **4z**. In contrast, the introduction of Boc protected amine to the ring system led to an increase in the yield (**4y**, 61%), indicating good tolerance for Boc-protected amines.

The protocol was further extended to reactions involving protected amino acids (Scheme 5). To assess possible concomitant racemization, we prepared reference samples using racemic carboxylic acids or amines (see the Supporting Information for **GP2** and **GP3**). Despite the reflux conditions, the catalyst succeeded in avoiding major racemization with protected amino acids (**4af**–**4ai**). Reactions with Boc-l-proline (**1s**) afforded the best enantiomeric ratios, giving rise to **4af** (99%, *er* > 99:1), **4ah** (92%, *dr* > 99:1), and **4ai** (61%, *er* of 97:3). Boc-d-phenylalanine (**1t**) together with **2a** also afforded a good enantiomeric ratio, leading to **4ag** in 99% yield and an *er* of 95:5. Amidations with secondary amines led to lower conversions even with prolonged reaction times (**4ai** 61%, **4al** 54%, and **4am** 57%). Unfortunately, amino amides **4al** and **4am** exhibited notable racemization under the applied conditions.

**Scheme 5:**
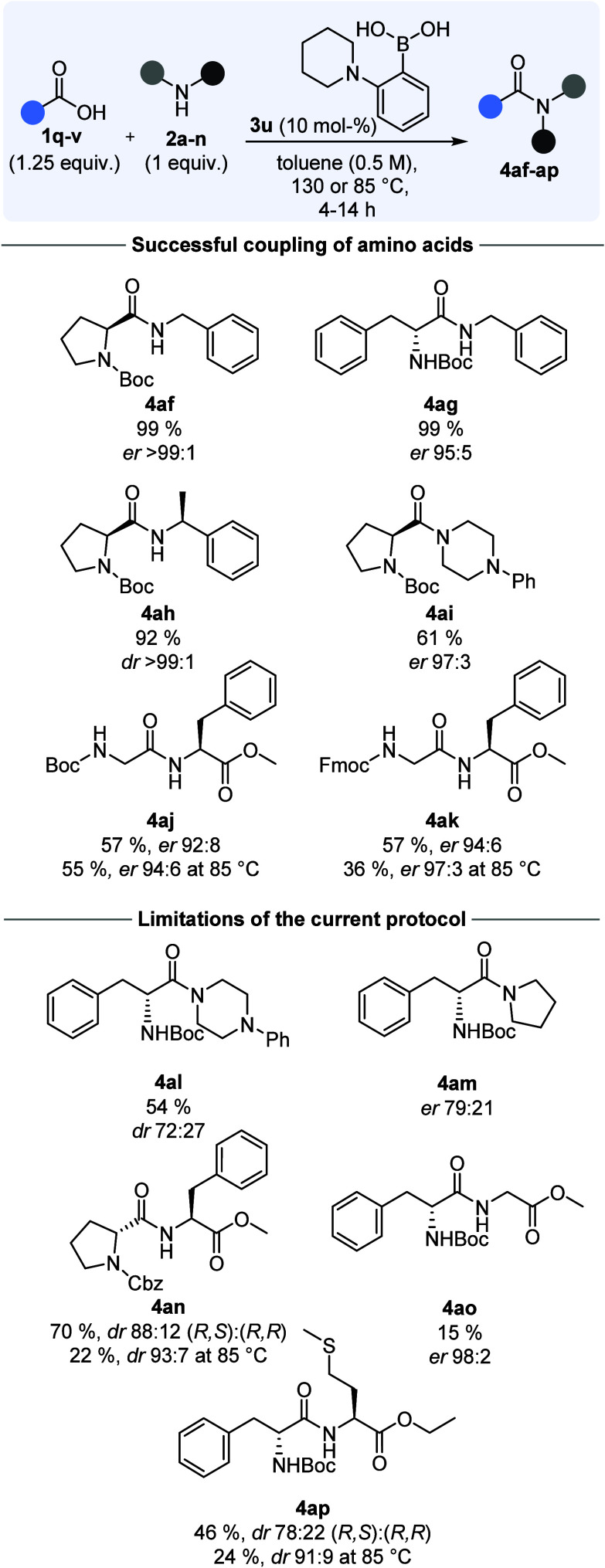
**3u** Catalyzed Amidation of Protected Amino Acids

We also explored the suitability of **3u** to the synthesis of dipeptides from protected amino acids (Scheme 5). In general, the catalytic synthesis of dipeptides with **3u** was hampered by low to moderate yields and significant racemization. In contrast, catalytic couplings of amino acids Boc-l-Pro (**1s**) and Boc-d-Phe (**1t**) with simple amines proceeded with little or no racemization (**4af**–**4ai**, Scheme 5), which indicated that the carboxylic acid component was less vulnerable toward racemization. Indeed, racemization among dipeptides (**4aj**, **4ak**, **4an**, and **4ap**) was observed at the C-terminus. As an example, coupling of Boc-(d,l)-Phe with l-Met-OEt (**2o**) using **3u** as the catalyst afforded a mixture of four diastereomers (10:10:40:40, d,d; l,d; d,l; l,l, respectively) indicating partial racemization of l-Met-OEt. When enantiopure Boc-d-Phe (**1t**) was used instead in coupling with l-Met-OEt (**2o**), the product was obtained as a diastereomeric mixture of Boc-d-Phe-l-Met-OEt and Boc-d-Phe-d-Met-OEt (**4ap**, 78:22, d,l;d,d, respectively). Similarly, Boc-Gly-l-Phe-OMe (**4aj**, 92:8, l:d), Fmoc-Gly-l-Phe-OMe (**4ak**, 94:6, l:d), and Cbz-d-Pro-l-Phe-OMe (**4an**, 88:12, d,l:d,d, respectively) suffered from racemization at the C-terminal amino acid.

We propose that the racemization observed in the dipeptide synthesis most likely precedes the condensation reaction and involves the free amino acid ester. This type of racemization has previously been reported by Takemoto and co-workers.^19^ If the racemization at the C-terminus would follow a similar pathway to the well-established oxazolone racemization pathway, it would require elimination and subsequent addition of the same alcohol.^30,31^ However, given the volatility of the eliminated alcohol, such a process is unlikely under the reaction temperature. Furthermore, no evidence of the oxazoline intermediate was observed during reaction monitoring by LC–MS.

Racemization with dipeptides (**4aj**, **4ak**, **4an**, **4ap**) could be partially suppressed by reducing the heater block temperature to 85 °C. However, this adjustment led to a substantial decrease in the conversions, with isolated yields dropping to approximately 20–50% (Scheme 5). The low-yielding amino acid couplings were also repeated in fluorobenzene (85 °C), 1:1 mixtures of toluene:Me-THF and toluene:EtOAc (80 °C), and trifluorotoluene (105 °C), but even lower yields were obtained (<20%). In addition, we suspected that the lower yields observed with dipeptides (**4aj**, **4ak**, **4an**, **4ao**, and **4ap**) were due to catalyst inhibition by the product. However, the addition of dipeptide **4aj** to a well-performing reaction between **1i** and **2a** did not inhibit the reaction, and an identical yield of **4o** was observed (99%, see the Supporting Information). Moreover, no signs of product degradation were observed in the reaction monitoring experiments.

It is evident that the high reflux temperature contributes significantly to the level of racemization. However, efficient water removal is well documented as being crucial for the catalytic activity of boronic acids.^15,16,32^ We likewise observed that at lower reflux temperatures (<130 °C in toluene), lower conversions were observed likely because water was not removed efficiently (Table 1, entries 4–6; Scheme 4). The sigmoidal shape of the reaction progress curves (Scheme 2, Schemes S2–S4) also suggests that either a sufficient amount of water must be removed before the reaction can proceed or that the boronic acid serves only as a precatalyst and not as the active species.^15,32^

In principle, the commonly employed water removal protocol, where dry DCM is used in combination with molecular sieves, could allow reactions to proceed under sufficiently dry conditions at lower temperatures. However, carrying out the reaction between **1a** and **2a** in dry DCM with either 4 Å or 5 Å molecular sieves (predried in a vacuum oven at 300 °C) led to no conversion, and using powdered molecular sieves did not help either. Similarly, the addition of molecular sieves to experiments with amino acid substrates (**4aj**, **4ak**, **4an**, and **4ap**) carried out at 85 °C in toluene or fluorobenzene failed to increase the yields. Previous reports indicate that effective activation of molecular sieves often requires heating at higher temperatures, for example, in a furnace oven.^13^ The challenges associated with using molecular sieves render the approach unattractive, especially on a larger scale.

As discussed earlier, dipeptide products do not degrade under the reaction conditions, nor do they display signs of product inhibition. Considering these results, along with the observed racemization and the need for high reaction temperatures (>85 °C), it appears unlikely that the limitations associated with dipeptide substrates can be fully addressed by further optimization of the reaction conditions alone. Rather, the development of alternative catalysts beyond simple boronic acids might be required to address these issues in the future. In this context, more complex multiboron catalysts have shown promising performance in dipeptide synthesis with minimal racemization.^12,19,33^ We hope that the simple structure of catalyst **3u** identified herein could serve as the starting point for the future evolution of even more selective catalysts.

## Conclusions

Herein, we present a structurally simple and readily accessible catalyst, 2-PPBA (**3u**), for the direct amidation of carboxylic acids with amines. The catalytic protocol obviates the use of halogenated solvents and additional drying agents, enabling amidation reactions in a more atom-economical, sustainable, and operationally straightforward manner. Racemization of the product was generally not significant under the reaction conditions with α-chiral carboxylic acids; however, in the synthesis of dipeptides, the amine donor components were often partially epimerized. Nevertheless, given the simplicity of the catalyst (only a single boronic acid functionality) and the high activity of the catalyst in benchmark experiments, this study offers an attractive alternative to classical amide coupling methods for a wide variety of substrates.

## Materials and Methods

Further information on the experimental procedures, catalyst screening, and characterization data are provided in the Supporting Information.

### General Procedure for Amidation with Aliphatic and Aromatic Carboxylic Acids and Amines

Carboxylic acid **1** (0.625 mmol, 1.25 equiv) and (2-piperidin-1-ylphenyl)­boronic acid **3u** (0.05 mmol, 0.1 equiv) were dissolved in dry toluene (1 mL) and stirred for 10 min in a vial sealed with septum. Amine **2** (limiting reagent, 0.5 mmol, 1 equiv) was added, and the mixture was stirred under reflux conditions on an aluminum heating block for 3–21 h depending on the substrates. The temperature of the aluminum heating block was set to 130 °C. In the small-scale reaction, the generated water condensed and remained on the vial walls during reflux. After the indicated reaction time, the reaction mixture was allowed to cool to rt and concentrated under reduced pressure. The products were purified by flash chromatography.

## Supplementary Material





## Data Availability

The data underlying this study are available in the published article and its Supporting Information.

## Notes

Experimental procedures, optimization, characterization data, and copies of NMR spectra and HPLC chromatograms (PDF)
FAIR Data is available as Supporting Information for Publication and includes the primary NMR FID files for compounds **4a**–**4ap**, **3s**, **3t**, **S1a**, and **S1b** (ZIP)
