# Supplementary material for: Catalytic Amidation of Carboxylic Acids Using ortho-Aminophenylboronic Acids
Source: ACS Org Inorg Au. 2026 May 12;6(4):422–9. doi: 10.1021/acsorginorgau.6c00021 (PMC13449423; doi:10.1021/acsorginorgau.6c00021)
Supplement: Supplementary file 1 [file gg6c00021_si_001.pdf]

Supporting Information

Catalytic Amidation of Carboxylic Acids Using Ortho-Aminophenylboronic Acids

Veera K. Bruce-Salmenkivi[†], Mikko Passiniemi[‡], Petri M. Pihko[†]*

[†]Department of Chemistry and NanoScience Center, University of Jyväskylä, P.O. Box 35, FI-40014, Jyväskylä, Finland
[‡] Orion Pharma, P.O. Box 65 (Orionintie 1), FI-02200 Espoo, Finland

petri.pihko@jyu.fi

Contents

1 General Information 4
2 Boronic acid catalyst screening.. 5
2.1 General procedure (GP1) for catalyst screening studies 5
2.2 Catalyst screening.. 5
2.3 Benchmarking.. 8
3 Spiking experiment with methyl methyl (*tert*-butoxycarbonyl)glycyl-(*S*)-phenylalaninate, 4aj 9
4 Experimental details 12
4.1 Numbering of starting materials 12
4.2 General procedures for the amidation reactions 13
4.2.1 *N*-Benzyl-2-phenylacetamide **4a**, MC020-313 14
4.2.2 *N*-Benzyl-2-(4-chlorophenyl)acetamide **4b**, MC020-327 14
4.2.3 *N*-Benzyl-2-(4-methoxyphenyl)acetamide **4c**, MC020-333 15
4.2.4 *N*-Benzylheptanamide **4d**, MC020-326 15
4.2.5 *N*-Benzylcyclohexanecarboxamide **4e**, MC020-336 16
4.2.6 *N*-Benzylcyclopentanecarboxamide **4f**, MC020-337 16
4.2.7 1-Morpholino-2-phenylethan-1-one **4g**, MC020-325............................ 17
4.2.8 2-Phenyl-1-(pyrrolidin-1-yl)ethan-1-one **4h**, MC020-356 17
4.2.9 2-Phenyl-1-(4-phenylpiperazin-1-yl)ethan-1-one **4i**, MC020-345/362 17
4.2.10 *tert*-Butyl (2-(2-phenylacetamido)ethyl)carbamate **4j**, MC020-334........ 18
4.2.11 *N*-Cyclohexyl-2-phenylacetamide **4k**, MC020-354 18
4.2.12 *N*-Hexyl-2-phenylacetamide **4l**, MC020-331 19
4.2.13 (*S*)-*N*-Benzyl-2-(6-methoxynaphthalen-2-yl)propenamide **4m**, MC020-350 19
4.2.14 N-(pyridin-2-yl)-2-(4-(trifluoromethyl)phenyl)acetamide **4n**, TJB-2-008.. 20
4.2.15 *N*-Benzylbenzamide **4o**, MC020-294 20
4.2.16 *N*-Benzyl-4-chlorobenzamide **4p**, MC020-295 20
4.2.17 *N*-Benzyl-4-(trifluoromethyl)benzamide **4q**, MC020-357 21
4.2.18 *N*-Benzyl-4-methoxybenzamide **4r**, MC020-296.................................. 21
4.2.19 *N*-Benzyl-2-methoxybenzamide **4s**, MC020-358 22
4.2.20 *N*-Benzyl-2-naphthamide **4t**, MC020-307 22

4.2.21 *N*-Benzyl-3,4-dihydro-2H-benzo[b][1,4]oxazine-6-carboxamide **4u**, MC020-298 23

4.2.22 *N*-Benzylpicolinamide **4v**, MC020-280 23

4.2.23 *N*-Benzyl-1*H*-indole-2-carboxamide **4w**, MC020-297 24

4.2.24 *N*-Benzylthiophene-2-carboxamide **4x**, MC020-304 24

4.2.25 *tert*-Butyl 4-benzamidopiperidine-1-carboxylate **4y**, MC020-303 25

4.2.26 *N*-Cyclohexylbenzamide **4z**, MC020-315 25

4.2.27 *N*-Phenylbenzamide **4aa**, MC020-316 26

4.2.28 *N,N*-Dimethylbenzamide **4ab**, MC020-308 26

4.2.29 Morpholino(phenyl)methanone **4ac**, MC020-291 26

4.2.30 Phenyl(pyrrolidin-1-yl)methanone **4ad**, MC020-288 27

4.2.31 N-(pyridin-2-yl)benzamide **4ae**, TJB-2-010 27

4.2.32 *tert*-Butyl (*S*)-2-(benzylcarbamoyl)pyrrolidine-1-carboxylate **4af**, MC020-338 28

4.2.33 *tert*-Butyl (*R*)-(1-(benzylamino)-1-oxo-3-phenylpropan-2-yl)carbamate **4ag**, MC020-317 28

4.2.34 *tert*-Butyl (*S*)-2-(((*S*)-1-phenylethyl)carbamoyl)pyrrolidine-1-carboxylate **4ah**, MC020-363, MC030-370 29

4.2.35 *tert*-Butyl (*S*)-2-(4-benzylpiperazine-1-carbonyl)pyrrolidine-1-carboxylate **4ai**, MC020-366 29

4.2.36 Methyl (*tert*-butoxycarbonyl)glycyl-(*S*)-phenylalaninate, **4aj**, MC020-410 30

4.2.37 Methyl (((9H-fluoren-9-yl)methoxy)carbonyl)glycyl-(*S*)-phenylalaninate **4ak**, MC020-388, MC020-406 30

4.2.38 *tert*-Butyl (*R*)-(1-oxo-3-phenyl-1-(4-phenylpiperazin-1-yl)propan-2-yl)carbamate **4al**, MC020-374 31

4.2.39 *tert*-Butyl (*R*)-(1-oxo-3-phenyl-1-(pyrrolidin-1-yl)propan-2-yl)carbamate **4am**, MC020-368, MC020-375 32

4.2.40 Benzyl (*R*)-2-(((*S*)-1-methoxy-1-oxo-3-phenylpropan-2-yl)carbamoyl)pyrrolidine-1-carboxylate **4an**, MC020-384, MC020-405 32

4.2.41 (*R*)-Methyl 2-(2-(*tert*-butoxycarbonylamino)-3-phenylpropanamido)acetate **4ao**, MC020-383 33

4.2.42 Methyl (*tert*-butoxycarbonyl)-(*R)*-phenylalanyl-*(S)*-methioninate **4ap**, MC020-389, MC020-409 34

4.3 Synthesis of boronic acids 35
4.3.1 1-(2-Iodophenyl)-2,2,6,6-tetramethylpiperidine **S1a**, MC020-293 35
4.3.2 1-(2-Iodo-4-methoxyphenyl)piperidine **S1b**, MC020-343 36
4.3.3 (2-(2,2,6,6-Tetramethylpiperidin-1-yl)phenyl)boronic acid **3s**, MC020-306 36
4.3.4 (5-Methoxy-2-(piperidin-1-yl)phenyl)boronic acid **3t**, MC020-349........ 37
5 References 38
6 Copies of NMR spectra and HPLC chromatograms 42

1 General Information

All reactions were carried out in open atmosphere unless otherwise noted. When needed, nonaqueous reagents were transferred under nitrogen via syringe and dried prior to use. THF, toluene and DCM were obtained by passing deoxygenated solvents through activated alumina columns (MBraun SPS-800 Series solvent purification system). Other solvents and reagents were used as obtained from supplier, unless otherwise noted. The reactions were cooled with acetone-dry ice mixture (external bath temperature –78 °C). The heating in reactions was done with an aluminum block.

Analytical TLC was performed using Merck silica gel F254 (230-400 mesh) plates and analyzed by UV light or by staining upon heating with $KMnO_4$ solution (1 g $KMnO_4$, 6.7 g K_2CO_3, 1.7 mL 1M NaOH, 100 mL H_2O). For silica gel chromatography, CombiFlash R_f 200 with RediSep Gold or Silver columns (20–40 µm spherical silica, 400– 632 mesh) were used with p.a. grade solvents unless otherwise noted.

The ^1H NMR and $^{13}C\{^1H\}$ NMR spectra were recorded in either $CDCl_3$ or $(CD_3)_2SO$ on Bruker Avance 400 or 600 MHz spectrometers. The chemical shifts are reported in ppm relative to $CHCl_3$ (δ 7.26) or DMSO-d_6 (2.50) for ^1H NMR. For the $^{13}C\{^1H\}$ NMR spectra, the residual $CDCl_3$ (δ 77.16), or $(CD_3)_2SO$ (39.52) were used as the internal standards. Melting points (mp) were determined in open capillaries using Buchi melting point B-545 apparatus. IR spectra were recorded on a Tensor27 FT-IR spectrometer. High resolution mass spectrometric data were measured using Waters Acquity UPLC with PDA and Xevo G2-XS Q-TOF mass spectrometer. LC-MS data was measured using Waters Acquity UPLC system with a Waters Acquity UPLC Photo Diode Array detector and a Single Quadrupole detector. The enantiomeric and diastereomeric ratios were determined using Waters Acquity UPC2 SFC Feeniks system.

2 Boronic acid catalyst screening

2.1 General procedure (GP1) for catalyst screening studies

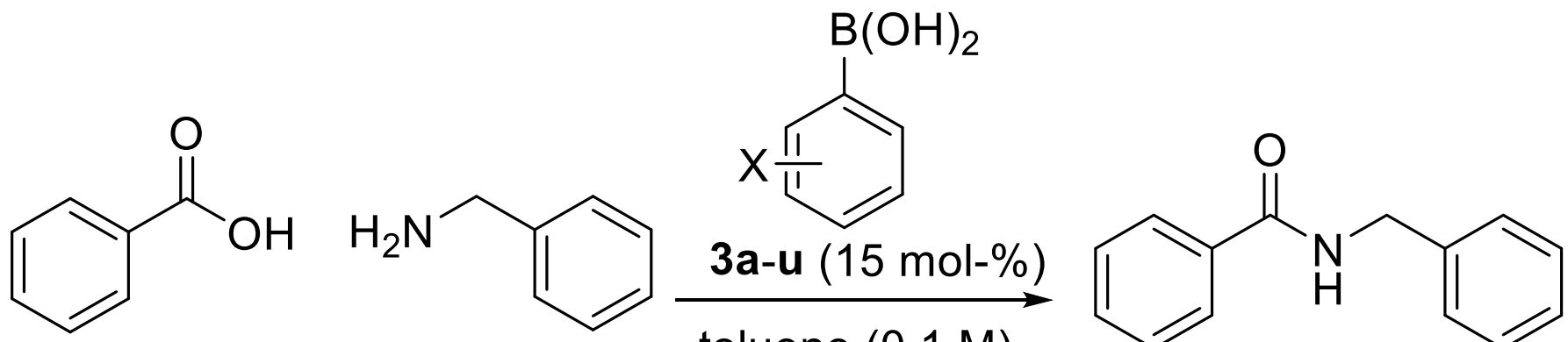

General procedure 1 (GP1): Benzoic acid **1i** (38.4 mg, 0.275 mmol, 1.25 equiv.), 1,3,5-trimethoxybenzene (42.1 mg, 0.25 mmol, 1 equiv.), boronic acid **3a-u** (0.04 mmol, 0.15 equiv.) and amine **2a** (27.6 µL, 0.25 mmol, 1 equiv.) was dissolved in dry toluene (2.50 mL) and the mixture was stirred at 130 °C (heater block temperature) on an aluminum heater block. Samples for LC-MS analysis were taken periodically every hour, and the chromatogram's peak areas were converted to concentrations using calibration curves and 1,3,5-trimethoxybenzene as an internal standard.

2.2 Catalyst screening

Screening of boronic acids was done with **GP1**, following the concentration of the product **4o**. A variety of sulfur- and oxygen-containing, as well as other heterocyclic boronic acids, were initially examined as the catalyst for the amidation (Scheme S2). Notably, *ortho*-substituted boronic acids, such as compounds **3c** and **3d** exhibited the highest activity, giving results comparable to those obtained with Su's catalyst **3a**.[1]

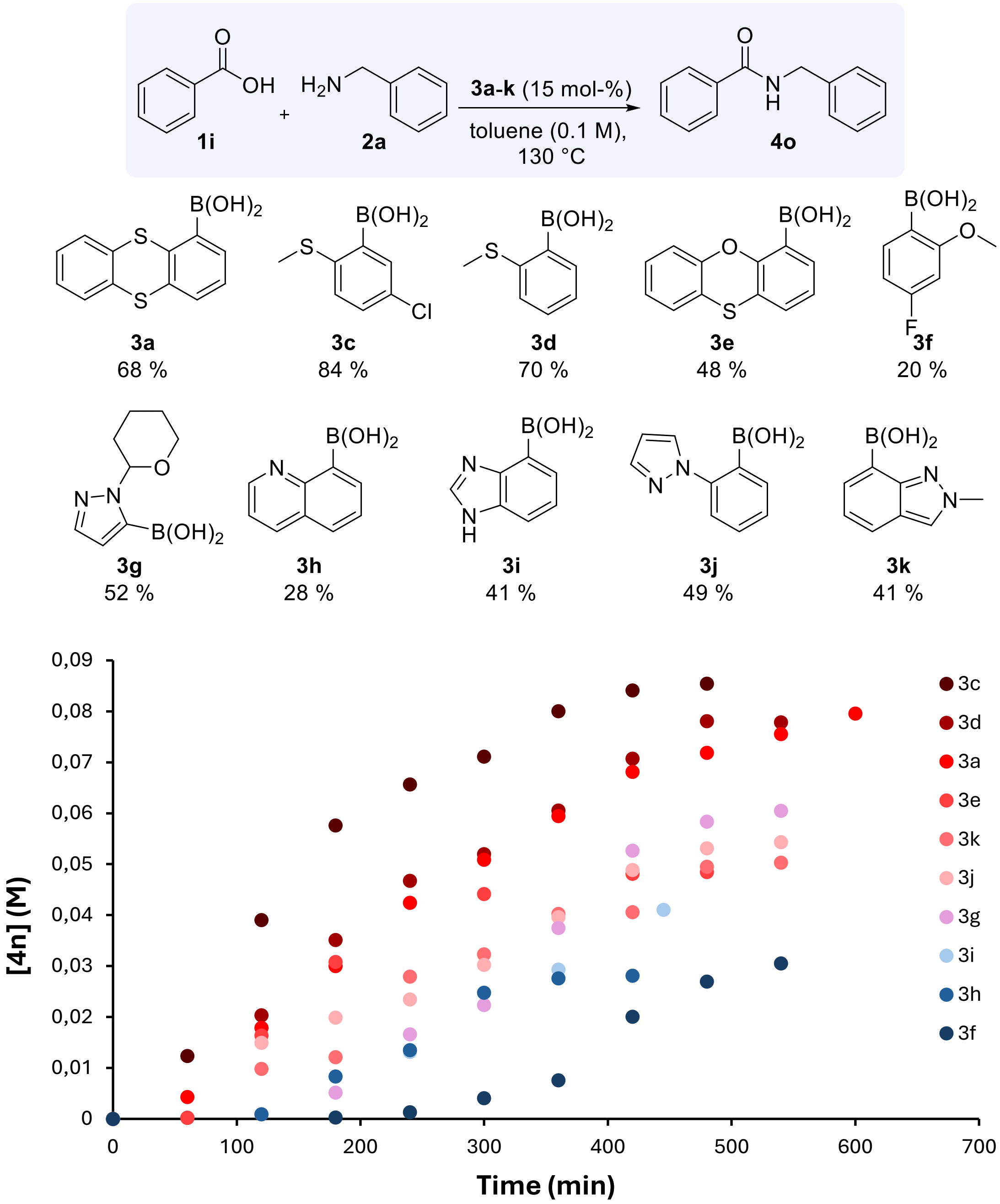

Scheme S2. Reaction progress plots from the reactions between **1i** and **2a** following the concentration of product **4o**. The plots are color-coded based on the catalyst used (**3a**–**3k**). The yields of product **4o** are recorded after 7 h.

Guided by the first round of catalyst screening, a scope of nitrogen containing *ortho*-substituted phenylboronic acids (**3n**–**3u**) were explored using **GP1** (Scheme S3). Among the catalysts evaluated, **3u** exhibited the highest activity.

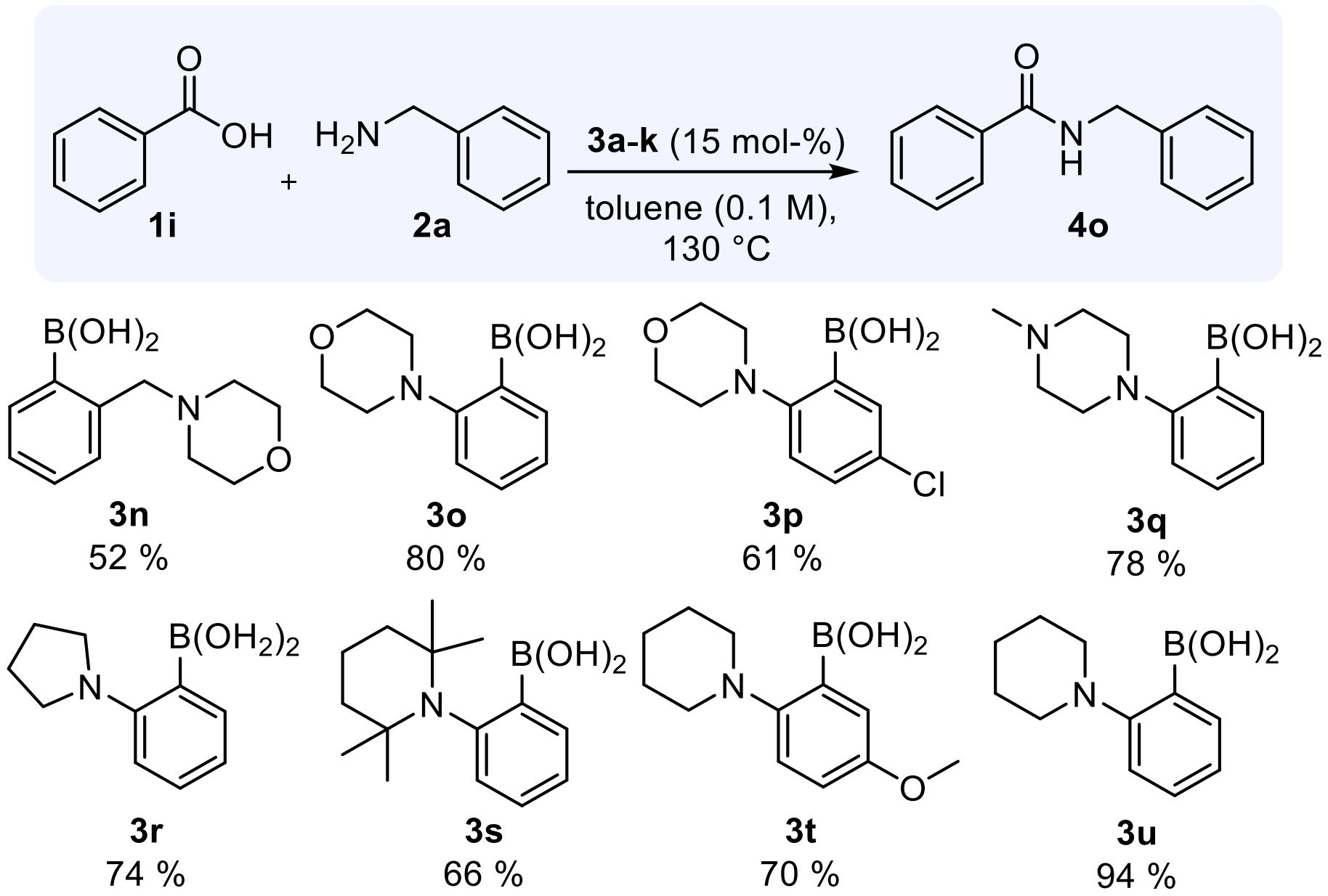

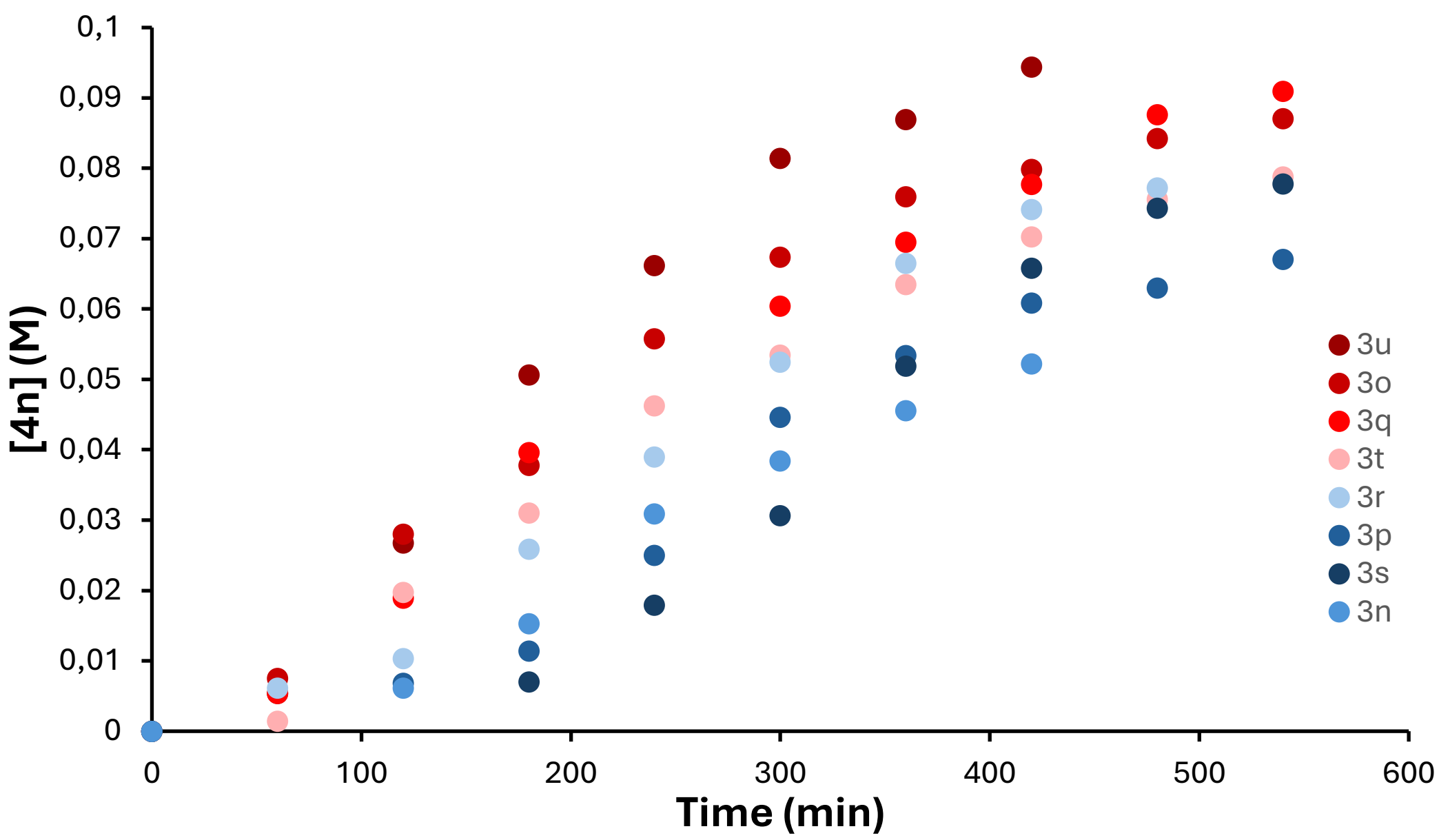

Scheme S3. Reaction progress plots from the reactions between **1i** and **2a** following the concentration of product **4o**. The plots are color-coded based on the catalyst used (**3n–3u**). The yields of product **4o** are recorded after 7 h.

2.3 Benchmarking

To confirm the desired enhancement achieved with boronic acid **3u**, it was compared to previously reported boronic acid catalysts **3a**, **3b**, **3l**, **3m** following the **GP1** (Scheme S4).[2–4] The results proved that (2-(piperidin-1-yl)phenyl)boronic acid **3u** remained the most effective, delivering the highest yields within the given reaction time.

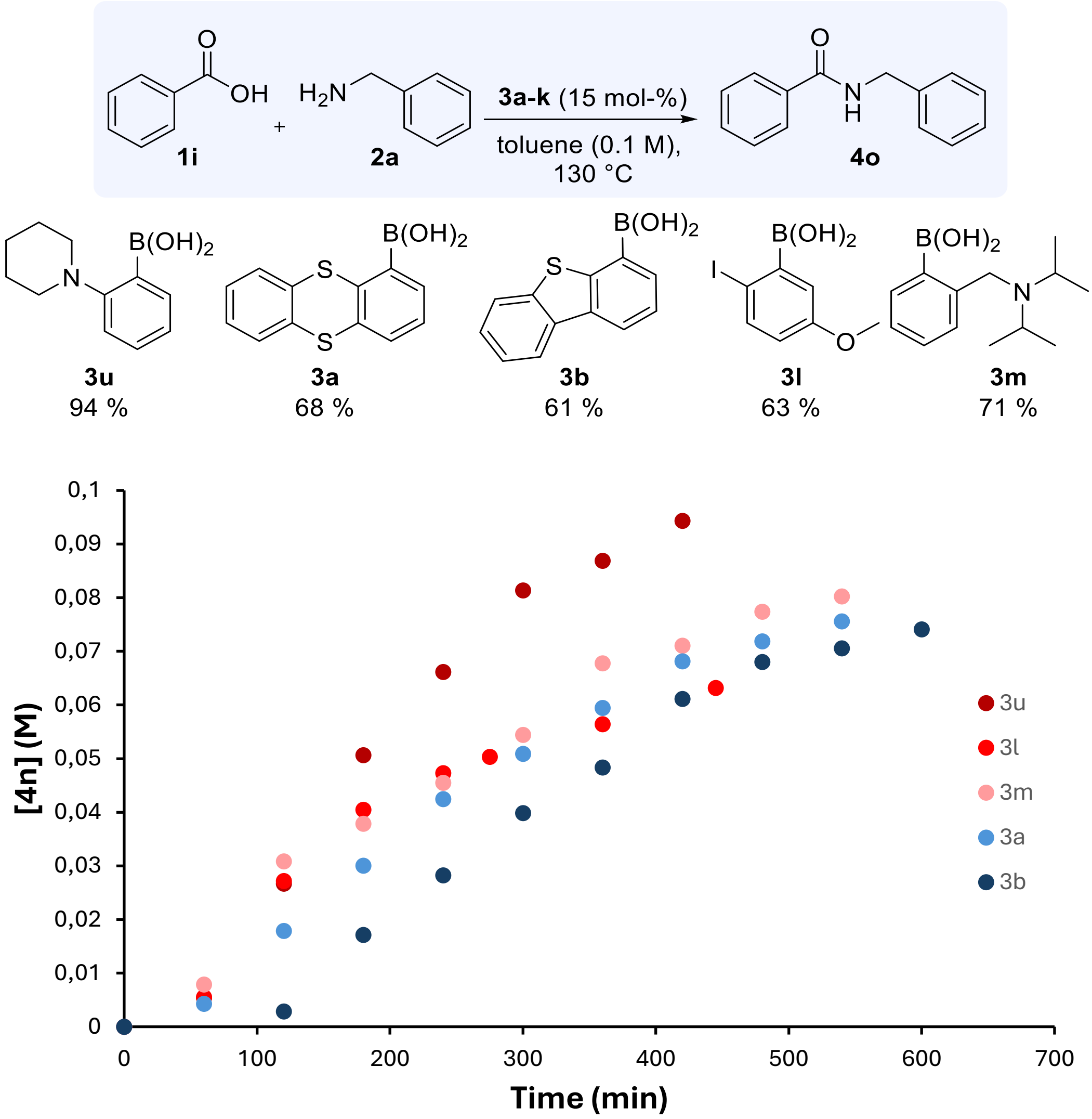

Scheme S4. Comparison of **3a**, **3b**, **3l**, **3m** to the boronic acid **3u**. The plots are color-coded based on the catalyst used. The yields of product **4o** are recorded after 7 h.

3 Spiking experiment with methyl methyl (*tert*-butoxycarbonyl)glycyl-(*S*)-phenylalaninate, 4aj

Due to the low yields observed in reactions involving methyl glycinate (**4ao**), a control experiment was designed to investigate potential product inhibition. In the experiment, methyl (*tert*-butoxycarbonyl)-*S*-phenylalanylglycinate **4aj** was added into a well-performing reaction between benzoic acid **1i** and benzyl amine **2a**. Two identical reactions were carried out (**1i** 1.25 equiv., **2a** 1 equiv., **3u** 10 mol-% in toluene at 130 °C), with one receiving an additional 20 mol-% of **4aj**. The progress of both reactions was monitored using acidic LC-MS. The resulting chromatograms (Figures S1–S4) clearly indicate that both reactions proceeded similarly, with no evidence of product inhibition.

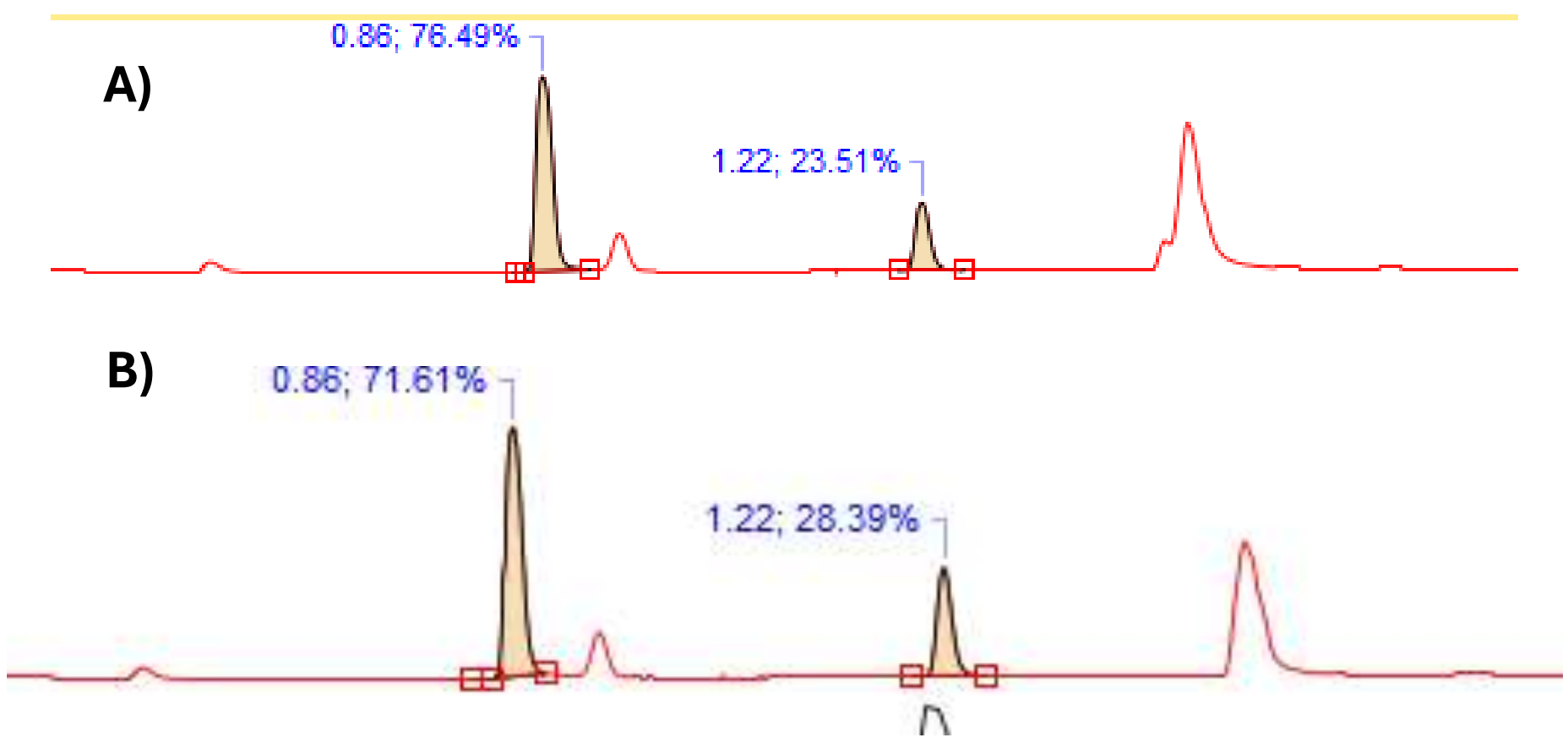

Figure S1. Chromatograms of test reactions after 1 h. A) corresponds to the reaction with 20 mol-% of **4aj** added, and B) to the reference reaction without any added dipeptide. The Peak at 0.86 min corresponds to **1i** and the peak at 1.22 min corresponds to the product **4o**.

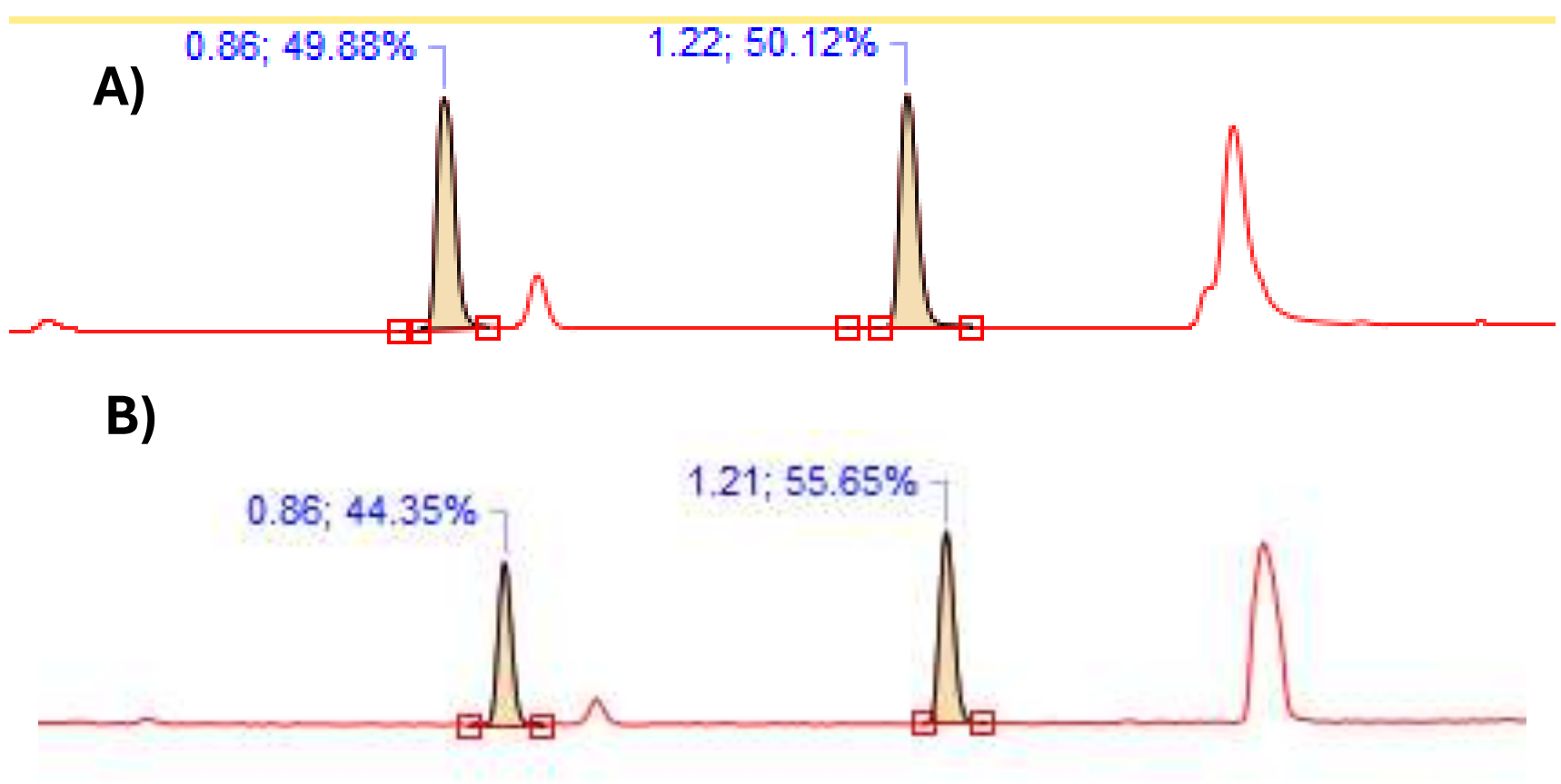

Figure S2. Chromatograms of test reactions after 2 h. A) corresponds to the reaction with 20 mol-% of **4aj** added, and B) to the reference reaction without any added dipeptide. The Peak at 0.86 min corresponds to **1i** and the peak at 1.22 min corresponds to the product **4o**.

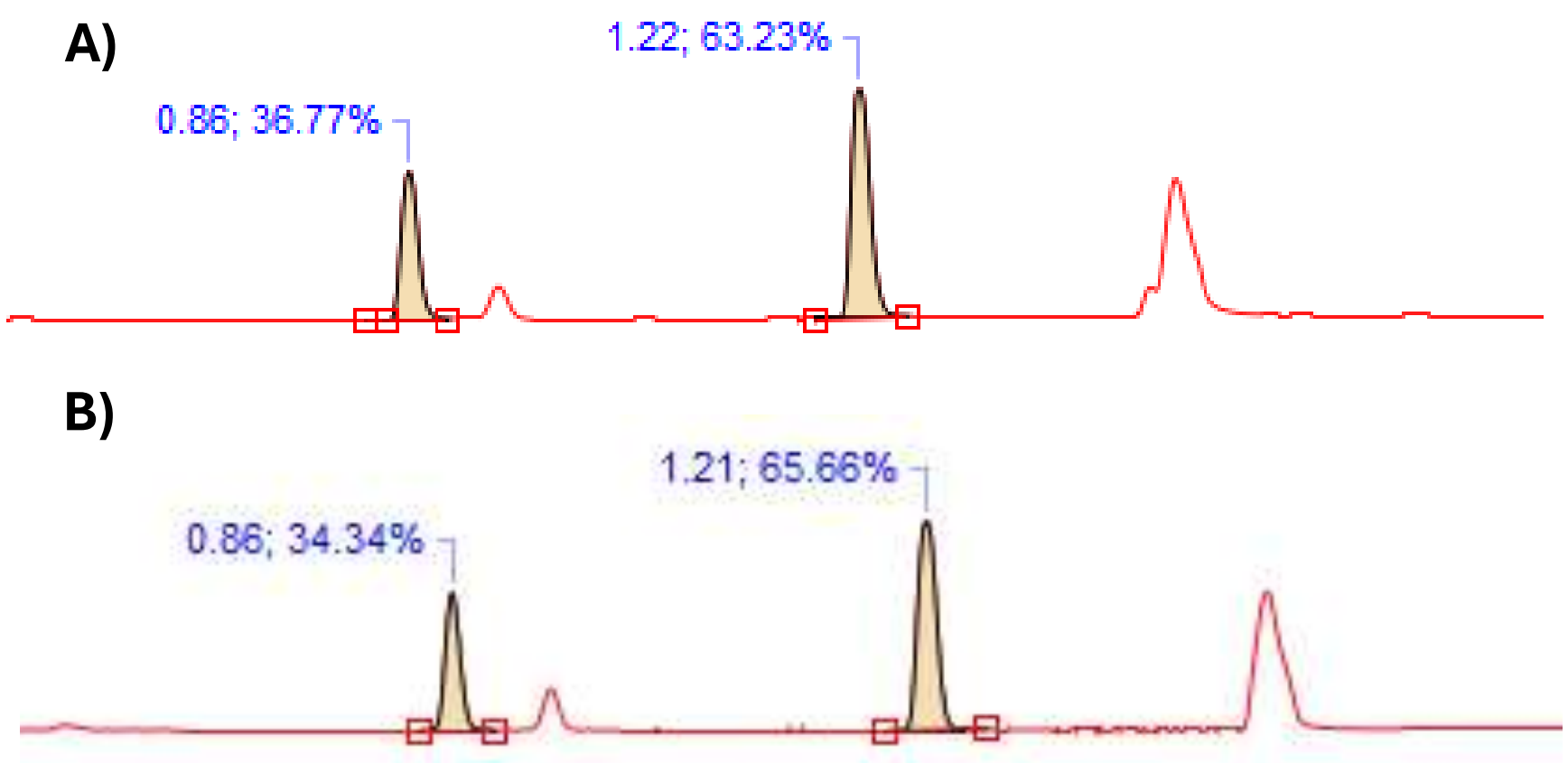

Figure S3. Chromatograms of test reactions after 4 h. A) corresponds to the reaction with 20 mol-% of **4aj** added, and B) to the reference reaction without any added dipeptide. The Peak at 0.86 min corresponds to **1i** and the peak at 1.22 min corresponds to the product **4o**.

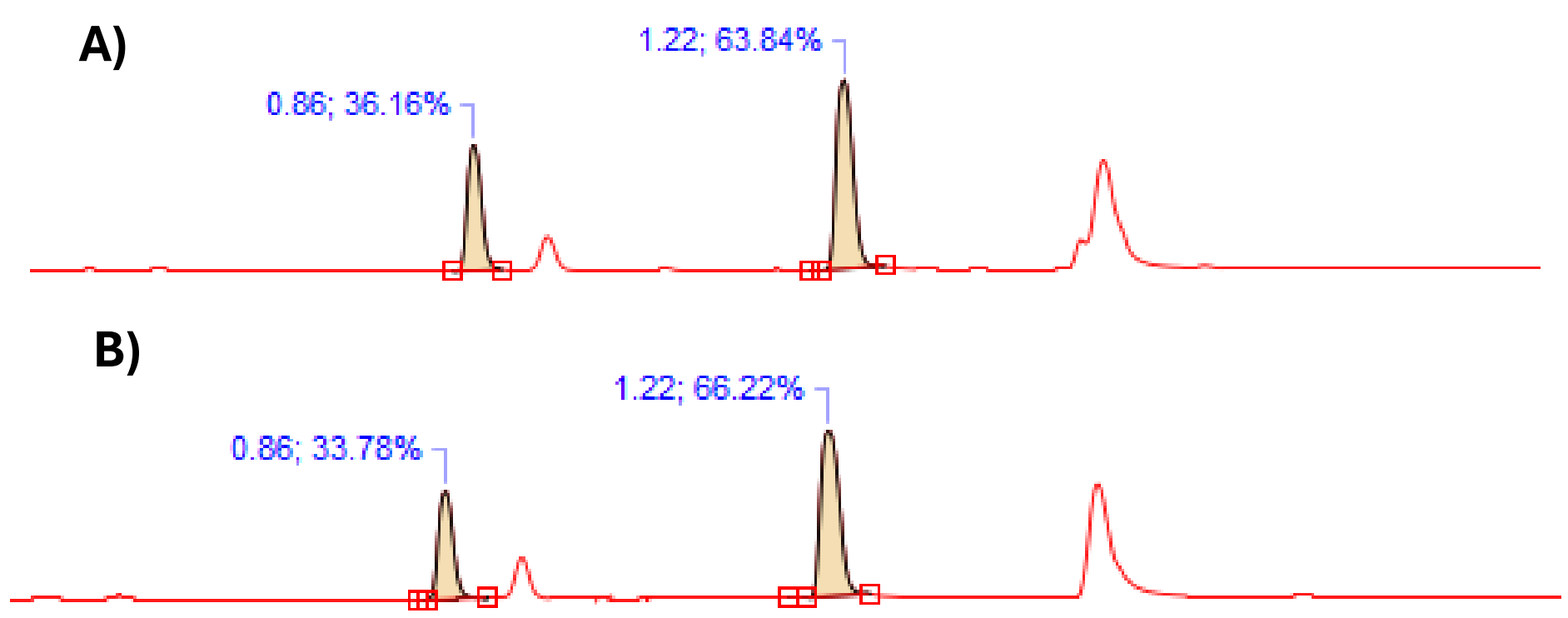

Figure S4. Chromatograms of test reactions after 6 h. A) corresponds to the reaction with 20 mol-% of **4aj** added, and B) to the reference reaction without any added dipeptide. The Peak at 0.86 min corresponds to **1i** and the peak at 1.22 min corresponds to the product **4o**.

4 Experimental details

4.1 Numbering of starting materials

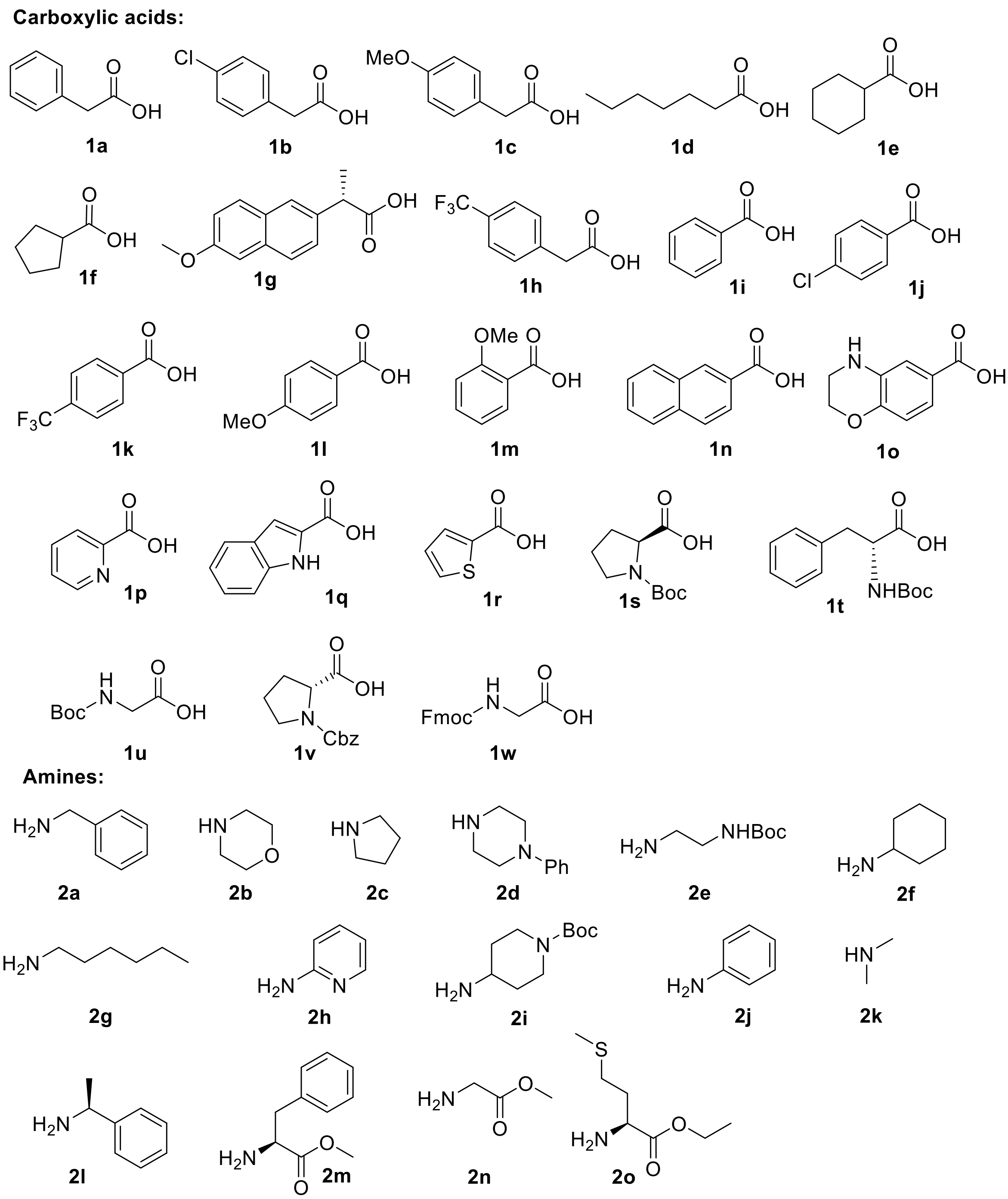

Scheme S5. Numbering of starting materials.

4.2 General procedures for the amidation reactions

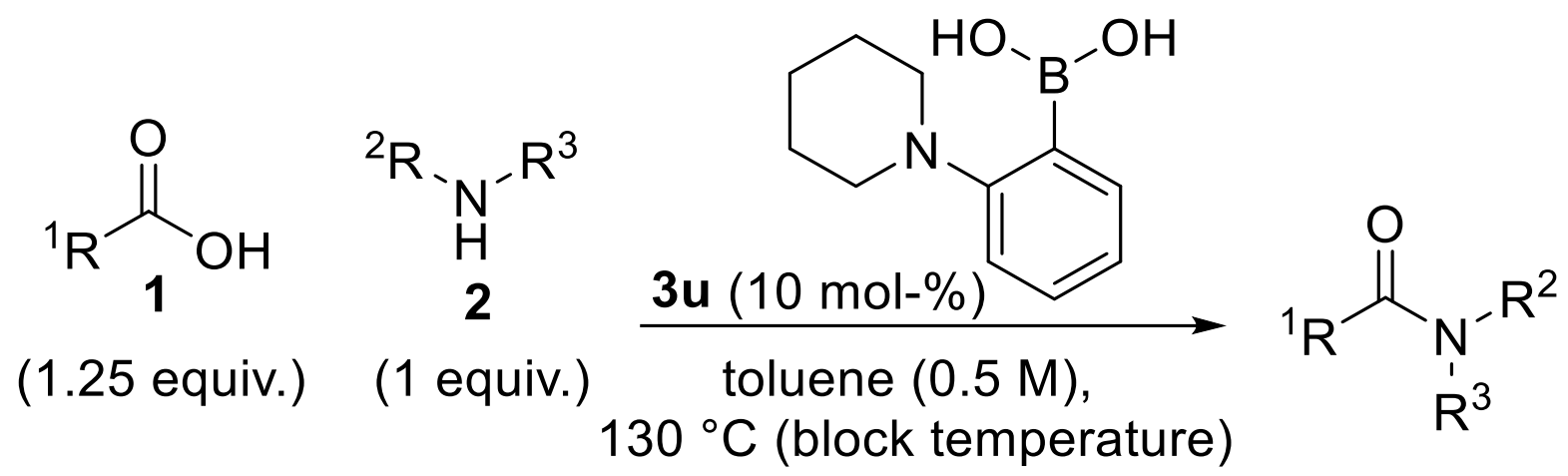

General procedure 2 (GP2): Carboxylic acid **1a–t** (0.625 mmol, 1.25 equiv.) and (2-piperidin-1-ylphenyl)boronic acid **3u** (0.05 mmol, 0.1 equiv.) was dissolved in dry toluene (1 mL) and stirred for 10 min. Amine **2a–l** (limiting reagent, 0.5 mmol, 1 equiv.) was added and the mixture was stirred under reflux conditions on an aluminum heater block for 3–21 h depending on the substrates. The temperature of the aluminum heater block was set to 130 °C. After the indicated reaction time, the reaction mixture was allowed to cool down to rt and concentrated under reduced pressure. The products were purified by flash chromatography. Reference samples of products **4m**, **4af**, **4ag**, **4ah**, **4ai**, **4al** and **4am** for enantiomeric and diastereomeric purity measurements were prepared using **GP2** with racemic carboxylic acid.

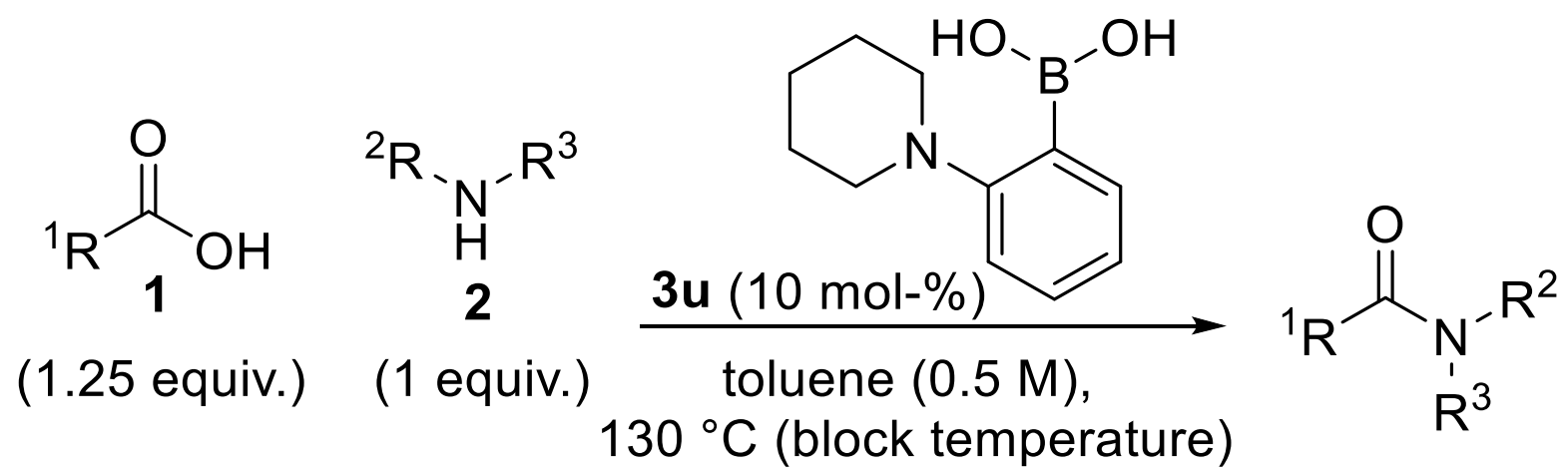

General procedure 3 (GP3): The hydrochloride salts of amino acid esters were treated with 10 % Na_2CO_3 (aq). The water phase was extracted with DCM (3 x 20 mL), dried using phase separator and concentrated under reduced pressure. Carboxylic acid **1s–w**(0.625 mmol, 1.25 equiv.) and (2-piperidin-1-ylphenyl)boronic acid **3u** (0.05 mmol, 0.1 equiv.) were dissolved in dry toluene (1 mL) and stirred for 10 min. The free amino acid ester **2a–o** (limiting reagent, 0.5 mmol, 1 equiv.) was added and the mixture was stirred under reflux conditions on an aluminum heater block for 7–10 h depending on the substrates. The temperature of the aluminum block was set either to 130 °C or 85 °C. After the indicated reaction time, the reaction mixture was allowed to cool down to rt and concentrated under reduced pressure. The products were purified by flash chromatography. Reference samples of products **4aj**, **4ak**, **4an**, **4ao** and **4ap** for enantiomeric and diastereomeric purity measurements were prepared using **GP3** with racemic carboxylic acid or amine.

4.2.1 *N*-Benzyl-2-phenylacetamide **4a**, MC020-313

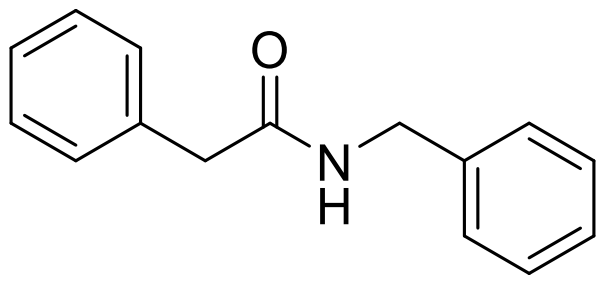

Prepared following **GP2** using **1a** (85.1 mg, 0.625 mmol) and **2a** (53.6 mg, 0.5 mmol) with 6 h reaction time. The residue was purified by flash chromatography (gradient heptane-EtOAc 100:0 to 53:47) to give **4a** as a white solid (103 mg, 0.46 mmol, 92 %). Spectral data corresponds to previously published data.[5]

mp 114-117 °C; R_f (EtOAc-Hept 40:60) = 0.38; IR (neat, ATR) ν_{max} 465, 601, 691, 1001, 1064, 1108, 1431, 1583, 1634, 2925, 3031, 3063, 3084, 3283; ^{1}H NMR (400 MHz, DMSO-d_6): δ 8.55 (br t, *J* = 5.2 Hz, 1H), 7.25-7.32 (m, 6H), 7.20-7.25 (m, 4H), 4.26 (d, *J* = 5.9 Hz, 2H), 3.47 (s, 2H); ^{13}C{^{1}H} NMR (101 MHz, DMSO-d_6): δ 170.2, 139.5, 136.4, 129.0, 128.3, 128.3, 127.2, 126.8, 126.4, 42.4, 42.2.

4.2.2 *N*-Benzyl-2-(4-chlorophenyl)acetamide **4b**, MC020-327

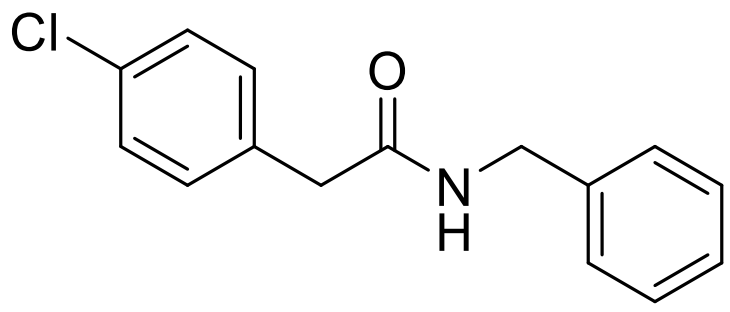

Prepared following **GP2** using **1b** (106.6 mg, 0.625 mmol) and **2a** (53.6 mg, 0.5 mmol) with 6 h reaction time. The residue was purified by flash chromatography (gradient heptane-EtOAc 100:0 to 67:33) to give **4b** as a white solid (122 mg, 0.47 mmol, 94 %). Spectral data corresponds to previously published data.[6]

mp 123-125 °C; R_f (EtOAc-Hept 60:40) = 0.51; IR (neat, ATR) ν_{max} 435, 448, 691, 738, 999, 1038, 1243, 1366, 1541, 1642, 2871, 2920, 3027, 3056, 3271; ^{1}H NMR (400 MHz, $CDCl_3$) δ 7.44 – 7.35 (m, 2H), 7.34 – 7.21 (m, 7H), 5.80 (s, 1H), 4.45 (d, *J* = 5.8 Hz, 2H), 3.76 (s, 2H); ^{13}C{^{1}H} NMR (101 MHz, $CDCl_3$): δ 169.7, 138.2, 134.5, 133.1, 131.9, 130.0, 129.1, 128.8, 127.6, 127.6, 127.5, 43.8, 41.6; HRMS (ESI+): m/z calcd for $[C_{15}H_{14}ClNO+H]^+$ = 260.0842, found 260.0879, Δ = 14.2 ppm.

4.2.3 *N*-Benzyl-2-(4-methoxyphenyl)acetamide **4c**, MC020-333

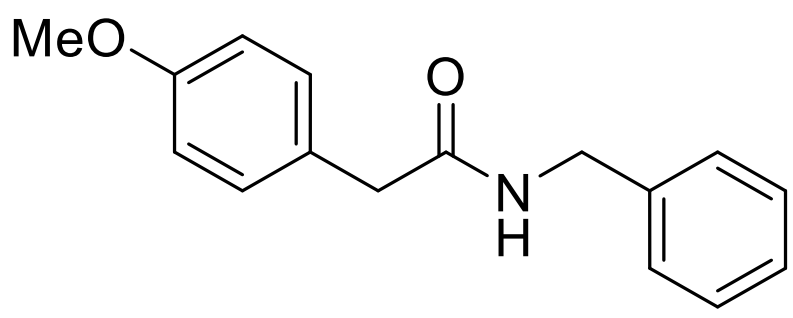

Prepared following **GP2** using **1c** (103.9 mg, 0.625 mmol) and **2a** (53.6 mg, 0.5 mmol) with 6 h reaction time. The residue was purified by flash chromatography (EtOAc) to give **4c** as a white solid (120 mg, 0.47 mmol, 94 %). Spectral data corresponds to previously published data.[7]

mp 134-135 °C; R_f (EtOAc-Hept 60:40) = 0.28; IR (neat, ATR) ν_{max} 421, 250, 510, 596, 607, 792, 1009, 1066, 1080, 1159, 1234, 1366, 1453, 1584, 1610, 1633, 2837, 2928, 3031, 3063, 3283; ^{1}H NMR (400 MHz, DMSO-d_6): δ 8.46 (br t, *J* = 5.5 Hz, 1H), 7.26-7.33 (m, 2H), 7.17-7.24 (m, 5H), 6.84-6.88 (m, 2H), 4.25 (d, *J* = 5.9 Hz, 2H), 3.72 (s, 3H), 3.39 (s, 2H); ^{13}C{^{1}H} NMR (101 MHz, DMSO-d_6): δ 170.3, 157.8, 139.4, 129.9, 128.2, 128.2, 127.1, 126.6, 113.5, 54.9, 42.1, 41.4.

4.2.4 *N*-Benzylheptanamide **4d**, MC020-326

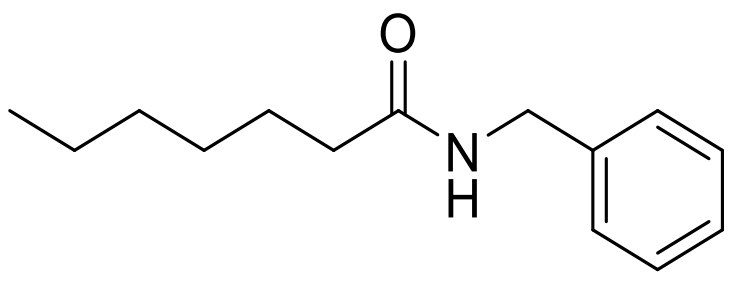

Prepared following **GP2** using **1d** (81.4 mg, 0.625 mmol) and **2a** (53.6 mg, 0.5 mmol) with 11 h reaction time. The residue was purified by flash chromatography (gradient heptane-EtOAc 100:0 to 68:32) to give **4d** as a white solid (102 mg, 0.50 mmol, 100 %). Spectral data corresponds to previously published data.[8]

mp 55-56 °C; R_f (EtOAc-Hept 60:40) = 0.57; IR (neat, ATR) ν_{max} 445, 458, 693, 7220, 1122, 1213, 1236, 1453, 1491, 1585, 1636, 2870, 2925, 2954, 3032, 3064, 3298; ^{1}H NMR (400 MHz, DMSO-d_6): δ 8.24-8.31 (m, 1H), 8.22-8.33 (m, 1H), 7.28-7.33 (m, 2H), 7.20-7.25 (m, 3H), 4.25 (d, *J* = 5.9 Hz, 2H), 2.12 (t, *J* = 7.4 Hz, 2H), 1.46-1.58 (m, 2H), 1.25 (br s, 6H), 0.84-0.87 (m, 3H); ^{13}C{^{1}H} NMR (101 MHz, DMSO-d_6): δ 172.2, 139.8, 128.3, 127.2, 126.7, 42.0, 35.4, 31.0, 28.4, 25.3, 22.0, 13.9.

4.2.5 *N*-Benzylcyclohexanecarboxamide **4e**, MC020-336

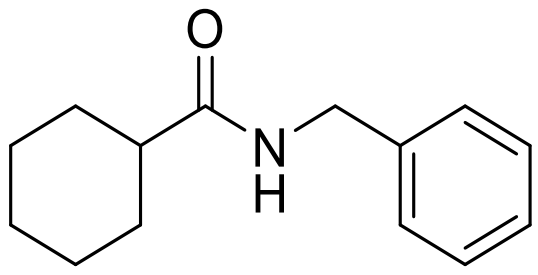

Prepared following **GP2** using **1e** (80.1 mg, 0.625 mmol) and **2a** (53.6 mg, 0.5 mmol) with 9 h reaction time. The residue was purified by flash chromatography (gradient heptane-EtOAc 100:0 to 67:33) to give **4e** as a white solid (82 mg, 0.38 mmol, 75 %). Spectral data corresponds to previously published data.[9]

mp 105-107 °C; R_f (EtOAc-Hept 40:60) = 0.3; IR (neat, ATR) ν_{max} 457, 525, 692, 743, 987, 1027, 1079, 1216, 1350, 1448, 1493, 1544, 1639, 2849, 2923, 3065, 3281; ^{1}H NMR (400 MHz, $CDCl_3$): δ 7.30-7.35 (m, 2H), 7.25-7.29 (m, 3H), 5.71 (br s, 1H), 4.44 (d, *J* = 5.7 Hz, 2H), 2.11 (tt, *J* = 11.7, 3.5 Hz, 1H), 1.89 (br d, *J* = 13.5 Hz, 2H), 1.79 (br dd, *J* = 9.8, 2.9 Hz, 2H), 1.64-1.70 (m, 1H), 1.62 (dd, *J* = 7.7, 3.2 Hz, 1H), 1.41-1.53 (m, 2H), 1.21-1.30 (m, 3H); ^{13}C{^{1}H} NMR (101 MHz, $CDCl_3$): δ 176.0, 138.7, 128.9, 127.9, 127.6, 45.7, 43.5, 29.9, 25.9.

4.2.6 *N*-Benzylcyclopentanecarboxamide **4f**, MC020-337

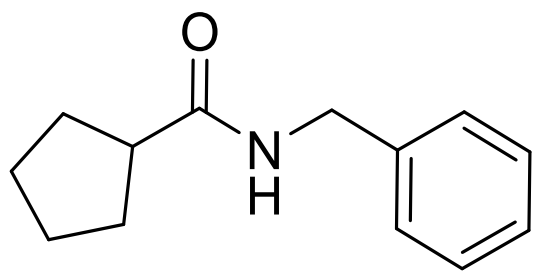

Prepared following **GP2** using **1f** (71.3 mg, 0.625 mmol) and **2a** (53.6 mg, 0.5 mmol) with 7 h reaction time. The residue was purified by flash chromatography (gradient heptane-EtOAc 100:0 to 65:35) to give **4f** as a white solid (102 mg, 0.50 mmol, 100 %). Spectral data corresponds to previously published data.[10]

mp 97-98 °C; R_f (EtOAc-Hept 60:40) = 0.62; IR (neat, ATR) ν_{max} 497, 578, 693, 747, 1027, 1230, 1473, 1498, 1537, 1632, 2863, 2950, 3032, 3288; ^{1}H NMR (400 MHz, $CDCl_3$): δ 7.30-7.36 (m, 2H), 7.25-7.29 (m, 3H), 5.76 (br s, 1H), 4.44 (d, *J* = 5.7 Hz, 2H), 2.50-2.58 (m, 1H), 1.71-1.91 (m, 6H), 1.52-1.68 (m, 2H); ^{13}C{^{1}H} NMR (101 MHz, $CDCl_3$): δ 176.2, 138.7, 128.8, 127.9, 127.6, 46.0, 43.7, 30.6, 26.0.

4.2.7 1-Morpholino-2-phenylethan-1-one **4g**, MC020-325

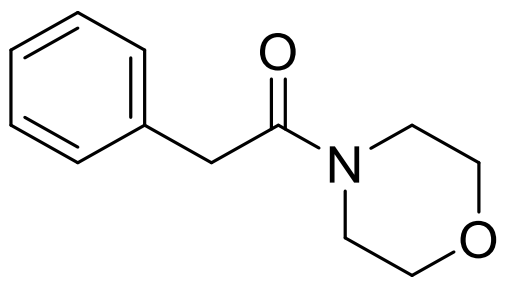

Prepared following **GP2** using **1a** (85.1 mg, 0.625 mmol) and **2b** (43.6 mg, 0.5 mmol) with 14 h reaction time. The residue was purified by flash chromatography (gradient heptane-EtOAc 100:0 to 40:60) to give **4g** as a white solid (87 mg, 0.43 mmol, 85 %). Spectral data corresponds to previously published data.[11]

mp 60-61 °C; R_f (EtOAc-Hept 60:40) = 0.22; IR (neat, ATR) ν_{max} 420, 526, 724, 771, 1069, 1113, 1196, 1253, 1299, 1360, 1453, 1714, 1943, 2242, 2855, 2965, 3481; ^{1}H NMR (400 MHz, $CDCl_3$): δ 7.37 – 7.31 (m, 2H), 7.29 – 7.23 (m, 3H), 3.75 (s, 2H), 3.66 (s, 4H), 3.52 – 3.42 (m, 4H); ^{13}C{^{1}H} NMR (101 MHz, $CDCl_3$): δ 169.8, 134.9, 128.9, 128.6, 127.0, 66.9, 66.6, 46.6, 42.3, 41.0.

4.2.8 2-Phenyl-1-(pyrrolidin-1-yl)ethan-1-one **4h**, MC020-356

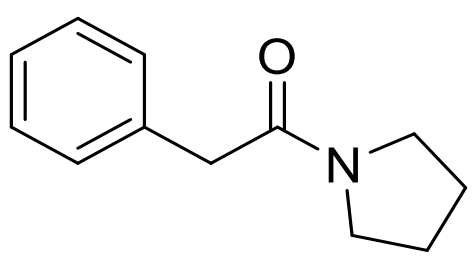

Prepared following **GP2** using **1a** (85.1 mg, 0.625 mmol) and **2c** (35.6 mg, 0.5 mmol) with 9 h reaction time. The residue was purified by flash chromatography (EtOAc 100 %) to give **4h** as an oil (93 mg, 0.49 mmol, 98 %). Spectral data corresponds to previously published data.[11]

R_f (EtOAc-Hept 50:50) = 0.3; IR (neat, ATR) ν_{max} 461, 522, 564, 719, 741, 1115, 1422, 1494, 1630, 2872, 2970, 3028, 3060, 3465; ^{1}H NMR (400 MHz, $CDCl_3$): δ 7.35 – 7.22 (m, 5H), 3.66 (s, 2H), 3.46 (dt, J = 28.1, 6.8 Hz, 4H), 1.95 – 1.79 (m, 4H);^{13}C{^{1}H} NMR (101 MHz, $CDCl_3$): δ 169.5, 135.0, 129.0, 128.6, 126.7, 46.9, 45.9, 42.3, 26.2, 24.4.

4.2.9 2-Phenyl-1-(4-phenylpiperazin-1-yl)ethan-1-one **4i**, MC020-345/362

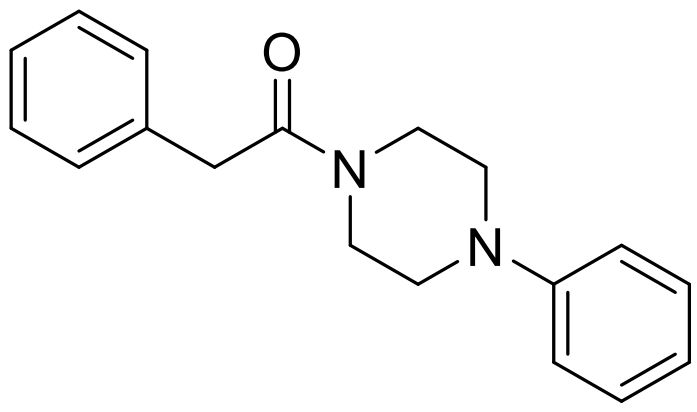

Prepared following **GP2** using **1a** (1.25 g, 9.18 mmol) and **2d** (1 g, 6.16 mmol) with 7 h reaction time using Dean-Stark. The residue was purified by flash chromatography

(gradient heptane-EtOAc 100:0 to 52:48) to give **4i** as an oil (1.73 g, 6.16 mmol, 100 %). Spectral data corresponds to previously published data.[11]

R_f (EtOAc-Hept 60:40) = 0.75; IR (neat, ATR) ν_{max} 416, 453, 535, 595, 684, 719, 869, 990, 1239, 1327, 1428, 1577, 1633, 2755, 2917, 3059; ^{1}H NMR (400 MHz, $CDCl_3$): δ 7.24-7.30 (m, 5H), 3.77-3.88 (m, 4H), 3.56-3.63 (m, 2H), 3.09-3.18 (m, 2H), 2.94-3.01 (m, 2H); ^{13}C{^{1}H} NMR (101 MHz, $CDCl_3$): δ 169.3, 150.7, 134.8, 129.1, 128.6, 128.4, 126.7, 120.3, 116.4, 49.3, 49.0, 45.8, 41.5, 40.9.

4.2.10 *tert*-Butyl (2-(2-phenylacetamido)ethyl)carbamate **4j**, MC020-334

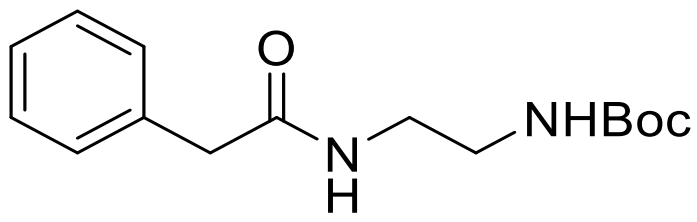

Prepared following **GP2** using **1a** (85.1 mg, 0.625 mmol) and **2e** (80.1 mg, 0.5 mmol) with 14 h reaction time. The residue was purified by flash chromatography (gradient heptane-EtOAc 100:0 to 40:60) to give **4j** as a white solid (105 mg, 0.38 mmol, 75 %). Spectral data corresponds to previously published data.[12]

mp 130-133 °C; R_f (EtOAc-Hept 60:40) = 0.22; IR (neat, ATR) ν_{max} 424, 534, 656, 866, 1031, 1163, 1275, 1315, 1364, 1532, 1682, 1731, 2933, 2983, 3312, 3352; ^{1}H NMR (600 MHz, DMSO-d_6): δ 8.04 (br t, *J* = 5.0 Hz, 1H), 7.26-7.30 (m, 2H), 7.19-7.25 (m, 3H), 6.79 (br t, *J* = 5.5 Hz, 1H), 3.38 (s, 2H), 3.06 (q, *J* = 6.3 Hz, 2H), 2.97 (q, *J* = 6.3 Hz, 2H), 1.37 (s, 9H); ^{13}C{^{1}H} NMR (151 MHz, DMSO-d_6): δ 170.2, 155.6, 136.4, 129.0, 128.2, 128.1, 126.3, 77.7, 42.4, 40.1, 38.8, 28.2.

4.2.11 *N*-Cyclohexyl-2-phenylacetamide **4k**, MC020-354

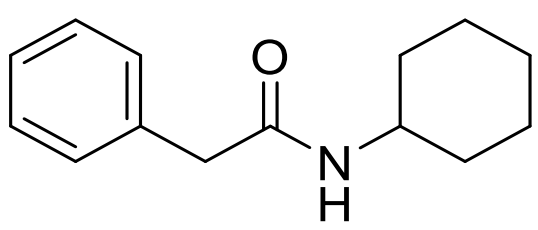

Prepared following **GP2** using **1a** (85.1 mg, 0.625 mmol) and **2f** (49.6 mg, 0.5 mmol) with 14 h reaction time. The residue was purified by flash chromatography (gradient heptane-EtOAc 100:0 to 63:37) to give **4k** as a white solid (85 mg, 0.39 mmol, 78 %). Spectral data corresponds to previously published data.[6]

mp 134-135 °C; R_f (EtOAc-Hept 50:50) = 0.5; IR (neat, ATR) ν_{max} 442, 531, 693, 713, 1029, 1169, 1255, 1370, 1602, 1631, 1665, 2852, 2929, 3064, 3265; ^{1}H NMR (400 MHz, $CDCl_3$): δ 7.22-7.37 (m, 5H), 5.22 (br s, 1H), 3.69-3.83 (m, 1H), 3.55 (s, 2H), 1.79-1.87 (m, 2H), 1.53-1.65 (m, 3H), 1.25-1.38 (m, 2H), 0.95-1.16 (m, 3H); ^{13}C{^{1}H} NMR (101 MHz, $CDCl_3$): δ = 170.2, 135.3, 129.5, 129.1, 127.4, 48.3, 44.2, 33.0, 25.6, 24.8.

4.2.12 *N*-Hexyl-2-phenylacetamide **4l**, MC020-331

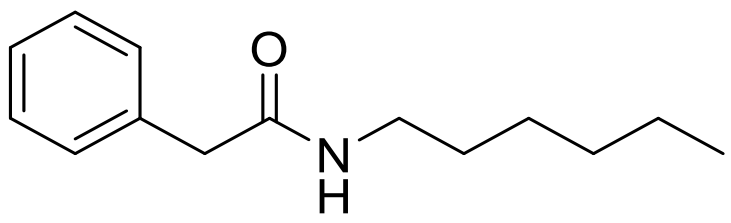

Prepared following **GP2** using **1a** (85.1 mg, 0.625 mmol) and **2g** (50.6 mg, 0.5 mmol) with 14 h reaction time. The residue was purified by flash chromatography (gradient heptane-EtOAc 100:0 to 60:40) to give **4l** as a white solid (95 mg, 0.44 mmol, 87 %). Spectral data corresponds to previously published data.[6]

mp 53-55 °C; R_f (EtOAc-Hept 60:40) = 0.62; IR (neat, ATR) ν_{max} 404, 542, 1026, 1265, 1352, 1369, 1428, 1599, 1626, 2860, 2935, 2958, 3065, 3250; ^{1}H NMR (400 MHz, $CDCl_3$): δ 7.40 – 7.24 (m, 5H), 5.39 (s, 1H), 3.58 (s, 2H), 3.21 (td, *J* = 7.2, 5.8 Hz, 2H), 1.42 (dd, *J* = 8.6, 5.5 Hz, 2H), 1.24 (tq, *J* = 9.0, 4.1 Hz, 6H), 0.90 – 0.84 (m, 3H); ^{13}C{^{1}H} NMR (101 MHz, $CDCl_3$): δ 171.0, 135.2, 129.6, 129.1, 127.5, 44.0, 39.8, 31.5, 29.5, 26.5, 22.6, 14.1.

4.2.13 (*S*)-*N*-Benzyl-2-(6-methoxynaphthalen-2-yl)propenamide **4m**, MC020-350

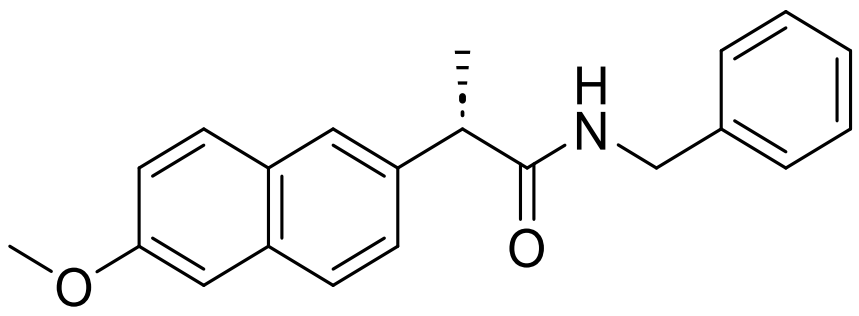

Prepared following **GP2** using **1g** (143.9 mg, 0.625 mmol) and **2a** (53.6 mg, 0.5 mmol) with 14 h reaction time. The residue was purified by flash chromatography (gradient heptane-EtOAc 100:0 to 65:35) to give **4m** as a white solid (150 mg, 0.47 mmol, 94 %). Enantiomeric ratio was determined with chiral SFC (EtOH/*i*-PrOH 1:1, 3.5 mL/min, Phenomenex LuxAMY1 5µm 4,6 x150 mm) t_R (*S*) = 4.47 min (82 %), t_R (*R*) = 5.18 min (18 %). Spectral data corresponds to previously published data.[13]

mp 122-127 °C; R_f (EtOAc-Hept 60:40) = 0.5; IR (neat, ATR) ν_{max} 474, 532, 649, 743, 811, 979, 1024, 1069, 1158, 1212, 1265, 1373, 1414, 1505, 1535, 1638, 2875, 2973, 3031, 3295; ^{1}H NMR (400 MHz, $CDCl_3$) δ 7.76 – 7.67 (m, 3H), 7.41 (dd, *J* = 8.4, 1.9 Hz, 1H), 7.29 – 7.23 (m, 3H), 7.19 – 7.12 (m, 4H), 5.72 (s, 1H), 4.48 – 4.31 (m, 2H), 3.94 (s, 3H), 3.76 (q, *J* = 7.1 Hz, 1H), 1.65 (d, *J* = 7.2 Hz, 3H); ^{13}C{^{1}H} NMR (101 MHz, $CDCl_3$): δ 174.3, 157.9, 138.4, 136.5, 133.9, 129.3, 129.1, 128.7, 127.7, 127.6, 127.5, 126.4, 126.3, 119.3, 105.8, 55.5, 47.2, 43.7, 18.7.

4.2.14 N-(pyridin-2-yl)-2-(4-(trifluoromethyl)phenyl)acetamide **4n**, TJB-2-008

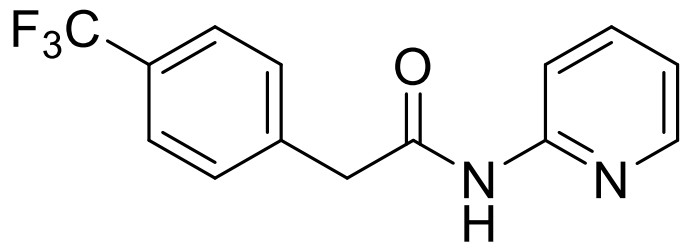

Prepared following **GP2** using **1h** (127.6 mg, 0.625 mmol) and **2h** (47.1 mg, 0.5 mmol) with 11 h reaction time. The residue was purified by flash chromatography (gradient heptane-EtOAc 100:0 to 80:20) to give **4n** as a white solid (65 mg, 0.23 mmol, 46 %). Spectral data corresponds to previously published data.[14]

mp 139–143 °C; R_f (EtOAc-Hex 50:50) = 0.5; IR (neat, ATR) ν_{max} 516, 653, 822, 851, 994, 1020, 1213, 1327, 1438, 1529, 1618, 1694, 3040, 3116, 3213, 3248; ^{1}H NMR (500 MHz, DMSO) δ 10.80 (s, 1H), 8.32 (ddd, *J* = 4.9, 2.0, 0.9 Hz, 1H), 8.04 (d, *J* = 8.4 Hz, 1H), 7.76 (ddd, *J* = 8.8, 7.4, 2.0 Hz, 1H), 7.70 (d, *J* = 8.0 Hz, 2H), 7.57 (d, *J* = 8.0 Hz, 2H), 3.85 (s, 2H); ^{13}C{^{1}H} NMR (126 MHz, DMSO) δ 169.3, 151.9, 148.0, 140.6, 138.2, 130.1, 127.4 (q, $^2J_{C\text{-}F}$ = 31.6 Hz), 125.2 (q, $^3J_{C\text{-}F}$ = 4.1 Hz), 124.4 (q, $^1J_{C\text{-}F}$ = 272.7 Hz), 119.5, 113.4, 42.6.

4.2.15 *N*-Benzylbenzamide **4o**, MC020-294

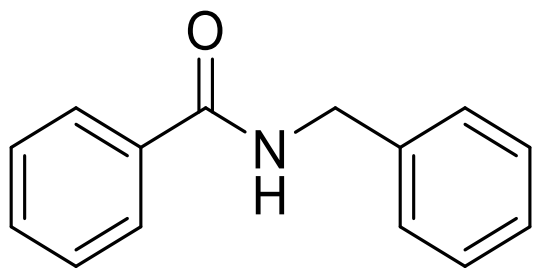

Prepared following **GP2** using **1i** (76.3 mg, 0.625 mmol) and **2a** (53.6 mg, 0.5 mmol) with 9 h reaction time. The residue was purified by flash chromatography (gradient heptane-EtOAc 100:0 to 67:33) to give **4o** as a white solid (104 mg, 0.50 mmol, 99 %) Spectral data corresponds to previously published data.[15]

mp 101-104 °C; R_f (EtOAc-Hept 40:60) = 0.4; IR (neat, ATR) ν_{max} 412, 519, 691, 724, 904, 985, 1054, 1260, 1299, 1361, 1546, 1600, 1635, 2926, 3029, 3061, 3280; ^{1}H NMR (400 MHz, DMSO-d_6,): δ 9.04 (br t, *J* = 5.9 Hz, 1H), 7.89 (d, *J* = 7.6 Hz, 2H), 7.45-7.56 (m, 3H), 7.30-7.34 (m, 4H), 7.21-7.27 (m, 1H), 4.48 (d, *J* = 6.1 Hz, 2H); ^{13}C{^{1}H} NMR (101 MHz, DMSO-d_6): δ 166.2, 139.7, 134.3, 131.3, 128.3, 128.3, 127.2, 127.2, 126.7, 42.6.

4.2.16 *N*-Benzyl-4-chlorobenzamide **4p**, MC020-295

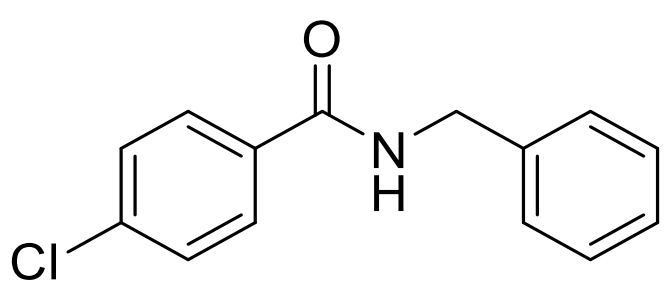

Prepared following **GP2** using **1j** (97.9 mg, 0.625 mmol) and **2a** (53.6 mg, 0.5 mmol) with 9 h reaction time. The residue was purified by flash chromatography (gradient heptane-

EtOAc 100:0 to 67:33) to give **4p** as a white solid (118 mg, 0.48 mmol, 96 %). Spectral data corresponds to previously published data.[16]

mp 157-161 °C; R_f (EtOAc-Hept 40:60) = 0.47; IR (neat, ATR) ν_{max} 470, 547, 694, 735, 794, 902, 1011, 1128, 117 7, 1360, 1484, 1591, 1637, 3029, 3061, 3308; ^{1}H NMR (400 MHz, DMSO-d$_6$): δ 9.12 (br t, J = 5.9 Hz, 1H), 7.88-7.95 (m, 2H), 7.52-7.58 (m, 2H), 7.29-7.34 (m, 4H), 7.20-7.27 (m, 1H), 4.47 (d, J = 6.0 Hz, 2H); ^{13}C{^{1}H} NMR (101 MHz, DMSO-d$_6$): δ 165.2, 139.5, 136.1, 133.1, 131.2, 129.2, 128.8, 128.4, 128.3, 127.2, 126.8, 42.7.

4.2.17 *N*-Benzyl-4-(trifluoromethyl)benzamide **4q**, MC020-357

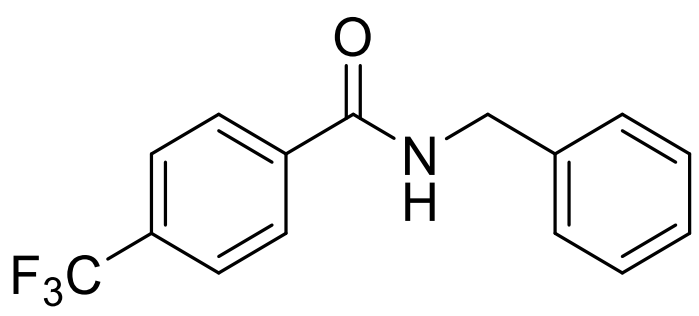

Prepared following **GP2** using **1k** (118.8 mg, 0.625 mmol) and **2a** (53.6 mg, 0.5 mmol) with 8 h reaction time. The residue was purified by flash chromatography (gradient heptane-EtOAc 100:0 to 77:23) to give **4q** as a white solid (140 mg, 0.50 mmol, 100 %). Spectral data corresponds to previously published data.[17]

Mp 169-171 °C; R_f (EtOAc-Hept 50:50) = 0.6; IR (neat, ATR) ν_{max} 405, 451, 599, 633, 795, 903, 1050, 1121, 1255, 1308, 1407, 1453, 1641, 2933, 3034, 3066, 3324; ^{1}H NMR (400 MHz, CDCl$_3$): δ 7.89 (d, J = 8.1 Hz, 2H), 7.67 (d, J = 8.2 Hz, 2H), 7.28-7.38 (m, 5H), 6.59 (br s, 1H), 4.64 (d, J = 5.6 Hz, 2H); ^{13}C{^{1}H} NMR (101 MHz, CDCl$_3$): δ166.3, 137.8, 137.7, 133.4 (q, $^2J_{C-F}$ = 32.8 Hz), 129.0, 128.1, 128.0, 127.6, 125.7 (q, $^3J_{C-F}$ = 3.8 Hz), 123.8 (q, $^1J_{C-F}$ = 272.5 Hz), 44.5.

4.2.18 *N*-Benzyl-4-methoxybenzamide **4r**, MC020-296

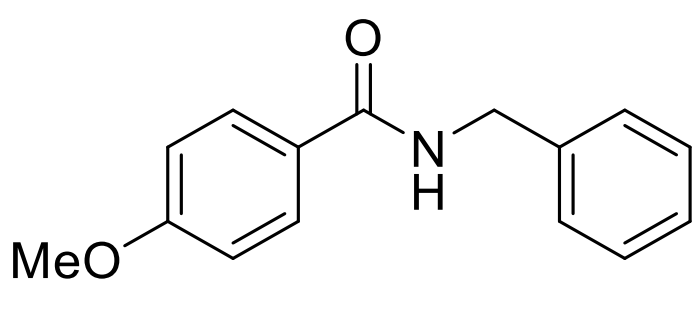

Prepared following **GP2** using **1l** (95.1 mg, 0.625 mmol) and **2a** (53.6 mg, 0.5 mmol) with 14 h reaction time. The residue was purified by flash chromatography (gradient heptane-EtOAc 100:0 to 68:32) to give **4r** as a white solid (91 mg, 0.38 mmol, 75 %). Spectral data corresponds to previously published data.[17]

mp 124-127 °C; R_f (EtOAc-Hept 40:60) = 0.24; IR (neat, ATR) ν_{max} 456, 530, 632, 841, 985, 1027, 1119, 1248, 1268, 1407, 1449, 1460, 15557, 1630, 2837, 2956, 3023, 3252; ^{1}H NMR (400 MHz, DMSO-d$_6$): δ 8.89 (s, 1H), 7.85-7.90 (m, 2H), 7.29-7.33 (m, 4H), 7.20-7.27 (m,

1H), 6.98-7.02 (m, 2H), 4.46 (d, *J* = 6.0 Hz, 2H), 3.81 (s, 3H); $^{13}C\{^1H\}$ NMR (101 MHz, DMSO-d_6): δ 165.7, 161.6, 139.9, 129.1, 128.3, 127.2, 126.7, 126.6, 113.5, 55.4, 42.5.

4.2.19 *N*-Benzyl-2-methoxybenzamide **4s**, MC020-358

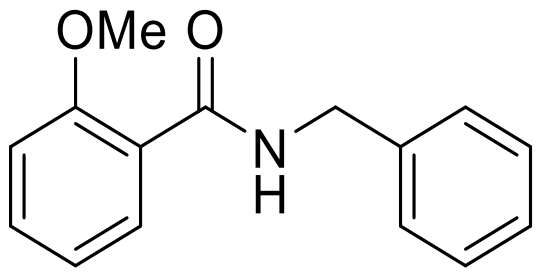

Prepared following **GP2** using **1m** (95.1 mg, 0.625 mmol) and **2a** (53.6 mg, 0.5 mmol) with 11 h reaction time. The residue was purified by flash chromatography (gradient heptane-EtOAc 100:0 to 65:35) to give **4s** as an oil (90 mg, 0.38 mmol, 75 %). Spectral data corresponds to previously published data.[18]

R_f (EtOAc-Hept 40:60) = 0.25; IR (neat, ATR) ν_{max} 484, 603, 697, 754, 1019, 1079, 1236, 1435, 1482, 1495, 1524, 1598, 1644, 2838, 2941, 3063, 3395; ^1H NMR (400 MHz, DMSO-d_6): δ 8.70 (br t, *J* = 5.8 Hz, 1H), 7.76 (dd, *J* = 7.7, 1.8 Hz, 1H), 7.47 (ddd, *J* = 8.4, 7.3, 1.9 Hz, 1H), 7.32-7.35 (m, 4H), 7.21-7.27 (m, 1H), 7.14 (d, *J* = 8.3 Hz, 1H), 7.04 (td, *J* = 7.5, 0.9 Hz, 1H), 4.51 (d, *J* = 6.2 Hz, 2H), 3.88 (s, 3H); $^{13}C\{^1H\}$ NMR (101 MHz, DMSO-d_6): δ 165.2, 157.0, 139.7, 132.2, 130.4, 128.3, 127.0, 126.7, 123.2, 120.5, 112.0, 55.9, 42.6.

4.2.20 *N*-Benzyl-2-naphthamide **4t**, MC020-307

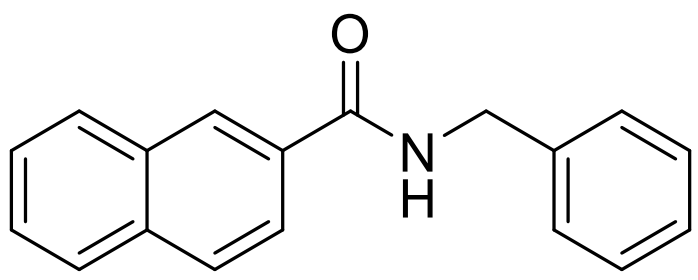

Prepared following **GP2** using **1n** (107.6 mg, 0.625 mmol) and **2a** (53.6 mg, 0.5 mmol) with 14 h reaction time. The residue was purified by flash chromatography (gradient heptane-EtOAc 100:0 to 70:30) to give **4t** as white solid (105 mg, 0.40 mmol, 80 %). Spectral data corresponds to previously published data.[19]

mp 132-136 °C; R_f (EtOAc-Hept 40:60) = 0.48; IR (neat, ATR) ν_{max} 415, 527, 578, 620, 693, 760, 829, 997, 1078, 1208, 1357, 1415, 1476, 1547, 1638, 2922, 3023, 3291; ^1H NMR (400 MHz, DMSO-d_6): δ 9.21 (br t, *J* = 5.8 Hz, 1H), 9.18-9.24 (m, 1H), 8.50 (s, 1H), 7.95-8.05 (m, 1H), 7.90-8.08 (m, 3H), 7.57-7.66 (m, 2H), 7.30-7.42 (m, 3H), 7.30-7.40 (m, 1H), 7.21-7.28 (m, 1H), 7.20-7.28 (m, 1H), 4.54 (d, *J* = 6.0 Hz, 2H); $^{13}C\{^1H\}$ NMR (101 MHz, DMSO-d_6): δ166.3, 139.7, 134.2, 132.2, 131.7, 128.9, 128.3, 127.9, 127.7, 127.5, 127.3, 126.8, 126.8, 124.2, 42.8.

4.2.21 *N*-Benzyl-3,4-dihydro-2H-benzo[b][1,4]oxazine-6-carboxamide **4u**, MC020-298

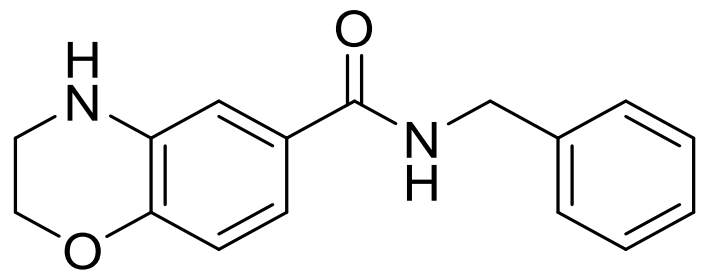

Prepared following **GP2** using **1o** (112.0 mg, 0.625 mmol) and **2a** (53.6 mg, 0.5 mmol) with 13 h reaction time. The residue was purified by flash chromatography (gradient heptane-EtOAc 100:0 to 65:35) to give **4u** as off white solid (98 mg, 0.37 mmol, 73 %).

mp 115-120 °C; R_f (EtOAc-Hept 60:40) = 0.2; IR (neat, ATR) ν_{max} 433, 510, 580, 721, 460, 818, 881, 971, 1034, 1145, 1267, 1362, 1450, 1504, 1606, 2876, 2985, 3061, 3316, 3385; ^{1}H NMR (400 MHz, DMSO-d$_6$): δ 8.71 (t, *J* = 6.1 Hz, 1H), 7.34 – 7.26 (m, 4H), 7.24 – 7.20 (m, 1H), 7.13 (d, *J* = 2.1 Hz, 1H), 7.05 (dd, *J* = 8.3, 2.1 Hz, 1H), 6.67 (d, *J* = 8.2 Hz, 1H), 5.90 (s, 1H), 4.42 (d, *J* = 6.0 Hz, 2H), 4.15 (t, *J* = 4.4 Hz, 2H), 3.28 (td, *J* = 4.3, 2.2 Hz, 2H); ^{13}C{^{1}H} NMR (101 MHz, DMSO-d$_6$): δ 166.3, 145.7, 140.1, 134.6, 128.2, 127.3, 127.1, 126.6, 116.0, 115.4, 114.3, 65.0, 42.5; HRMS (ESI+): m/z calcd for $[C_{16}H_{16}N_2O_2+H]^+$ = 269.1290, found 269.1293, Δ = 1.1 ppm.

4.2.22 *N*-Benzylpicolinamide **4v**, MC020-280

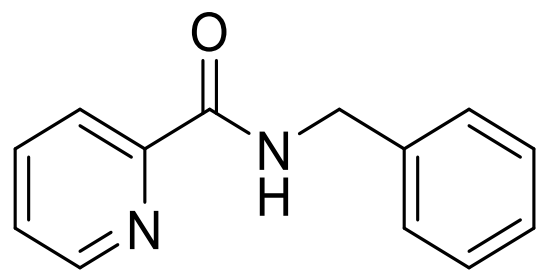

Prepared following **GP2** using **1p** (76.9 mg, 0.625 mmol) and **2a** (53.6 mg, 0.5 mmol) with 14 h reaction time. The residue was purified by flash chromatography (gradient heptane-EtOAc 100:0 to 64:36) to give **4v** as off white solid (106 mg, 0.50 mmol, 100 %). Spectral data corresponds to previously published data.[20]

mp 86-89 °C; R_f (EtOAc-Hept 40:60) = 0.34; IR (neat, ATR) ν_{max} 439, 515, 686, 741, 970, 1166, 1250, 1329, 1432, 1569, 1656, 2927, 3028, 3083, 3300; ^{1}H NMR (400 MHz, DMSO-d$_6$): δ 9.32 (br t, *J* = 6.1 Hz, 1H), 8.65 (d, *J* = 4.8 Hz, 1H), 7.97-8.07 (m, 2H), 7.61 (ddd, *J* = 7.4, 4.8, 1.4 Hz, 1H), 7.28-7.35 (m, 4H), 7.20-7.27 (m, 1H), 4.50 (d, *J* = 6.5 Hz, 2H); ^{13}C{^{1}H} NMR (101 MHz, DMSO-d$_6$): δ 163.9, 150.0, 148.5, 139.6, 137.8, 128.3, 127.4, 126.8, 126.6, 122.0, 42.4.

4.2.23 *N*-Benzyl-1*H*-indole-2-carboxamide **4w**, MC020-297

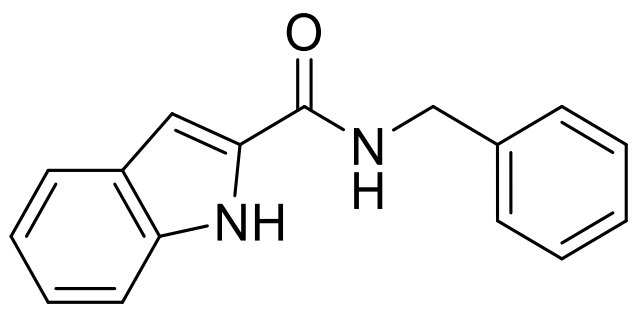

Prepared following **GP2** using **1q** (100.7 mg, 0.625 mmol) and **2a** (53.6 mg, 0.5 mmol) with 14 h reaction time. The residue was purified by flash chromatography (gradient DCM-MeOH 100:0 to 95:5) to give **4w** as red solid (84 mg, 0.34 mmol, 67 %). Spectral data corresponds to previously published data.[4]

mp 224-229 °C; R_f (EtOAc-Hept 40:60) = 0.47; IR (neat, ATR) ν_{max} 409, 472, 571, 610, 737, 1030, 1160, 1218, 1314, 1417, 1493, 1541, 1632, 2926, 3030, 3261, 3417; ^{1}H NMR (400 MHz, DMSO-d_6): δ 11.58 (s, 1H), 9.02 (t, *J* = 6.1 Hz, 1H), 7.61 (d, *J* = 8.0 Hz, 1H), 7.42 (d, *J* = 8.4 Hz, 1H), 7.34 (d, *J* = 4.4 Hz, 4H), 7.25 (dq, *J* = 8.5, 4.3 Hz, 1H), 7.14-7.20 (m, 2H), 7.03 (t, *J* = 7.5 Hz, 1H), 4.51 (d, *J* = 6.1 Hz, 2H); ^{13}C{^{1}H} NMR (101 MHz, DMSO-d_6): δ 161.2, 139.6, 136.5, 131.6, 128.4, 127.2, 127.1, 126.8, 123.3, 121.5, 119.8, 112.3, 102.6, 42.2.

4.2.24 *N*-Benzylthiophene-2-carboxamide **4x**, MC020-304

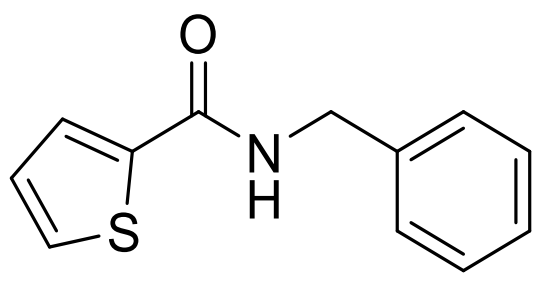

Prepared following **GP2** using **1r** (80.1 mg, 0.625 mmol) and **2a** (53.6 mg, 0.5 mmol) with 14 h reaction time. The residue was purified by flash chromatography (gradient heptane-EtOAc 100:0 to 70:30) to give **4x** as off white solid (103 mg, 0.48 mmol, 95 %). Spectral data corresponds to previously published data.[17]

mp 112-115 °C; R_f (EtOAc-Hept 40:60) = 0.4; IR (neat, ATR) ν_{max} 438, 494, 638, 729, 783, 1022, 1201, 1371, 1492, 1540, 1618, 3030, 3089, 3349; ^{1}H NMR (400 MHz, DMSO-d): δ 9.05 (br t, *J* = 5.8 Hz, 1H), 7.78 (m, 2H), 7.29-7.35 (m, 4H), 7.22-7.28 (m, 1H), 7.15 (dd, *J* = 5.0, 3.8 Hz, 1H), 4.45 (d, *J* = 6.1 Hz, 2H); ^{13}C{^{1}H} NMR (101 MHz, DMSO-d): δ 161.2, 139.9, 139.5, 130.9, 128.4, 128.1, 128.0, 127.3, 126.9, 42.5.

4.2.25 *tert*-Butyl 4-benzamidopiperidine-1-carboxylate **4y**, MC020-303

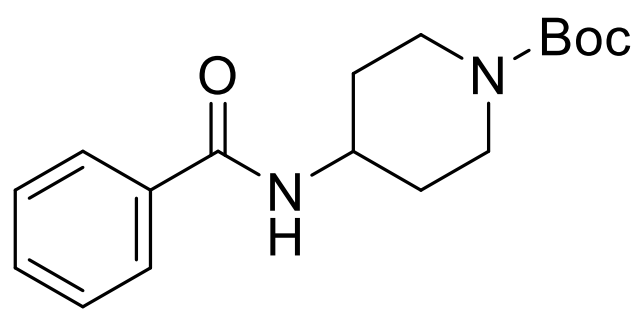

Prepared following **GP2** using **1i** (76.3 mg, 0.625 mmol) and **2i** (100.1 mg, 0.5 mmol) with 14 h reaction time. The residue was purified by flash chromatography (gradient heptane-EtOAc 100:0 to 48:52) to give **4y** as white solid (93 mg, 0.31 mmol, 61 %). Spectral data corresponds to previously published data.[21]

mp 171-172 °C; R_f (EtOAc-Hept 60:40) = 0.43; IR (neat, ATR) ν_{max} 429, 694, 946, 1006, 1114, 1175, 1275, 1294, 1307, 1363, 1433, 1490, 1577, 1646, 1699, 2854, 2929, 2979, 3274; ^{1}H NMR (400 MHz, $CDCl_3$): δ 7.78 – 7.70 (m, 2H), 7.45 (tdd, *J* = 6.5, 3.2, 1.6 Hz, 1H), 7.42 – 7.32 (m, 2H), 6.48 – 6.29 (m, 1H), 4.08 (td, *J* = 10.9, 5.2 Hz, 3H), 2.84 (s, 2H), 2.02 – 1.90 (m, 2H), 1.50 – 1.24 (m, 12H); ^{13}C{^{1}H} NMR (101 MHz, $CDCl_3$): δ 167.0, 154.8, 134.6, 131.5, 131.5, 128.6, 128.5, 127.0, 127.0, 79.7, 47.3, 42.8, 32.1, 28.5.

4.2.26 *N*-Cyclohexylbenzamide **4z**, MC020-315

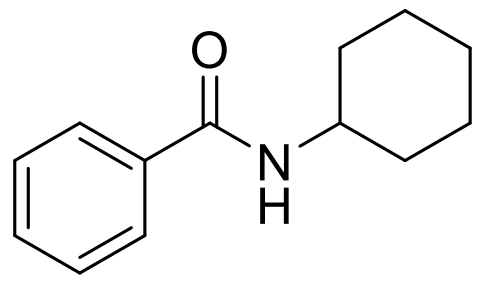

Prepared following **GP2** using **1i** (76.3 mg, 0.625 mmol) and **2f** (49.6 mg, 0.5 mmol) with 21 h reaction time. The residue was purified by flash chromatography (gradient heptane-EtOAc 100:0 to 70:30) to give **4z** as a white solid (47 mg, 0.23 mmol, 46 %). Spectral data corresponds to previously published data.[22]

mp 144-147 °C; R_f (EtOAc-Hept 40:60) = 0.38; IR (neat, ATR) ν_{max} 409, 552, 691, 720, 845, 969, 1128, 1241, 1326, 1445, 1529, 1576, 1626, 1682, 2850, 2928, 3326; ^{1}H NMR (400 MHz, DMSO-d_6): δ 8.17 (d, *J* = 8.0 Hz, 1H), 7.88 – 7.79 (m, 2H), 7.54 – 7.38 (m, 3H), 3.75 (d, *J* = 8.7 Hz, 1H), 1.86 – 1.66 (m, 4H), 1.61 (d, *J* = 13.0 Hz, 1H), 1.37 – 1.22 (m, 4H), 1.20 – 1.04 (m, 1H); ^{13}C{^{1}H} NMR (101 MHz, DMSO-d_6): δ 165.2, 134.8, 130.8, 128.0, 127.1, 48.2, 32.3, 25.2, 24.8.

4.2.27 *N*-Phenylbenzamide **4aa**, MC020-316

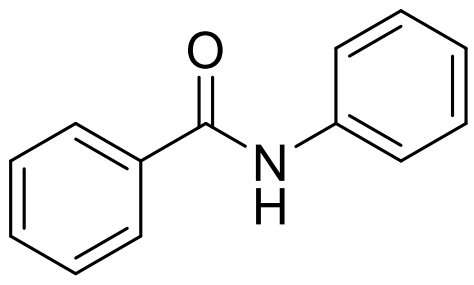

Prepared following **GP2** using **1i** (76.3 mg, 0.625 mmol) and **2j**(46.6 mg, 0.5 mmol) with 6 h reaction time. The residue was purified by flash chromatography (gradient heptane-EtOAc 100:0 to 70:30) to give **4aa** as a white solid (85 mg, 0.43 mmol, 86 %). Spectral data corresponds to previously published data.[17]

mp 155-159 °C; R_f (EtOAc-Hept 40:60) = 0.46; IR (neat, ATR) ν_{max} 489, 581, 644, 687, 747, 883, 926, 1025, 1112, 1127, 1177, 1295, 1434, 1489, 1524, 1597, 1651, 3025, 3051, 3341; ^{1}H NMR (400 MHz, DMSO- d_6): δ 10.24 (s, 1H), 7.93-7.96 (m, 2H), 7.77 (d, J = 7.7 Hz, 2H), 7.57-7.62 (m, 1H), 7.53 (t, J = 7.6 Hz, 2H), 7.35 (t, J = 7.9 Hz, 2H), 7.10 (t, J = 7.4 Hz, 1H); ^{13}C{^{1}H} NMR (101 MHz, DMSO- d_6): δ 165.6, 139.2, 135.0, 131.6, 128.6, 128.4, 127.7, 123.7, 120.4.

4.2.28 *N,N*-Dimethylbenzamide **4ab**, MC020-308

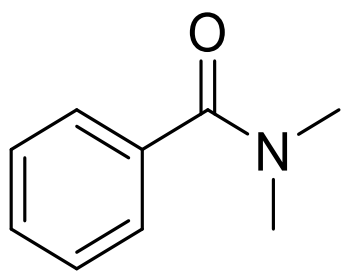

Prepared following **GP2** using **1i** (76.3 mg, 0.625 mmol) and **2k** (22.5 mg, 0.5 mmol) with 14 h reaction time. The residue was purified by flash chromatography (gradient heptane-EtOAc 100:0 to 70:30) to give **4ab** as an oil (35 mg, 0.24 mmol, 47 %). Spectral data corresponds to previously published data.[15]

R_f (EtOAc-Hept 60:40) = 0.25; IR (neat, ATR) ν_{max} 437, 637, 712, 789, 1025, 1083, 1178, 1263, 1444, 1481, 1576, 1623, 2928, 3026, 3057, 3476; ^{1}H NMR (400 MHz, DMSO-d_6): δ 7.41-7.45 (m, 3H), 7.37-7.41 (m, 2H), 2.98 (br s, 3H), 2.89 (br s, 3H); ^{13}C{^{1}H} NMR (101 MHz, DMSO-d_6): δ 170.2, 136.5, 129.3, 128.3, 126.9, 39.0, 34.7.

4.2.29 Morpholino(phenyl)methanone **4ac**, MC020-291

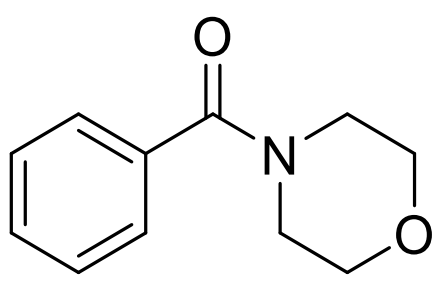

Prepared following **GP2** using **1i** (76.3 mg, 0.625 mmol) and **2b** (43.6 mg, 0.5 mmol) with 14 h reaction time. The residue was purified by flash chromatography (gradient heptane-

EtOAc 100:0 to 70:30) to give **4ac** as an oil (69 mg, 0.36 mmol, 71 %). Spectral data corresponds to previously published data.[15]

R_f (EtOAc-Hept 60:40) = 0.25; IR (neat, ATR) ν_{max} 431, 646, 734, 889, 932, 1031, 1108, 1213, 1398, 1392, 1423, 1492, 1622, 2858, 2912, 2978; ^{1}H NMR (400 MHz, $CDCl_3$): δ 7.37-7.45 (m, 5H), 3.57-3.87 (m, 6H), 3.34-3.57 (m, 2H); ^{13}C{^{1}H} NMR (101 MHz, $CDCl_3$): δ 170.5, 135.4, 130.0, 128.7, 127.2, 67.0, 48.3, 42.7.

4.2.30 Phenyl(pyrrolidin-1-yl)methanone **4ad**, MC020-288

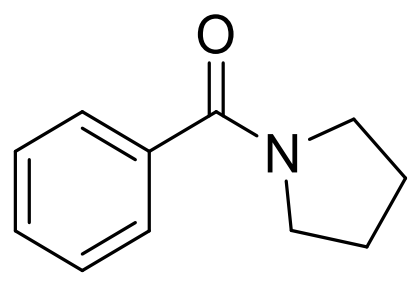

Prepared following **GP2** using **1i** (76.3 mg, 0.625 mmol) and **2c** (35.6 mg, 0.5 mmol) with 14 h reaction time. The residue was purified by flash chromatography (gradient heptane-EtOAc 100:0 to 70:30) to give **4ad** as an oil (64 mg, 0.37 mmol, 73 %). Spectral data corresponds to previously published data.[20]

R_f (EtOAc-Hept 60:40) = 0.2; IR (neat, ATR) ν_{max} 469, 594, 699, 745, 872, 1026, 1228, 1291, 1339, 1414, 1483, 1574, 614, 2874, 2969, 3057, 3470; ^{1}H NMR (400 MHz, $CDCl_3$): δ 7.55 – 7.47 (m, 2H), 7.39 (dd, *J* = 5.2, 1.9 Hz, 3H), 3.65 (t, *J* = 7.0 Hz, 2H), 3.42 (t, *J* = 6.6 Hz, 2H), 1.95 (q, *J* = 6.7 Hz, 2H), 1.87 (q, *J* = 6.6 Hz, 2H);^{13}C{^{1}H} NMR (101 MHz, $CDCl_3$): δ 169.9, 137.4, 129.9, 128.4, 127.2, 49.7, 46.3, 26.5, 24.6.

4.2.31 N-(pyridin-2-yl)benzamide **4ae**, TJB-2-010

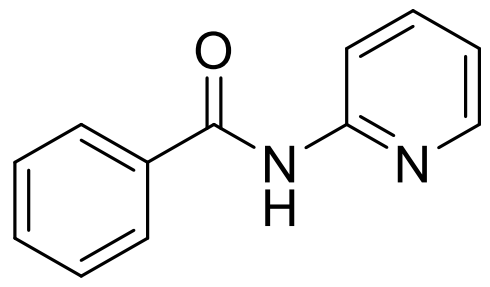

Prepared following **GP2** using **1i** (76.3 mg, 0.625 mmol) and **2h** (47.1 mg, 0.5 mmol) with 16 h reaction time. The residue was purified by flash chromatography (gradient heptane-EtOAc 100:0 to 77:23) to give **4ae** as an oil (70 mg, 0.36 mmol, 71 %). Spectral data corresponds to previously published data.[23]

R_f (EtOAc-Hept 50:50) = 0.5; IR (neat, ATR) ν_{max} 409, 519, 705, 775, 892, 992, 1149, 1301, 1429, 1462, 1517, 1594, 1671, 2058, 2327; ^{1}H NMR (500 MHz, $CDCl_3$) δ 9.14 (s, 1H), 8.41 (d, *J* = 8.4 Hz, 1H), 8.21 – 8.15 (m, 1H), 7.97 – 7.91 (m, 2H), 7.75 (ddd, *J* = 8.7, 7.3, 1.9 Hz, 1H), 7.58 – 7.53 (m, 1H), 7.51 – 7.46 (m, 2H), 7.04 (ddd, *J* = 7.4, 4.9, 1.1 Hz, 1H); ^{13}C{^{1}H} NMR (126 MHz, $CDCl_3$) δ 166.1, 151.8, 147.8, 138.7, 134.5, 132.3, 128.9, 127.5, 120.0, 114.5.

4.2.32 *tert*-Butyl (*S*)-2-(benzylcarbamoyl)pyrrolidine-1-carboxylate **4af**, MC020-338

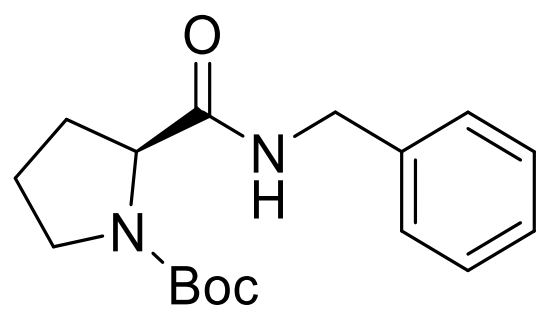

Prepared following **GP2** using **1s** (134.5 mg, 0.625 mmol) and **2a** (53.6 mg, 0.5 mmol) with 4 h reaction time. The residue was purified by flash chromatography (gradient heptane-EtOAc 100:0 to 40:60) to give **4af** as a white solid (150 mg, 0.49 mmol, 99 %). Reference sample of **4af** for enantiomeric purity measurements was prepared using **GP2** with (±)-Boc-Pro-OH. Enantiomeric ratio was determined with chiral SFC (EtOH/*i*-PrOH 1:1, 3.5 mL/min, Phenomenex LuxCel5 5µm 4,6 x150 mm) t_R (*S*) = 4.34 min (major, >99 %), t_R (*R*) = 5.39 min (minor, <1%). Spectral data corresponds to previously published data.[24]

mp 118-120 °C; R_f (EtOAc-Hept 60:40) = 0.35; IR (neat, ATR) ν_{max} 406, 586, 693, 798, 847, 898, 1077, 1127, 1167, 1200, 1247, 1309, 1370, 1392, 1453, 1476, 1652, 1681, 2872, 2977, 3310; ^{1}H NMR (400 MHz, DMSO-d$_6$): δ 8.31-8.41 (m, 1H), 7.17-7.34 (m, 5H), 4.30-4.36 (m, 1H), 4.15-4.25 (m, 1H), 4.05-4.15 (m, 1H), 3.37-3.44 (m, 1H), 3.24-3.32 (m, 1H), 2.04-2.18 (m, 1H), 1.71-1.87 (m, 3H), 1.37-1.44 (m, 3H), 1.28 (s, 6H); ^{13}C{^{1}H} NMR (101 MHz, DMSO-d$_6$, major rotamer): δ 172.4, 153.4, 139.6, 128.1, 127.3, 126.7, 78.4, 59.9, 46.5, 42.0, 31.1, 27.9, 23.1.

4.2.33 *tert*-Butyl (*R*)-(1-(benzylamino)-1-oxo-3-phenylpropan-2-yl)carbamate **4ag**, MC020-317

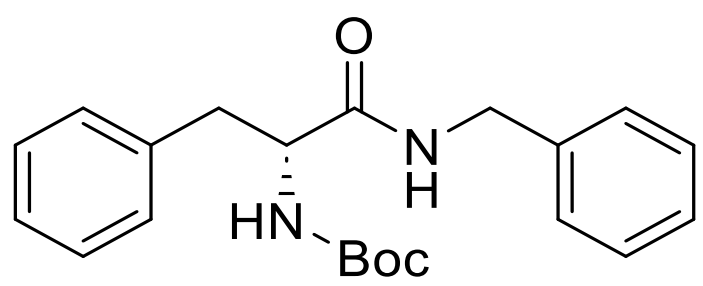

Prepared following **GP2** using **1t** (165.8 mg, 0.625 mmol) and **2a** (53.6 mg, 0.5 mmol) with 4.5 h reaction time. The residue was purified by flash chromatography (gradient heptane-EtOAc 100:0 to 65:35) to give **4ag** as a white solid (175 mg, 0.49 mmol, 99 %). Reference sample of **4ag** for enantiomeric purity measurements was prepared using **GP2** with (±)-Boc-Phe-OH. Enantiomeric ratio was determined with chiral SFC (EtOH/*i*-PrOH 1:1, 3.5 mL/min, Phenomenex LuxAMY1 5µm 4,6 x150 mm) t_R (*S*) = 4.23 min (minor, 5 %), t_R (*R*) = 4.79 min (major, 95 %). Spectral data corresponds to previously published data.[25]

mp 130-132 °C; R_f (EtOAc-Hept 60:40) = 0.64; IR (neat, ATR) ν_{max} 431, 487, 629,695, 779, 865, 1000, 1043, 1164, 1291, 1366, 1453, 1518, 1656, 680, 2928, 3027, 3062, 3332; ^{1}H NMR (400 MHz, CDCl$_3$): δ 7.37 – 7.24 (m, 6H), 7.25 – 7.20 (m, 2H), 7.12 (d, *J* = 6.9 Hz, 2H), 6.13 (s, 1H), 5.10 (s, 1H), 4.38 (d, *J* = 5.9 Hz, 2H), 3.18 – 3.00 (m, 2H), 1.42 (s, 9H);^{13}C{^{1}H}

NMR (101 MHz, $CDCl_3$): δ 171.2, 155.5, 137.8, 136.8, 129.5, 128.9, 128.7, 127.8, 127.6, 127.1, 80.4, 56.2, 43.6, 38.7, 28.4.

4.2.34 *tert*-Butyl (*S*)-2-(((*S*)-1-phenylethyl)carbamoyl)pyrrolidine-1-carboxylate **4ah**, MC020-363, MC030-370

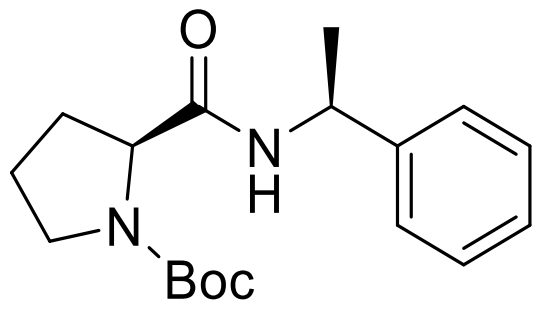

Prepared following **GP2** using **1s** (134.5 mg, 0.625 mmol) and **2l** (60.6, 0.5 mmol) with 7 h reaction time. The residue was purified by flash chromatography (gradient heptane-EtOAc 100:0 to 52:42) to give **4ah** as a white solid (130 mg, 0.44 mmol, 87 %). Reference sample of **4ah** for diastereomeric purity measurements was prepared using **GP2** with (±)-Boc-Pro-OH. Diastereomeric ratio was determined with chiral SFC (EtOH/*i*-PrOH 1:1, 3.5 mL/min, Phenomenex LuxCel2 5μm 4,6 x150 mm) t_R (*S,S*) = 4.00 min (major, >99 %), t_R (*R,S*) = 4.50 min (minor, <1 %). Spectral data corresponds to previously published data.[26]

mp 96-100 °C; R_f (EtOAc-Hept 50:50) = 0.4; IR (neat, ATR) ν_{max} 416, 493, 535, 58, 665, 760, 881, 918, 1021, 1119, 1276, 1349, 1451, 1495, 1586, 1651, 1668, 1694, 2876, 2930, 2972, 3065, 3283, 3529; ^{1}H NMR (600 MHz, DMSO-d$_6$, 85°C): δ 7.85-8.00 (m, 1H), 7.29-7.33 (m, 4H), 7.18-7.24 (m, 1H), 4.96 (quin, *J* = 7.2 Hz, 1H), 4.13 (dd, *J* = 8.4, 3.2 Hz, 1H), 3.34-3.43 (m, 1H), 3.29-3.34 (m, 1H), 2.03-2.15 (m, 1H), 1.71-1.83 (m, 3H), 1.34-1.43 (m, 12H); ^{13}C{^{1}H} NMR (151 MHz, DMSO-d$_6$, 85°C): δ 171.8, 154.1, 145.0, 128.6, 126.9, 126.3, 79.0, 60.3, 48.1, 47.1, 31.1, 28.6, 23.8, 22.5.

4.2.35 *tert*-Butyl (*S*)-2-(4-benzylpiperazine-1-carbonyl)pyrrolidine-1-carboxylate **4ai**, MC020-366

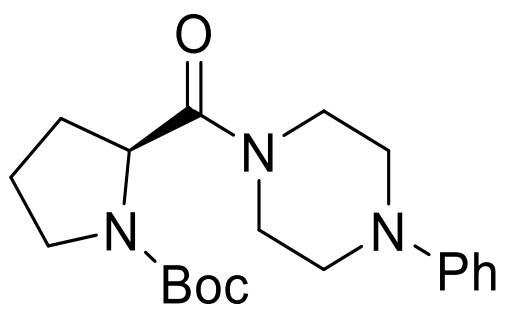

Prepared following **GP2** using **1s** (134.5 mg, 0.625 mmol) and **2d** (81.1 mg, 0.5 mmol) with 13 h reaction time. The residue was purified by flash chromatography (gradient heptane-EtOAc 100:0 to 38:62) to give **4ai** as an oil (110 mg, 0.31 mmol, 61 %). Reference sample of **4ai** for enantiomeric purity measurements was prepared using **GP2** with (±)-Boc-Pro-OH. Enantiomeric ratio was determined with chiral SFC (MeOH, 3.5 mL/min, Phenomenex LuxCel2 5μm 4,6 x150 mm) t_R (*S*) = 4.67 min (major, 97 %), t_R (*R*) = 5.44 min (minor, 3 %).

mp 95-100 °C; R_f (EtOAc-Hept 50:50) = 0.2; IR (neat, ATR) ν_{max} 412, 518, 647, 731, 873, 909, 946, 1021, 1124, 1196, 1281, 1335, 136, 1502, 1653, 1692, 2820, 2880, 2970; ^{1}H NMR (400 MHz, DMSO-d$_6$): δ 7.23 (dd, *J* = 8.7, 7.3 Hz, 2H), 6.93-6.98 (m, 2H), 6.82 (t, *J* = 7.2 Hz, 1H), 4.60-4.70 (m, 1H), 3.39 (br s, 5H), 2.88-3.29 (m, 5H), 2.11-2.26 (m, 1H), 1.70-1.85 (m, 3H), 1.33-1.41 (m, 4H), 1.30 (s, 5H); ^{13}C{^{1}H} NMR (101 MHz, DMSO-d$_6$, major rotamer): δ 170.4, 153.2, 150.8, 129.0, 119.5, 115.9, 78.2, 56.4, 48.9, 48.5, 46.4, 44.4, 41.4, 29.9, 28.1, 23.0; HRMS (ESI+): m/z calcd for $[C_{20}H_{29}N_3O_3+H]^+$ = 360.2287, found 360.2303, Δ = 4.4 ppm.

4.2.36 Methyl (*tert*-butoxycarbonyl)glycyl-(*S*)-phenylalaninate, **4aj**, MC020-410

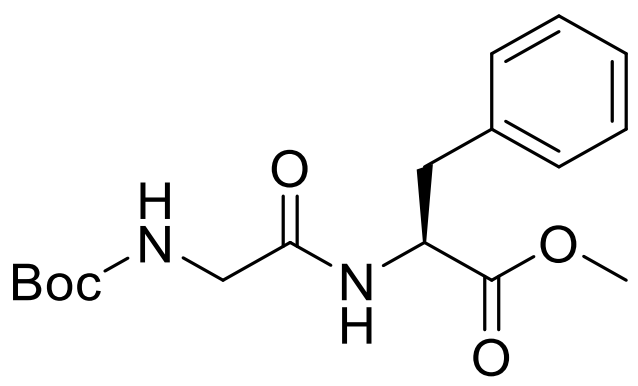

Prepared following **GP3** using **1u** (109.5mg, 0.625 mmol) and **2m** (89.6 mg, 0.5 mmol) with 13 h reaction time at 85 °C. The residue was purified by flash chromatography (gradient heptane-EtOAc 100:0 to 55:45) to give **4aj** as an oil (93 mg, 0.28 mmol, 55 %). Reference sample of **4aj** for enantiomeric purity measurements was prepared using **GP3** with (±)-Phe-OMe. Enantiomeric ratio was determined with chiral SFC (ACN/MeOH 1:1, 3.5 mL/min, Phenomenex LuxCel2 5µm 4,6 x150 mm) t_R (*S*/L) = 3.95 min (major, 94 %), t_R (*R*/D) = 4.96 min (minor, 6 %). Spectral data corresponds to previously published data.[27]

R_f (EtOAc-Hept 60:40): 0.5; IR (neat, ATR) ν_{max} 492, 700, 734, 861, 1079, 1213, 1247, 1268, 1366, 1437, 1498, 1669, 1740, 2932, 2977, 3308; IR (neat, ATR) ν_{max} ; ^{1}H NMR (400 MHz, CDCl$_3$): δ 7.21-7.30 (m, 3H), 7.09-7.13 (m, 2H), 6.79 (br s, 1H), 5.36 (br s, 1H), 4.86 (q, *J* = 7.5 Hz, 1H), 3.88 (s, 2H), 3.67-3.72 (m, 3H), 3.11 (qd, *J* = 9.7, 5.7 Hz, 2H), 1.44 (s, 9H); ^{13}C{^{1}H} NMR (101 MHz, CDCl$_3$): δ 171.7, 169.3, 155.9, 135.6, 129.1, 128.5, 127.0, 80.1, 53.0, 52.2, 44.0, 37.8, 28.2.

4.2.37 Methyl (((9H-fluoren-9-yl)methoxy)carbonyl)glycyl-(*S*)-phenylalaninate **4ak**, MC020-388, MC020-406

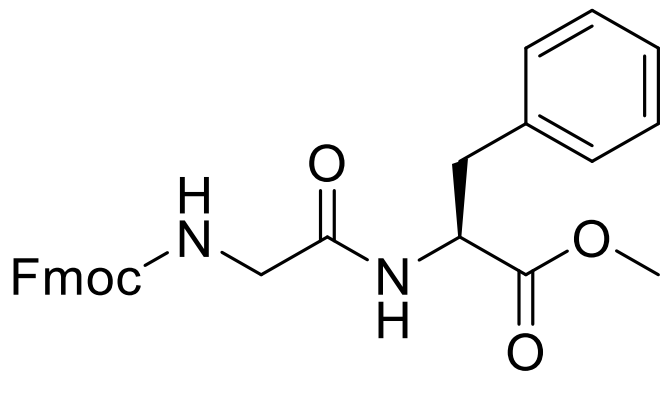

Prepared following **GP3** using **1v** (185.8 mg, 0.625 mmol) and **2m** (89.6 mg, 0.5 mmol) with 7 h reaction time at 130 °C. The residue was purified by reverse phase flash

chromatography (gradient 10 % NH_2OH (aq.)-ACN 90:10 to 40:60) to give **4ak** as white solid (130 mg, 0.29 mmol, 57 %). Reference sample of **4ak** for enantiomeric purity measurements was prepared using **GP3** with (±)-Phe-OMe. Enantiomeric ratio was determined with chiral SFC (ACN/MeOH 1:1, 3.5 mL/min, Phenomenex LuxCel4 5µm 4,6 x150 mm) t_R (*S*/L) = 6.59 min (major, 94 %), t_R (*R*/D) = 7.81 min (minor, 6 %). The reaction was repeated with **GP3** with 13 h reaction time at 85 °C. The residue was purified by reverse phase flash chromatography (gradient 10 % NH_2OH (aq.)-ACN 90:10 to 40:60) to give **4ak** as white solid (83 mg, 0.18 mmol, 36 %). Diastereomeric ratio was determined Enantiomeric ratio was determined with chiral SFC (ACN/MeOH 1:1, 3.5 mL/min, Phenomenex LuxCel4 5µm 4,6 x150 mm) t_R (*S*/L) = 6.63 min (major, 97 %), t_R (*R*/D) = 7.85 min (minor, 3 %). Spectral data corresponds to previously published data.[27]

mp 64-68 °C; R_f (EtOAc-Hept 60:40) = 0.4; IR (neat, ATR) v_{max} 425, 583, 740, 759, 1214, 1319, 1447, 1478, 1665, 1725, 2949, 3064, 3306; ^{1}H NMR (400 MHz, $CDCl_3$): δ 7.79 (d, *J* = 7.6 Hz, 2H), 7.61 (br d, *J* = 7.0 Hz, 2H), 7.43 (t, *J* = 7.5 Hz, 2H), 7.30-7.38 (m, 2H), 7.20-7.30 (m, 4H), 7.10 (d, *J* = 7.0 Hz, 2H), 6.38 (br d, *J* = 7.5 Hz, 1H), 5.40 (br s, 1H), 4.88-4.95 (m, 1H), 4.43 (d, *J* = 7.2 Hz, 2H), 4.24 (t, *J* = 7.0 Hz, 1H), 3.83-3.96 (m, 2H), 3.75 (s, 3H), 3.09-3.2 (m, 2H); ^{13}C{^{1}H} NMR (101 MHz, $CDCl_3$): δ 171.8, 168.5, 156.7, 143.9, 141.4, 135.6, 129.4, 128.8, 127.9, 127.4, 127.3, 125.2, 120.2, 67.5, 53.2, 52.6, 47.2, 44.5, 38.0.

4.2.38 *tert*-Butyl (*R*)-(1-oxo-3-phenyl-1-(4-phenylpiperazin-1-yl)propan-2-yl)carbamate **4al**, MC020-374

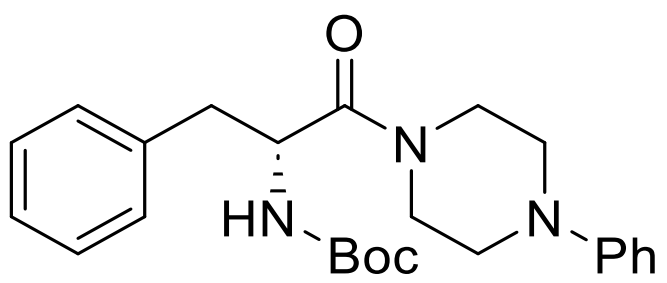

Prepared following **GP2** using **1t** (165.8 mg, 0.625 mmol) and **2d** (81.1 mg, 0.5 mmol) with 13 h reaction time. The residue was purified by flash chromatography (gradient heptane-EtOAc 100:0 to 38:62) to give **4al** as an oil (110 mg, 0.27 mmol, 54 %). Reference sample of **4al** for enantiomeric purity measurements was prepared using **GP2** with (±)-Boc-Phe-OH. Enantiomeric ratio was determined with chiral SFC (EtOH/ *i*-PrOH 1:1, 3.5 mL/min, Phenomenex LuxCel1 5µm 4,6 x150 mm) t_R (*R*) = 3.758 min (major, 72 %), t_R (*S*) = 4.30 min (minor, 27 %).

R_f (EtOAc-Hept 50:50) = 0.2; IR (neat, ATR) v_{max} 410, 615, 663, 736, 775, 918, 1012, 1076, 1163, 1237, 1291, 1352, 1435, 1473, 1524, 1630, 1695, 2828, 2918, 2978, 3000, 3031, 3269; ^{1}H NMR (400 MHz, DMSO-d): δ 7.16-7.28 (m, 8H), 6.86-6.92 (m, 2H), 6.76-6.83 (m, 1H), 4.63 (br d, *J* = 7.1 Hz, 1H), 3.44-3.64 (m, 4H), 2.87-3.09 (m, 4H), 2.75-2.84 (m, 2H), 1.29-1.34 (m, 9H); ^{13}C{^{1}H}NMR (101 MHz, DMSO-d_6: δ 169.8, 155.1, 150.7, 137.7, 129.4, 129.1, 129.0, 128.1, 126.3, 119.4, 115.8, 78.1, 51.2, 48.4, 48.4, 48.3, 44.7, 41.4, 37.5,

28.2; HRMS (ESI+): m/z calcd for $[C_{24}H_{31}N_3O_3+H]^+$ = 410.2438, found 410.2431, Δ = –1.7 ppm.

4.2.39 *tert*-Butyl (*R*)-(1-oxo-3-phenyl-1-(pyrrolidin-1-yl)propan-2-yl)carbamate **4am**, MC020-368, MC020-375

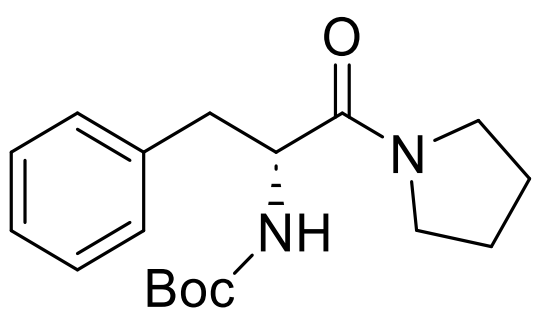

Prepared following **GP2** using **1t** (165.8 mg, 0.625 mmol) and **2c** (35.6 mg, 0.5 mmol) with 14 h reaction time. The residue was purified by flash chromatography (gradient heptane-EtOAc 100:0 to 50:50) to give **4am** as an oil (90 mg, 0.29 mmol, 57 %). Reference sample of **4am** for enantiomeric purity measurements was prepared using **GP2** with (±)-Boc-Phe-OH. Enantiomeric ratio was determined with chiral SFC (ACN/MeOH 1:1, 3.5 mL/min, Phenomenex LuxAMY2 5µm 4,6 x150 mm) t_R (*S*) = 3.11 min (minor, 21 %), t_R (*R*) = 3.59 min (major, 79 %). Spectral data corresponds to previously published data.[28]

R_f (EtOAc-Hept 50:50) = 0.3; IR (neat, ATR) ν_{max} 463, 516, 575, 590, 746, 915, 1052, 1162, 1340, 1523, 1703, 2875, 2974, 3028, 3271; ^{1}H NMR (400 MHz, $CDCl_3$): δ 7.31 – 7.19 (m, 6H), 5.48 (d, *J* = 8.7 Hz, 1H), 4.60 (td, *J* = 8.9, 6.0 Hz, 1H), 3.49 – 3.28 (m, 3H), 2.98 (qd, *J* = 12.8, 7.4 Hz, 2H), 2.58 (q, *J* = 5.1 Hz, 1H), 1.75 (tt, *J* = 12.6, 6.4 Hz, 2H), 1.65 (q, *J* = 8.0 Hz, 1H), 1.55 (q, *J* = 8.5 Hz, 1H), 1.43 (s, 9H);^{13}C{^{1}H} NMR (101 MHz, $CDCl_3$): δ 170.2, 155.3, 136.7, 129.6, 128.5, 127.0, 79.7, 53.7, 46.4, 45.8, 40.3, 28.5, 25.9, 24.1.

4.2.40 Benzyl (*R*)-2-(((*S*)-1-methoxy-1-oxo-3-phenylpropan-2-yl)carbamoyl)pyrrolidine-1-carboxylate **4an**, MC020-384, MC020-405

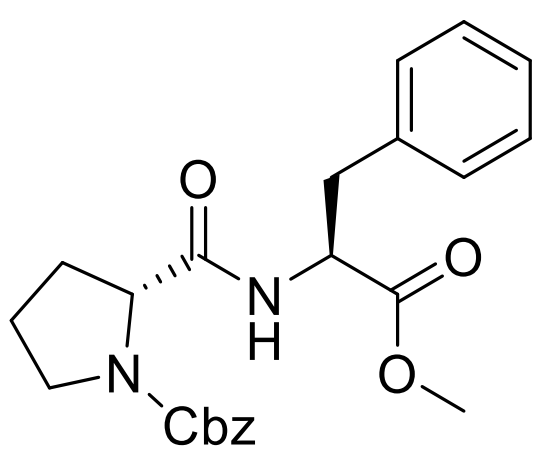

Prepared following **GP3** using **1w** (155.8 mg, 0.625 mmol) and **2m** (89.6 mg, 0.5 mmol) with 8.5 h reaction time at 130 °C. The residue was purified by flash chromatography (gradient heptane-EtOAc 100:0 to 50:50) to give **4an** as an oil (140 mg, 0.29 mmol, 70 %). Reference sample of **4an** for diastereomeric purity measurements was prepared using **GP3** with (±)-Cbz-Pro-OH. Diastereomeric ratio was determined with chiral SFC (ACN/MeOH 1:1, 3.5 mL/min, Phenomenex LuxAMY1 5µm 4,6 x150 mm) t_R (*R,R*/ D,D) = 3.70 min (minor, 12 %), t_R (*R,S*/ D,L) = 4.02 min (major, 88 %). The reaction was repeated

with **GP3** with 12 h reaction time at 85 °C. The residue was purified by flash chromatography (gradient heptane-EtOAc 100:0 to 60:40) to give **4an** as an oil (45 mg, 0.11 mmol, 22 %). Diastereomeric ratio was determined with chiral SFC (ACN/MeOH 1:1, 3.5 mL/min, Phenomenex LuxAMY1 5µm 4,6 x150 mm) t_R (*R,R*/ D,D) = 3.63 min (minor, 7 %), t_R (*R,S*/ D,L) = 3.97 min (major, 93 %).

R_f (EtOAc-Hept 60:40): 0.4; IR (neat, ATR) ν_{max} 492, 610, 733, 768, 1088, 1115, 1204, 1267, 1353, 1412, 1497, 1520, 1585, 1675, 1742, 2881, 2952, 3030, 3062, 3307; ^{1}H NMR (600 MHz, DMSO-d$_6$, 85°C): δ 8.02 (br s, 1H), 7.27-7.38 (m, 5H), 7.22-7.27 (m, 2H), 7.17-7.21 (m, 3H), 4.95-5.08 (m, 2H), 4.57 (td, *J* = 8.6, 5.5 Hz, 1H), 4.20-4.27 (m, 1H), 3.53-3.66 (m, 3H), 3.34-3.43 (m, 2H), 2.87-3.06 (m, 4H), 1.96-2.07 (m, 1H), 1.66-1.77 (m, 2H), 1.58-1.63 (m, 1H); ^{13}C{^{1}H} NMR (101 MHz, DMSO-d$_6$, major rotamer): δ = 172.3, 154.2, 137.7, 137.5, 129.6, 128.8, 128.6, 128.6, 127.9, 127.3, 126.9, 66.1, 59.6, 53.5, 52.3, 47.4, 37.3, 31.6, 23.3; HRMS (ESI+): m/z calcd for $[C_{23}H_{26}N_2O_5+H]^+$ = 411.1915, found 411.1920, Δ = 1.2 ppm

4.2.41 (*R*)-Methyl 2-(2-(*tert*-butoxycarbonylamino)-3-phenylpropanamido)acetate **4ao**, MC020-383

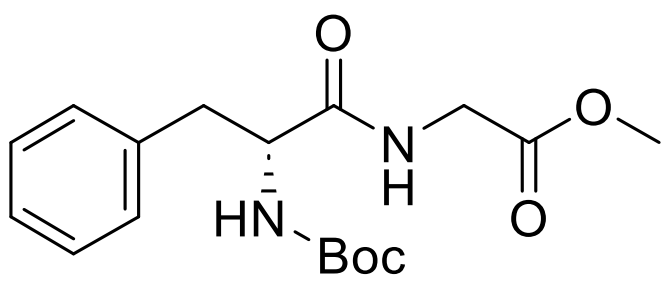

Prepared following **GP3** using **1t** (165.8 mg, 0.625 mmol) and **2n** (55.7 mg, 0.5 mmol) with 10 h reaction time at 130 °C. The residue was purified by flash chromatography (gradient heptane-EtOAc 100:0 to 60:40) to give **4ao** as an oil (25 mg, 0.08 mmol, 15 %). Reference sample of **4ao** for enantiomeric purity measurements was prepared using **GP3** with (±)-Boc-Phe-OH. Diastereomeric ratio was determined with chiral SFC (ACN/MeOH 1:1, 3.5 mL/min, Phenomenex LuxAMY1 5µm 4,6 x150 mm) t_R (*S*) = 3.50 min (minor, 2 %), t_R (*R*) = 3.82 min (major, 98 %). Spectral data corresponds to previously published data.[29]

R_f (EtOAc-Hept 60:40) = 0.5; IR (neat, ATR) ν_{max} 485, 552, 593, 661, 759, 856, 1025, 1110, 1207, 1302, 1364, 1414, 1455, 1532, 1605, 1690, 1760, 2929, 2975, 3264; ^{1}H NMR (400 MHz, $CDCl_3$): δ 7.20-7.32 (m, 5H), 6.63 (br s, 1H), 5.14 (br d, *J* = 8.2 Hz, 1H), 4.44 (br s, 1H), 3.89-4.10 (m, 2H), 3.74 (s, 3H), 2.97-3.20 (m, 2H), 1.40 (s, 9H); ^{13}C{^{1}H} NMR (101 MHz, $CDCl_3$): δ 171.8, 170.1, 155.6, 136.7, 129.5, 129.4, 128.7, 127.0, 80.4, 55.7, 52.5, 41.3, 38.5, 28.4.

4.2.42 Methyl (*tert*-butoxycarbonyl)-(*R)*-phenylalanyl-*(S)*-methioninate **4ap**, MC020-389, MC020-409

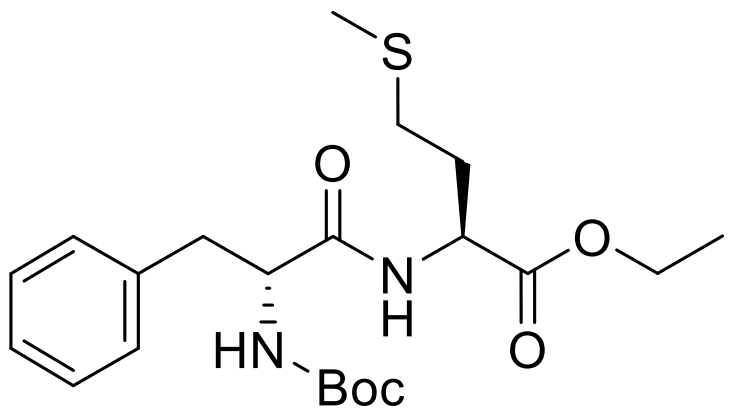

Prepared following **GP3** using **1t** (165.8 mg, 0.625 mmol) and **2o** (102.0 mg, 0.5 mmol) with 7 h reaction time at 130 °C. The residue was purified by reverse phase flash chromatography (gradient 10 % NH_2OH (aq.)-ACN 90:10 to 45:55) to give **4ap** as off-white solid (110 mg, 0.27 mmol, 53 %). Reference sample of **4ap** for diastereomeric purity measurements was prepared using **GP3** with (±)-Boc-Phe-OH. Diastereomeric ratio was determined with chiral SFC (ACN/MeOH 1:1, 3.5 mL/min, Phenomenex LuxAMY1 5µm 4,6 x150 mm) t_R (*R,R/* D,D) = 6.23 min (minor, 22 %), t_R (*R,S/*D,L) = 8.37 min (major, 78 %). The reaction was repeated with **GP3** with 13 h reaction time at 85 °C. The residue was purified by reverse phase flash chromatography (gradient 10 % NH_2OH (aq.)-ACN 90:10 to 40:60) to give **4ap** as white solid (51 mg, 0.12 mmol, 24 %). Diastereomeric ratio was determined with chiral SFC (MeOH:IPA 1:1, 3.5 mL/min, Phenomenex LuxAMY1 5µm 4,6 x150 mm) t_R (*R,R/* D,D) = 4.01 min (minor, 9 %), t_R (*R,S/*D,L) = 4.82 min (major, 91 %).

mp 82-86 °C; R_f (EtOAc-Hept 60:40) = 0.7; IR (neat, ATR) ν_{max} 465, 501, 591, 752, 777, 989, 1021, 1062, 1096, 1117, 1163, 1244, 1294, 1366, 1389, 144, 1516, 1660, 1682, 1734, 2917, 2971, 3337; ^{1}H NMR (600 MHz, DMSO-d$_6$, two romaters): δ 8.32 (br d, *J* = 7.9 Hz, 1H), 7.21-7.30 (m, 4H), 7.16-7.21 (m, 1H), 6.83-6.92 (m, 1H), 4.26-4.42 (m, 1H), 4.17-4.26 (m, 1H), 4.06-4.13 (m, 2H), 2.85-2.98 (m, 1H), 2.75 (br dd, *J* = 13.4, 9.7 Hz, 1H), 2.26-2.41 (m, 2H), 1.99-2.07 (m, 3H), 1.79-1.94 (m, 2H), 1.22-1.33 (m, 9H), 1.15-1.21 (m, 3H); ^{13}C{^{1}H} NMR (151 MHz, DMSO-d$_6$): δ = 172.2, 172.1, 155.5, 138.3, 129.7, 128.5, 126.7, 78.5, 61.1, 56.0, 51.4, 38.3, 31.0, 29.8, 28.6, 15.0, 14.5, 14.5; HRMS (ESI+): m/z calcd for $[C_{21}H_{32}N_2O_5S+H]^+$ = 425.2105, found 425.2153, Δ = 10.8 ppm.

4.3 Synthesis of boronic acids

4.3.1 1-(2-Iodophenyl)-2,2,6,6-tetramethylpiperidine **S1a**, MC020-293

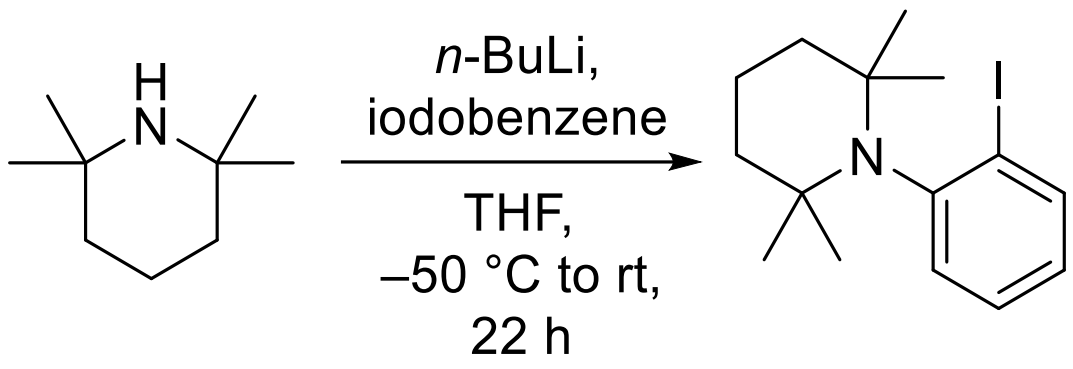

2,2,6,6-tetramethylpiperidine (2.00 g, 14.0 mmol, 1 equiv.) in dry THF (20 mL) was cooled to -40 °C. *n*-BuLi (5.6 mL, 14 mmol, 1 equiv.) was added dropwise and the mixture was stirred for 30 min at –40 °C. The mixture was cooled to –50 °C and iodobenzene (5.83 g, 28 mmol, 2 equiv.) in dry THF (7 mL) was added dropwise. NOTE: *n*-Butyllithium is extremely flammable and can ignite spontaneously upon contact with air or moisture; accordingly, it must be handled strictly under an inert atmosphere. Iodobenzene is harmful if inhaled or absorbed through the skin, and therefore proper personal protective equipment (safety goggles, gloves, and lab coat) should be worn at all times. All work should be conducted in a well-ventilated fume hood. The mixture was let to slowly warm up to rt and stirred for 22 h. After, saturated aq. NH_4Cl (10 mL) was added, and the biphasic mixture was extracted with heptane (3 × 20 mL). The combined organic layers were dried using phase separator and concentrated. The residue was purified by flash chromatography (heptane) to give impure tert-butyl (2-(2-phenylacetamido)ethyl)carbamate as a colourless oil. The remainings were purified again by reverse phase flash chromatography (ACN) to give **S1a** as a colourless oil (2.70 g, 7.98 mmol, 57 %). Spectral data corresponds to previously published data.[30]

R_f (EtOAc-Hept 60:40) = 0.71; IR (neat, ATR) ν_{max} 454,563, 633, 747, 839, 925, 947, 992, 1013, 1059, 1242, 1349, 1363, 1422, 1457, 2848, 2867, 2919, 3005; ^{1}H NMR (400 MHz, $CDCl_3$): δ 7.94 (dd, *J* = 7.9, 1.6 Hz, 1H), 7.42 (dd, *J* = 7.9, 1.7 Hz, 1H), 7.29 (ddd, *J* = 7.9, 7.2, 1.6 Hz, 1H), 6.89 (ddd, *J* = 7.9, 7.2, 1.6 Hz, 1H), 1.95 – 1.86 (m, 1H), 1.84 – 1.75 (m, 2H), 1.68 – 1.61 (m, 1H), 1.59 – 1.54 (m, 2H), 1.29 (s, 6H), 0.91 (s, 6H).^{13}C{^{1}H} NMR (101 MHz, $CDCl_3$): δ 149.1, 139.9, 131.8, 128.2, 127.4, 114.5, 56.3, 41.4, 31.7, 25.8, 18.8.

4.3.2 1-(2-Iodo-4-methoxyphenyl)piperidine **S1b**, MC020-343

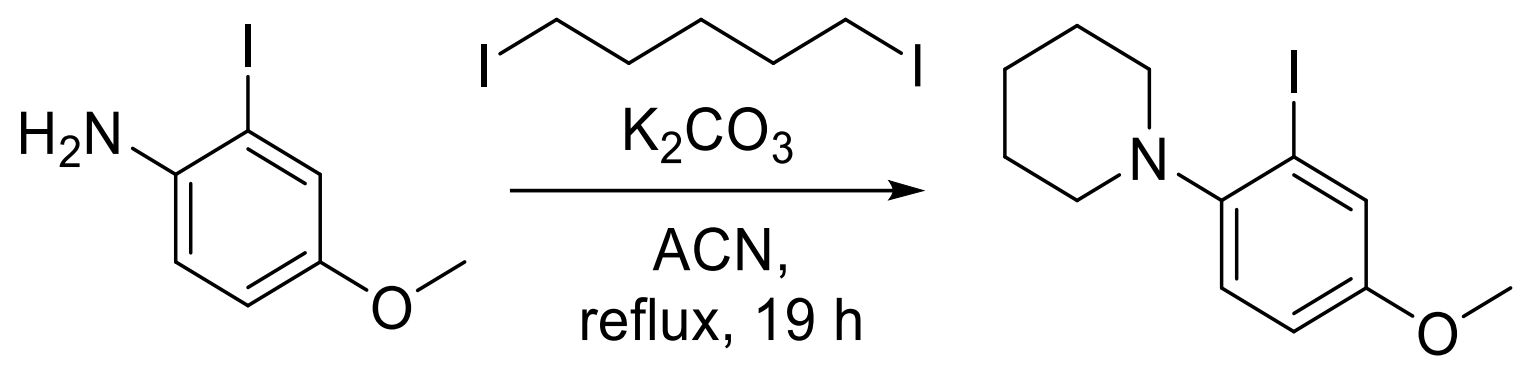

To a mixture of 2-iodo-4-methoxyaniline (1.00 g, 3.81 mmol, 1 equiv.) and K_2CO_3 (1.32 g, 9.54 mmol, 2.5 equiv.) in ACN (20 mL) was added 1,5-diiodopentane (1.95 g, 5.72 mmol, 1.5 equiv.) dropwise. The reaction was stirred at reflux for 19 hours. After, the mixture was let to warm up to rt and water (5 mL) was added. The mixture was extracted with EtOAc (3 x 15 ml), dried with phase separator and concentrated. The crude was purified with reverse phase chromatography (100 % ACN) to give **S1b** as brown oil (0.98 g, 3.09 mmol, 81 %).

R_f (EtOAc-Hept 40:60) = 0.75; IR (neat, ATR) ν_{max} 447, 493, 661, 719, 842, 919, 1029, 1107, 1149, 1208, 326, 1397, 1149, 1289, 1397, 2670, 2745, 2833, 2926, 2953, 3002, 3057; ^{1}H NMR (600 MHz, DMSO-d$_6$): δ 7.37 (d, *J* = 2.9 Hz, 1H), 7.04 (d, *J* = 8.7 Hz, 1H), 6.95 (dd, *J* = 8.7, 2.9 Hz, 1H), 3.71 (s, 3H), 2.75 (d, *J* = 4.4 Hz, 4H), 1.65 (p, *J* = 5.6 Hz, 4H), 1.50 (s, 2H); ^{13}C{^{1}H} NMR (151 MHz, DMSO-d$_6$): δ 155.9, 147.6, 124.2, 121.4, 114.7, 99.6, 55.5, 53.9, 26.0, 23.5; HRMS (ESI+): m/z calcd for $[C_{12}H_{16}INO + H]^+$ = 318.0349, found 318.0339, Δ = –3.1 ppm.

4.3.3 (2-(2,2,6,6-Tetramethylpiperidin-1-yl)phenyl)boronic acid **3s**, MC020-306

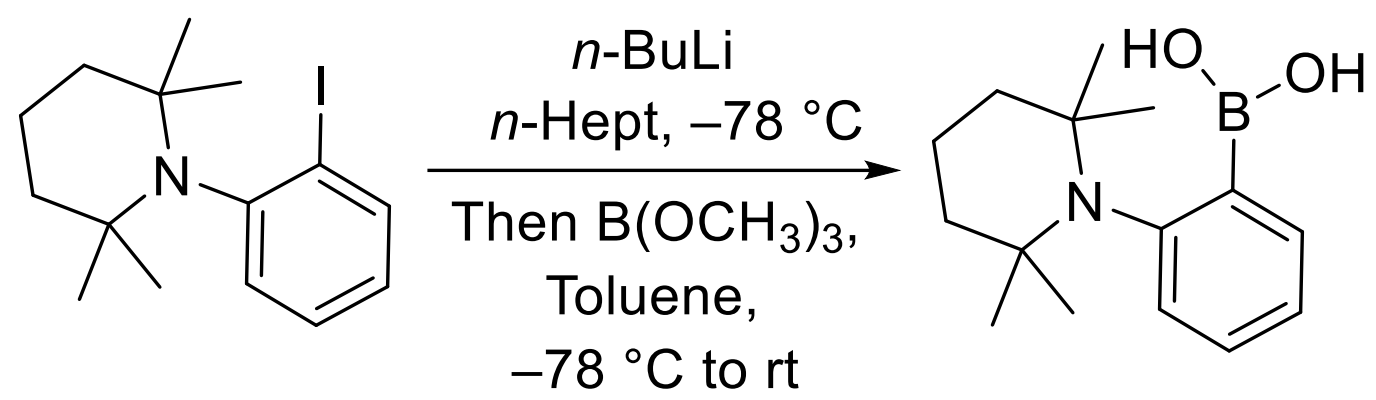

1-(2-iodophenyl)-2,2,6,6-tetramethylpiperidine(0.40 g, 1.17 mmol, 1 equiv.) in heptane (4 mL) was cooled to –78 °C and *n*-BuLi (0.80 mL, 1.198 mmol, 1.7 equiv.) was added dropwise with stirring. The mixture was let to warm up to rt and stirred for 4 h. After, the reaction mixture was concentrated and the remainings were dissolved in toluene (4 mL). The mixture was cooled to –78 °C and trimethyl borate (0.50 mL, 4.66 mmol, 4 equiv.) was added dropwise. NOTE: *n*-Butyllithium is extremely flammable and can ignite spontaneously upon contact with air or moisture; accordingly, it must be handled strictly under an inert atmosphere. Trimethyl borate is highly flammable, toxic and may damage fertility and unborn child. Therefore, appropriate personal protective equipment (safety goggles, gloves, lab coat) must be worn at all times, and all operations should be performed in a well-ventilated fume hood. The mixture was let to warm up to rt and stirred overnight. Water (2 mL) was added, and the mixture was stirred for another 3 h. The

biphasic mixture was extracted with $CHCl_3$ (3 x 10 mL), dried using phase separator and concentrated. Trituration of the crude product in 1 mL of water did not result in solid compound so the mixture was concentrated in lyophilizer. The resulting orange oil was triturated in cool heptane (5 mL) and filtered resulting in brown solid that still contained impurities. Trituration was repeated in 1:5 EtOAC:heptane mixture (5 mL) to give **3s** (20 mg, 0.08 mmol, 7 %) as brown solid. NOTE: a large amount of product was lost in the trituration part. Spectral data corresponds to previously published data.[31]

mp 180-185 °C; ^{1}H NMR (400 MHz, $CDCl_3$): δ 8.45 (br s, 1H), 7.96 (d, *J* = 7.9 Hz, 1H), 7.37-7.43 (m, 2H), 7.26-7.31 (m, 1H), 1.88-2.02 (m, 1H), 1.67-1.76 (m, 5H), 1.42 (s, 6H), 0.86 (s, 6H); ^{13}C{^{1}H} NMR (101 MHz, $CDCl_3$): δ 151.6, 135.2, 130.5, 129.6, 126.1, 56.8, 41.9, 32.2, 25.2, 18.2; ^{11}B NMR (128 MHz, $CDCl_3$): δ = 29.9.

4.3.4 (5-Methoxy-2-(piperidin-1-yl)phenyl)boronic acid **3t**, MC020-349

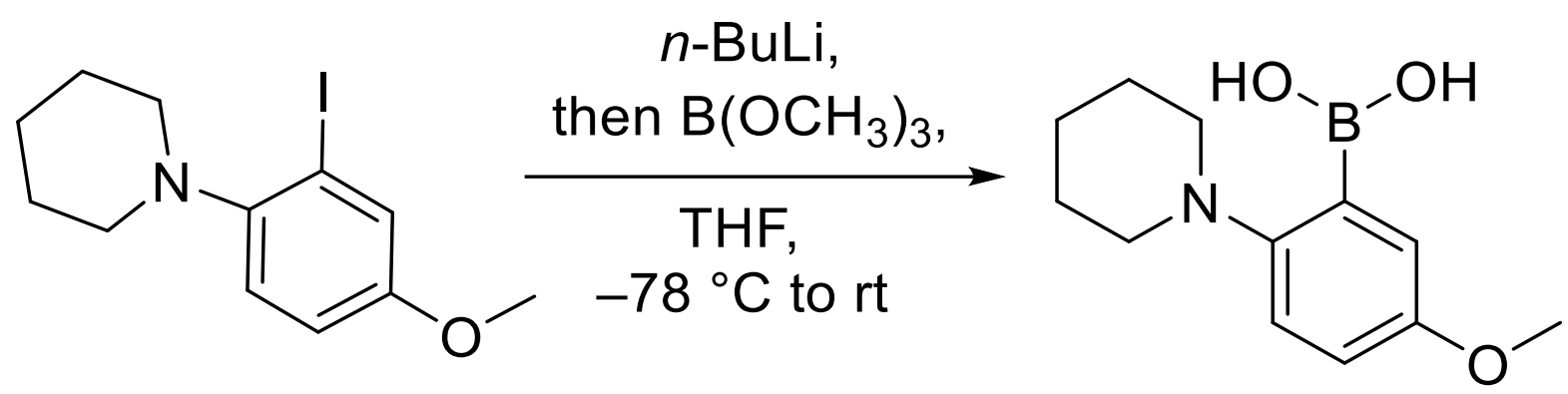

1-(2-iodo-4-methoxyphenyl)piperidine (0.50 g, 1.58 mmol, 1 equiv.) in dry THF (4 mL) was cooled to –78 °C. *n*-BuLi (0.95 mL, 2.37 mmol, 1.5 equiv.) was added dropwise and the mixture was stirred at –78 °C for 1 h. Trimethyl borate (0.21 mL, 1.89 mmol, 1.2 equiv.) was added dropwise and the mixture was let to slowly warm up to rt and stirred for 1.5 h. NOTE: *n*-Butyllithium is extremely flammable and can ignite spontaneously upon contact with air or moisture; accordingly, it must be handled strictly under an inert atmosphere. Trimethyl borate is highly flammable, toxic and may damage fertility and unborn child. Therefore, appropriate personal protective equipment (safety goggles, gloves, lab coat) must be worn at all times, and all operations should be performed in a well-ventilated fume hood. After, 2 N HCl was added to make the mixture acidic and the mixture was stirred for another 25 min. Water (2 mL) was added and the biphasic mixture was extracted with EtOAc (3 x 10 mL), dried using phase separator and concentrated. The aqueous phase was extracted again with DCM (10 mL) and the organic phase was dried and concentrated. The remainings were purified by reverse flash chromatography (gradient ACN-10 % formic acid (aq) 0:100 to 10:90) to give **3t** as a white solid (0.12 g, 0.52 mmol, 33 %).

mp 111-116 °C; IR (neat, ATR) ν_{max} 447, 521, 728, 765, 993, 1013, 1183, 1230, 1301, 1453, 1486, 1582, 1679, 2524, 2678, 2836, 2940; ^{1}H NMR (400 MHz, DMSO-d_6): δ 9.27 (s, 2H), 7.33 (d, *J* = 8.7 Hz, 1H), 7.27 (d, *J* = 3.1 Hz, 1H), 6.98 (dd, *J* = 8.7, 3.2 Hz, 1H), 3.73 (s, 3H), 2.80 (br t, *J* = 5.1 Hz, 4H), 1.60-1.71 (m, 4H), 1.54 (br d, *J* = 4.9 Hz, 2H); ^{13}C{^{1}H} NMR (101

MHz, DMSO-d_6): δ 156.3, 152.4, 122.5, 119.3, 116.9, 55.1, 55.0, 26.4, 23.0; ^{11}B NMR (128 MHz, DMSO-d_6): δ = 29.9; HRMS (ESI+): m/z calcd for $[C_{12}H_{18}BNO_3+H]^+$ = 235.1494, found 235.1518, Δ = 10.2 ppm.

5 References

(1) Pan, B.; Huang, D. M.; Sun, H. T.; Song, S. N.; Su, X. Bin. Heterocyclic Boron Acid Catalyzed Dehydrative Amidation of Aliphatic/Aromatic Carboxylic Acids with Amines. *J. Org. Chem.* **2023**, *88* (5), 2832–2840. DOI: 10.1021/acs.joc.2c02515

(2) Gernigon, N.; Zheng, H.; Hall, D. G. Solid-Supported Ortho-Iodoarylboronic Acid Catalyst for Direct Amidation of Carboxylic Acids. *Tetrahedron Lett.* **2013**, *54* (33), 4475–4478. DOI: 10.1016/j.tetlet.2013.06.043

(3) Arnold, K.; Batsanov, A. S.; Davies, B.; Whiting, A. Synthesis, Evaluation and Application of Novel Bifunctional N, N-Di-Isopropylbenzylamineboronic Acid Catalysts for Direct Amide Formation between Carboxylic Acids and Amines. *Green Chem.* **2008**, *10* (1), 124–13. DOI: 10.1039/b712008g

(4) Pan, B.; Huang, D. M.; Sun, H. T.; Song, S. N.; Su, X. Bin. Heterocyclic Boron Acid Catalyzed Dehydrative Amidation of Aliphatic/Aromatic Carboxylic Acids with Amines. *J. Org. Chem.* **2023**, *88* (5), 2832–2840. DOI: 10.1021/acs.joc.2c02515

(5) Bai, J.; Zambroń, B. K.; Vogel, P. Amides in One Pot from Carboxylic Acids and Amines via Sulfinylamides. *Org. Lett.* **2014**, *16* (2), 604–607. DOI: doi.org/10.1021/ol403508j

(6) Tosi, E.; Campagne, J. M.; De Figueiredo, R. M. Amine Activation: "Inverse" Dipeptide Synthesis and Amide Function Formation through Activated Amino Compounds. *J. Org. Chem.* **2022**, *87* (18), 12148–12163. DOI: 10.1021/acs.joc.2c01288

(7) Charvieux, A.; Le Moigne, L.; Borrego, L. G.; Duguet, N.; Métay, E. Solvent-Free N-Alkylation of Amides with Alcohols Catalyzed by Nickel on Silica–Alumina. *Eur. J. Org. Chem.* **2019**, *2019* (40), 6842–6846. DOI: 10.1002/ejoc.201901291

(8) Wang, A.; Xie, Y.; Wang, J.; Shi, D.; Yu, H. Atom-Economic Amide Synthesis by Using an Iron-Substituted Polyoxometalate Catalyst. *Chem. Commun.* **2022**, *58* (8), 1127–1130. DOI: 10.1039/D1CC05417A

(9) Hibbard, J. P.; Yam, J. G.; Alsalek, E. B.; Bahamonde, A. Mild Sustainable Amide Alkylation Protocol Enables a Broad Orthogonal Scope. *J. Org. Chem.* **2022**, *87* (18), 12036–12040. DOI: 10.1021/acs.joc.2c01028

(10) Liu, Y.; Zhou, C.; Jiang, M.; Arndtsen, B. A. Versatile Palladium-Catalyzed Approach to Acyl Fluorides and Carbonylations by Combining Visible Light- and Ligand-Driven Operations. *J. Am. Chem. Soc.* **2022**, *144* (21), 9413–9420. DOI: 10.1021/jacs.2c01951

(11) Lanigan, R. M.; Starkov, P.; Sheppard, T. D. Direct Synthesis of Amides from Carboxylic Acids and Amines Using B(OCH2CF3)3. *J. Org. Chem.* **2013**, *78* (9), 4512–4523. DOI: 10.1021/jo400509n

(12) Wiget, P. A.; Manzano, L. A.; Pruet, J. M.; Gao, G.; Saito, R.; Monzingo, A. F.; Jasheway, K. R.; Robertus, J. D.; Anslyn, E. V. Sulfur Incorporation Generally Improves Ricin Inhibition in Pterin-Appended Glycine-Phenylalanine Dipeptide Mimics. *Bioorg. Med. Chem. Lett.* **2013**, *23* (24), 6799–6804. DOI: 10.1016/j.bmcl.2013.10.017

(13) Cuesta-Galisteo, S.; Schörgenhumer, J.; Hervieu, C.; Nevado, C. Dual Nickel/Photoredox-Catalyzed Asymmetric Carbamoylation of Benzylic C(Sp3)−H Bonds. *Angew. Chem. Int. Ed.* **2024**, *63* (12). DOI: 10.1002/anie.202313717

(14) Nawrot, D.; Koutníková, B.; Janďourek, O.; Konečná, K.; Novák, M.; Paterová, P.; Bárta, P.; Bouz, G.; Zitko, J.; Doležal, M. Antimicrobial and Antiproliferative Properties of 2-Phenyl- *N* -(Pyridin-2-yl)Acetamides. *Chem. Biol. Drug Des.* **2025**, *105* (1). DOI: 10.1111/cbdd.70030

(15) Elwood, J. M. L.; Henry, M. C.; Lopez-Fernandez, J. D.; Mowat, J. M.; Boyle, M.; Buist, B.; Livingstone, K.; Jamieson, C. Functionalized Tetrazoles as Latent Active Esters in the Synthesis of Amide Bonds. *Org. Lett.* **2022**, *24* (51), 9491–9496. DOI: 10.1021/acs.orglett.2c03971

(16) Samuel Rajan, I. A. P.; Rajendran, S. Amidic Resonance Not a Barrier for Transamidation of N-Pivaloyl Activated Amides: Catalyst, Base and Additive Free Conditions. *Org. Biomol. Chem.* **2023**, *21* (23), 4760–4765. DOI: 10.1039/D3OB00418J

(17) Ye, P.; Shao, Y.; Ye, X.; Zhang, F.; Li, R.; Sun, J.; Xu, B.; Chen, J. Homoleptic Bis(Trimethylsilyl)Amides of Yttrium Complexes Catalyzed Hydroboration Reduction of Amides to Amines. *Org. Lett.* **2020**, *22* (4), 1306–1310. DOI: 10.1021/acs.orglett.9b04606

(18) Sawant, D. N.; Bagal, D. B.; Ogawa, S.; Selvam, K.; Saito, S. Diboron-Catalyzed Dehydrative Amidation of Aromatic Carboxylic Acids with Amines. *Org. Lett.* **2018**, *20* (15), 4397–4400. DOI: 10.1021/acs.orglett.8b01480

(19) Roberts, B.; Liptrot, D.; Alcaraz, L.; Luker, T.; Stocks, M. J. Molybdenum-Mediated Carbonylation of Aryl Halides with Nucleophiles Using Microwave Irradiation. *Org. Lett.* **2010**, *12* (19), 4280–4283. DOI: 10.1021/ol1016965

(20) Laha, J. K.; Gulati, U.; Gupta, A. Decarboxylative Amidation of Aryl/Heteroarylacetic Acids via Activated Esters through Traceless α-Functionalized Benzylic Radicals. *Org. Lett.* **2023**, *25* (19), 3402–3406. DOI: 10.1021/acs.orglett.3c00927

(21) Xiong, W.; Lai, G.; Liu, W. H. A Type of Stable Amides Behaves as Acyl Transfer Reagents upon Visible-Light Irradiation through Self-Aromatization. *Chem. - Eur. J.* **2024**, *30* (41). DOI: 10.1002/chem.202401619

(22) Liu, G.; Hao, F.; Liang, L.; Jin, Z.; Wu, J. Reductive Hydrodehalogenation of Halogenated Carboxylic Acid Derivatives Using a DMSO/HCOONa·2H2O System. *Org. Lett.* **2023**, *25* (15), 2691–2695. DOI: 10.1021/acs.orglett.3c00782

(23) Procter, R. J.; Alamillo-Ferrer, C.; Shabbir, U.; Britton, P.; Bučar, D.-K.; Dumon, A. S.; Rzepa, H. S.; Burés, J.; Whiting, A.; Sheppard, T. D. Borate-Catalysed Direct Amidation Reactions of Coordinating Substrates. *Chem. Sci.* **2025**, *16* (11), 4718–4724. DOI: 10.1039/D4SC07744J

(24) Bansagi, J.; Wilson-Konderka, C.; Debrauwer, V.; Narayanan, P.; Batey, R. A. N-Alkyl Carbamoylimidazoles as Isocyanate Equivalents: Exploration of the Reaction Scope for the Synthesis of Ureas, Hydantoins, Carbamates, Thiocarbamates, and Oxazolidinones. *J. Org. Chem.* **2022**, *87* (17), 11329–11349. DOI: 10.1021/acs.joc.2c00803

(25) Lenstra, D. C.; Nguyen, D. T.; Mecinović, J. Zirconium-Catalyzed Direct Amide Bond Formation between Carboxylic Esters and Amines. *Tetrahedron* **2015**, *71* (34), 5547–5553. DOI: 10.1016/j.tet.2015.06.066

(26) Due-Hansen, M. E.; Pandey, S. K.; Christiansen, E.; Andersen, R.; Hansen, S. V. F.; Ulven, T. A Protocol for Amide Bond Formation with Electron Deficient Amines and Sterically Hindered Substrates. *Org. Biomol. Chem.* **2016**, *14* (2), 430–433. DOI: 10.1039/c5ob02129d

(27) Terrey, M. J.; Perry, C. C.; Cross, W. B. Postsynthetic Modification of Phenylalanine Containing Peptides by C–H Functionalization. *Org. Lett.* **2019**, *21* (1), 104–108. DOI: 10.1021/acs.orglett.8b03536

(28) Lenstra, D. C.; Damen, E.; Leenders, R. G. G.; Blaauw, R. H.; Rutjes, F. P. J. T.; Wegert, A.; Mecinović, J. Structure–Activity Relationship Studies on (R)-PFI-2 Analogues as Inhibitors of Histone Lysine Methyltransferase SETD7. *ChemMedChem* **2018**, *13* (14), 1405–1413. DOI: 10.1002/cmdc.201800242

(29) Erapalapati, V.; Hale, U. A.; Madhavan, N. Phosphorus Pentoxide for Amide and Peptide Bond Formation with Minimal By-Products. *Tetrahedron Lett.* **2019**, *60* (49), 151311. https://doi.org/10.1016/j.tetlet.2019.151311.

(30) Chernichenko, K.; Nieger, M.; Leskelä, M.; Repo, T. Hydrogen Activation by 2-Boryl-N,N-Dialkylanilines: A Revision of Piers' Ansa-Aminoborane. *Dalton Trans.* **2012**, *41* (30), 9029. DOI: 10.1039/c2dt30926b

(31) Légaré, M.-A.; Rochette, É.; Légaré Lavergne, J.; Bouchard, N.; Fontaine, F.-G. Bench-Stable Frustrated Lewis Pair Chemistry: Fluoroborate Salts as Precatalysts for the C–H Borylation of Heteroarenes. *Chem. Commun.* **2016**, *52* (31), 5387–5390. DOI: 10.1039/C6CC01267A

6 Copies of NMR spectra and HPLC chromatograms

5.1 ^{1}H NMR spectrum 4a (400 MHz, DMSO-d$_6$)

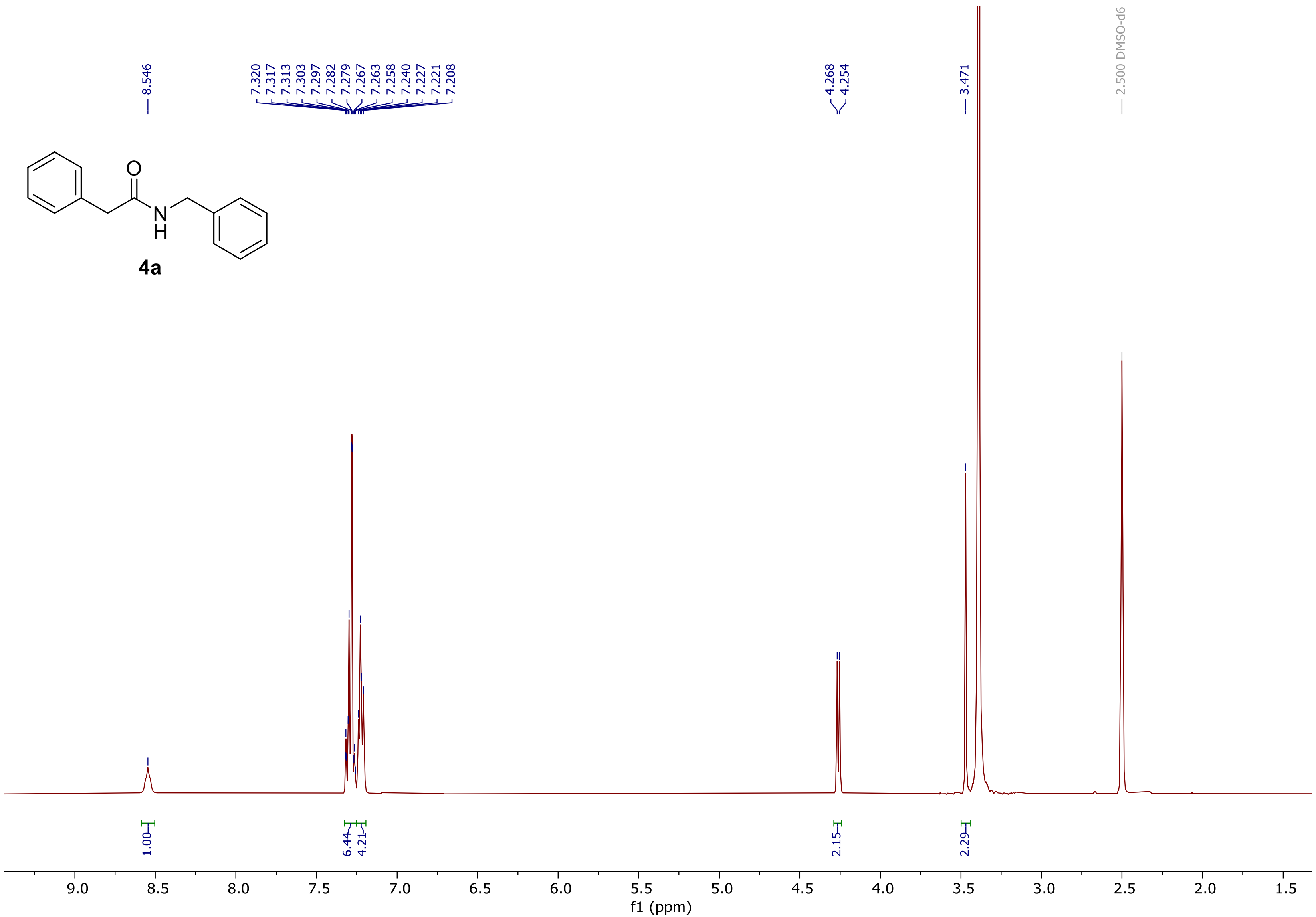

5.2 $^{13}C\{^{1}H\}$ NMR spectrum 4a (101 MHz, DMSO-d6)

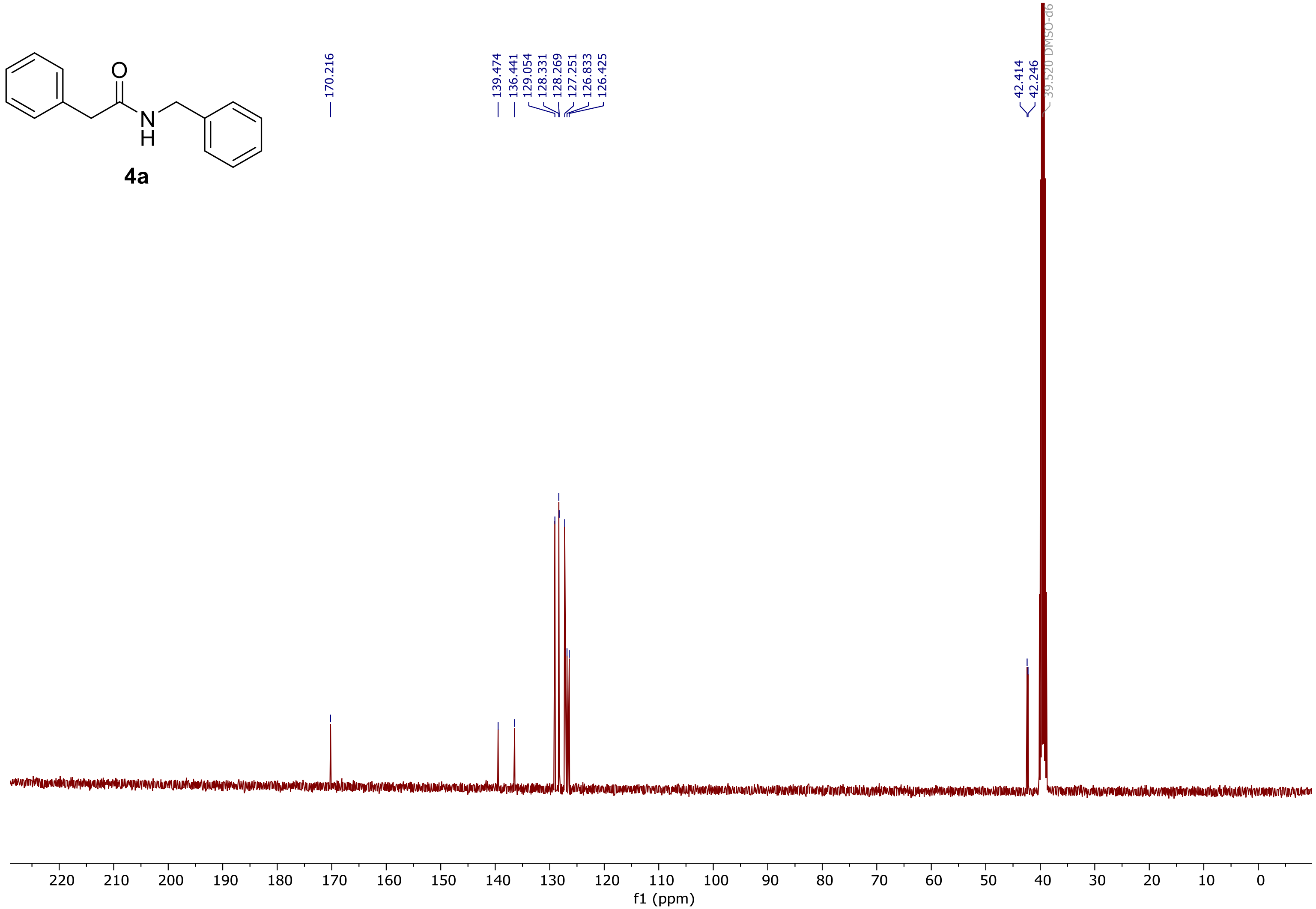

5.3 ^{1}H NMR spectrum 4b (400 MHz, DMSO-d$_6$)

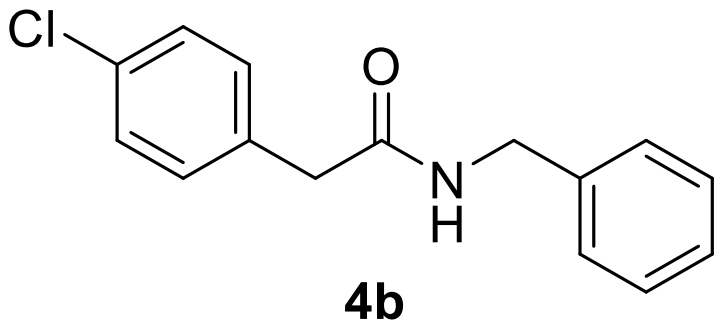

4b

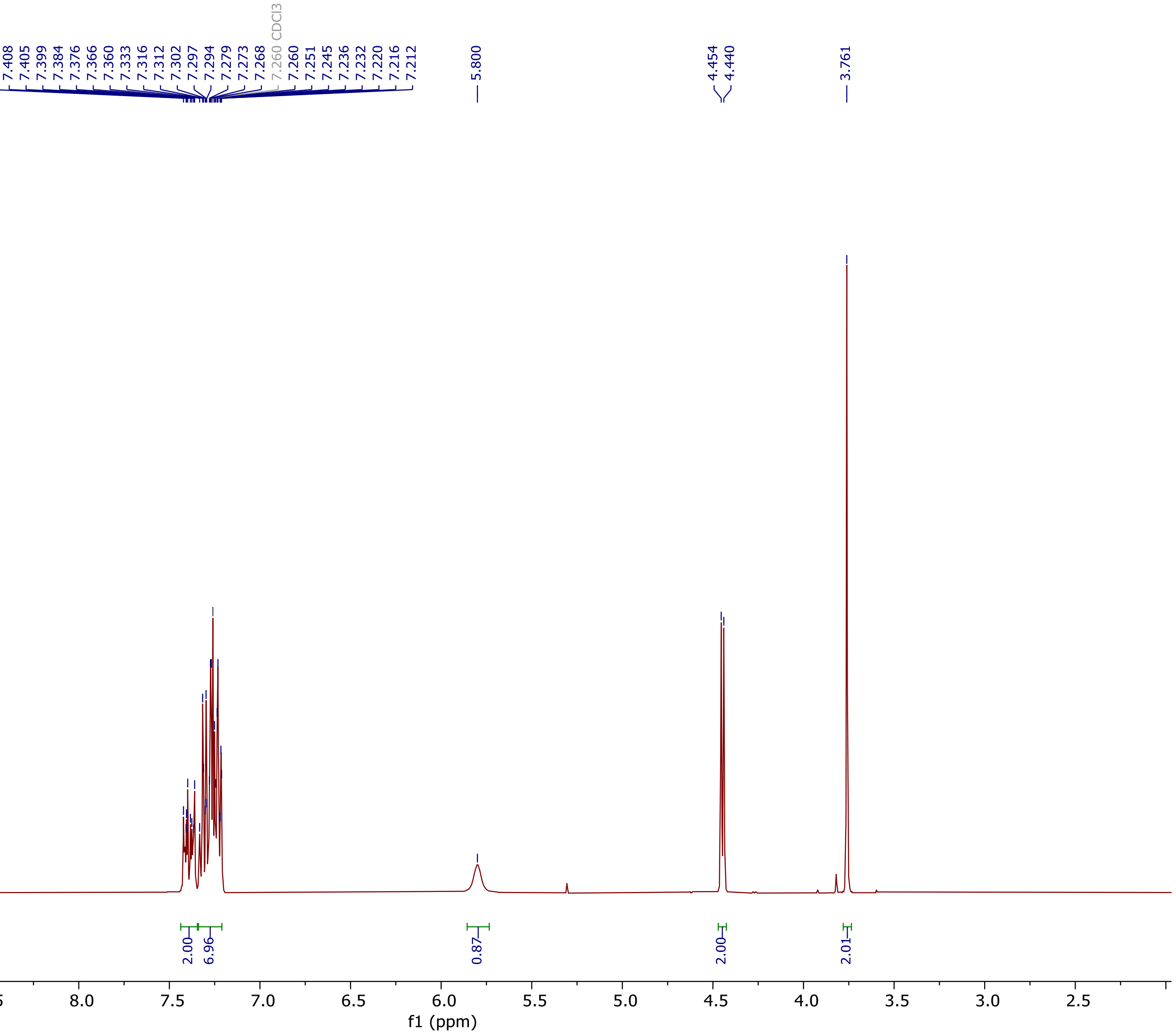

5.4 $^{13}C\{^{1}H\}$ NMR spectrum 4b (101 MHz, DMSO-d$_6$)

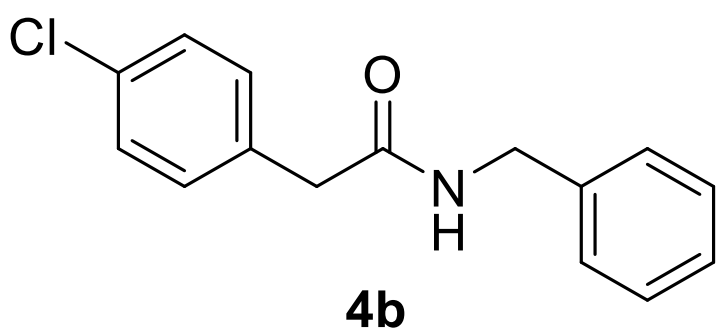

4b

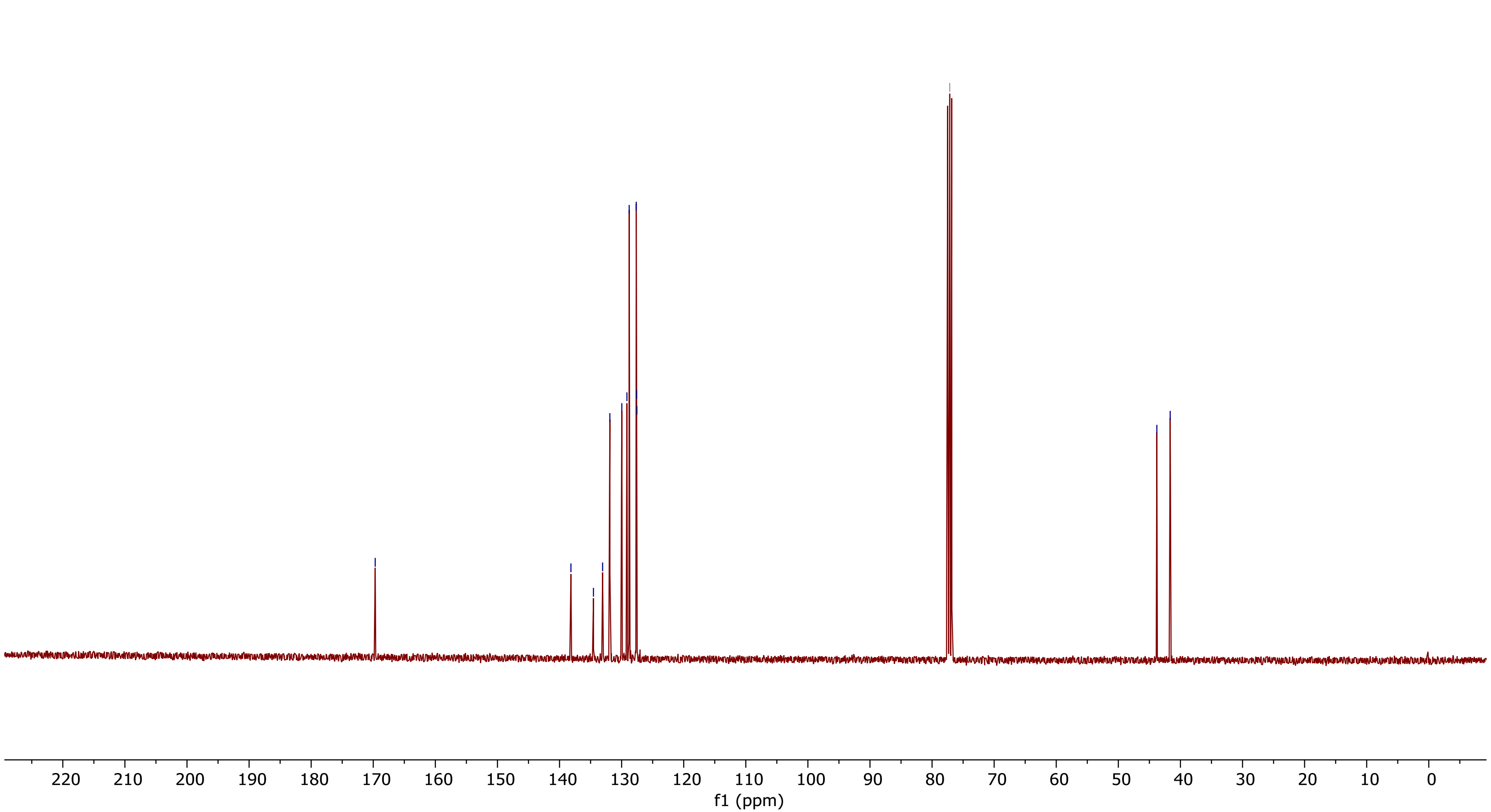

5.5 ^{1}H NMR spectrum 4c (400 MHz, DMSO-d$_6$)

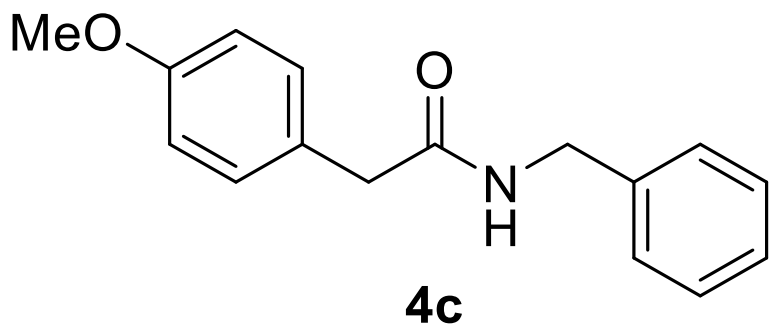

4c

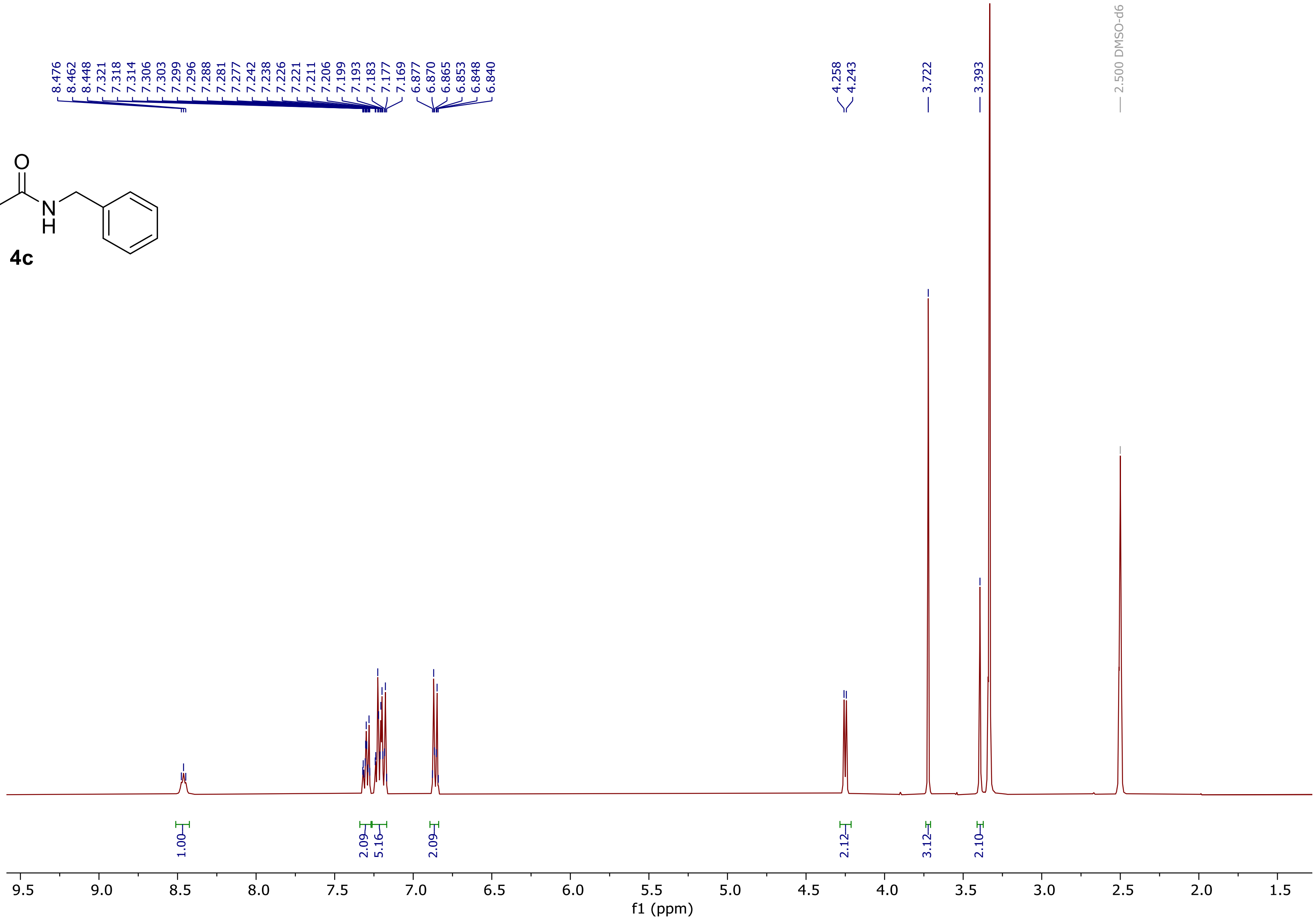

5.6 $^{13}C\{^{1}H\}$ NMR spectrum 4c (101 MHz, DMSO-d$_6$)

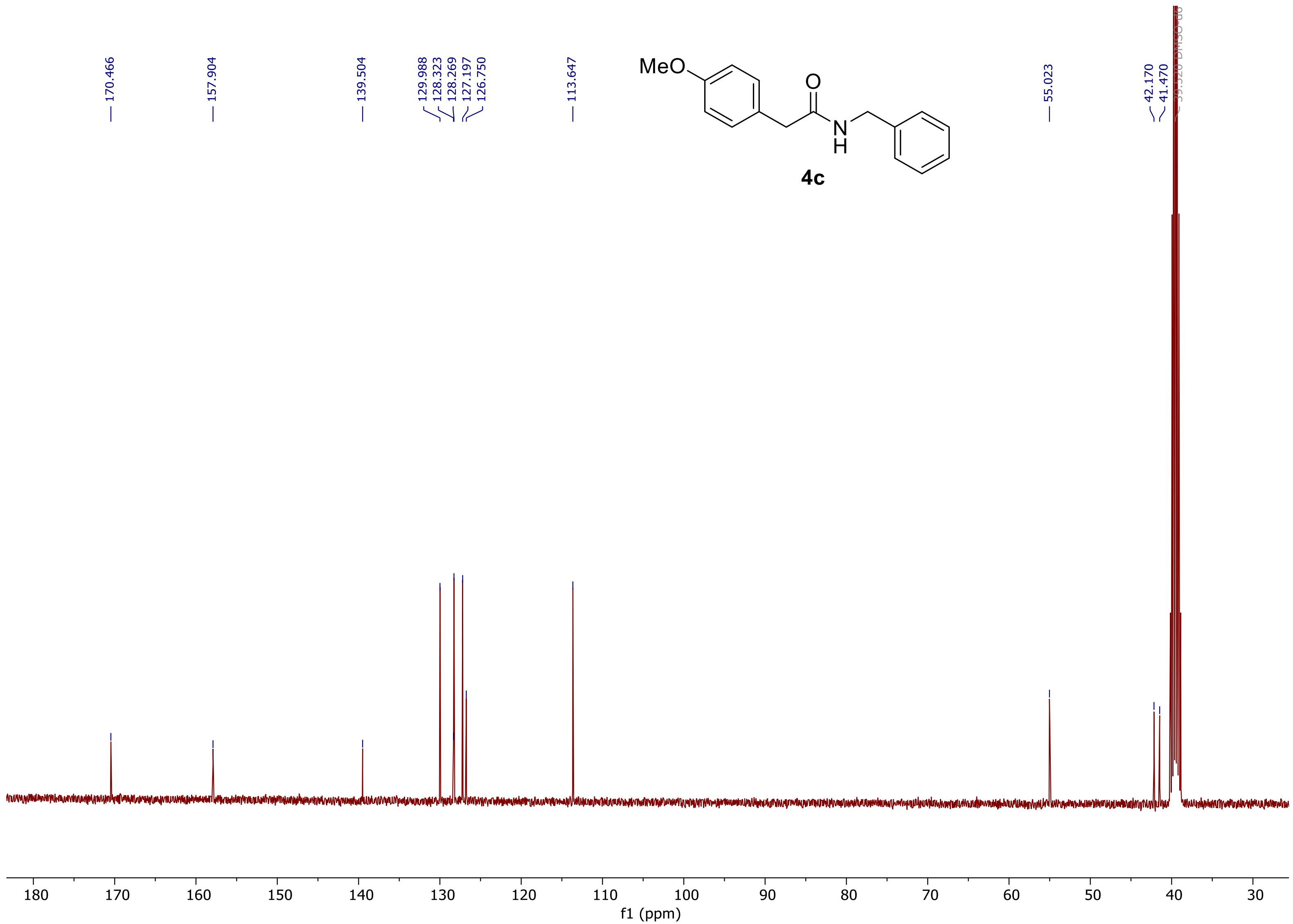

5.7 ^{1}H NMR spectrum 4d (400 MHz, DMSO-d_6)

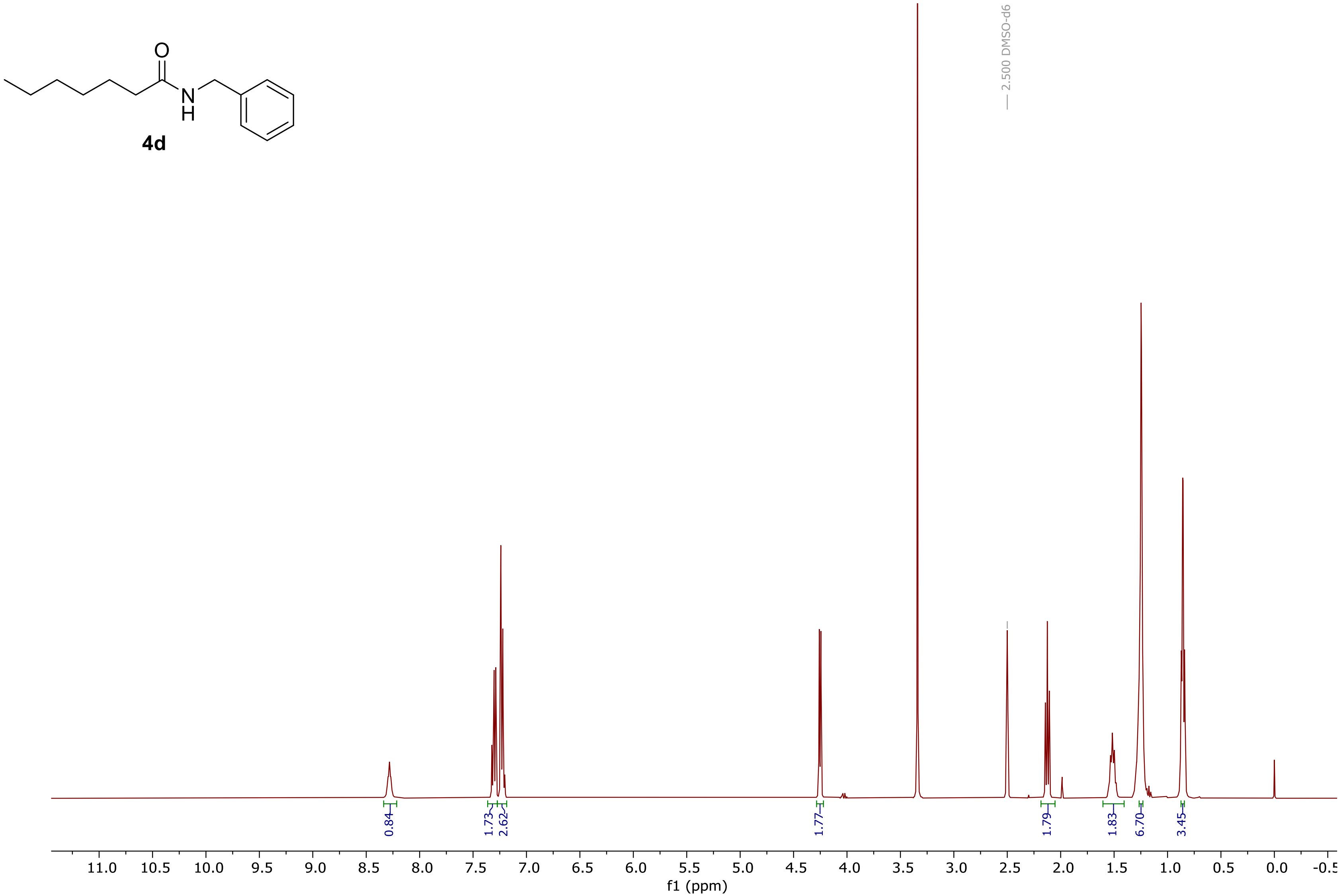

5.8 $^{13}C\{^{1}H\}$ NMR spectrum 4d (101 MHz, DMSO-d_6)

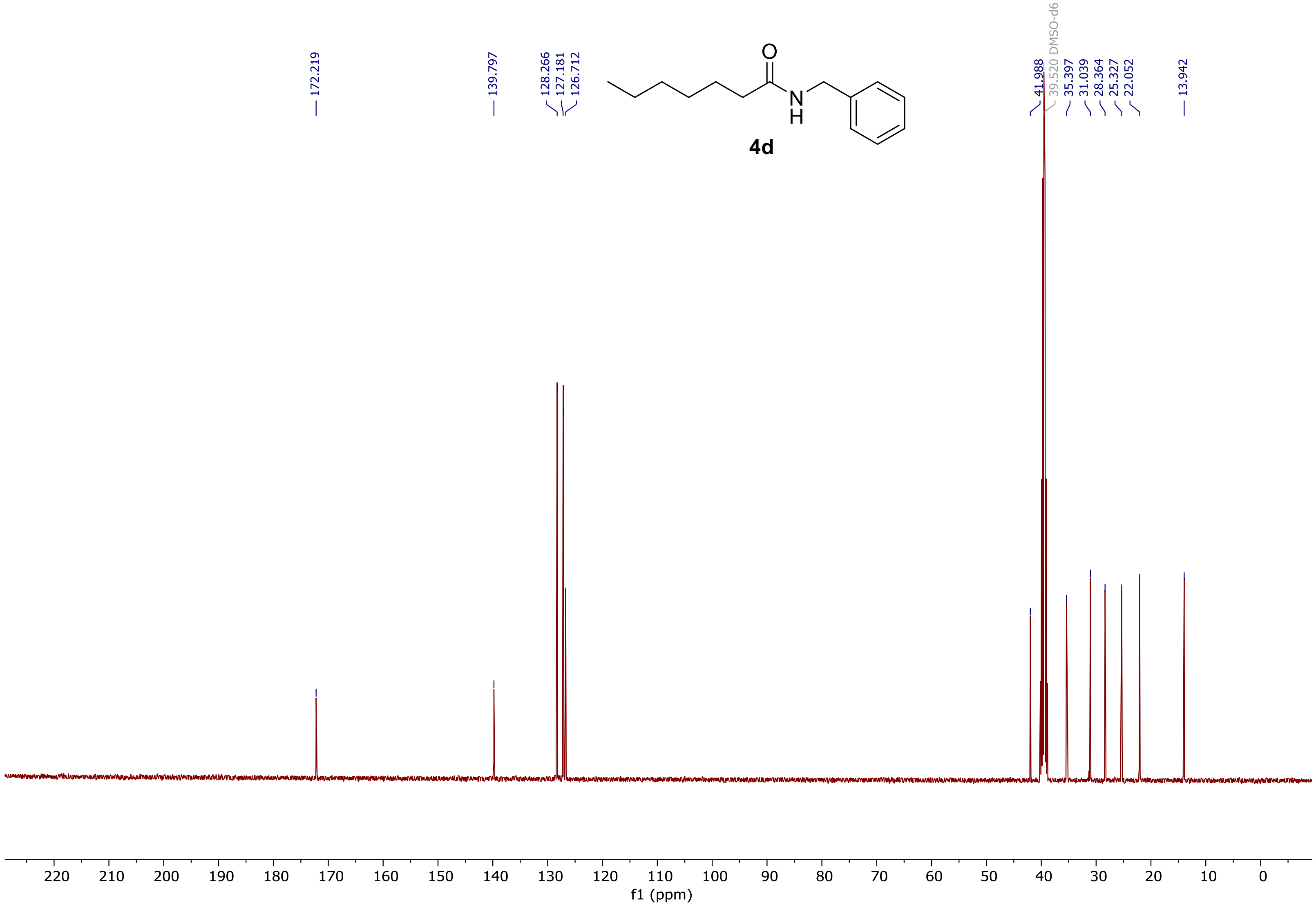

5.9 ^{1}H NMR spectrum 4e (400 MHz, DMSO-d$_6$)

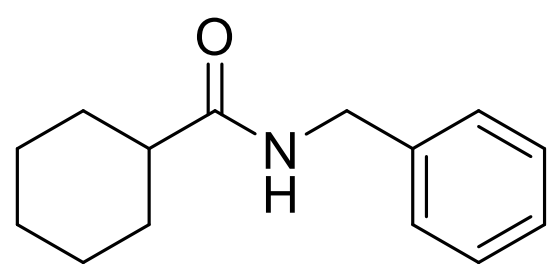

4e

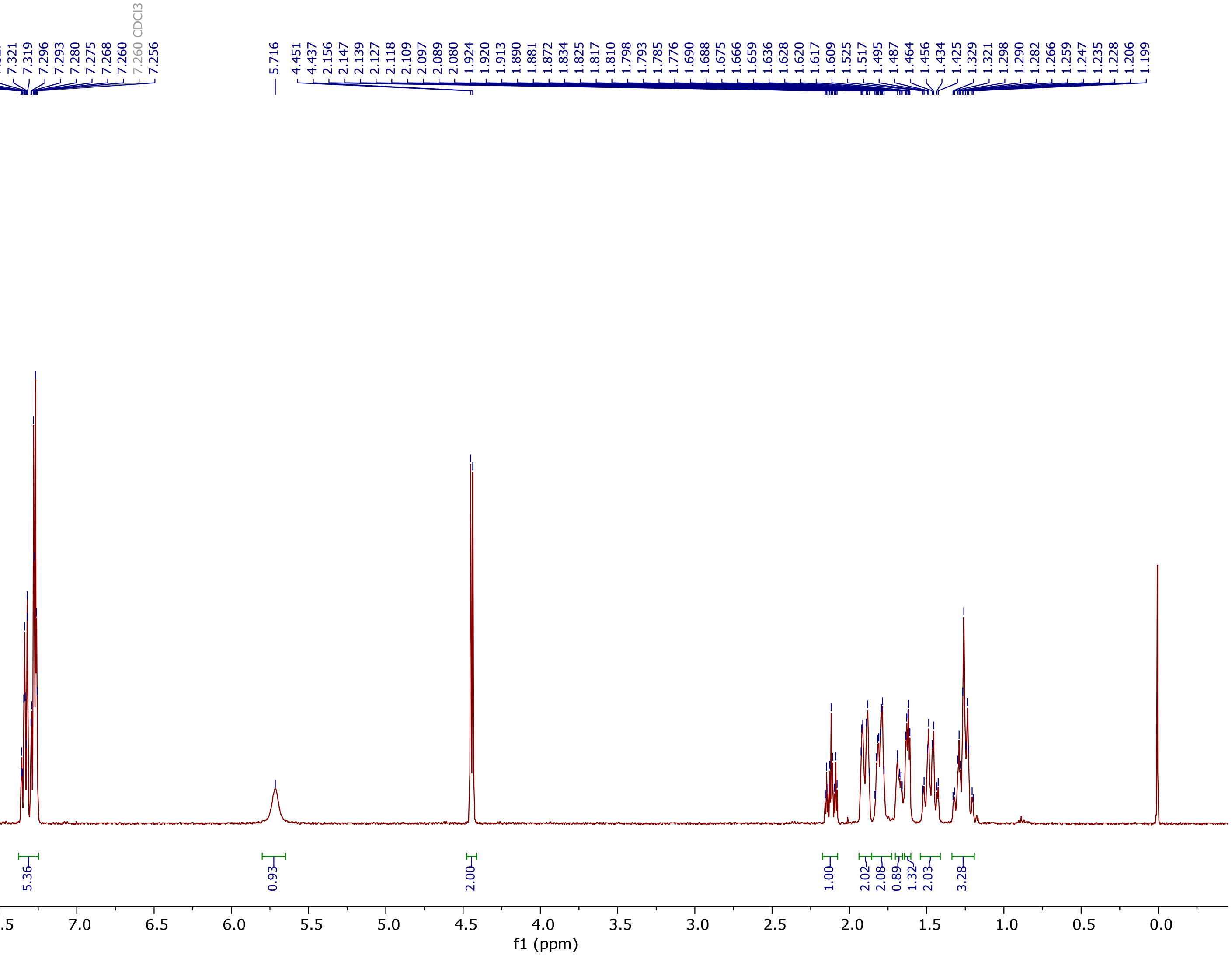

5.10 $^{13}C\{^1H\}$ NMR spectrum 4e (101 MHz, DMSO-d$_6$)

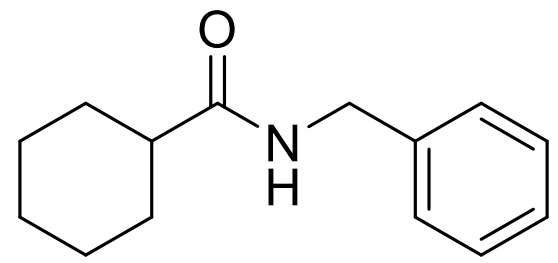

4e

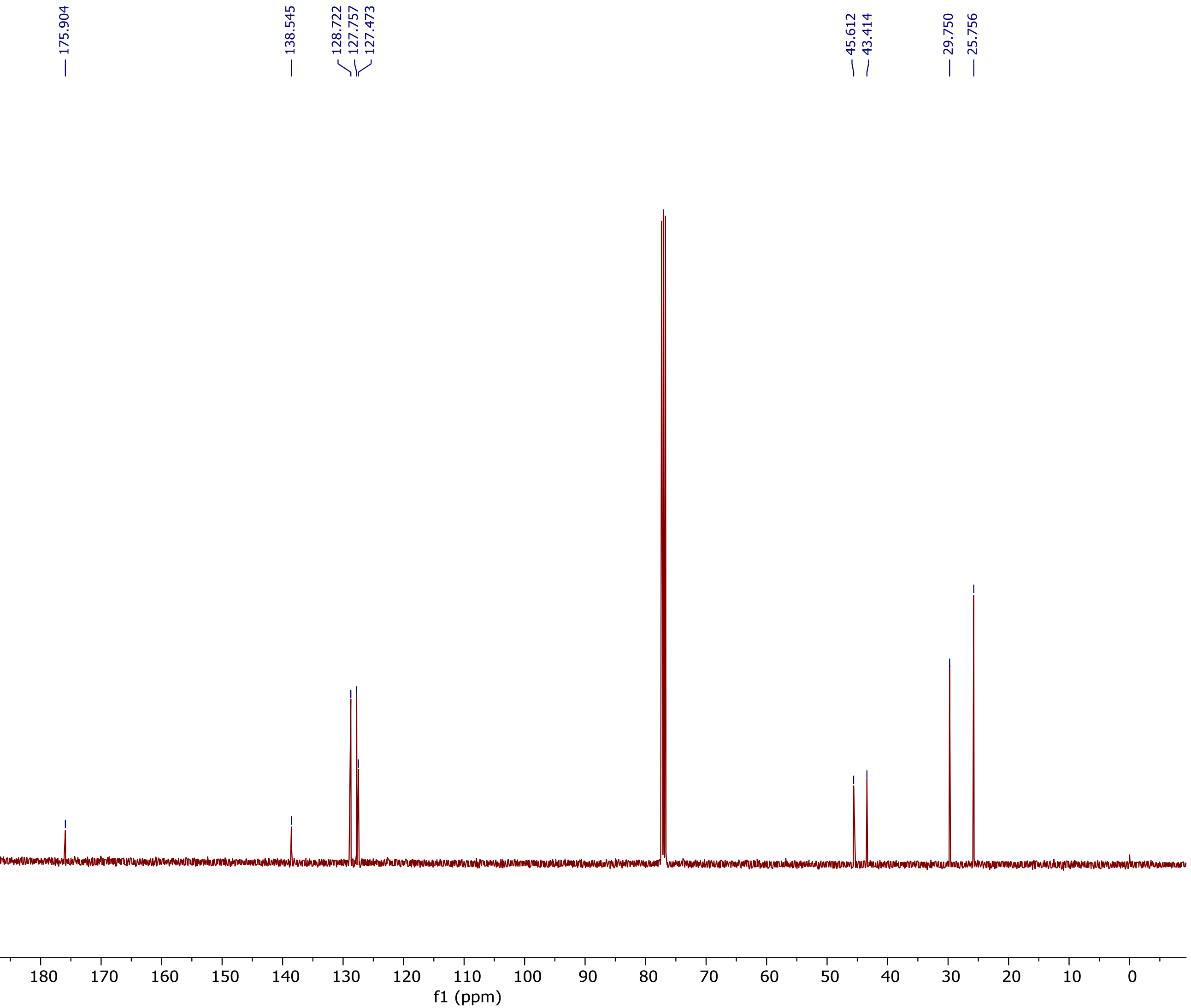

5.11 ^{1}H NMR spectrum 4f (400 MHz, DMSO-d$_6$)

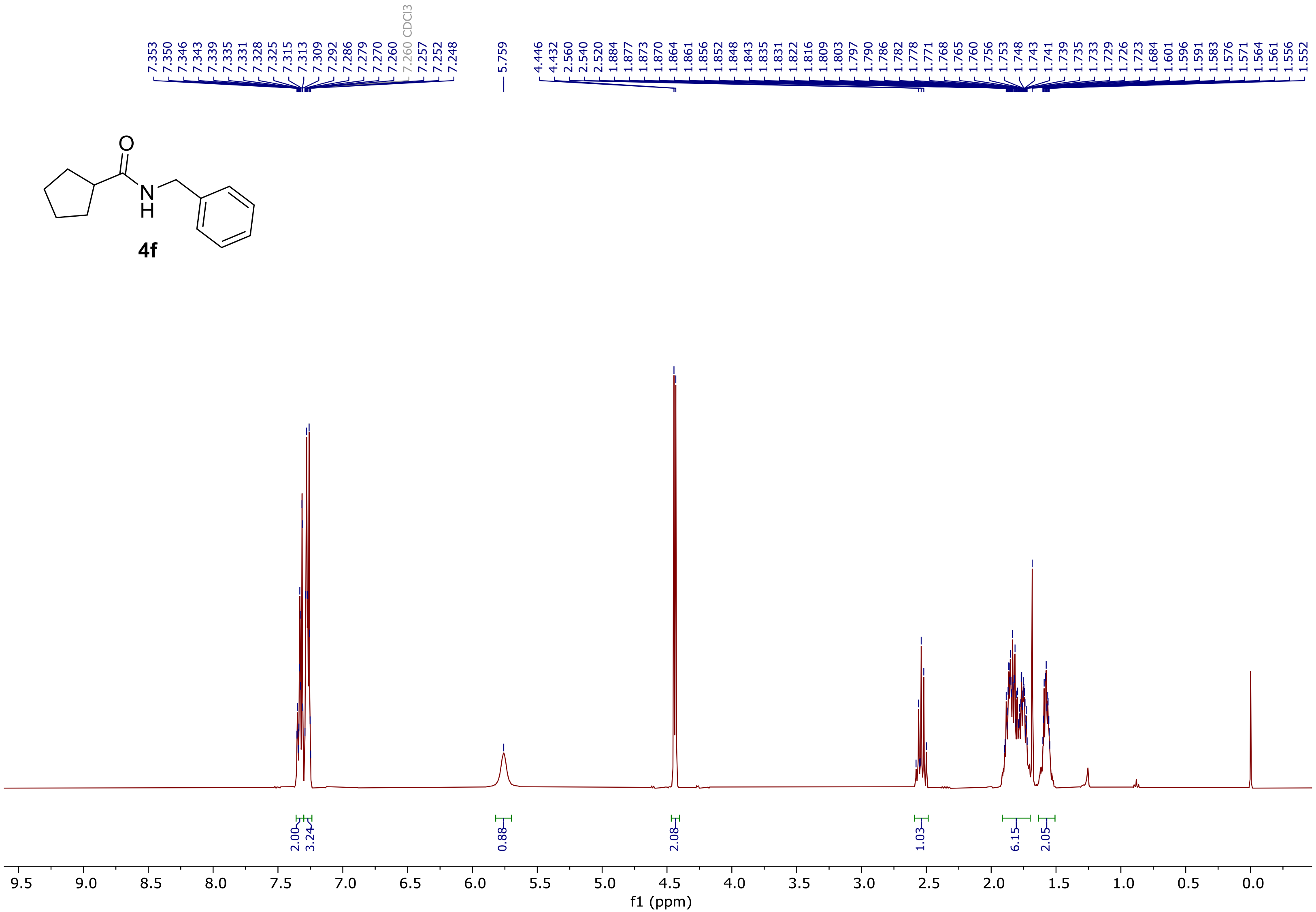

5.12 $^{13}C\{^1H\}$ NMR spectrum 4f (101 MHz, DMSO-d_6)

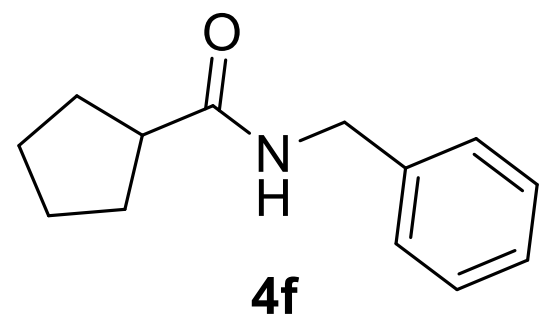

4f

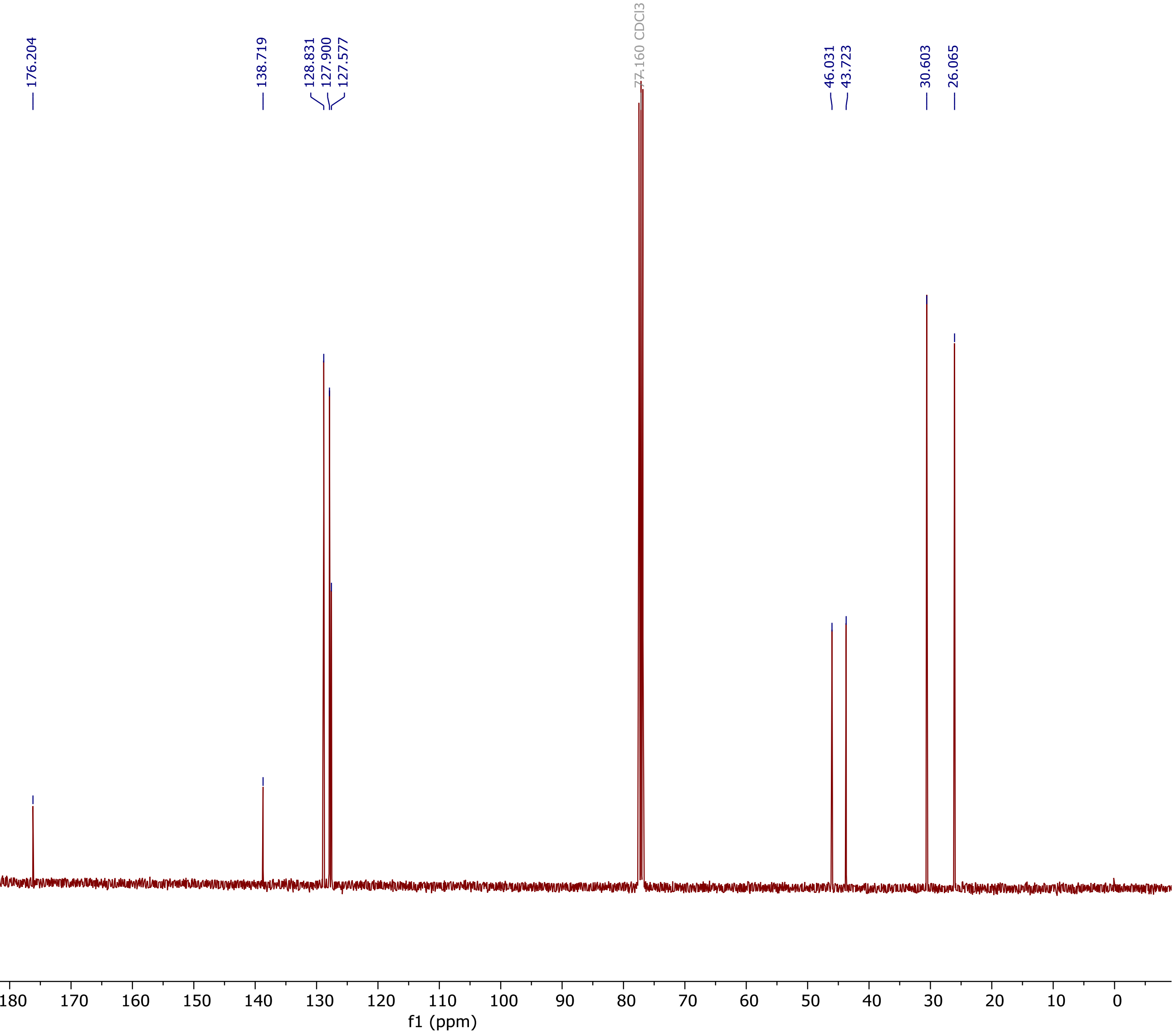

5.13 ^{1}H NMR spectrum 4g (400 MHz, $CDCl_3$)

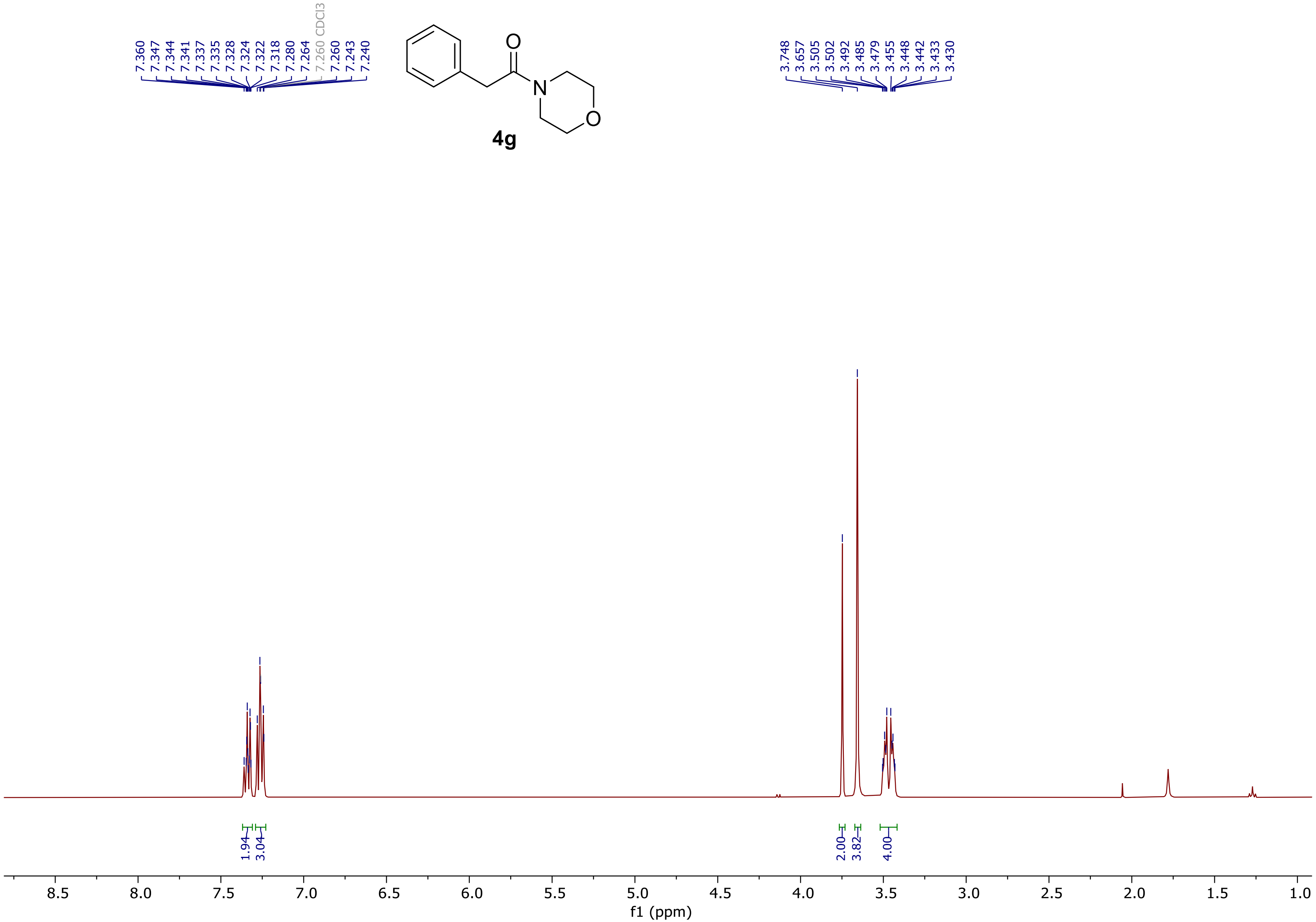

5.14 $^{13}C\{^{1}H\}$ NMR spectrum 4g (101 MHz, $CDCl_3$)

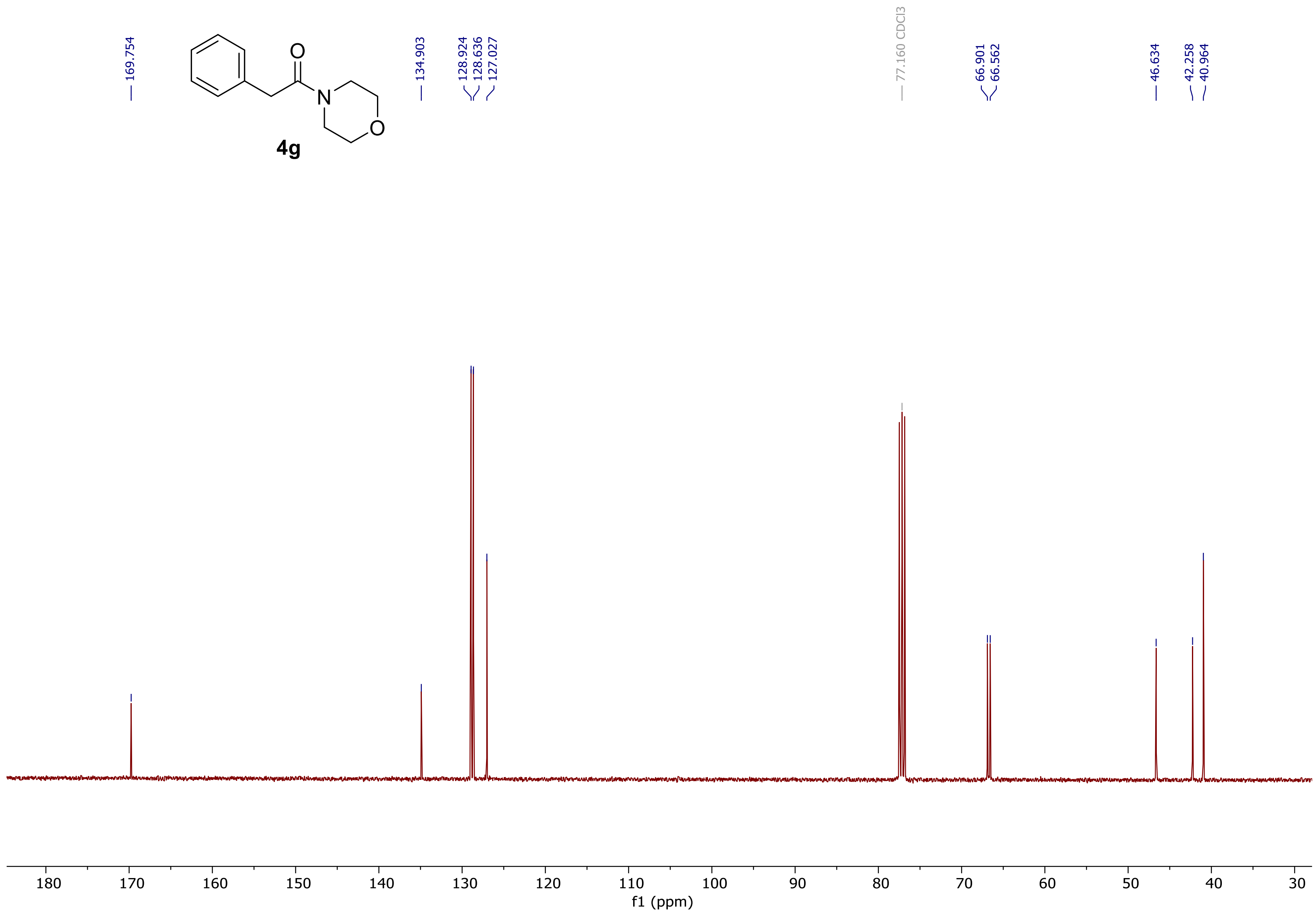

5.15 ^{1}H NMR spectrum 4h (400 MHz, $CDCl_3$)

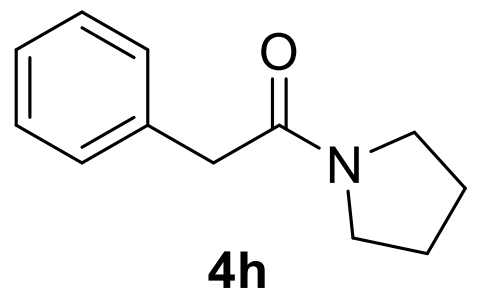

4h

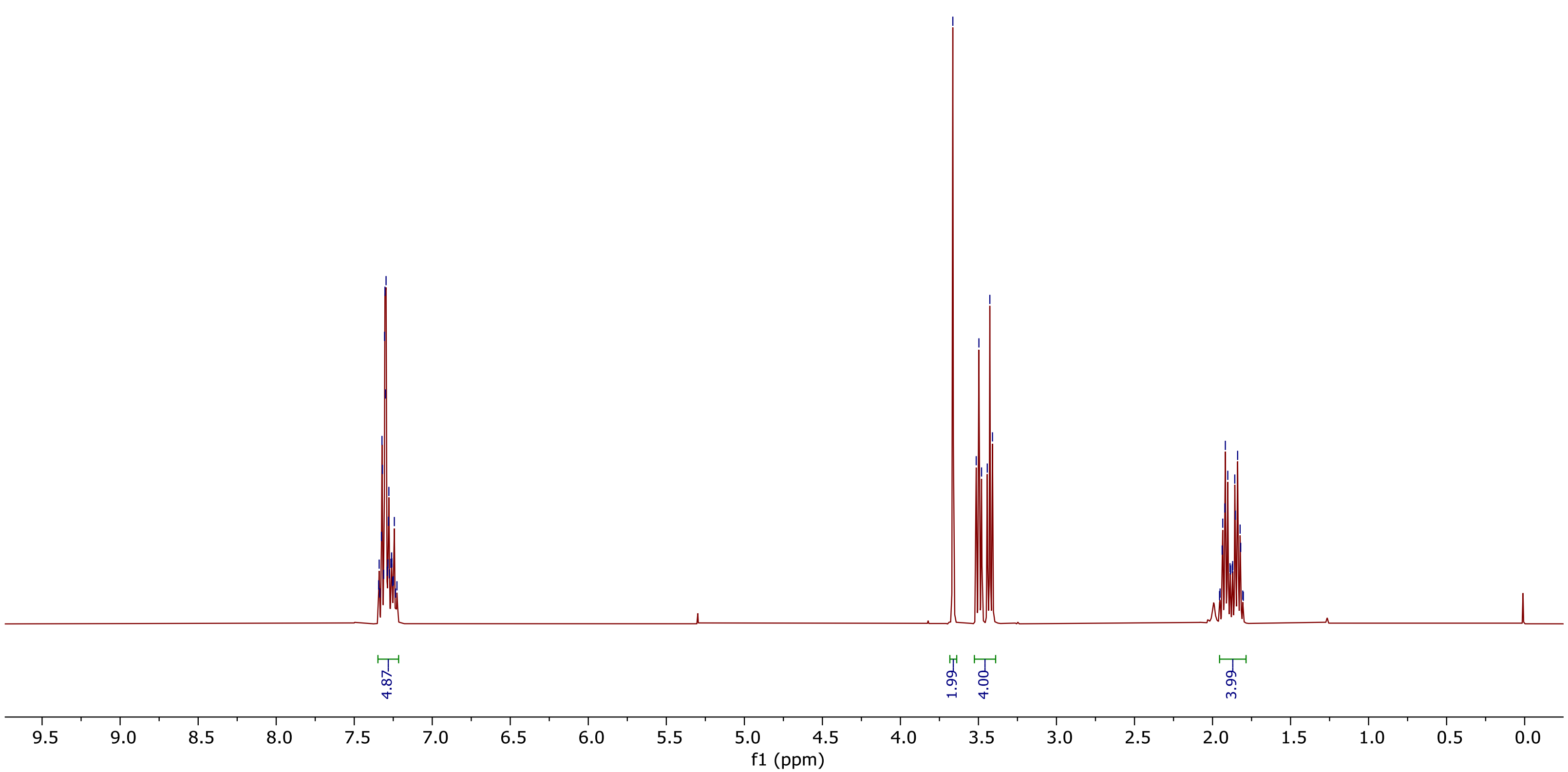

5.16 $^{13}C\{^{1}H\}$ NMR spectrum 4h (101 MHz, $CDCl_3$)

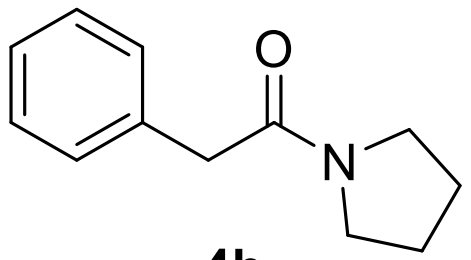

4h

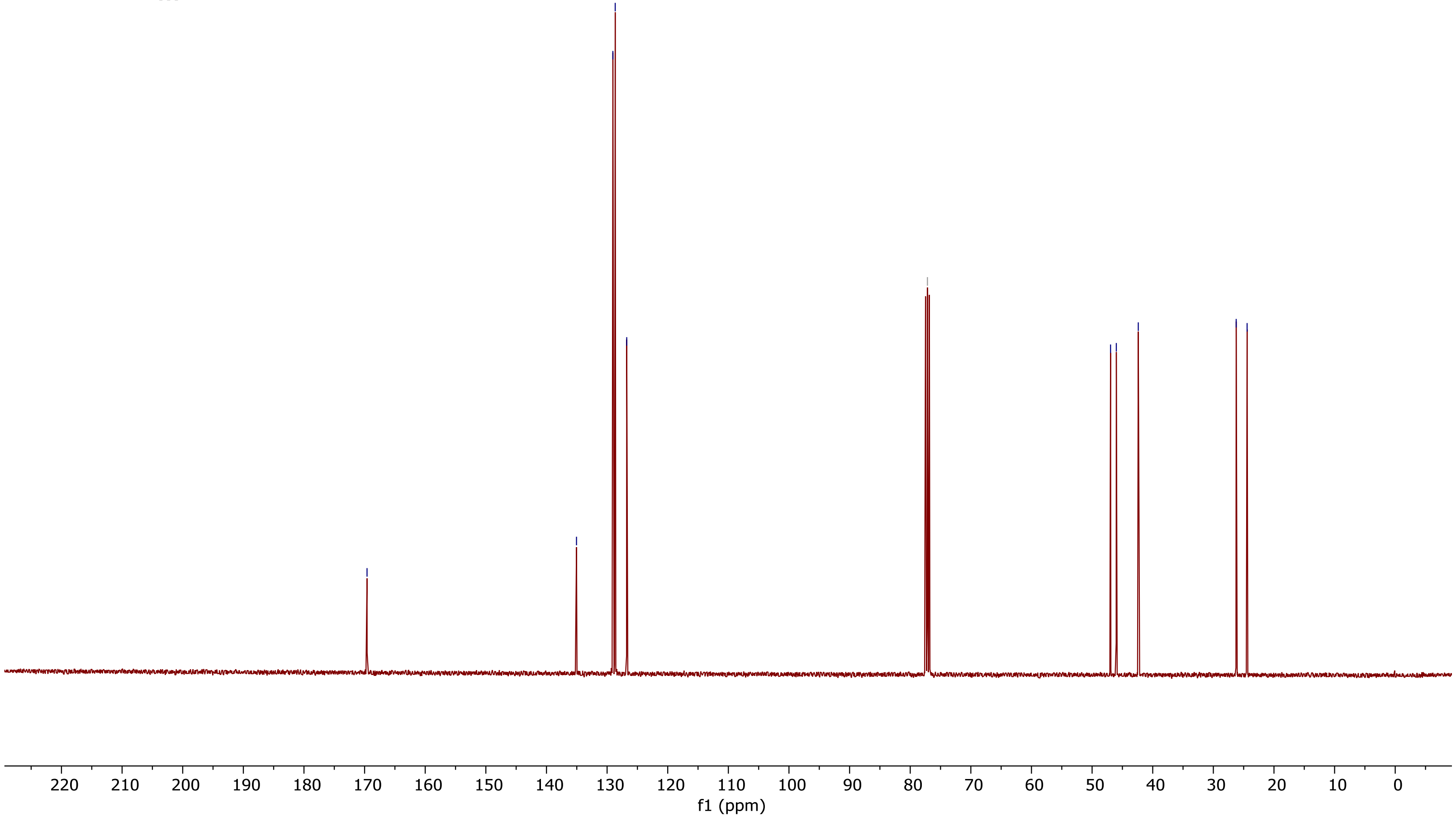

5.17 ^{1}H NMR spectrum 4i (400 MHz, $CDCl_3$)

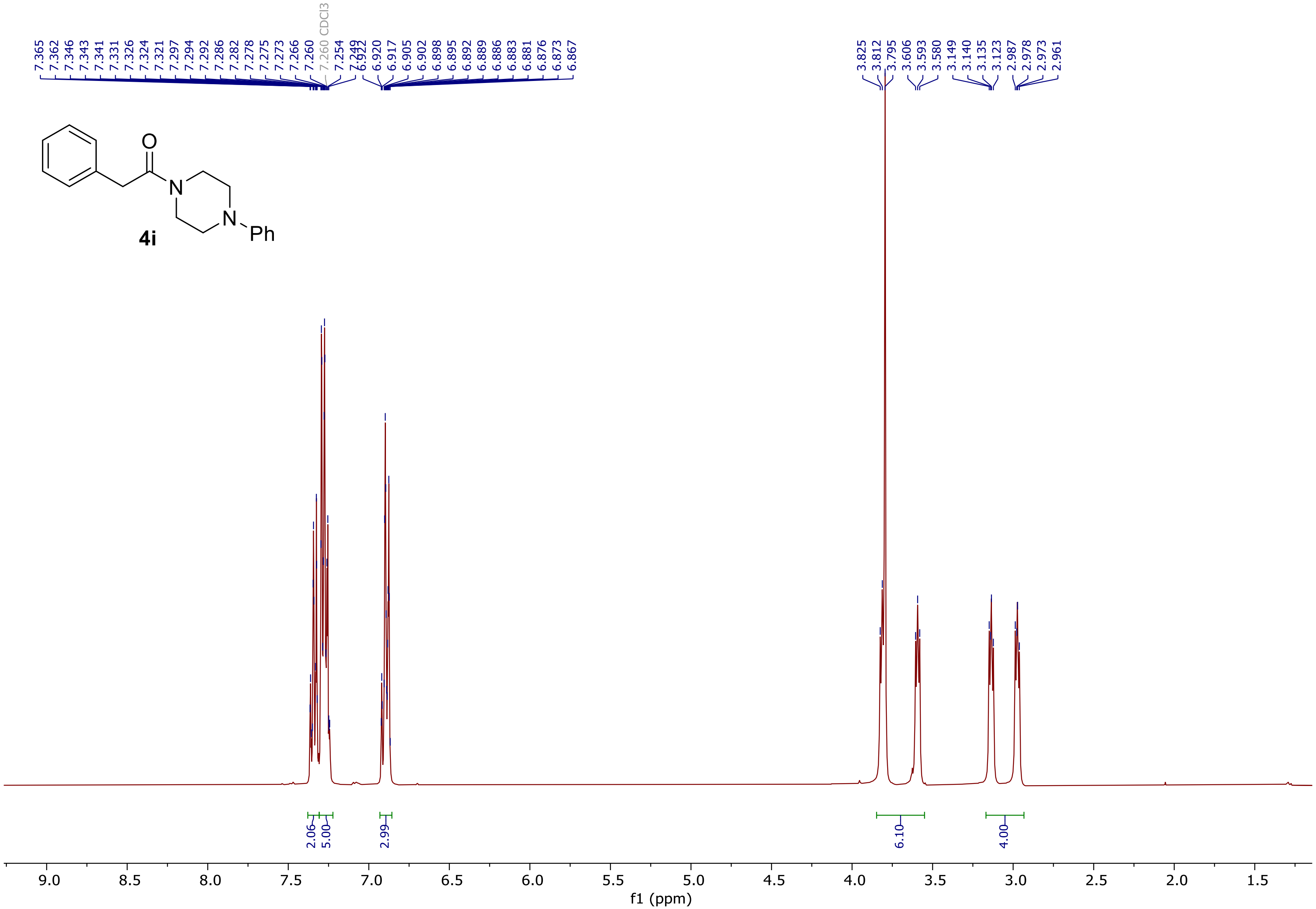

5.18 $^{13}C\{^1H\}$ NMR spectrum 4i (101 MHz, $CDCl_3$)

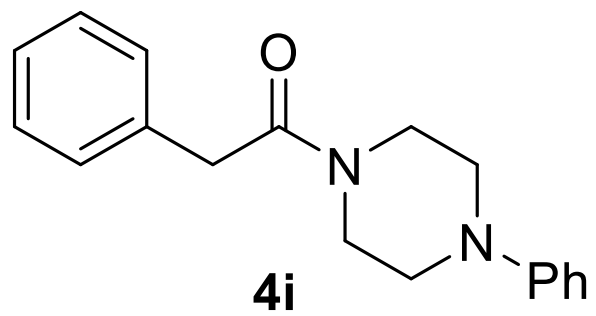
4i

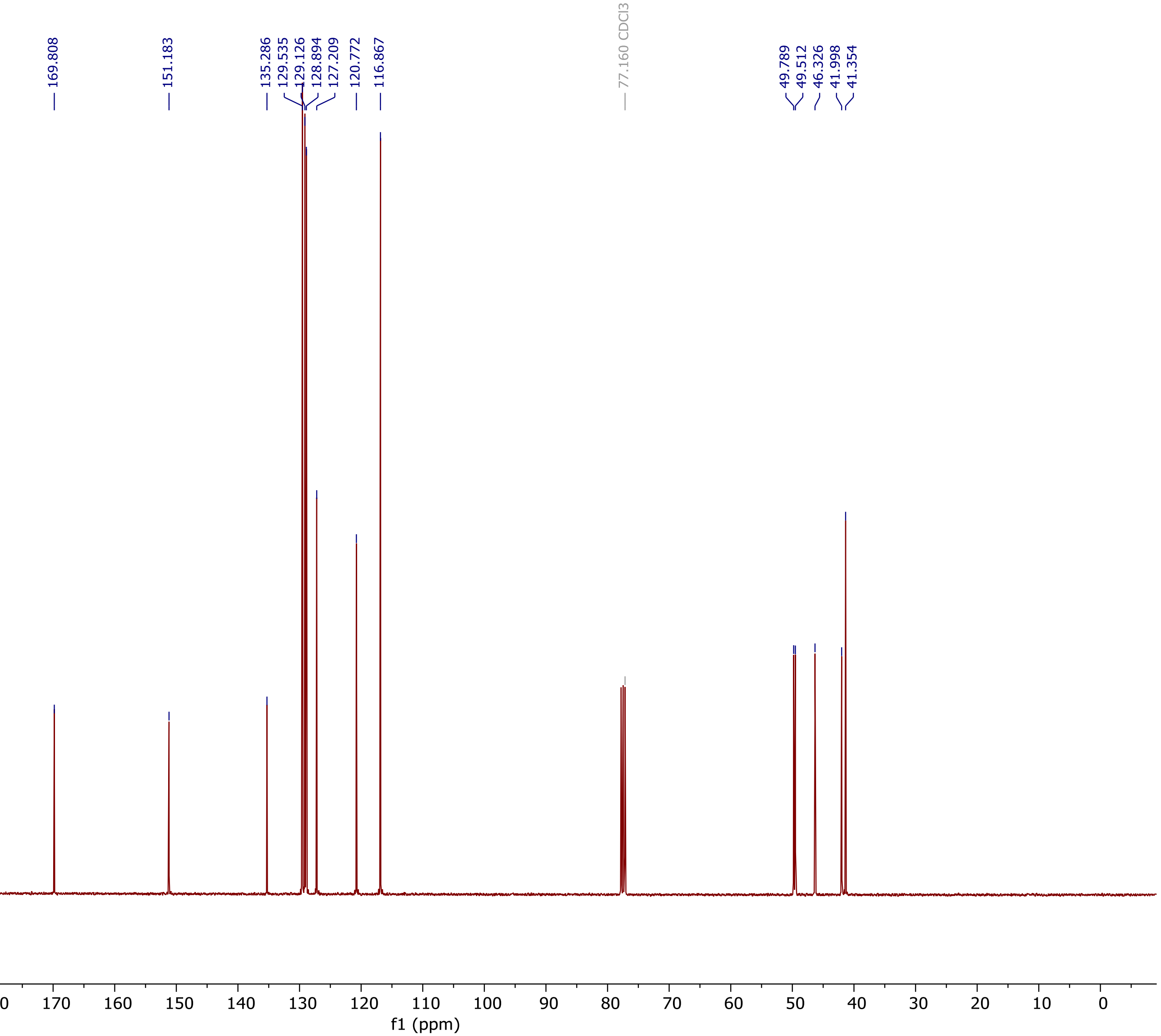

5.19 ^{1}H NMR spectrum 4j (600 MHz, DMSO-d$_6$)

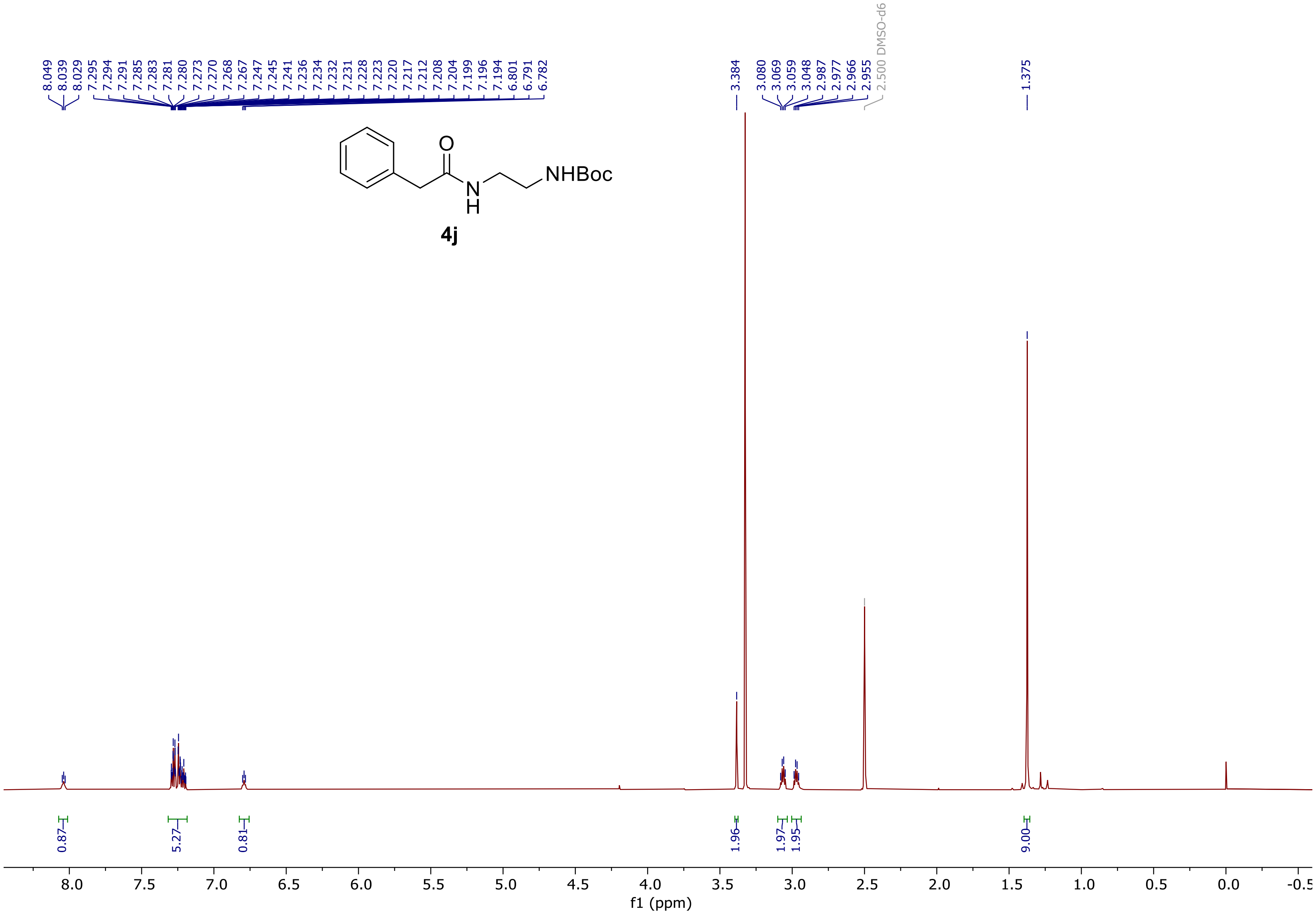

5.20 $^{13}C\{^{1}H\}$ NMR spectrum 4j (151 MHz, DMSO-d_6)

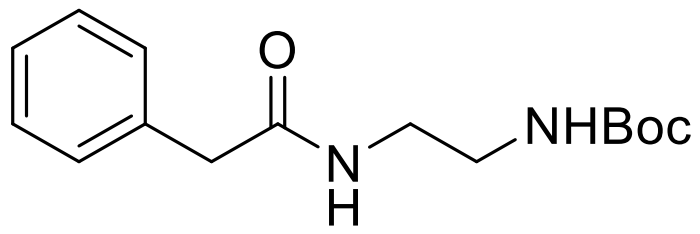

4j

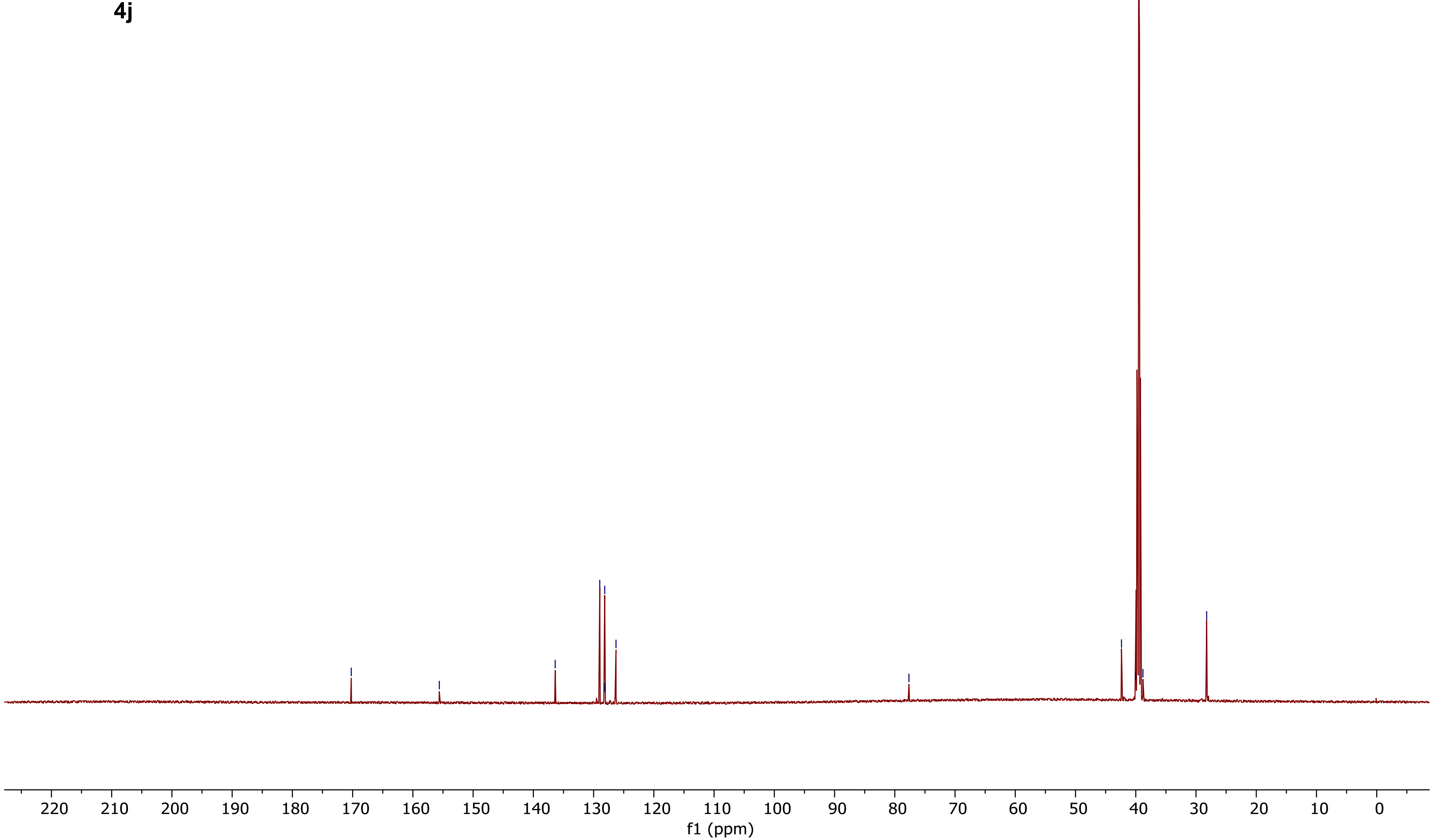

5.21 ^{1}H NMR spectrum 4k (400 MHz, $CDCl_3$)

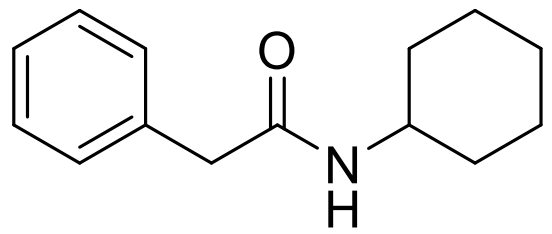

4k

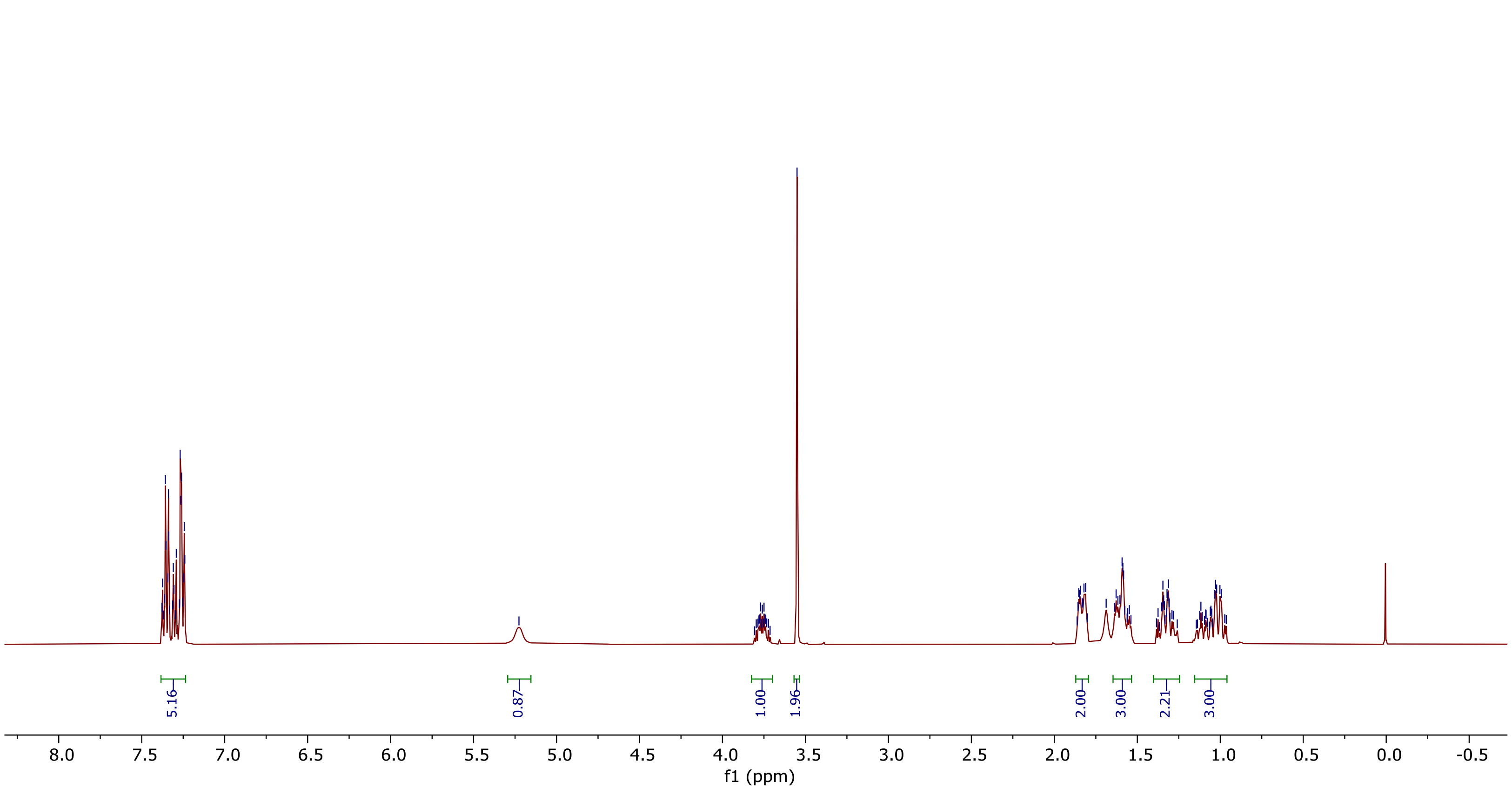

5.22 $^{13}C\{^1H\}$ NMR spectrum 4k (101 MHz, $CDCl_3$)

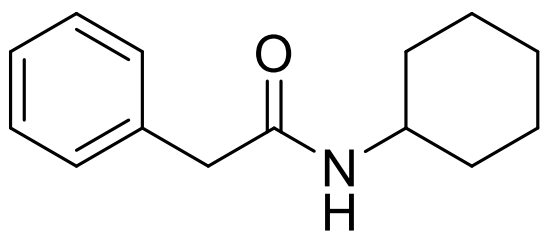

4k

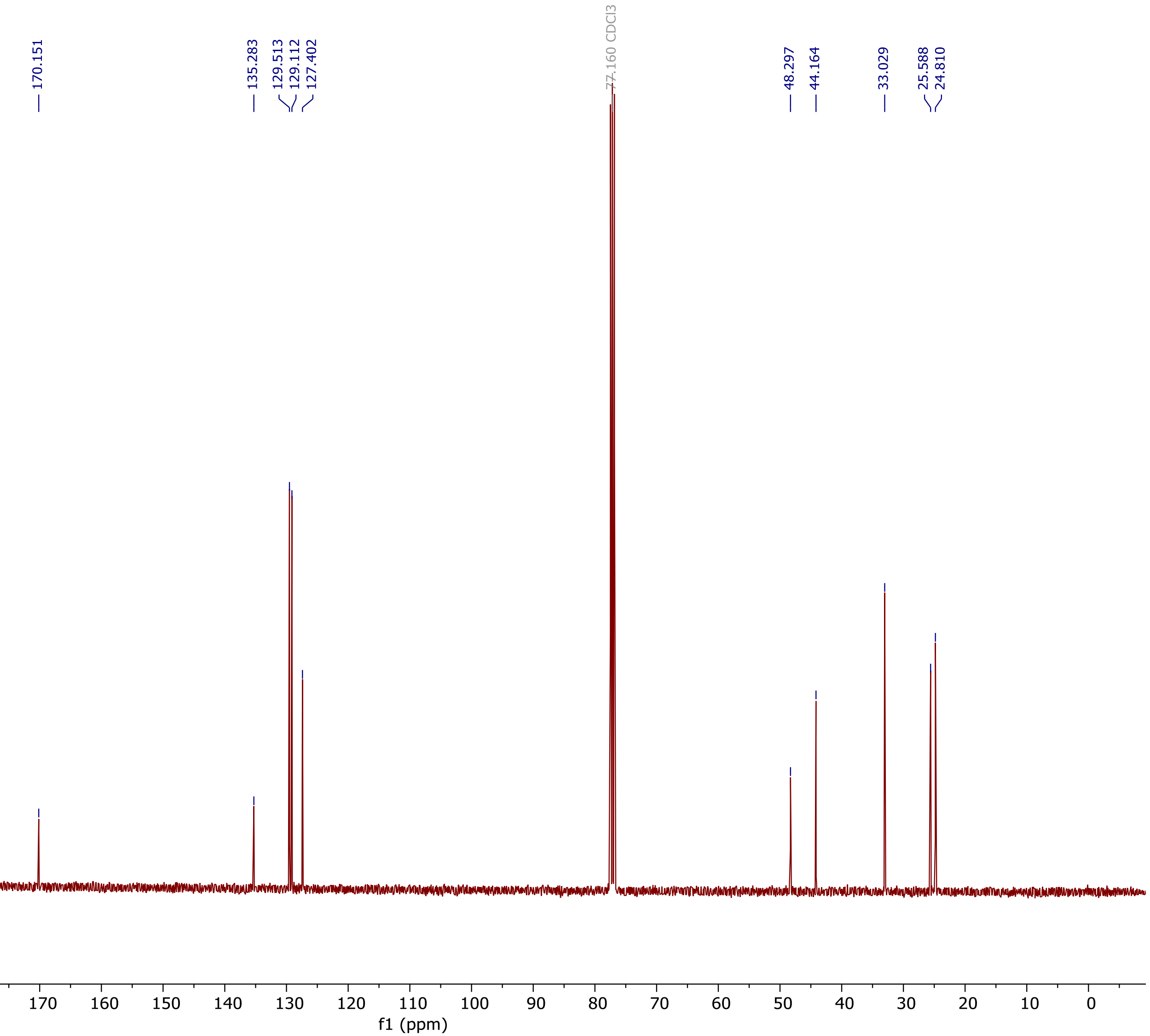

5.23 ^{1}H NMR spectrum 4l (400 MHz, $CDCl_3$)

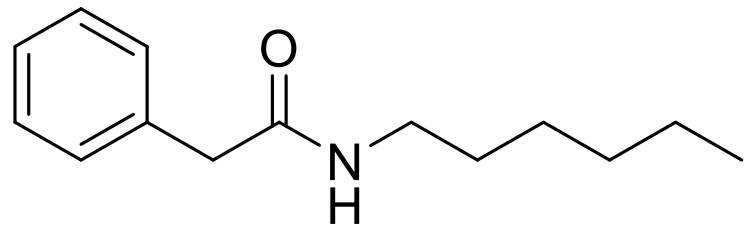

4l

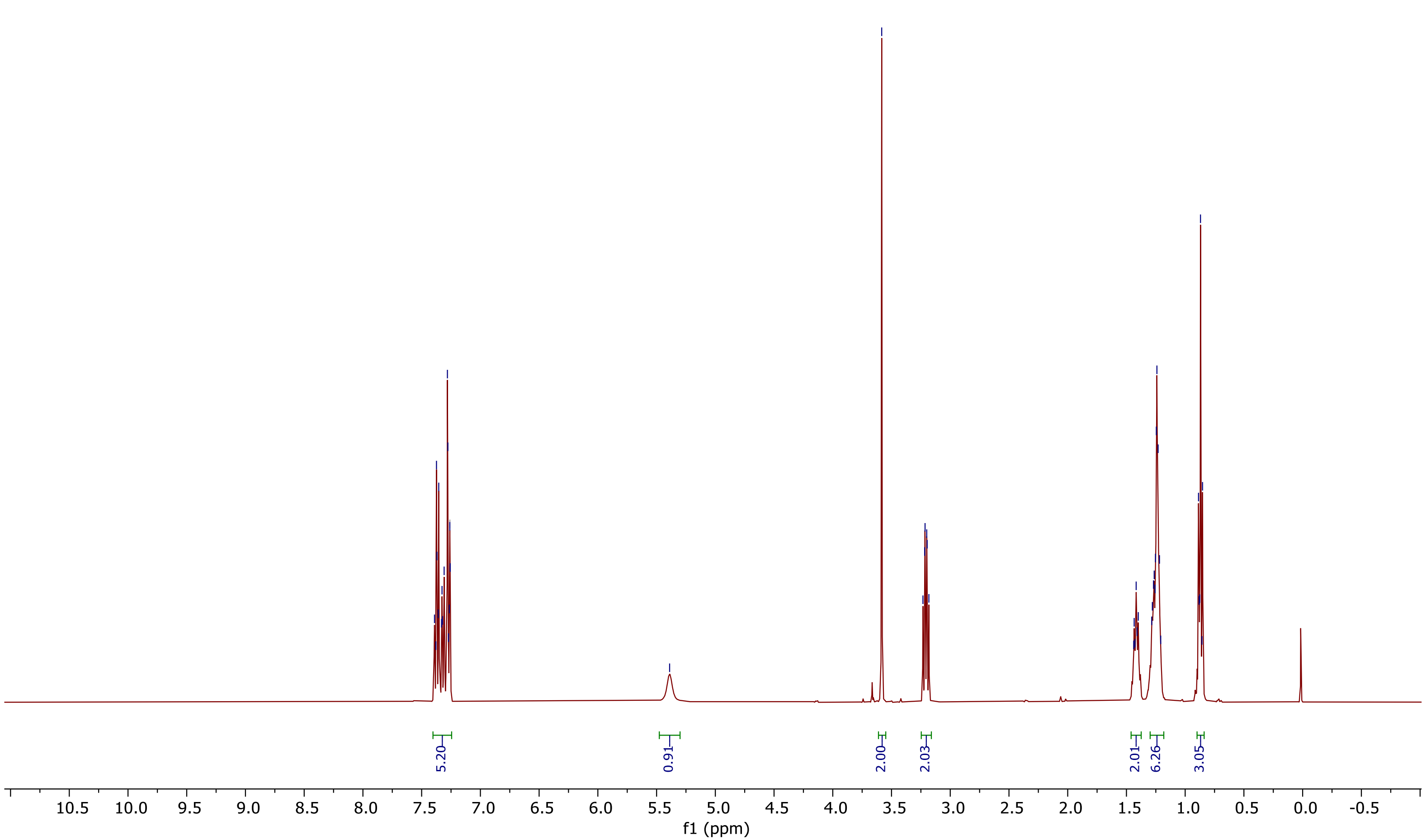

5.24 $^{13}C\{^{1}H\}$ NMR spectrum 4l (101 MHz, $CDCl_3$)

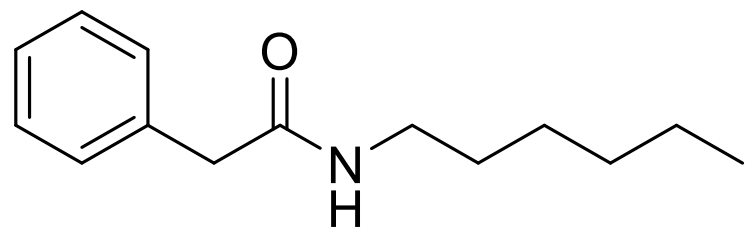

4l

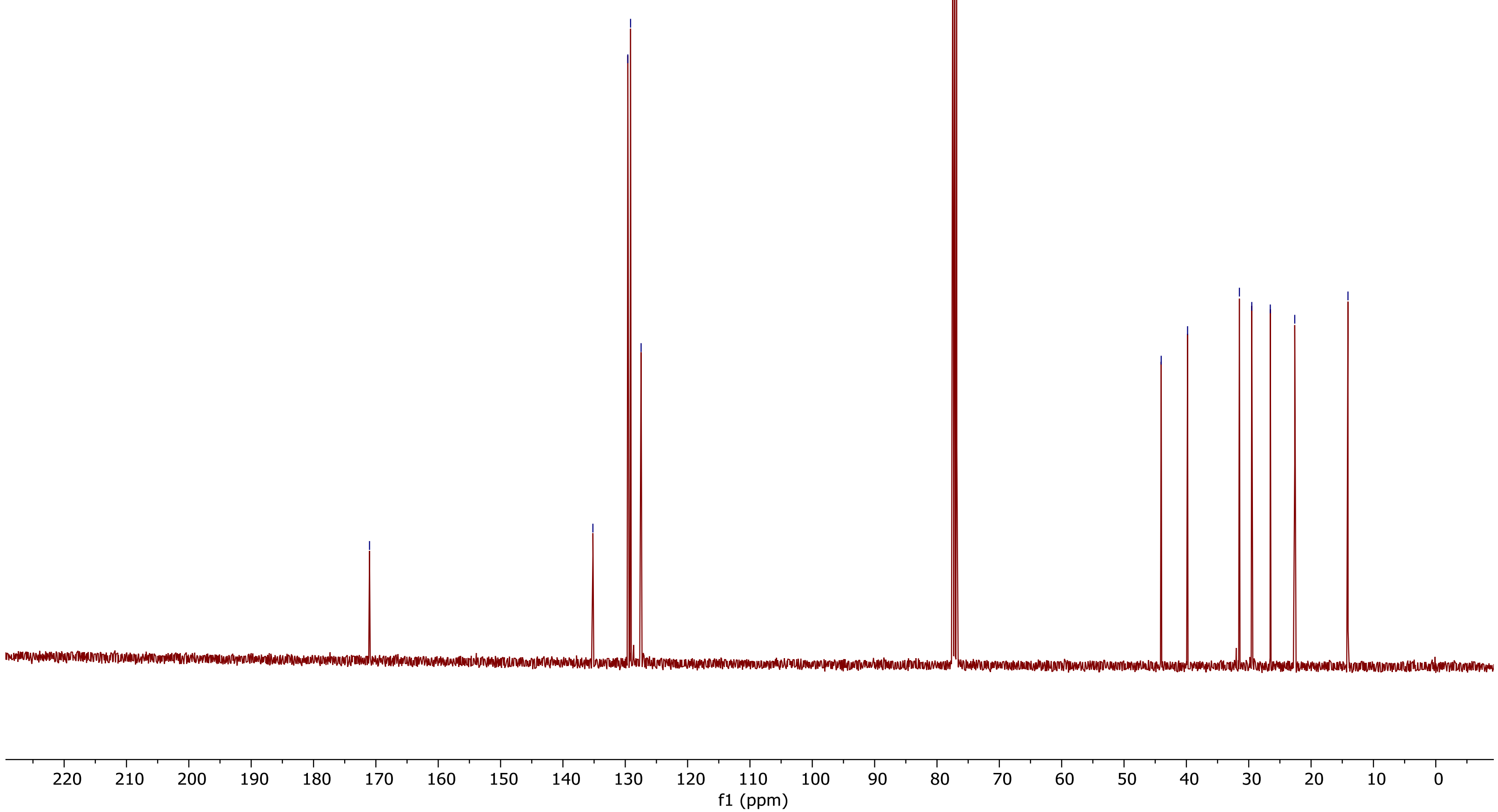

5.25 ¹H NMR spectrum 4m (400 MHz, $CDCl_3$)

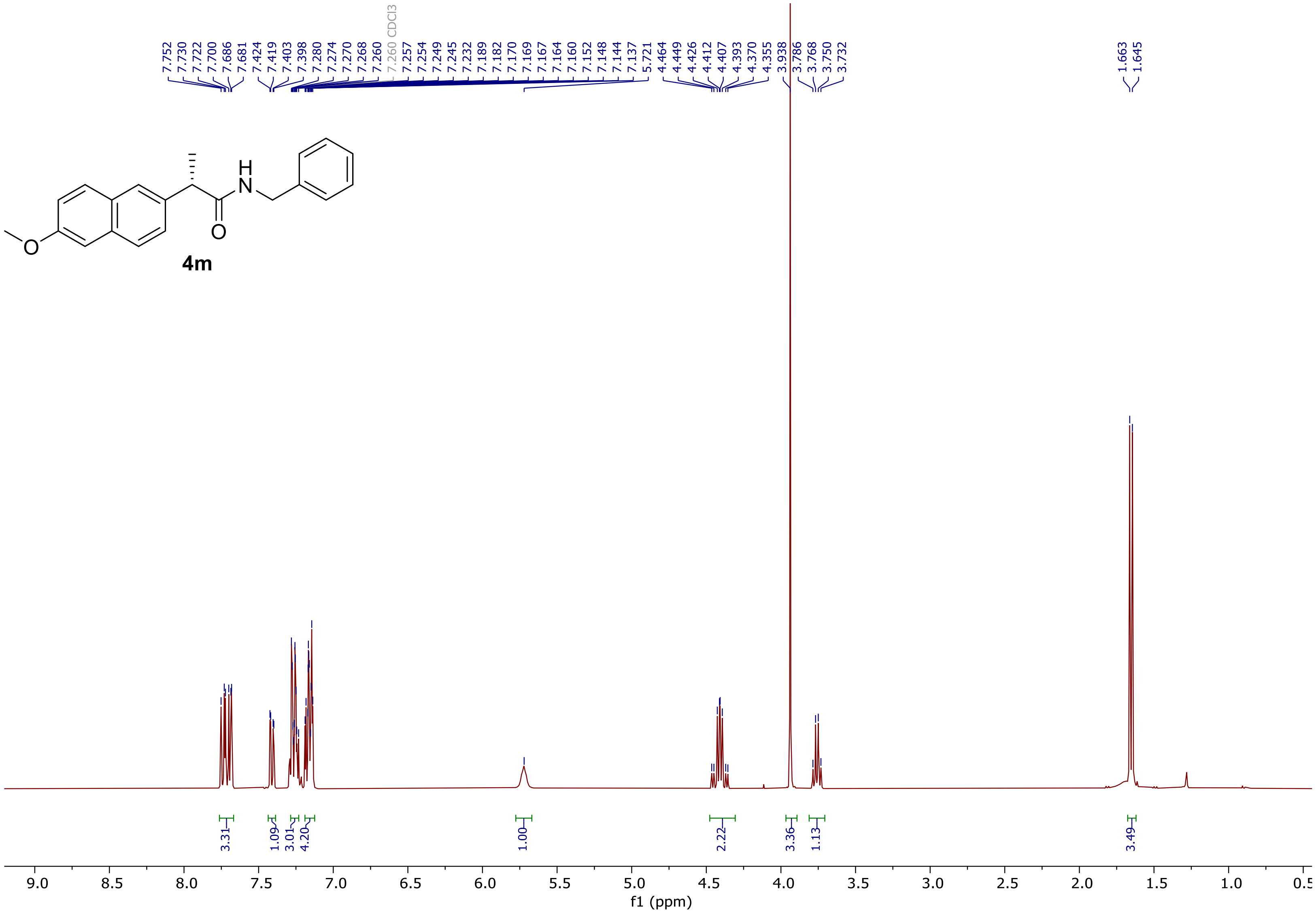

5.26 $^{13}C\{^1H\}$ NMR spectrum 4m (101 MHz, $CDCl_3$)

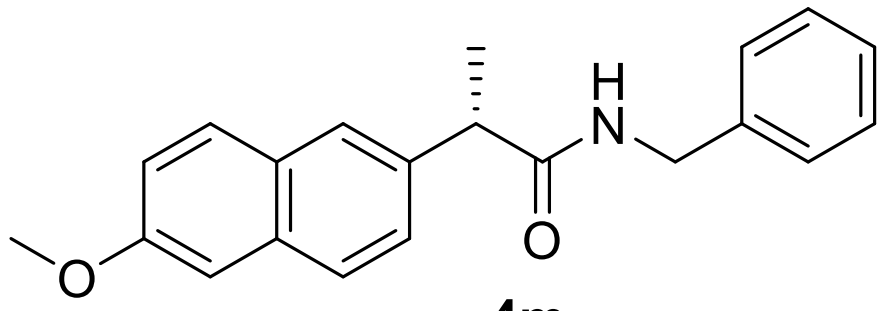

4m

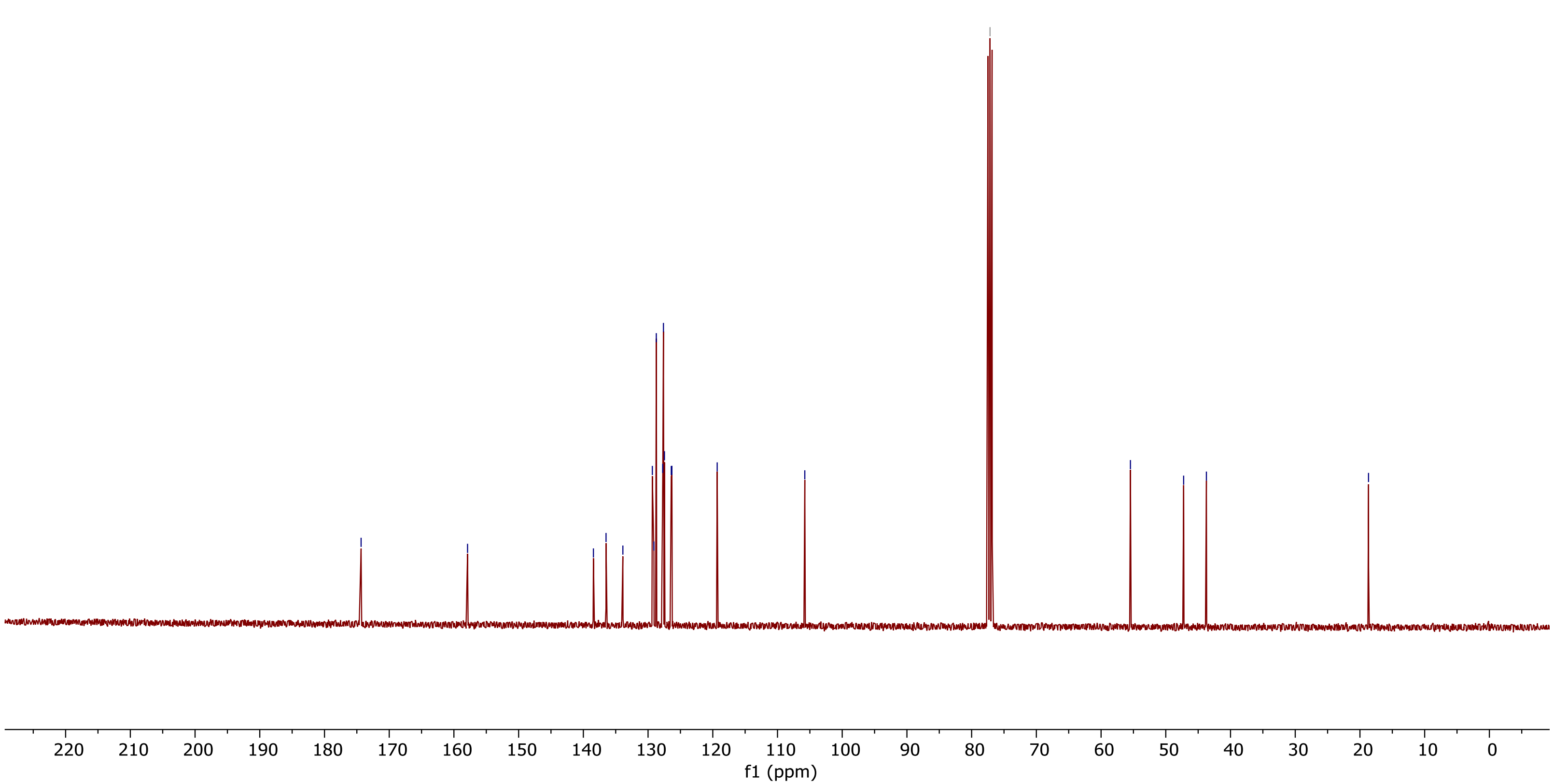

5.27 ¹H NMR spectrum 4n (400 MHz, $CDCl_3$)

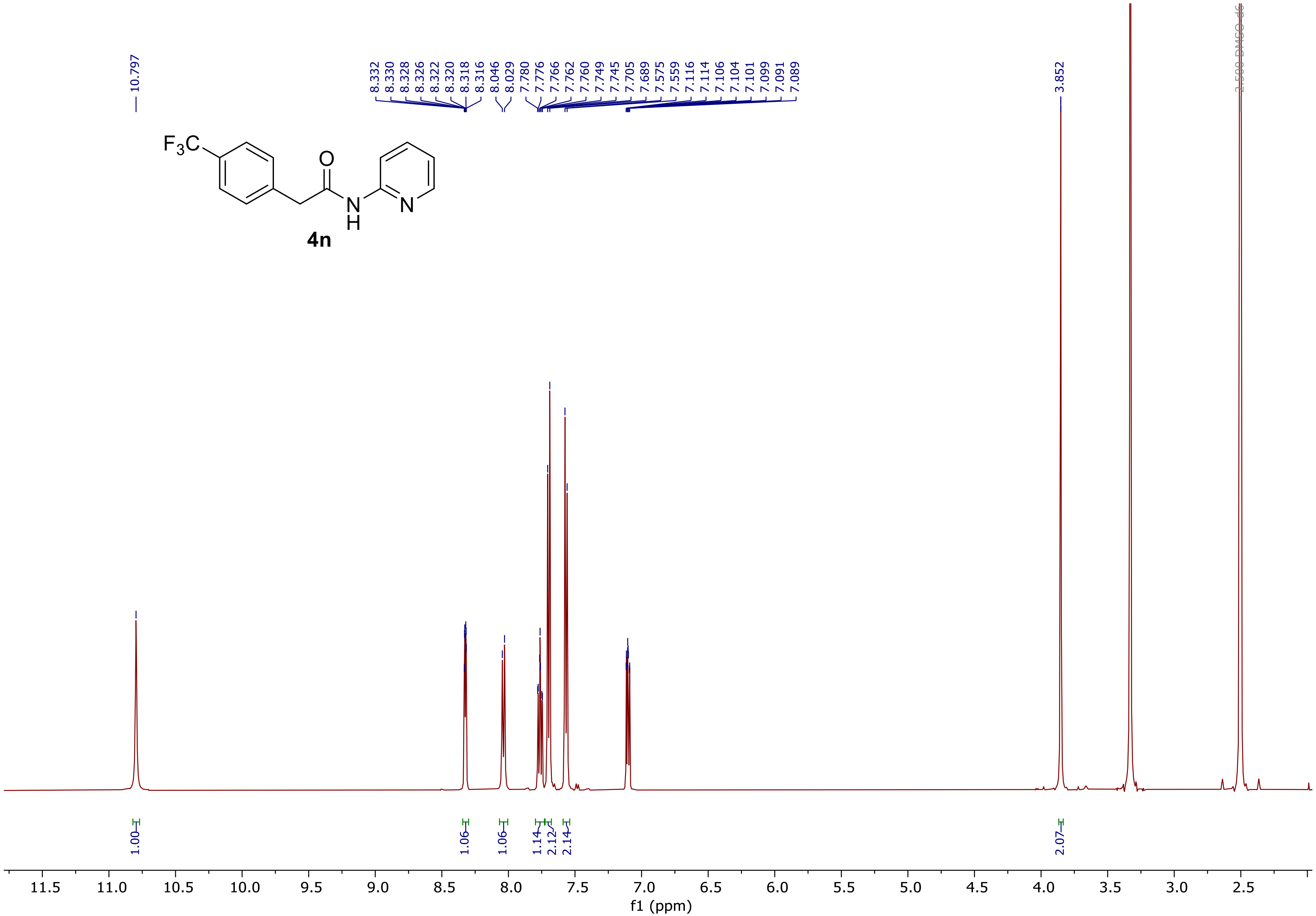

5.28 $^{13}C\{^{1}H\}$ NMR spectrum 4n (101 MHz, $CDCl_3$)

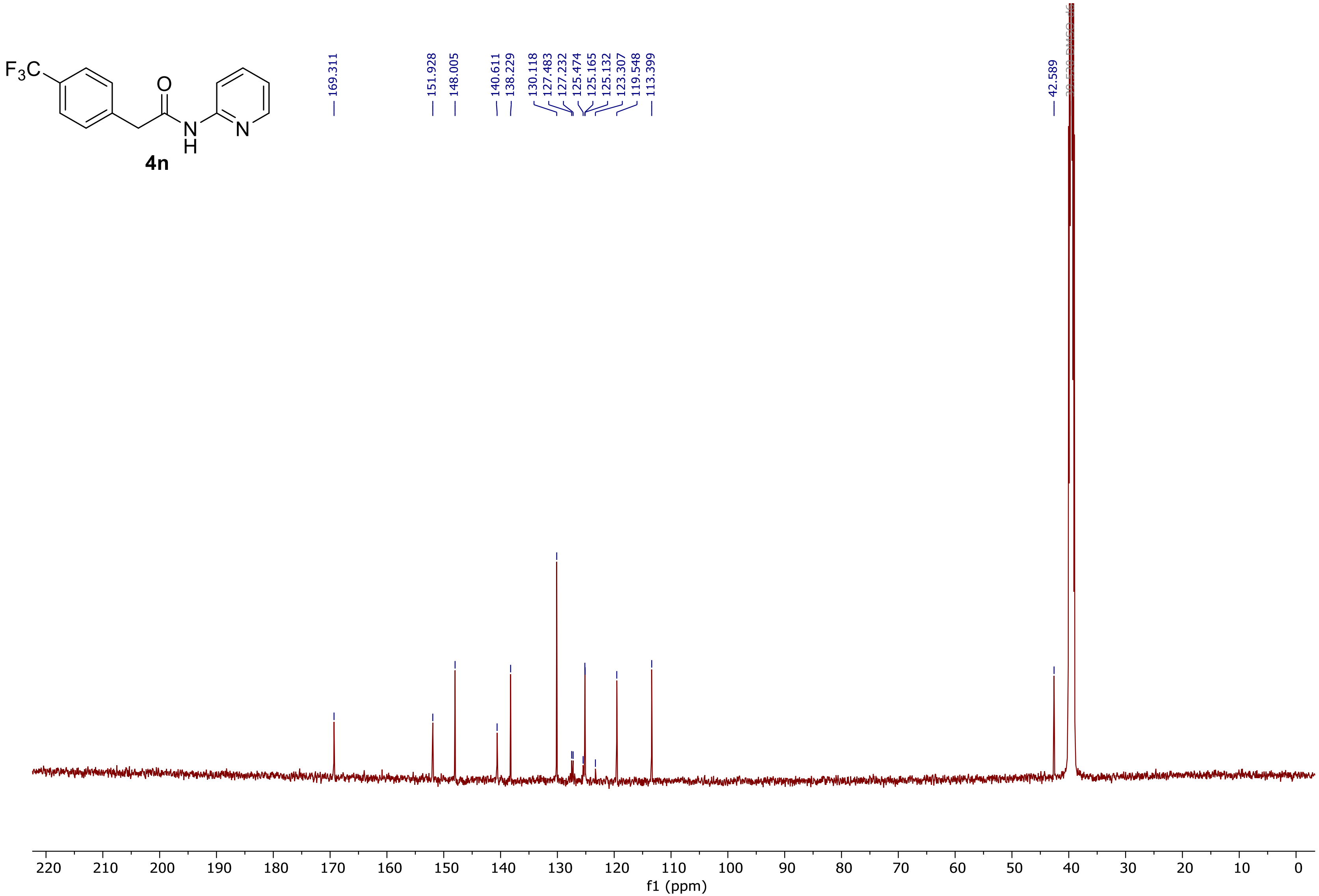

5.29 ^{1}H NMR spectrum 4o (400 MHz, DMSO-d_6)

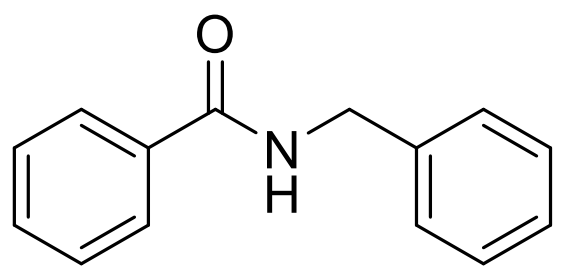

4o

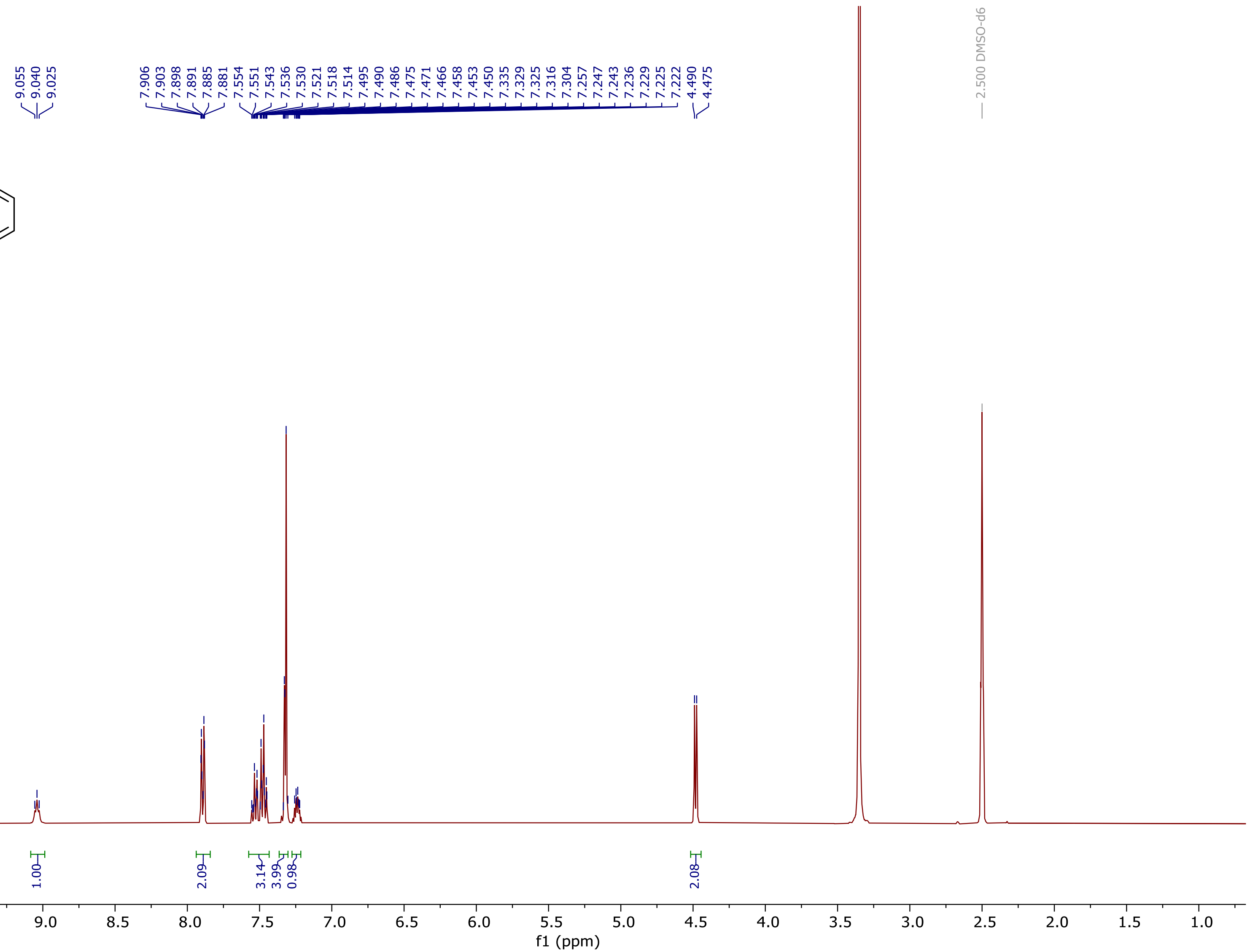

5.30 $^{13}C\{^{1}H\}$ NMR spectrum 4o (101 MHz, DMSO-d$_6$)

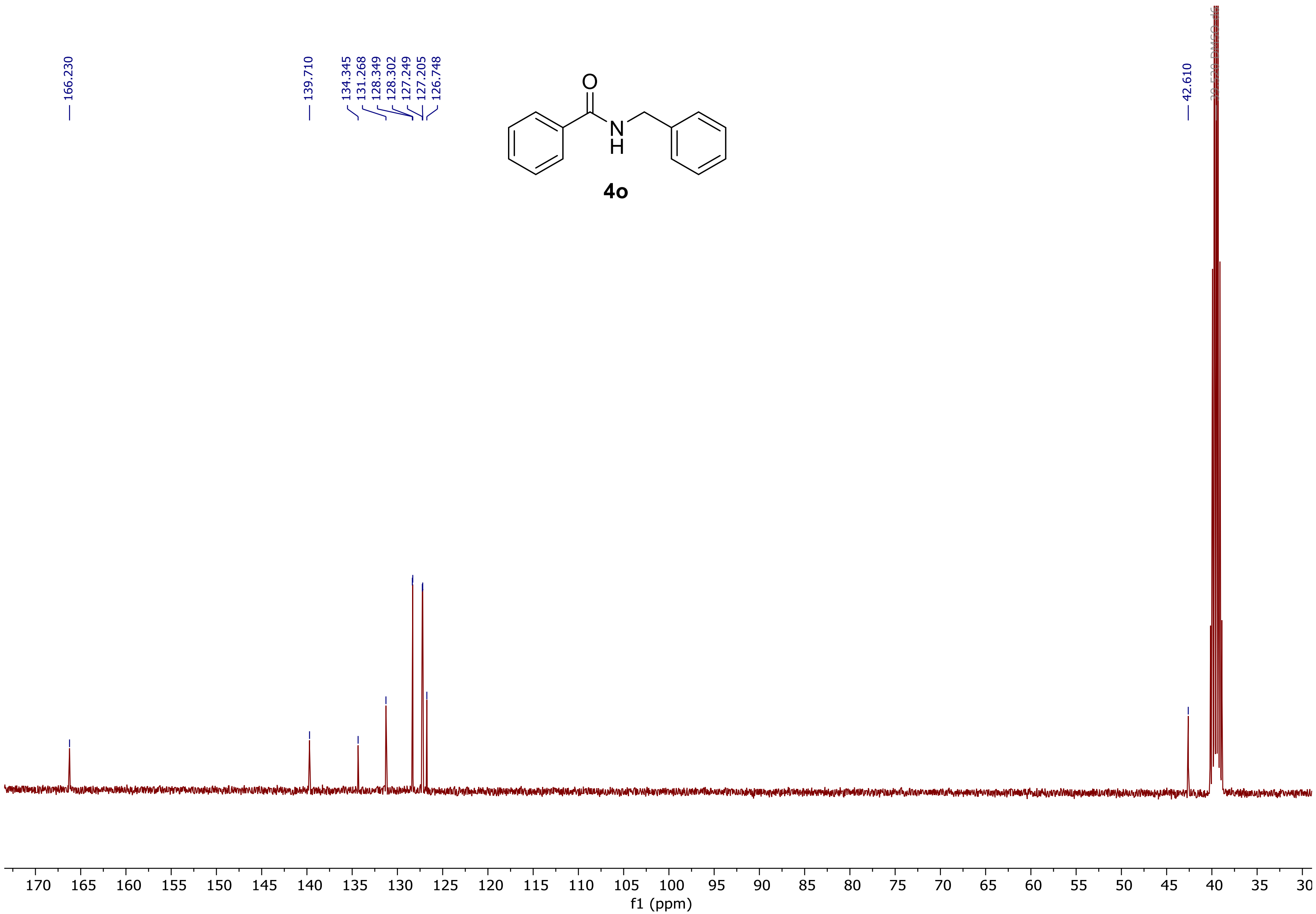

5.31 ^{1}H NMR spectrum 4p (400 MHz, DMSO-d$_6$)

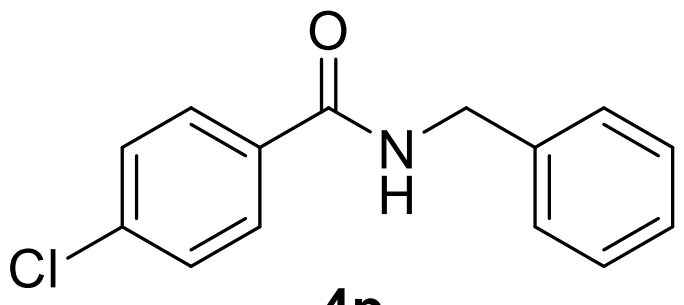

4p

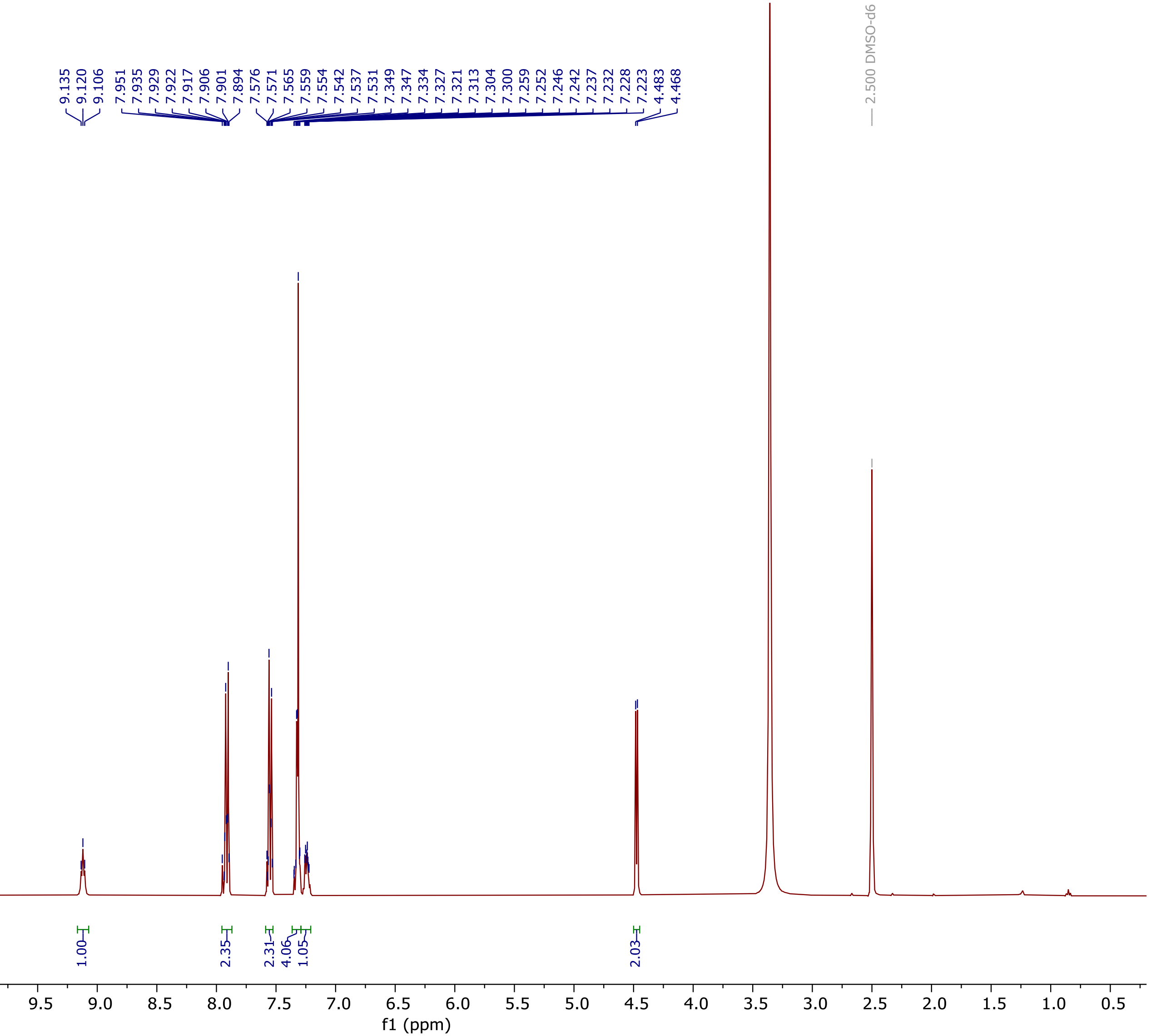

5.32 $^{13}C\{^{1}H\}$ NMR spectrum 4p (101 MHz, DMSO-d_6)

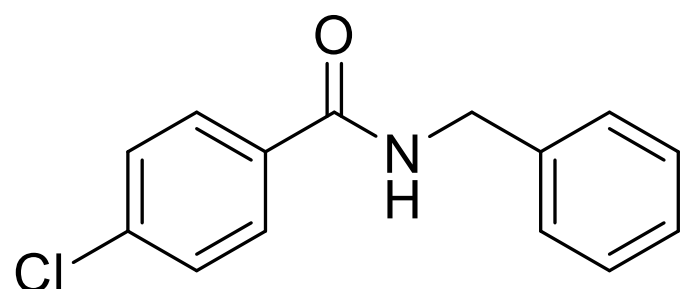

4p

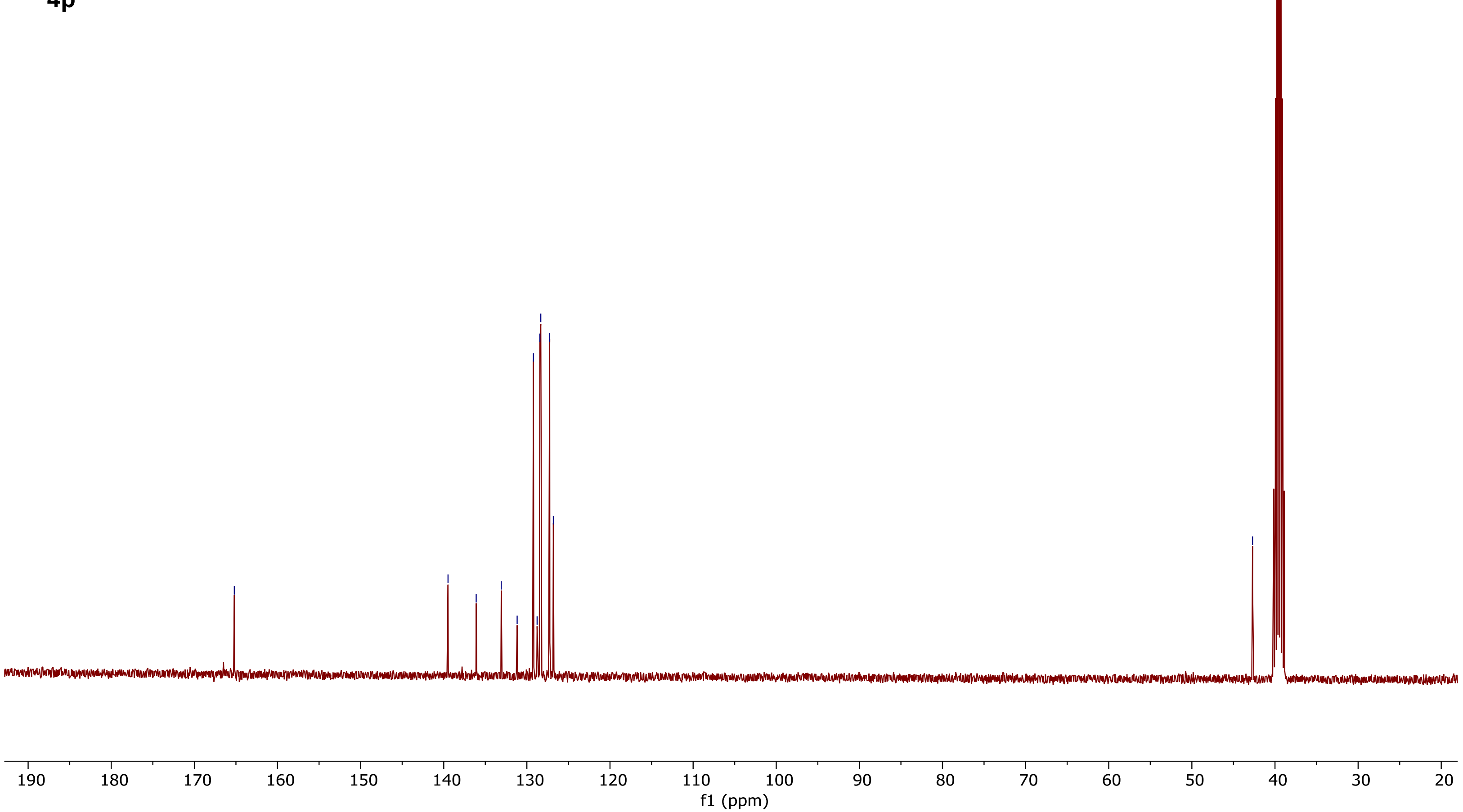

5.33 ^{1}H NMR spectrum 4q (400 MHz, $CDCl_3$)

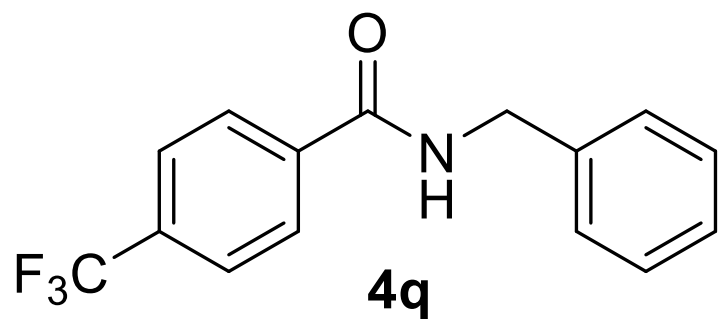

4q

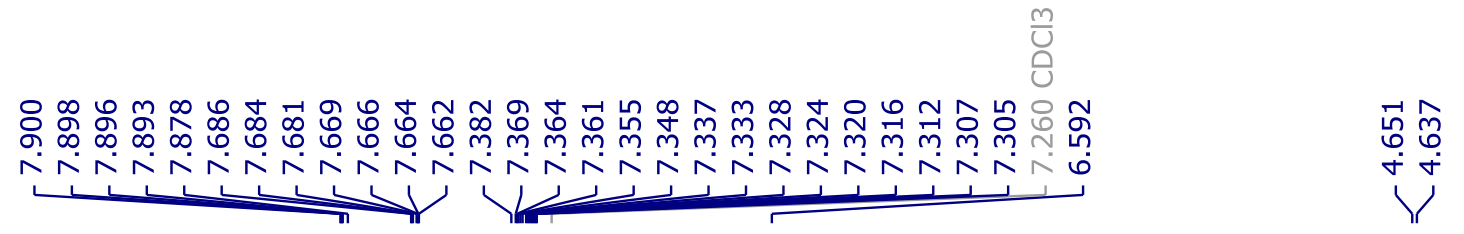

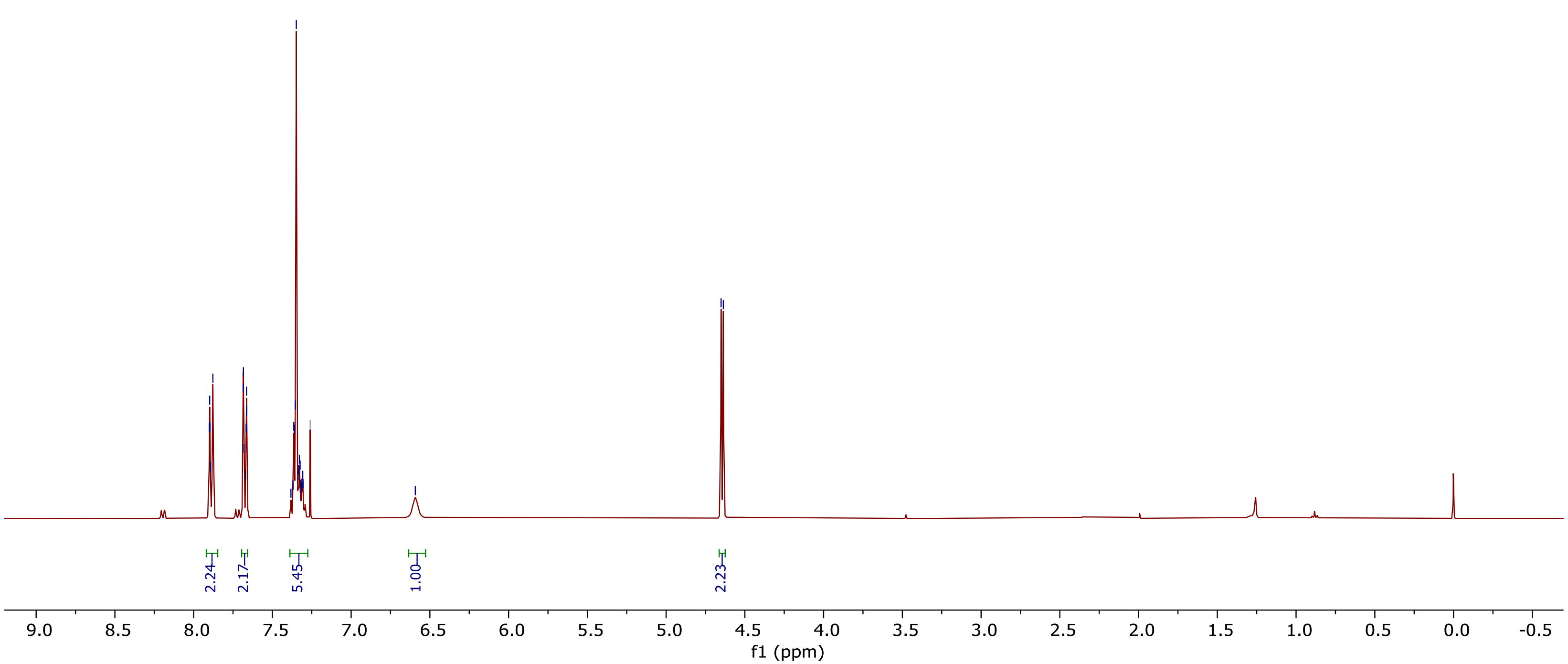

5.34 $^{13}C\{^{1}H\}$ NMR spectrum 4q (101 MHz, $CDCl_3$)

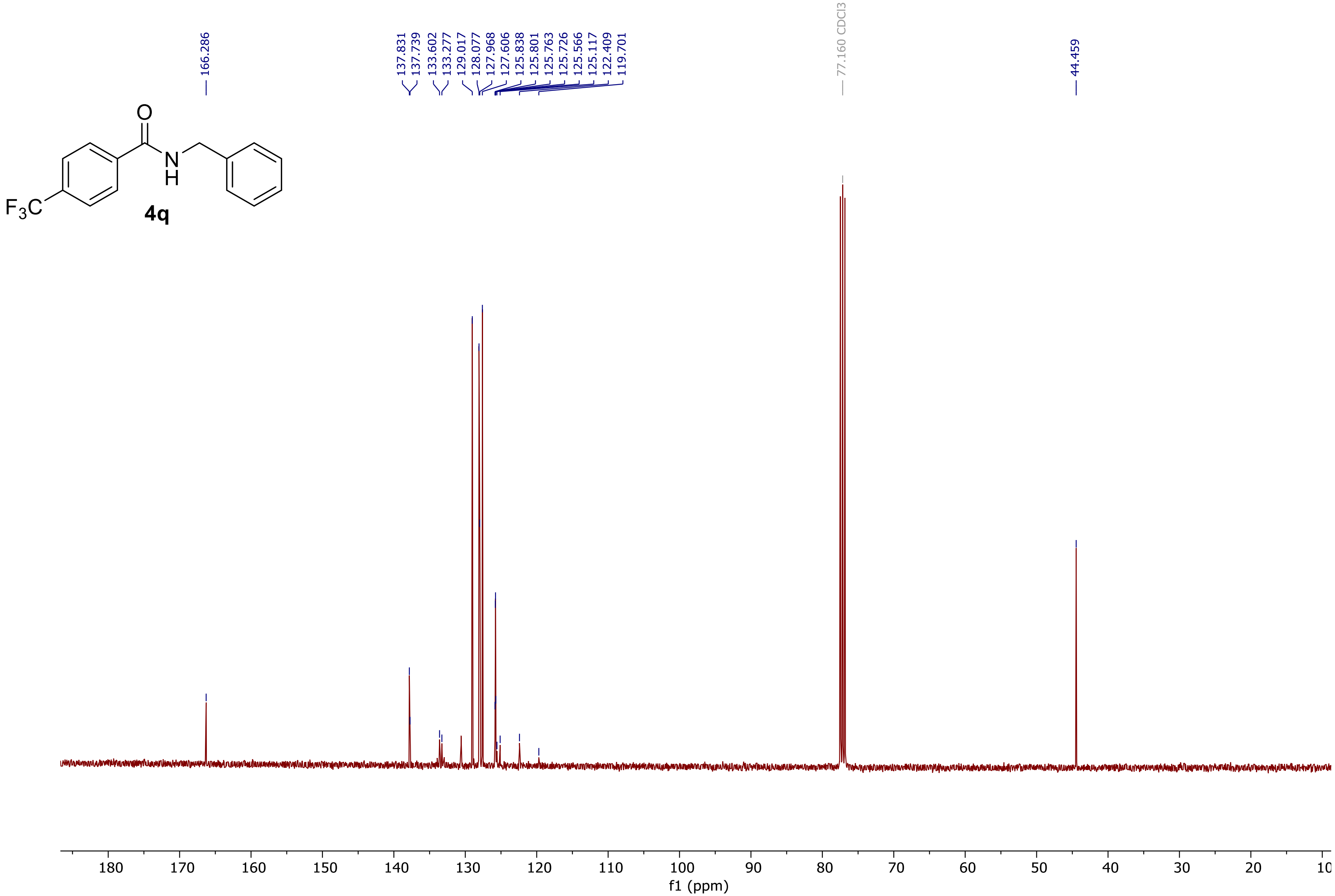

5.35 ^{1}H NMR spectrum 4r (400 MHz, DMSO-d_6)

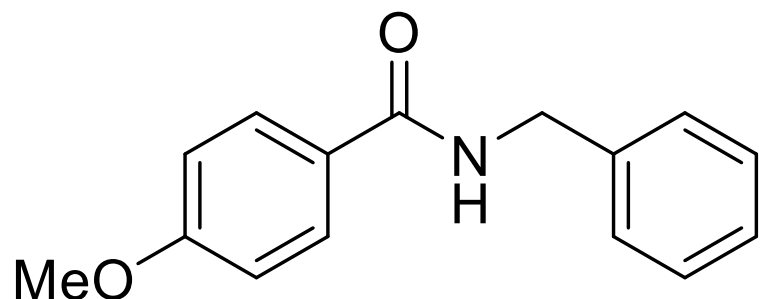

4r

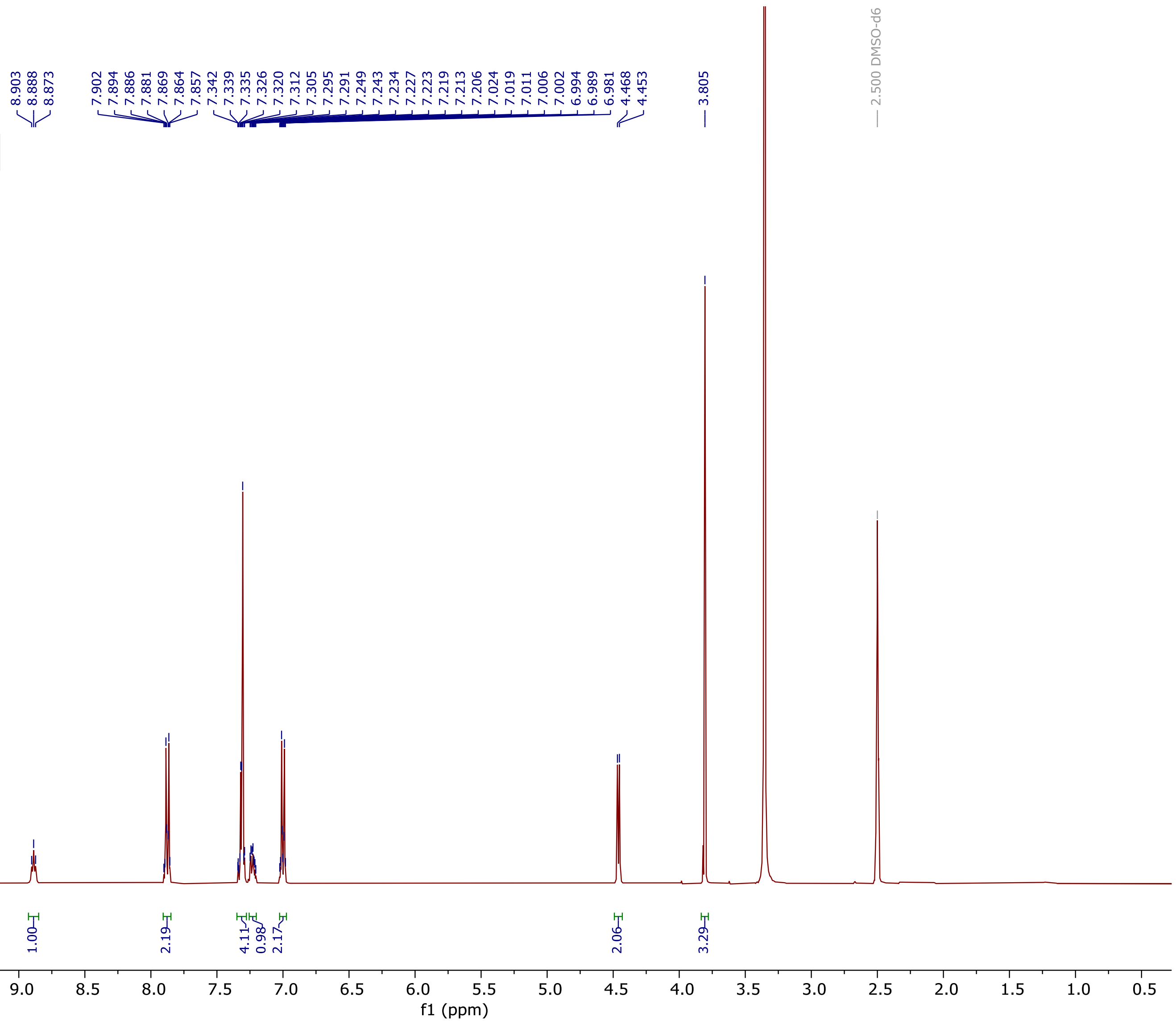

5.36 $^{13}C\{^{1}H\}$ NMR spectrum 4r (101 MHz, DMSO-d$_6$)

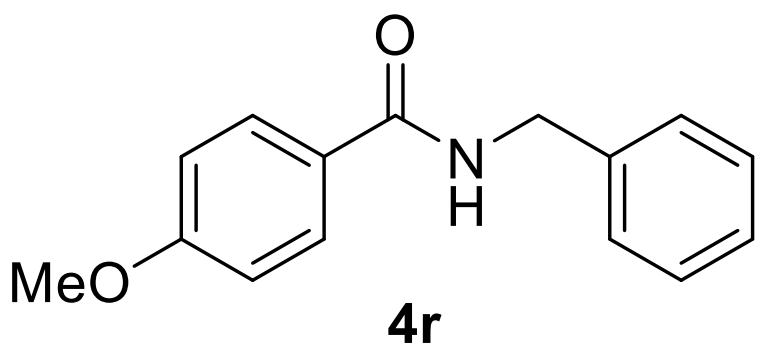

4r

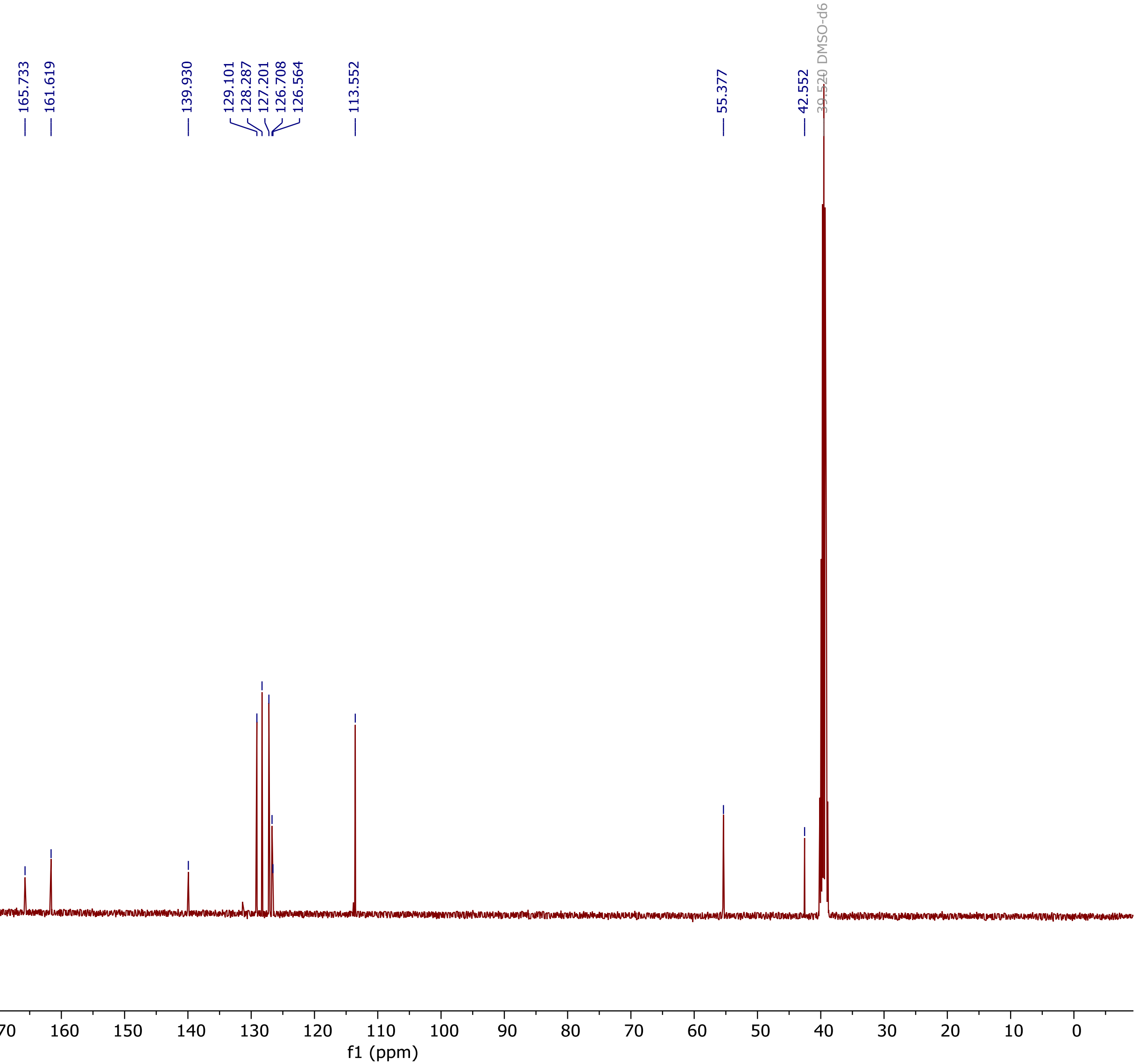

5.37 ^{1}H NMR spectrum 4s (400 MHz, DMSO-d_6)

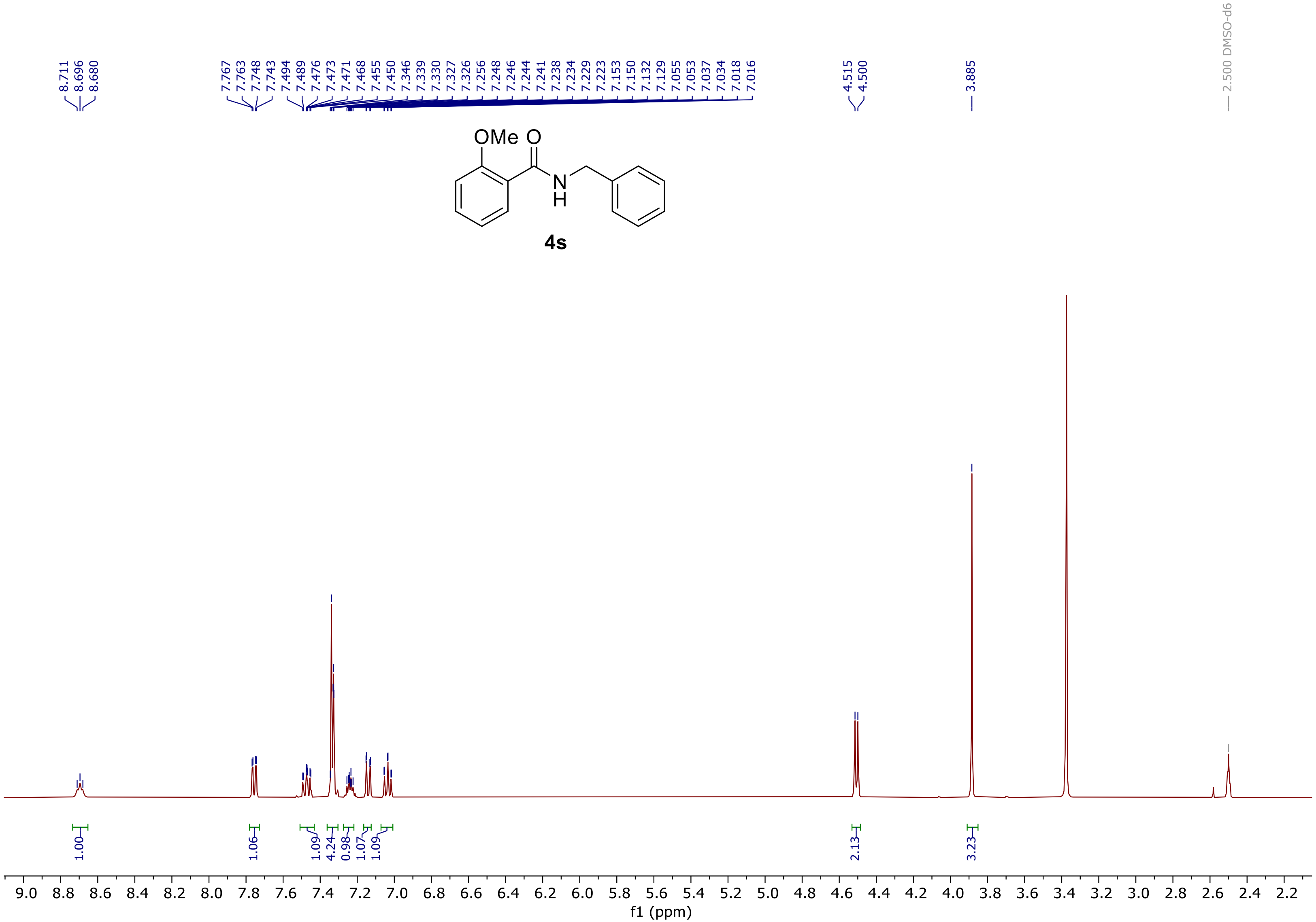

5.38 $^{13}C\{^{1}H\}$ NMR spectrum 4s (101 MHz, DMSO-d$_6$)

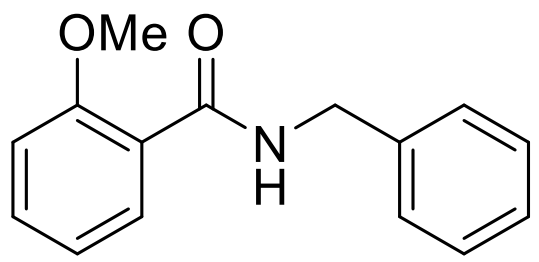

4s

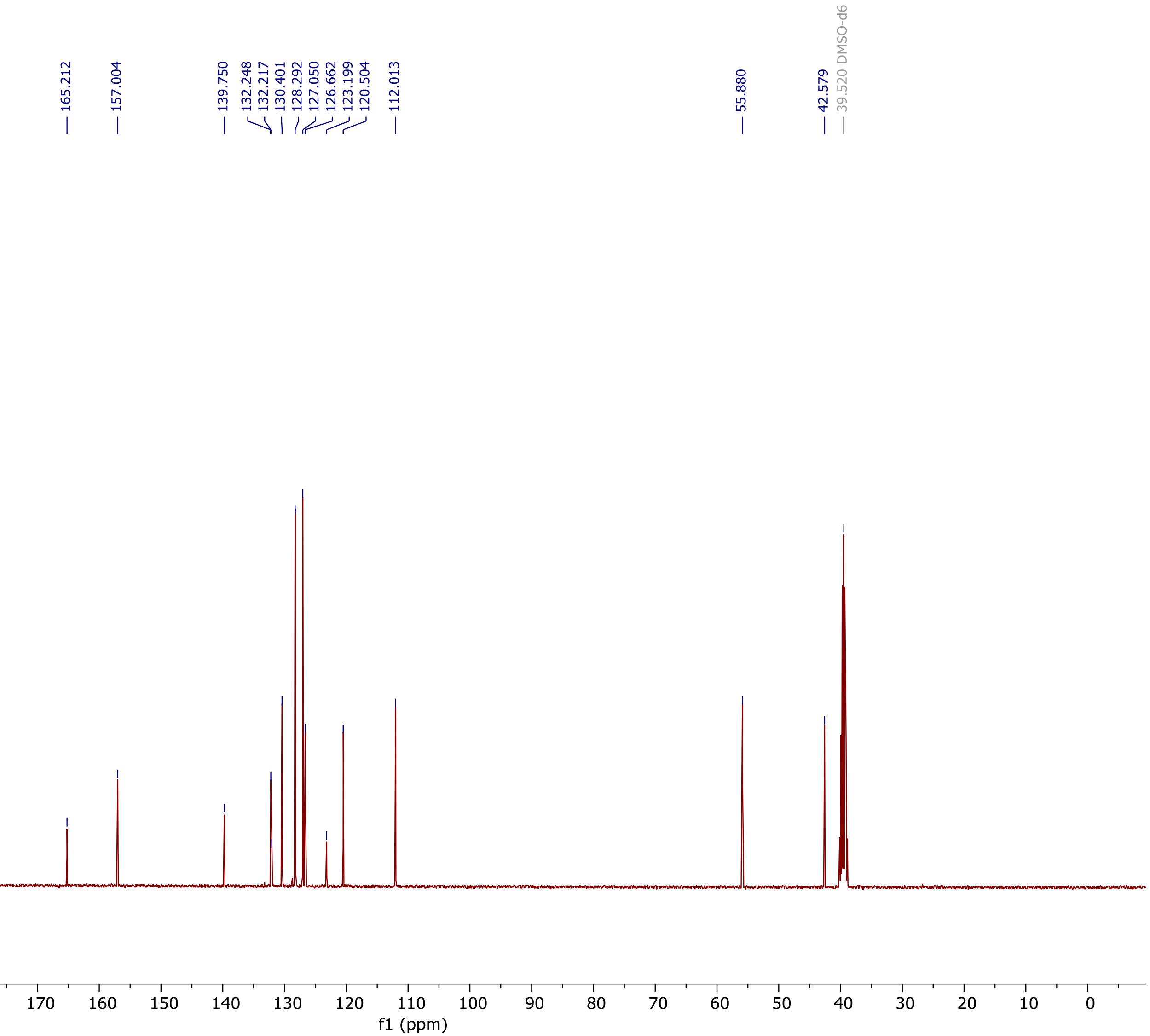

5.39 ^{1}H NMR spectrum 4t (400 MHz, DMSO-d_6)

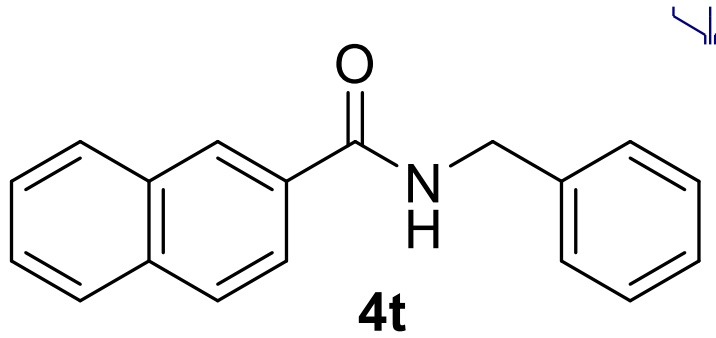
4t

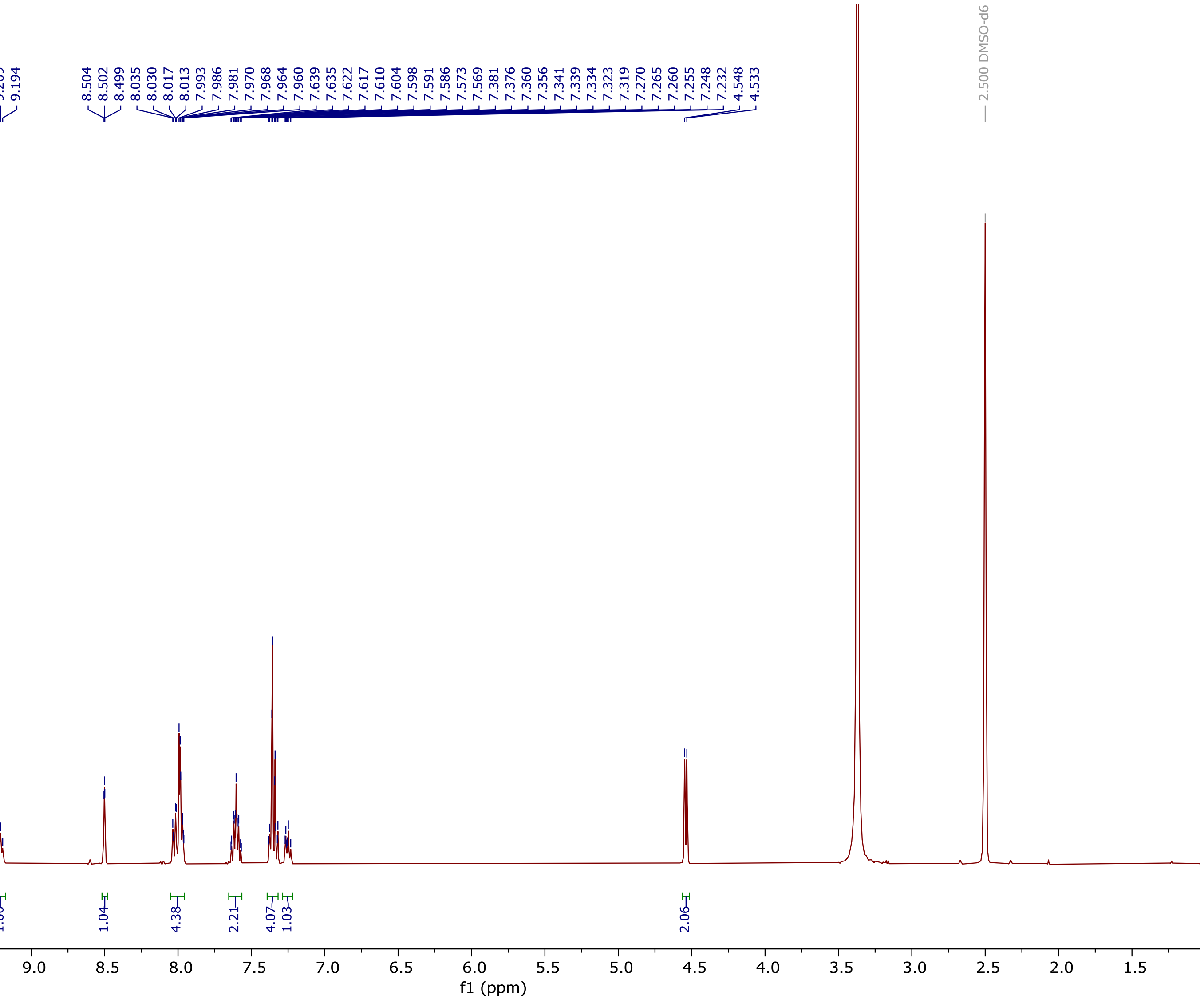

5.40 $^{13}C\{^{1}H\}$ NMR spectrum 4t (101 MHz, DMSO-d$_6$)

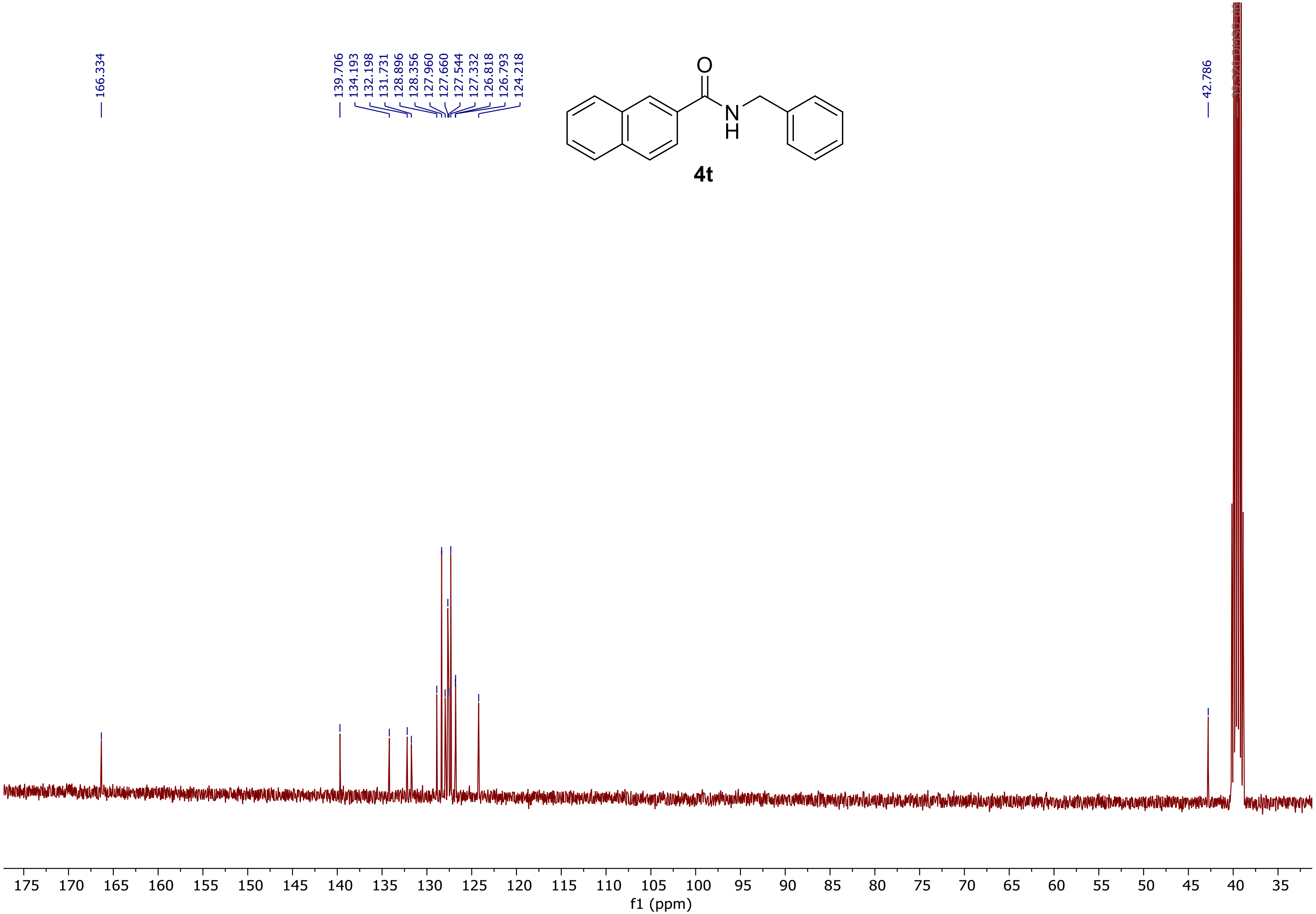

5.41 ^{1}H NMR spectrum 4u (400 MHz, DMSO-d_6)

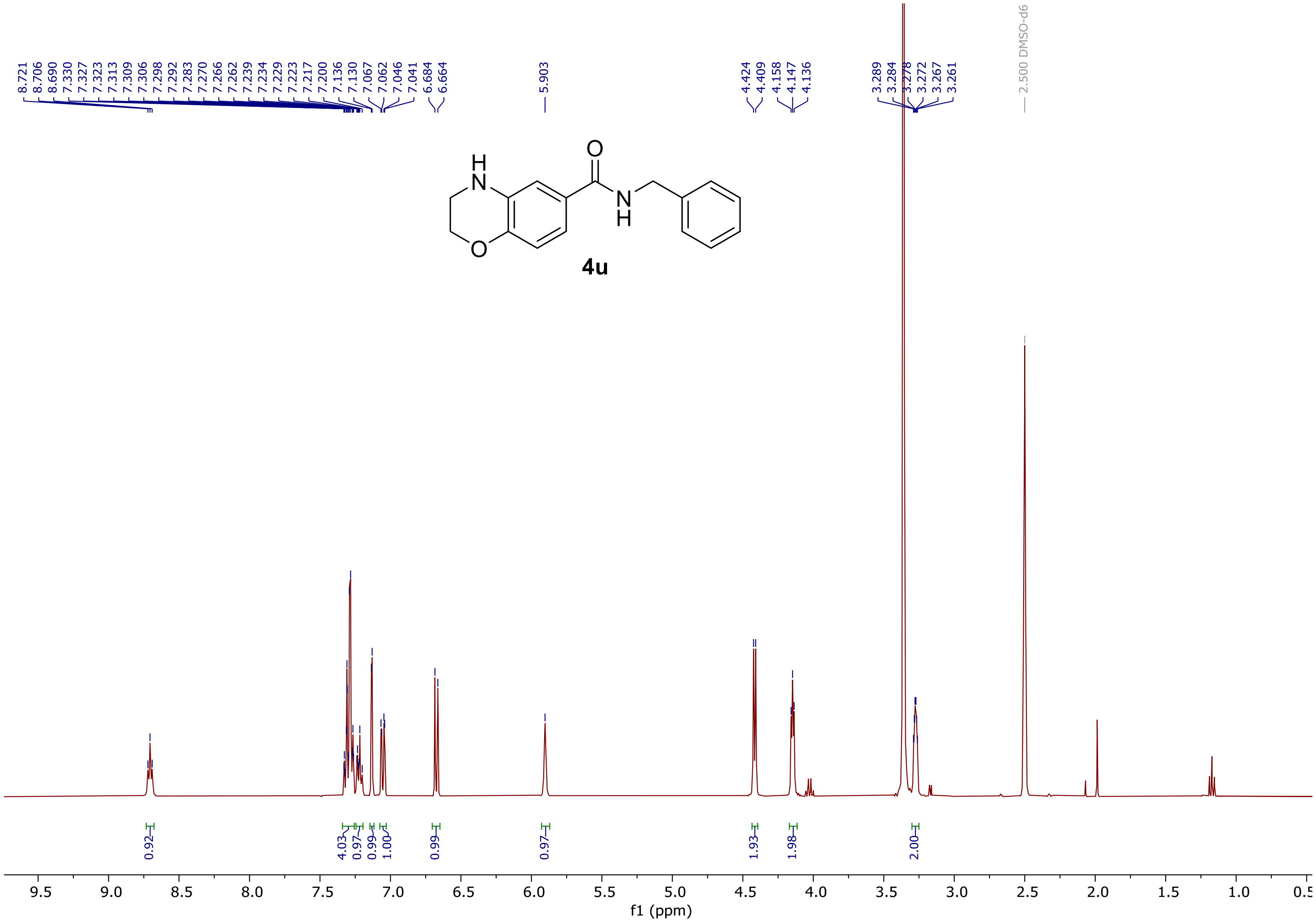

5.42 $^{13}C\{^{1}H\}$ NMR spectrum 4u (101 MHz, DMSO-d$_6$)

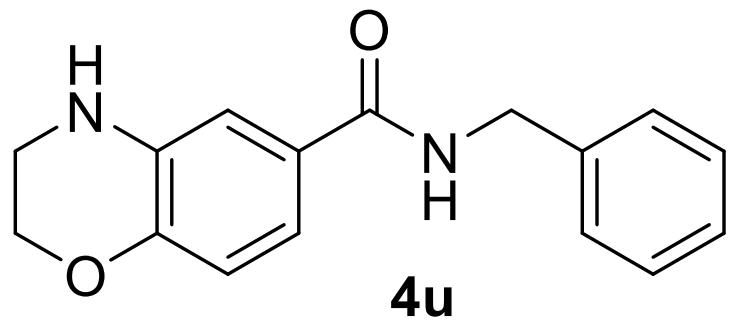

4u

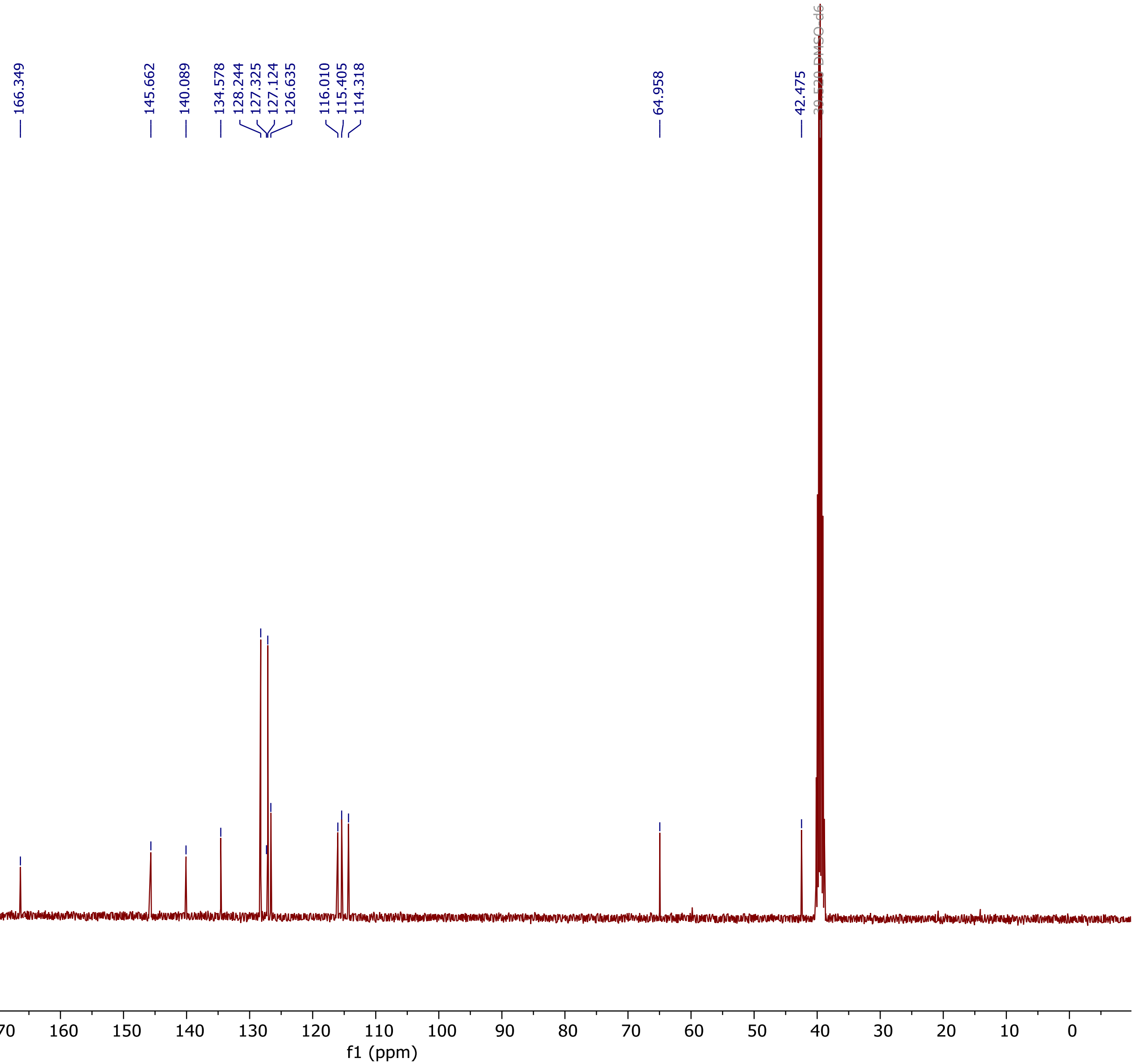

5.43 ^{1}H NMR spectrum 4v (400 MHz, DMSO-d$_6$)

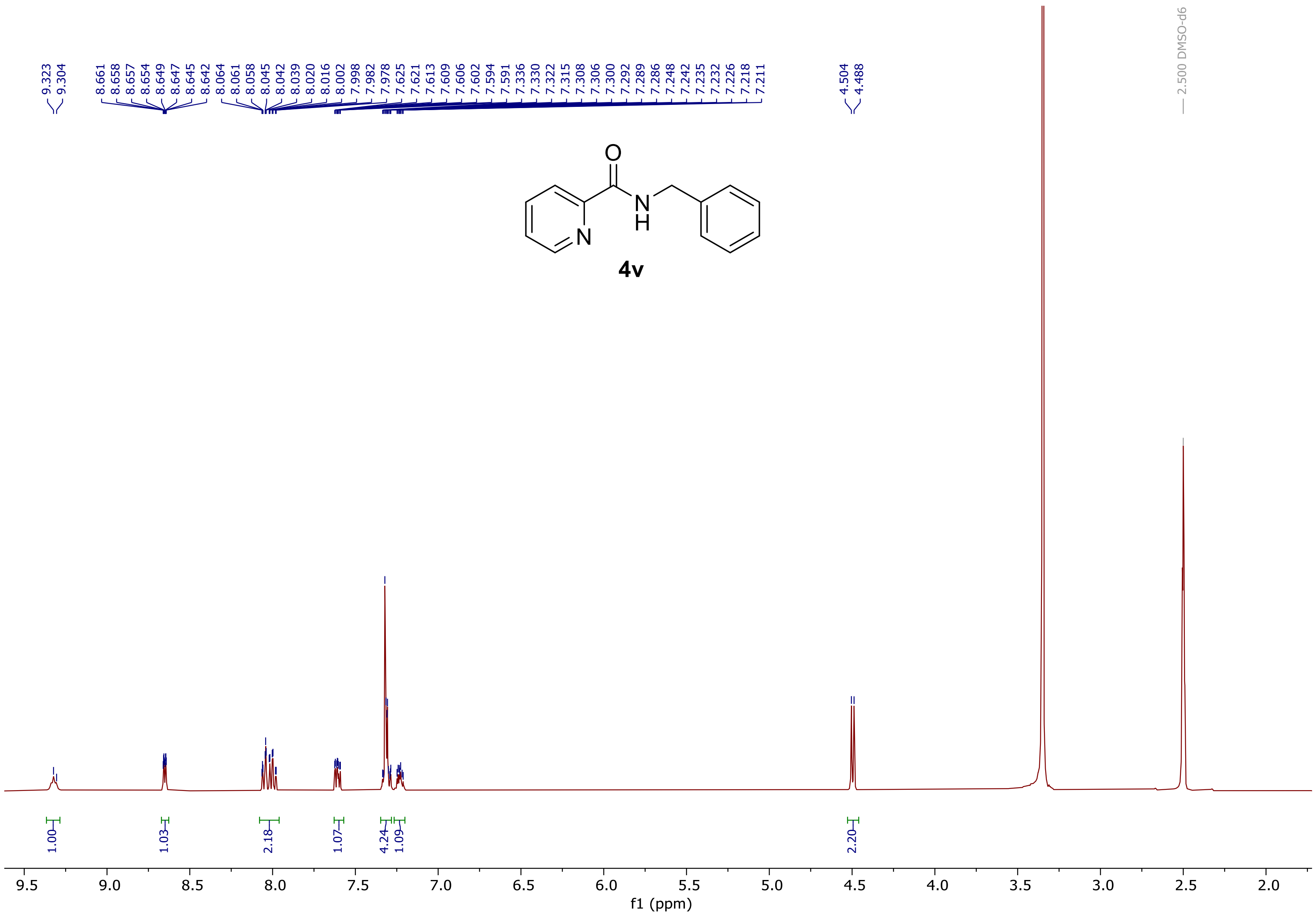

5.44 $^{13}C\{^{1}H\}$ NMR spectrum 4v (101 MHz, DMSO-d$_6$)

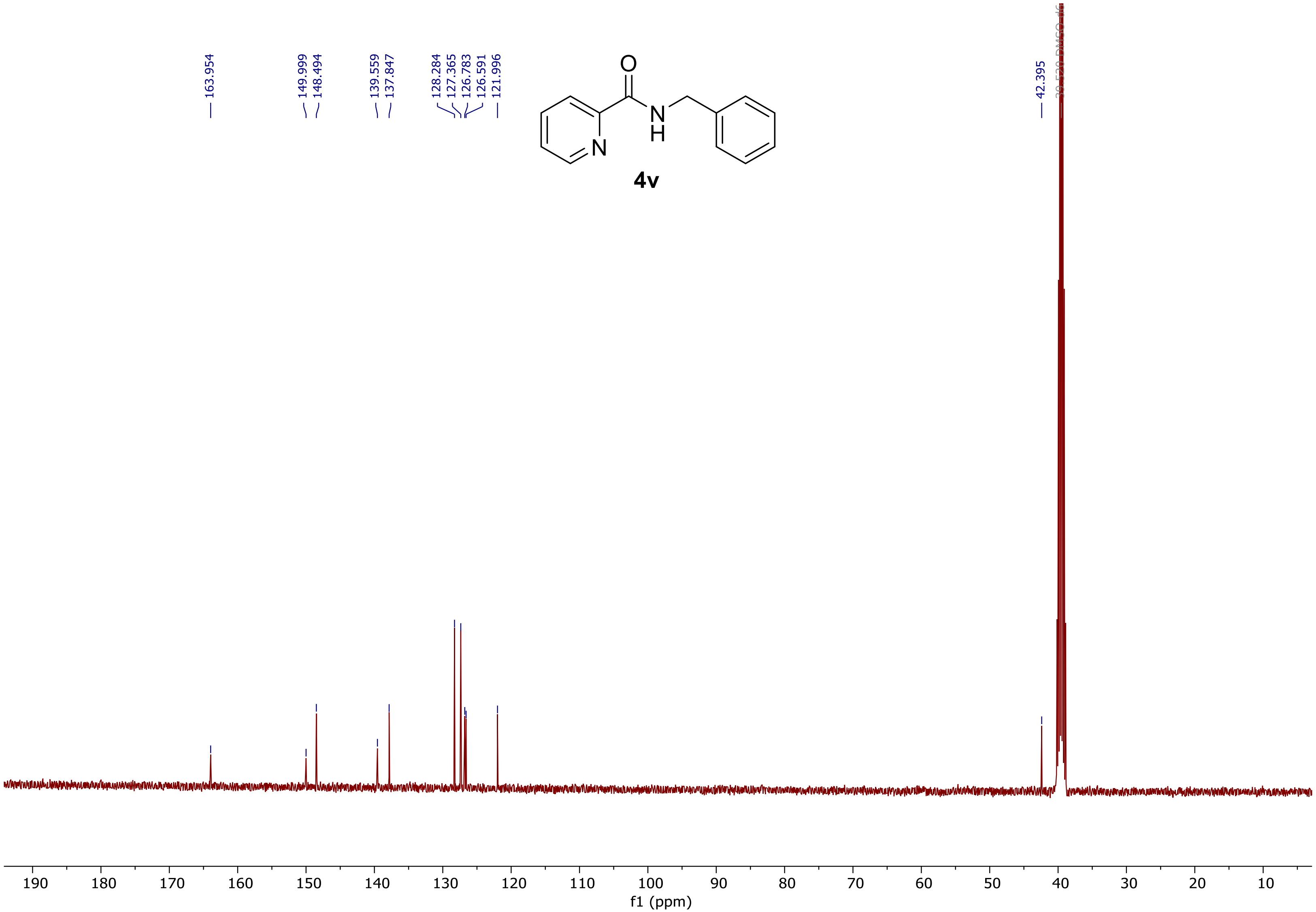

5.45 ^{1}H NMR spectrum 4w (400 MHz, DMSO-d$_6$)

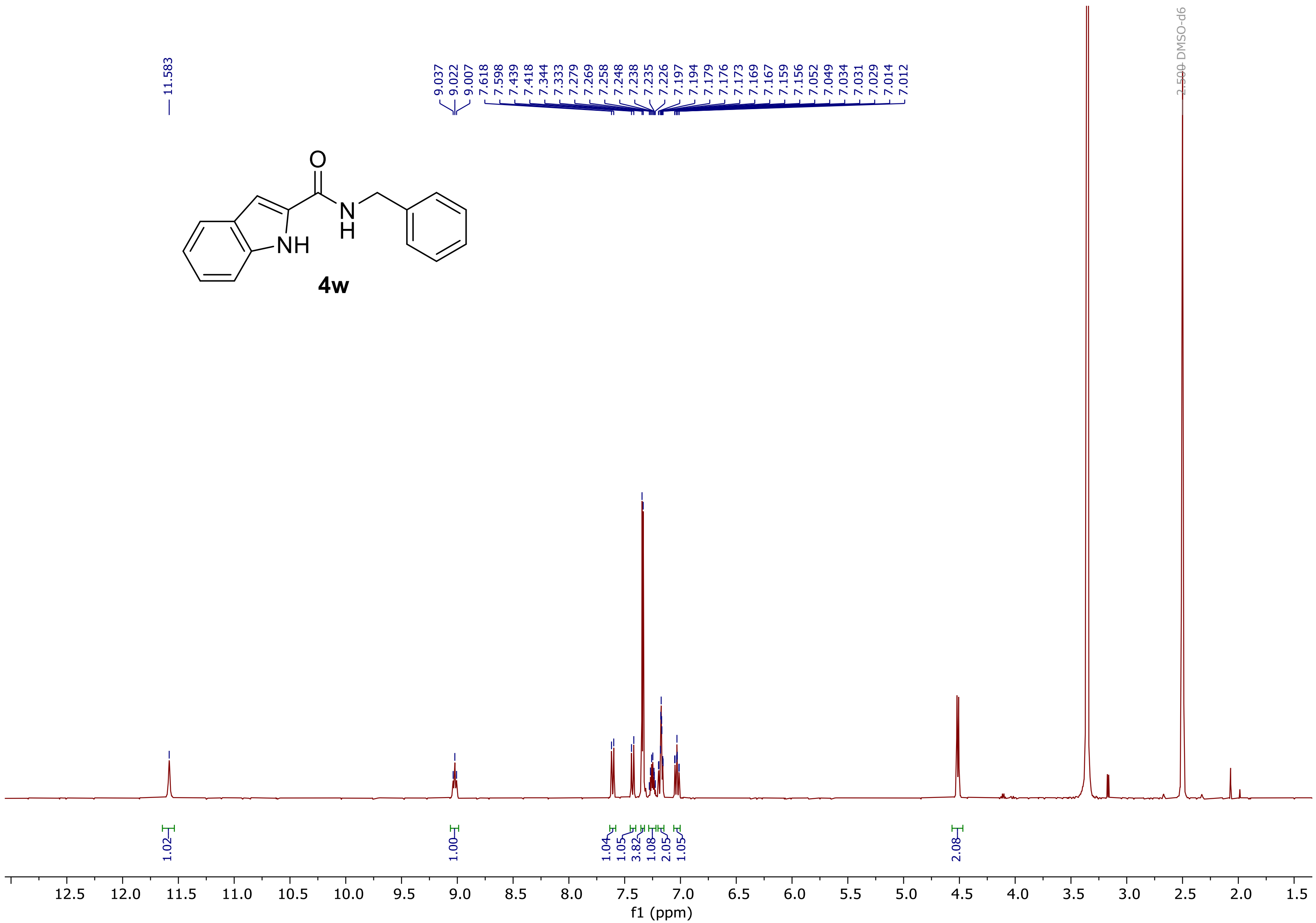

5.46 $^{13}C\{^1H\}$ NMR spectrum 4w (101 MHz, DMSO-d_6)

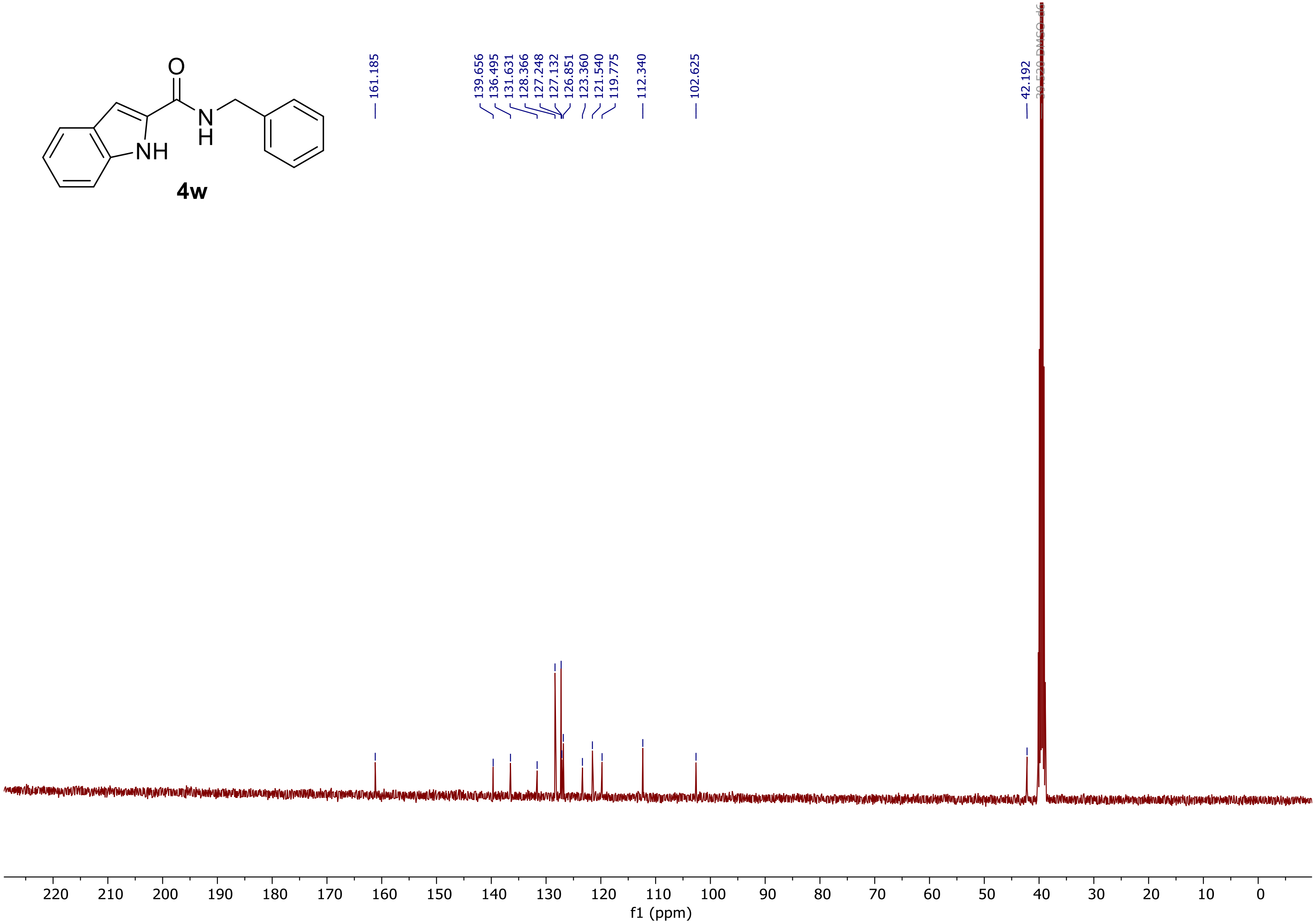

5.47 ^{1}H NMR spectrum 4x (400 MHz, DMSO-d_6)

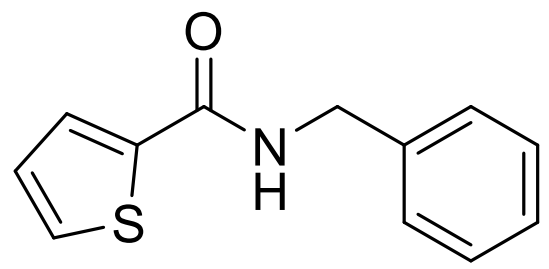

4x

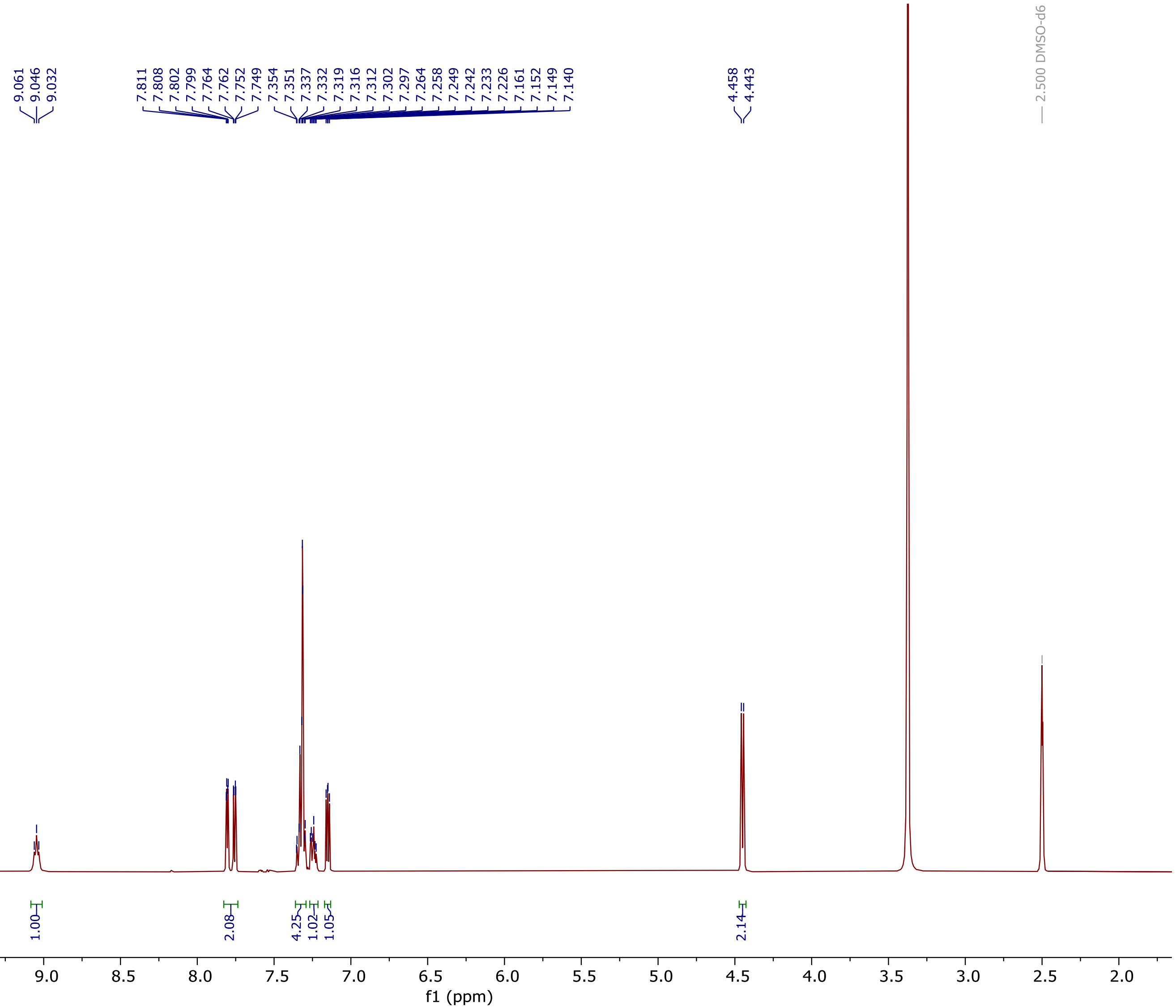

5.48 $^{13}C\{^{1}H\}$ NMR spectrum 4x (101 MHz, DMSO-d_6)

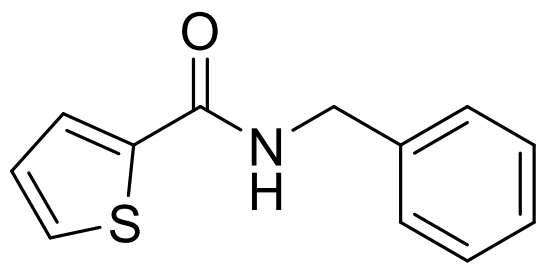

4x

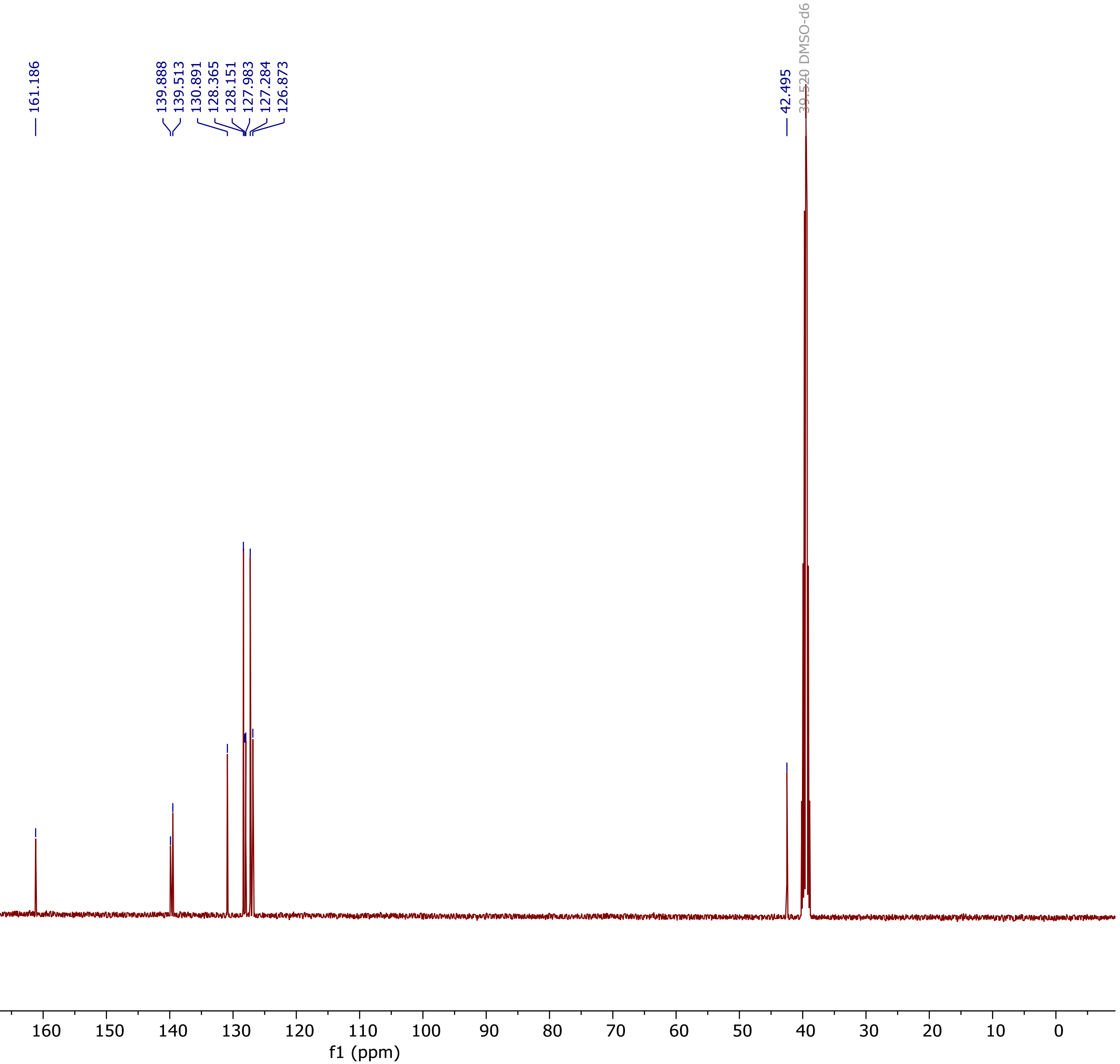

5.49 ^{1}H NMR spectrum 4y (400 MHz, $CDCl_3$)

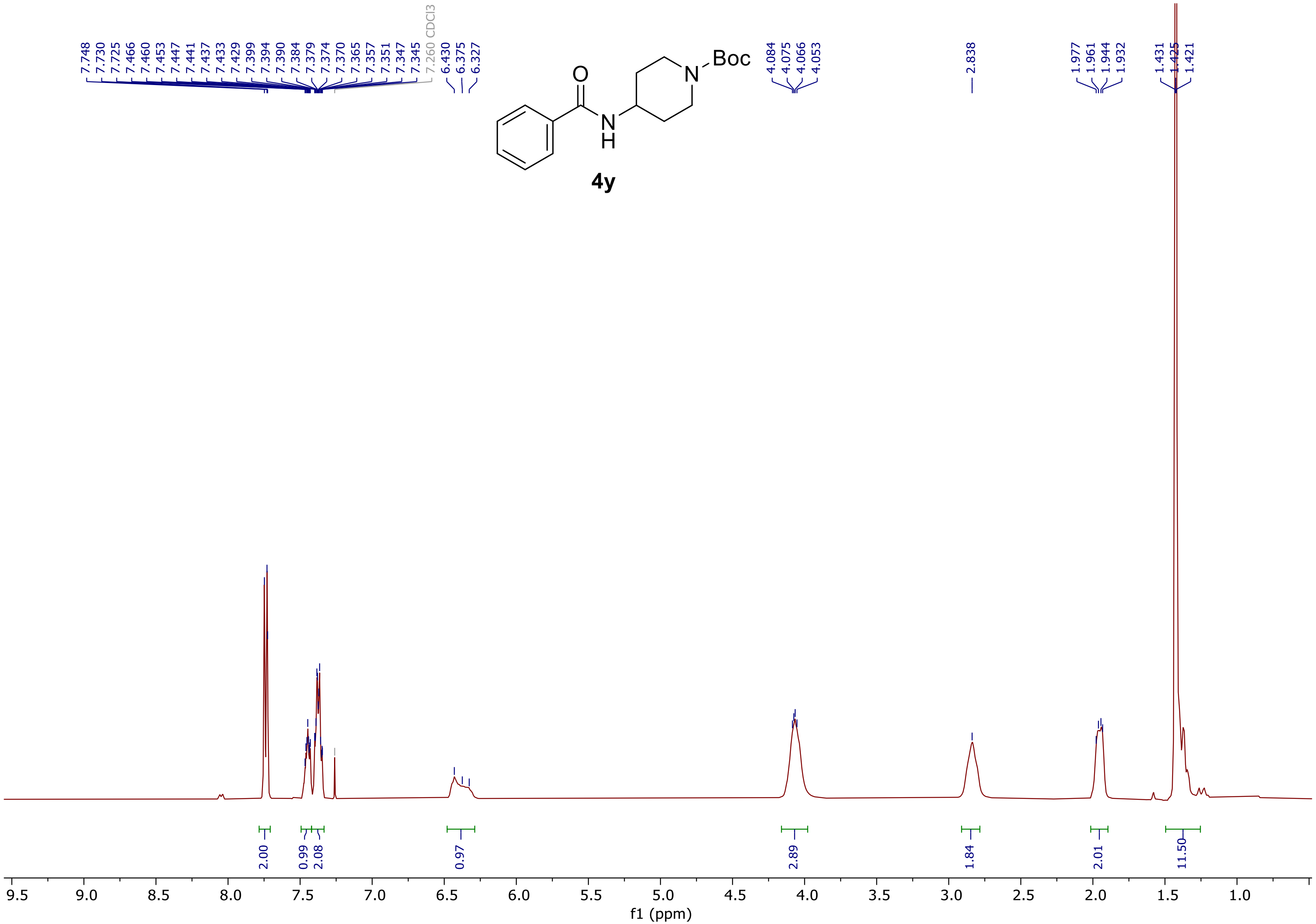

5.50 $^{13}C\{^{1}H\}$ NMR spectrum 4y (101 MHz, $CDCl_3$)

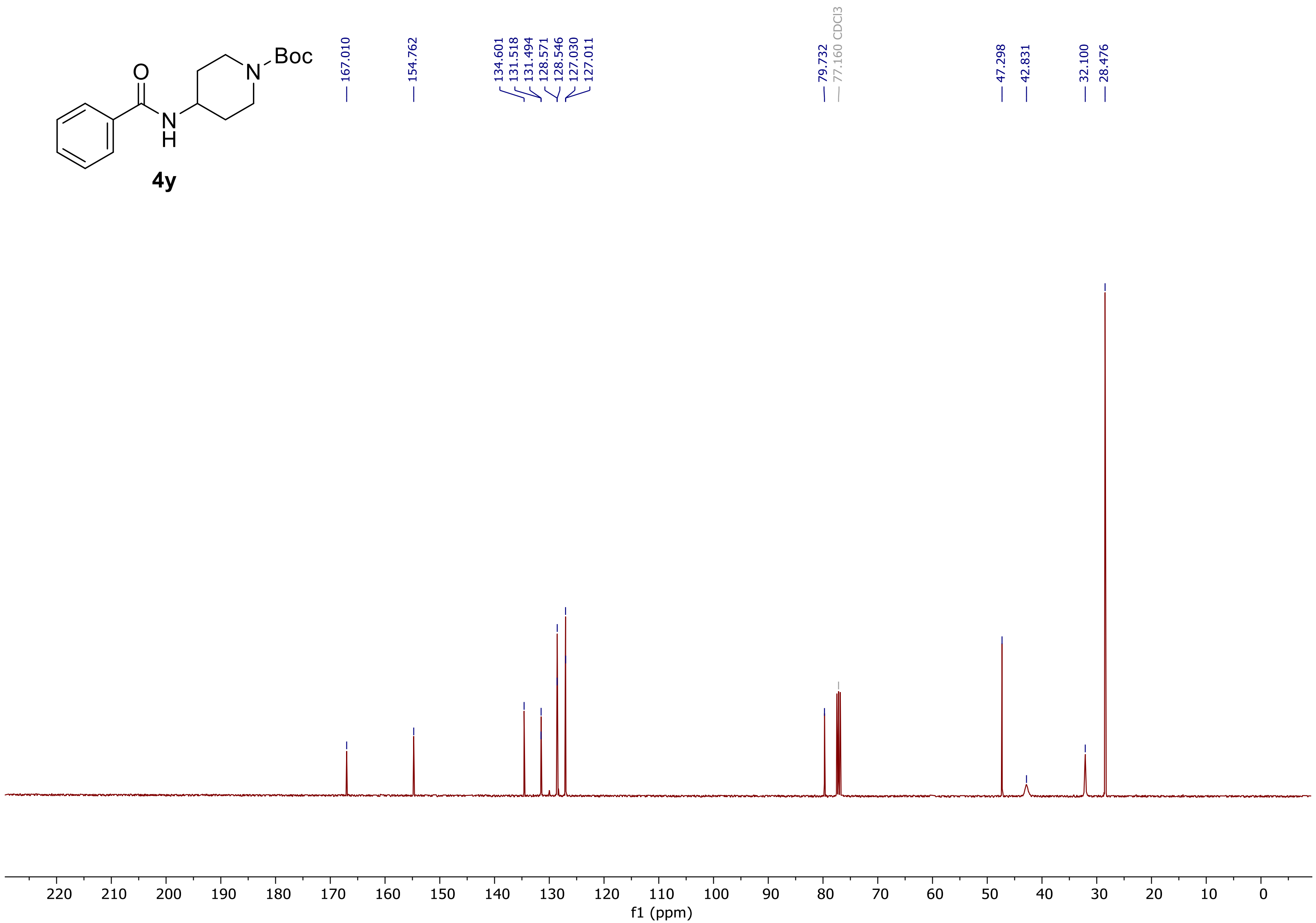

5.51 ^{1}H NMR spectrum 4z (400 MHz, DMSO-d_6)

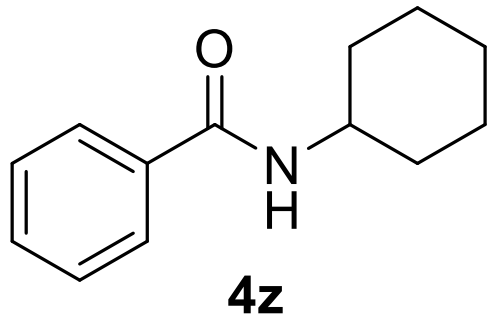

4z

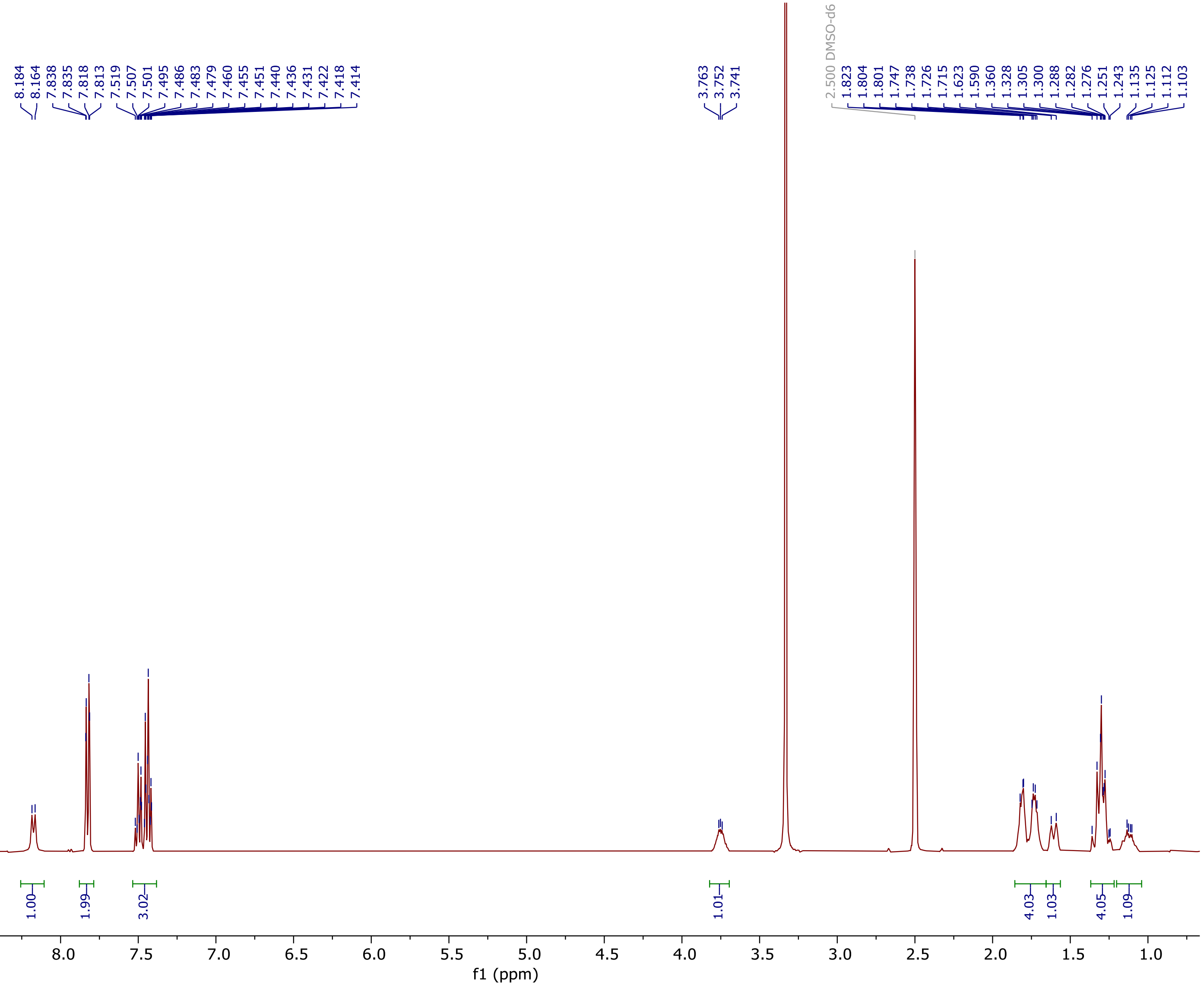

5.52 $^{13}C\{^{1}H\}$ NMR spectrum 4z (101 MHz, DMSO-d$_6$)

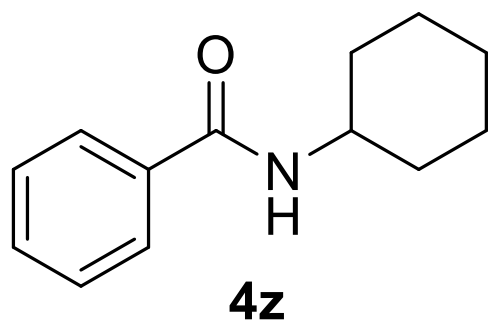

4z

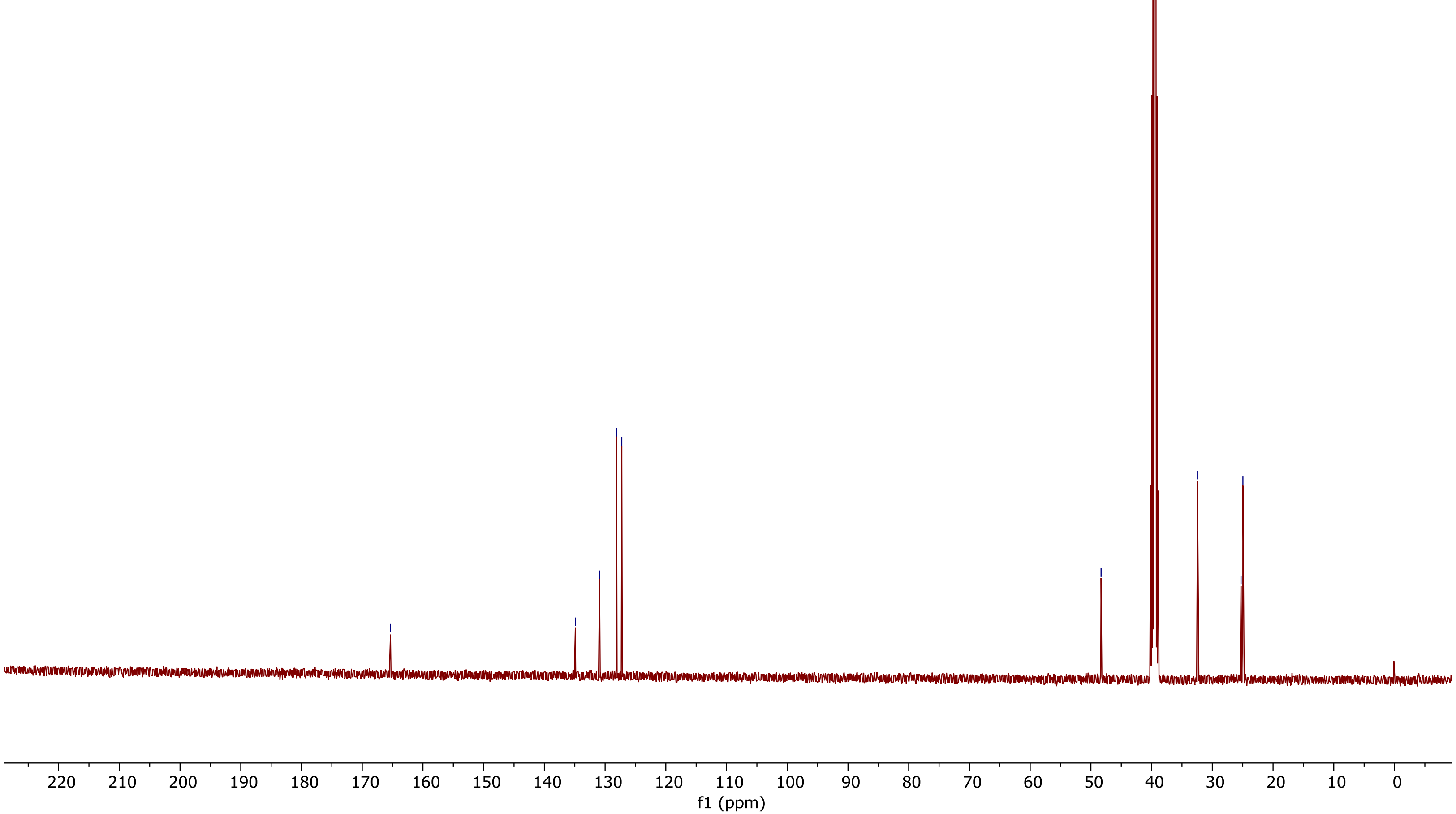

5.53 ^{1}H NMR spectrum 4aa (400 MHz, DMSO-d_6)

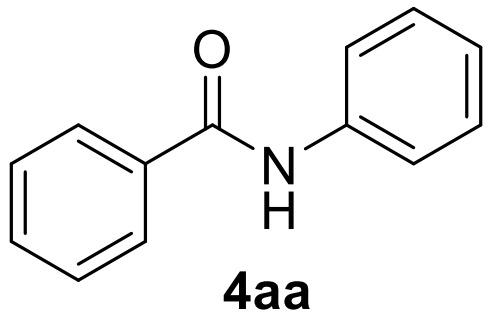

4aa

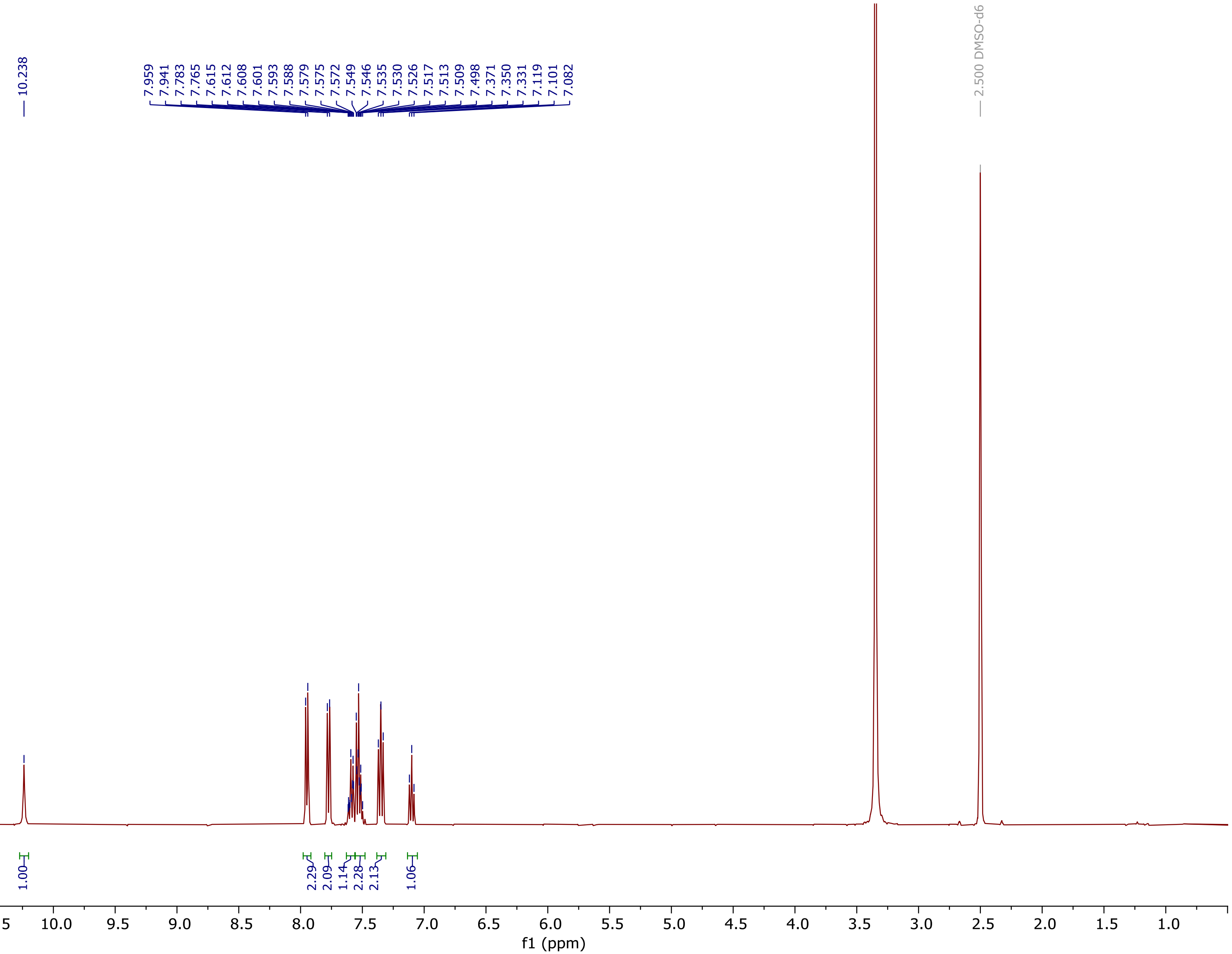

5.54 $^{13}C\{^{1}H\}$ NMR spectrum 4aa (101 MHz, DMSO-d$_6$)

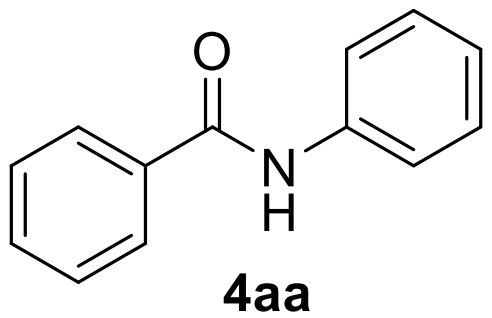

4aa

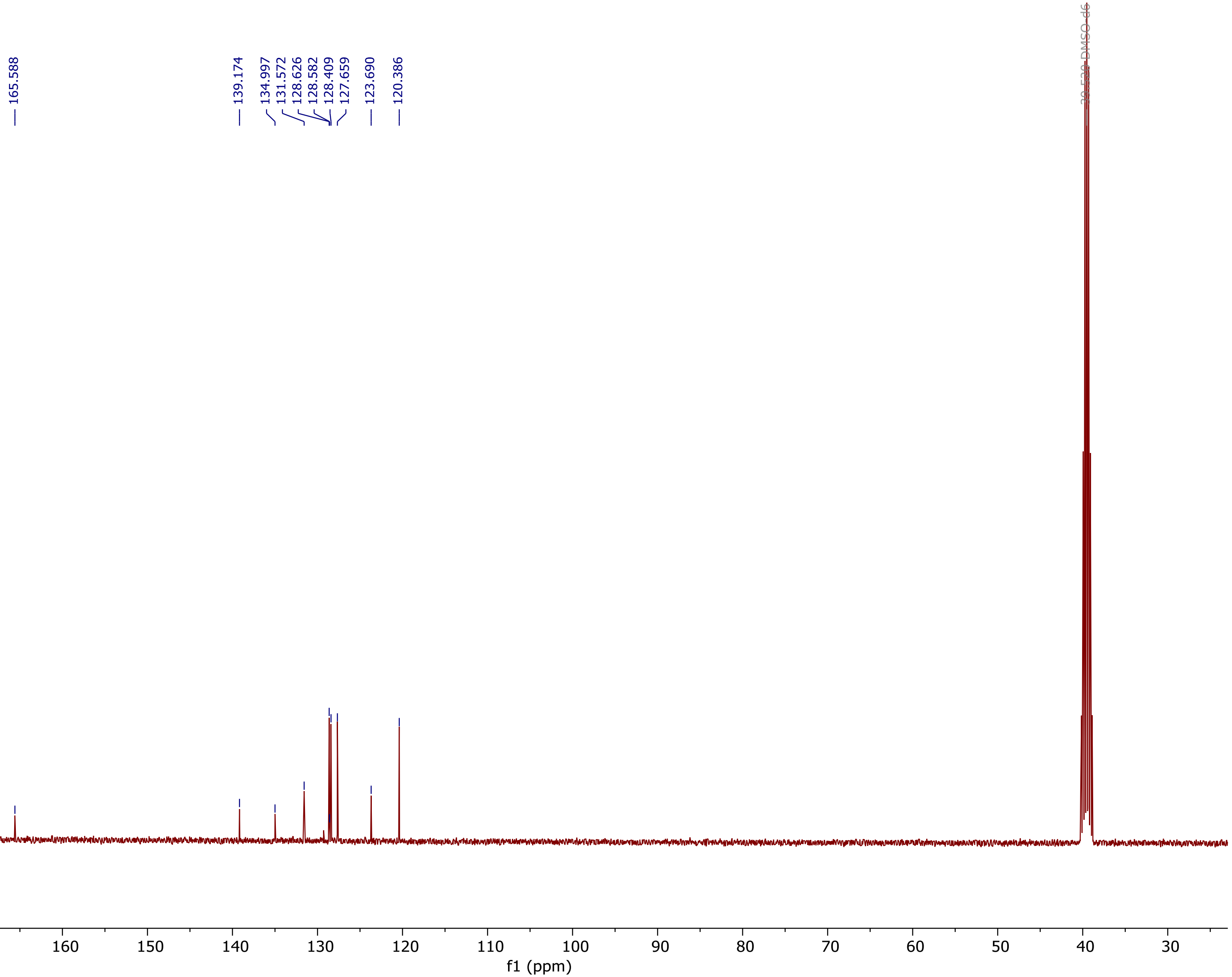

5.55 ^{1}H NMR spectrum 4ab (400 MHz, DMSO-d_6)

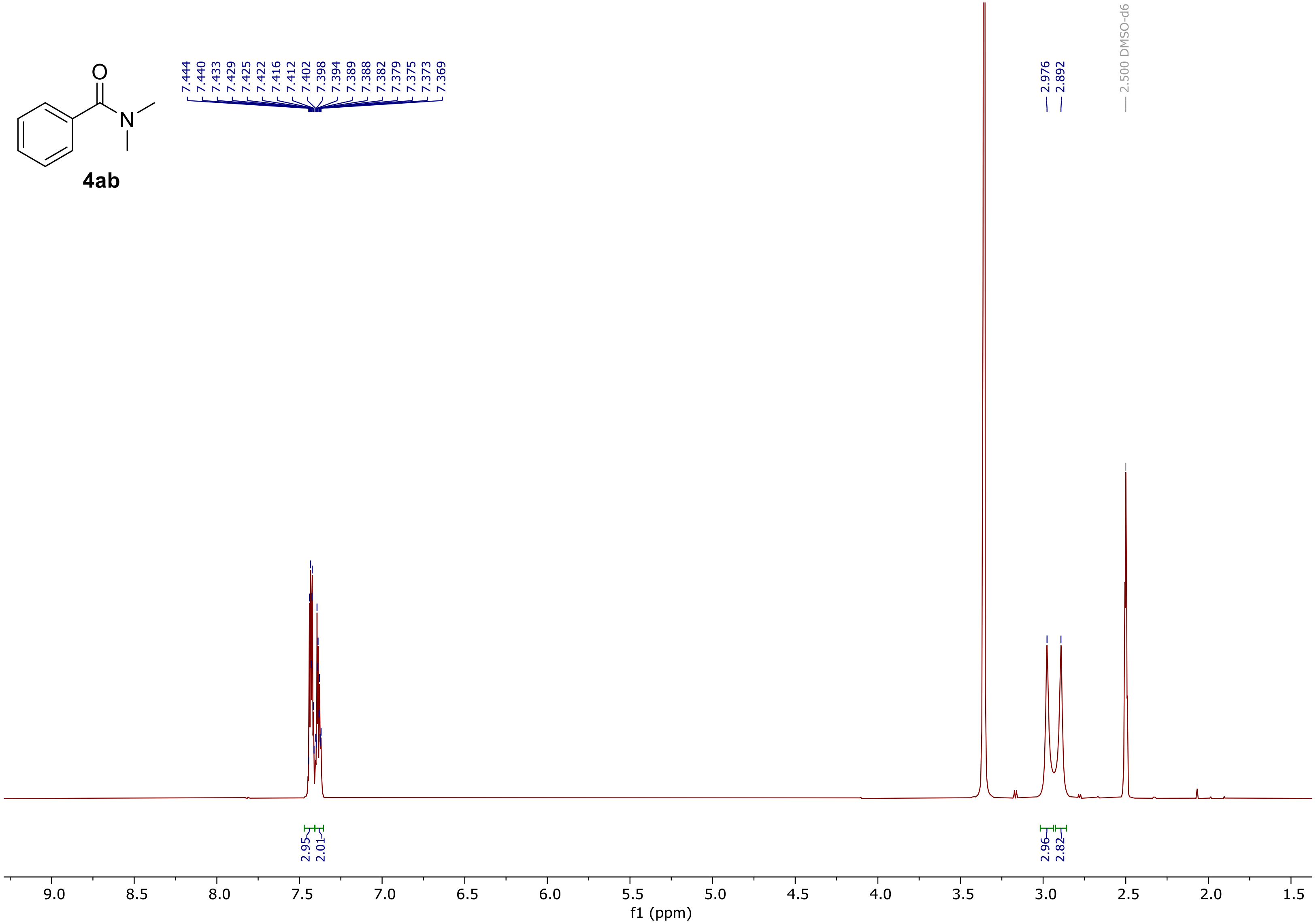

5.56 $^{13}C\{^1H\}$ NMR spectrum 4ab (101 MHz, DMSO-d$_6$)

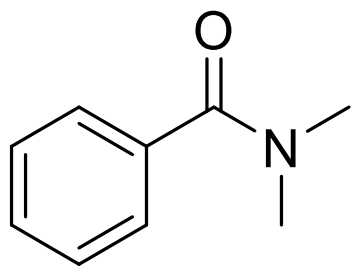

4ab

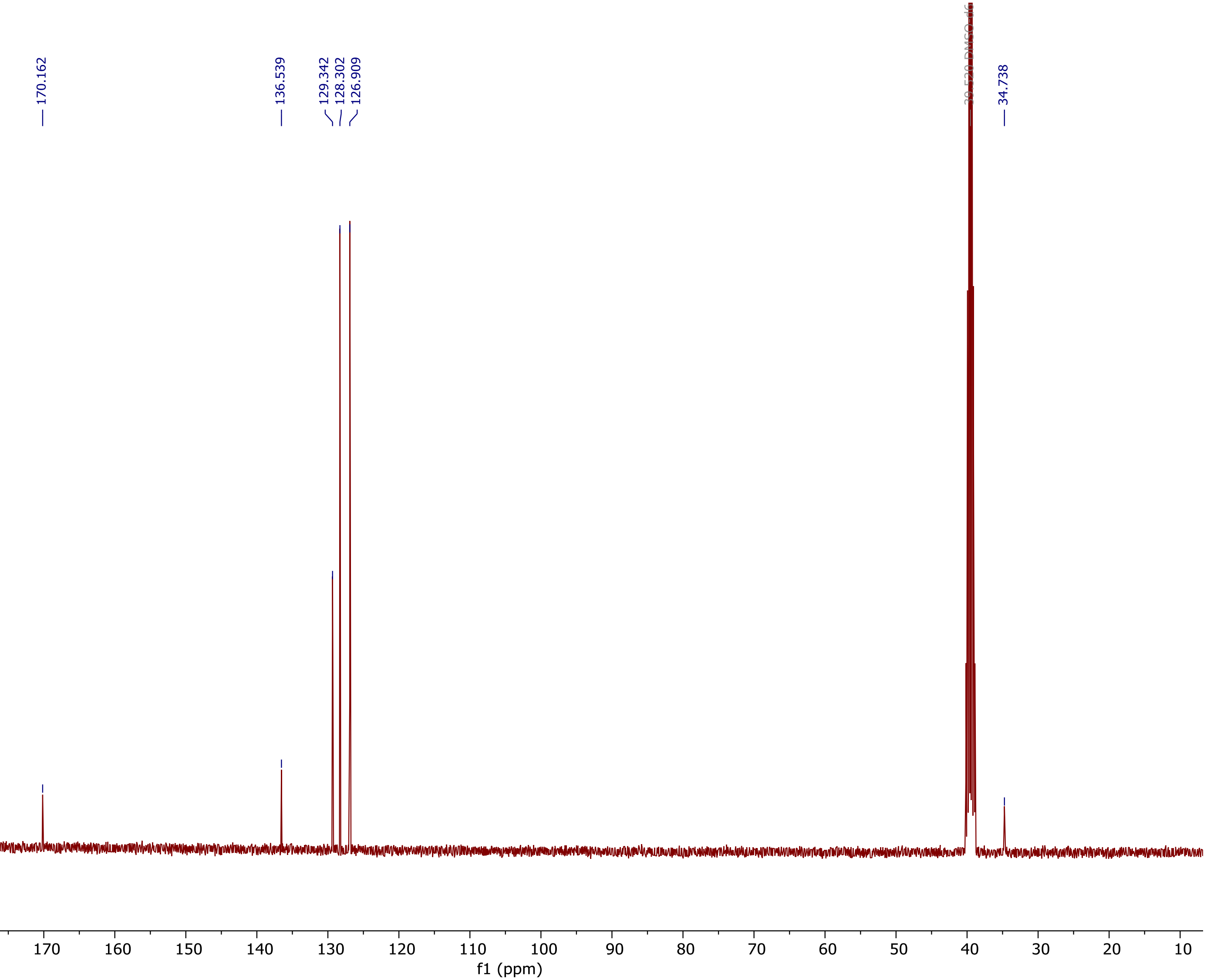

5.57 ^{1}H NMR spectrum 4ac (400 MHz, $CDCl_3$)

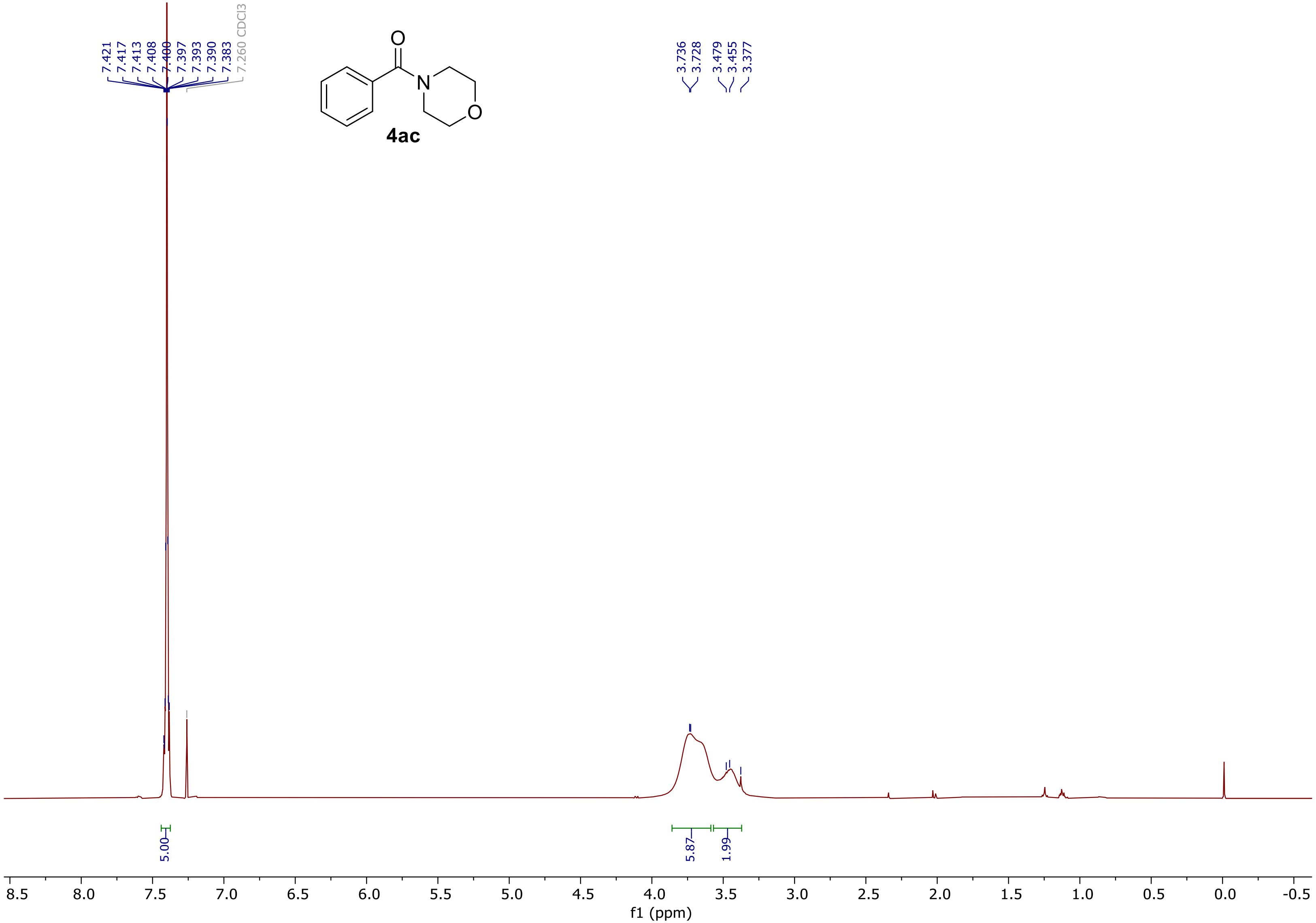

5.58 $^{13}C\{^{1}H\}$ NMR spectrum 4ac (101 MHz, $CDCl_3$)

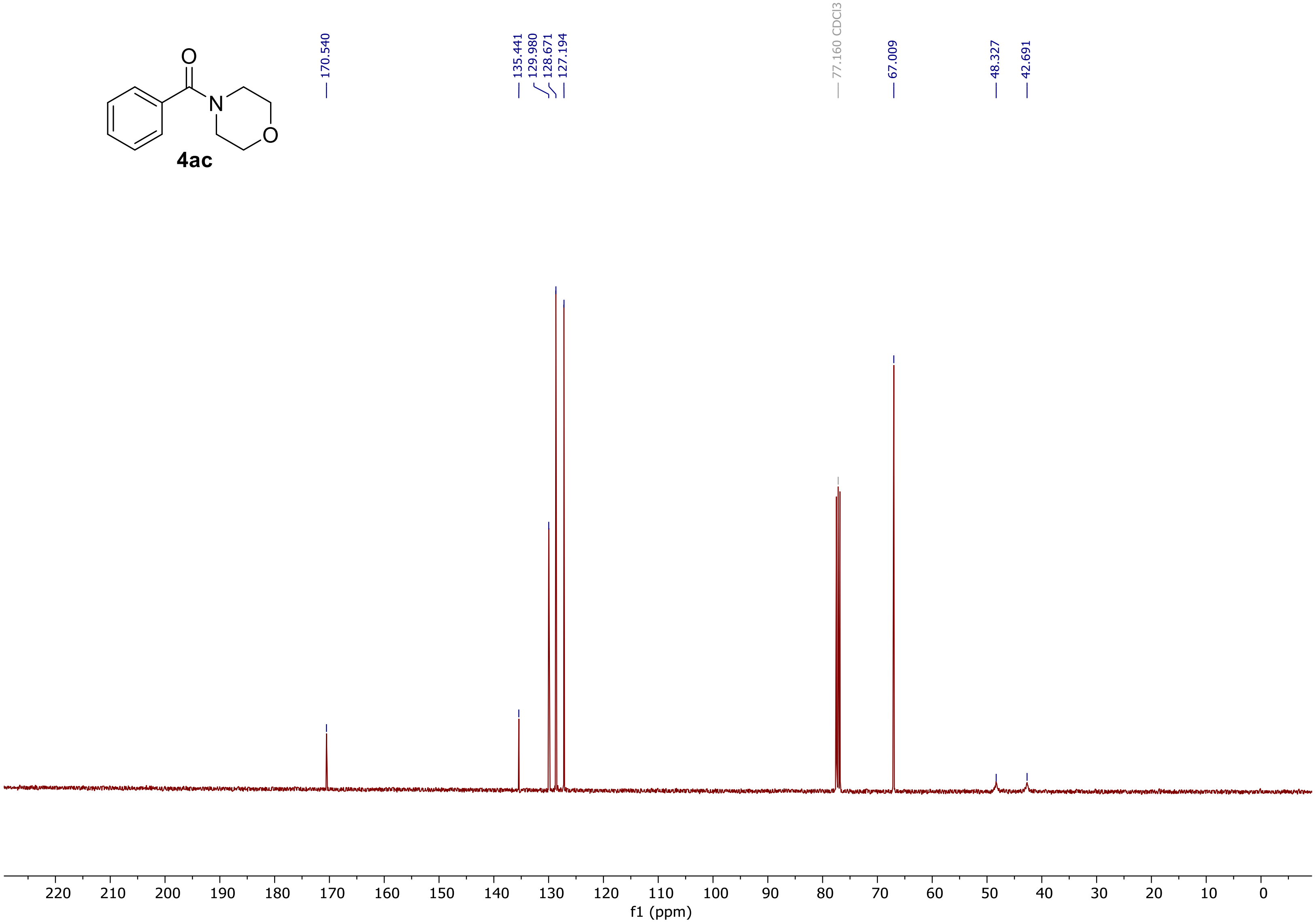

5.59 ^{1}H NMR spectrum 4ad (400 MHz, $CDCl_3$)

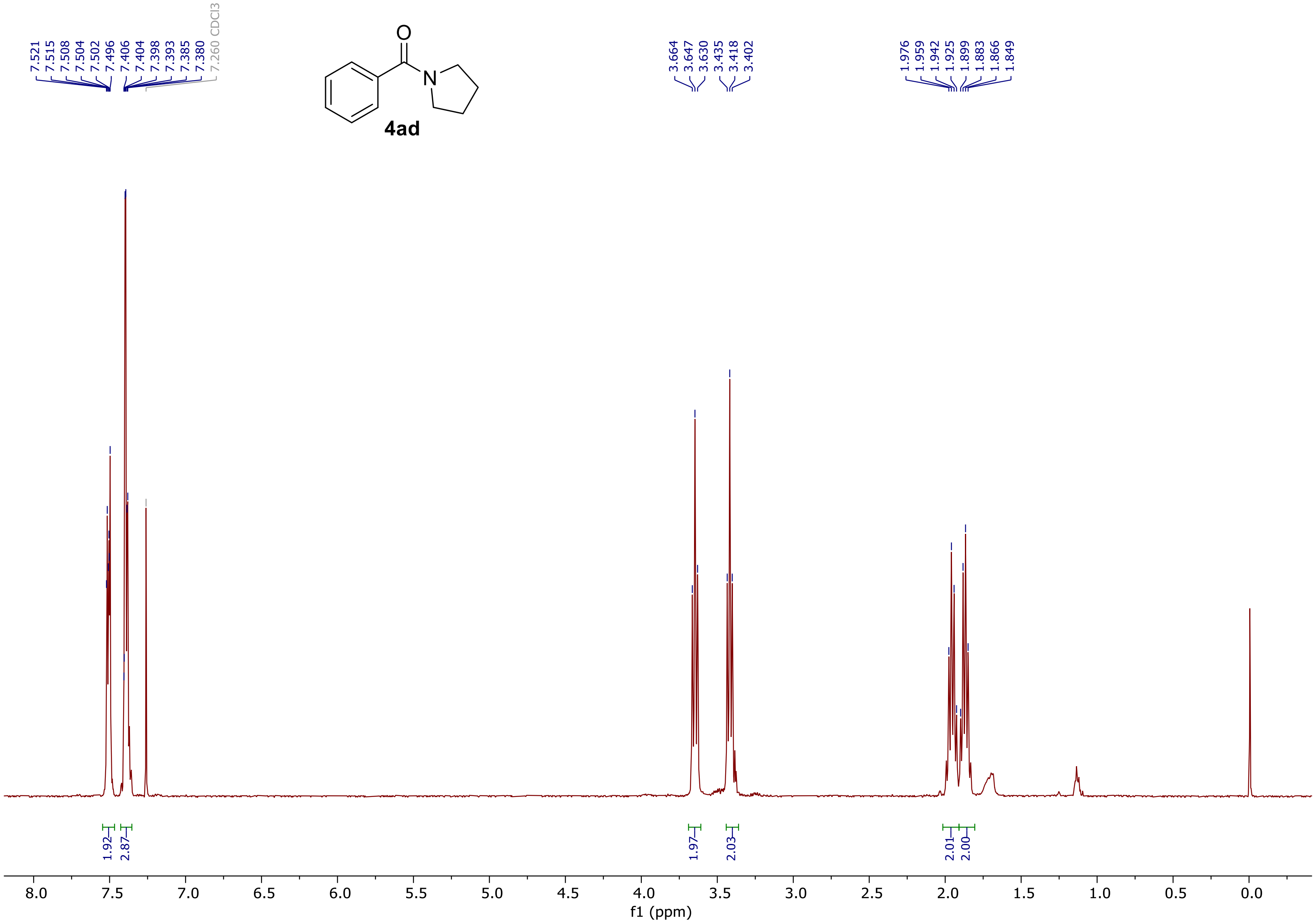

5.60 $^{13}C\{^{1}H\}$ NMR spectrum 4ad (101 MHz, $CDCl_3$)

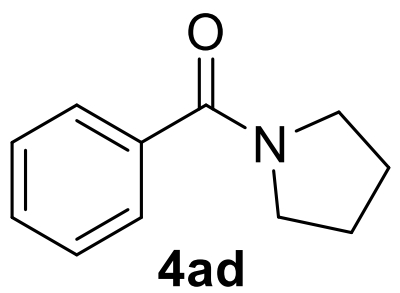

4ad

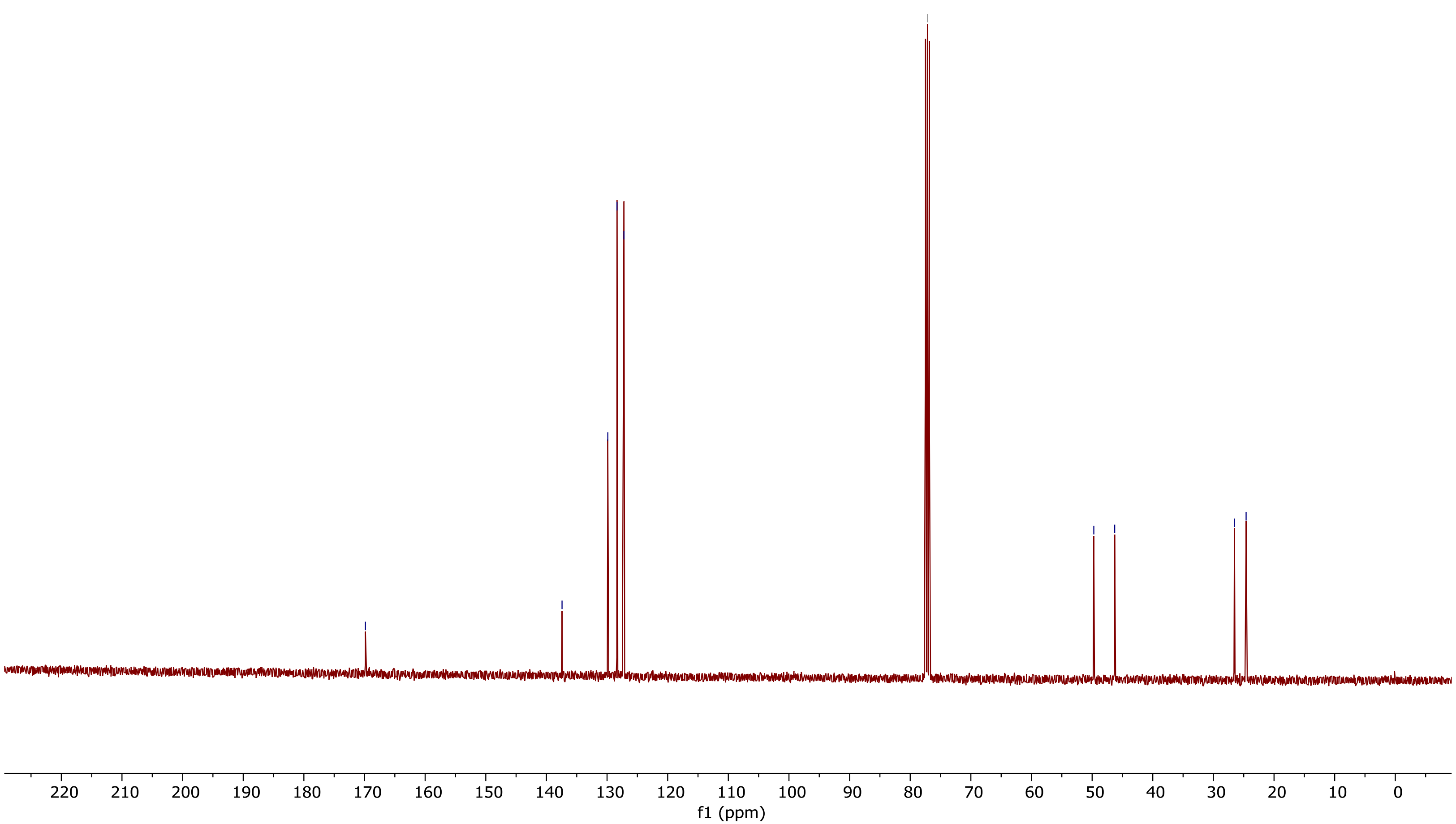

5.61 ^{1}H NMR spectrum 4ae (500 MHz, $CDCl_3$)

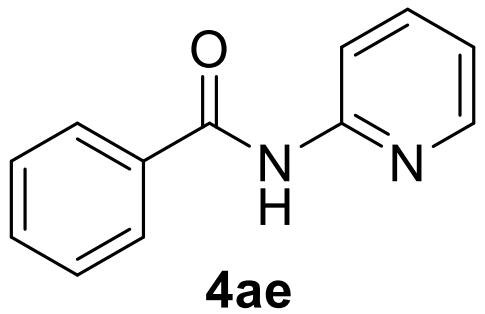

4ae

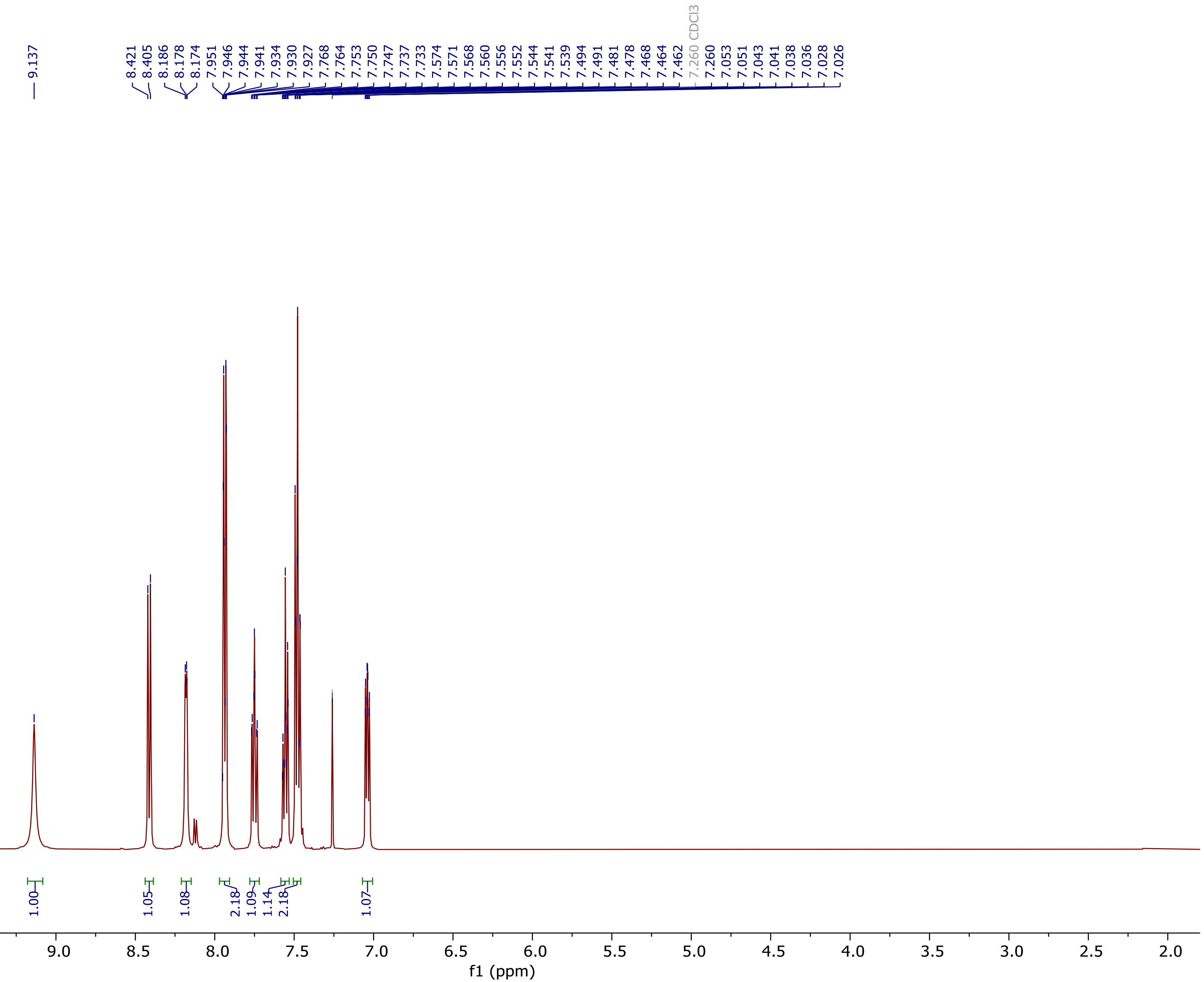

5.62 $^{13}C\{^{1}H\}$ NMR spectrum 4ae (127 MHz, $CDCl_3$)

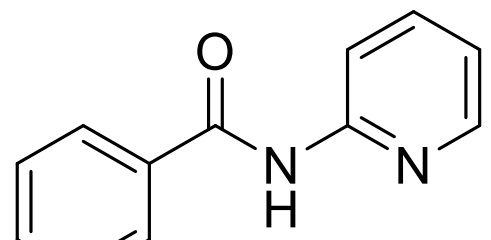

4ae

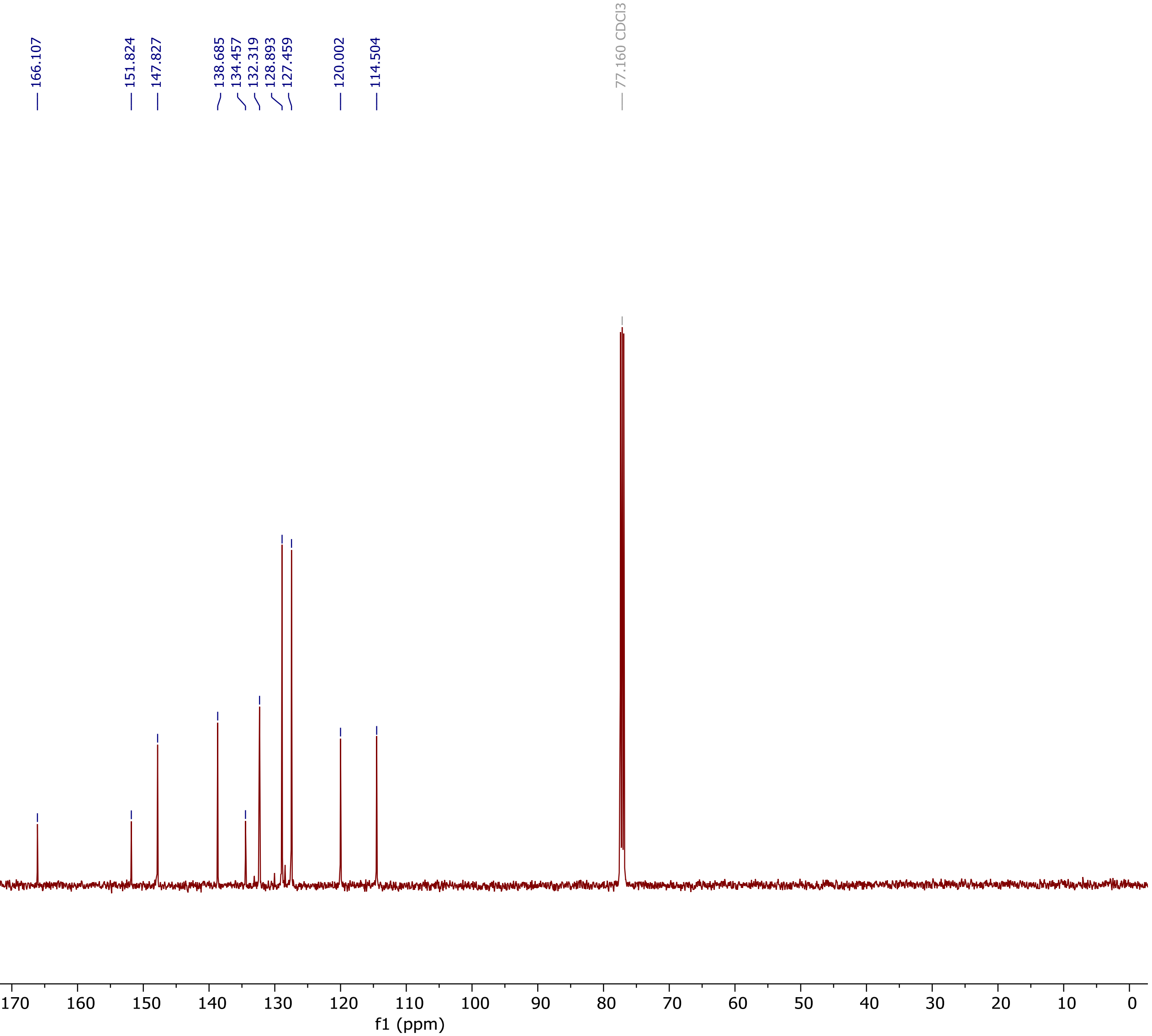

5.63 ^{1}H NMR spectrum 4af (400 MHz, DMSO-d$_6$)

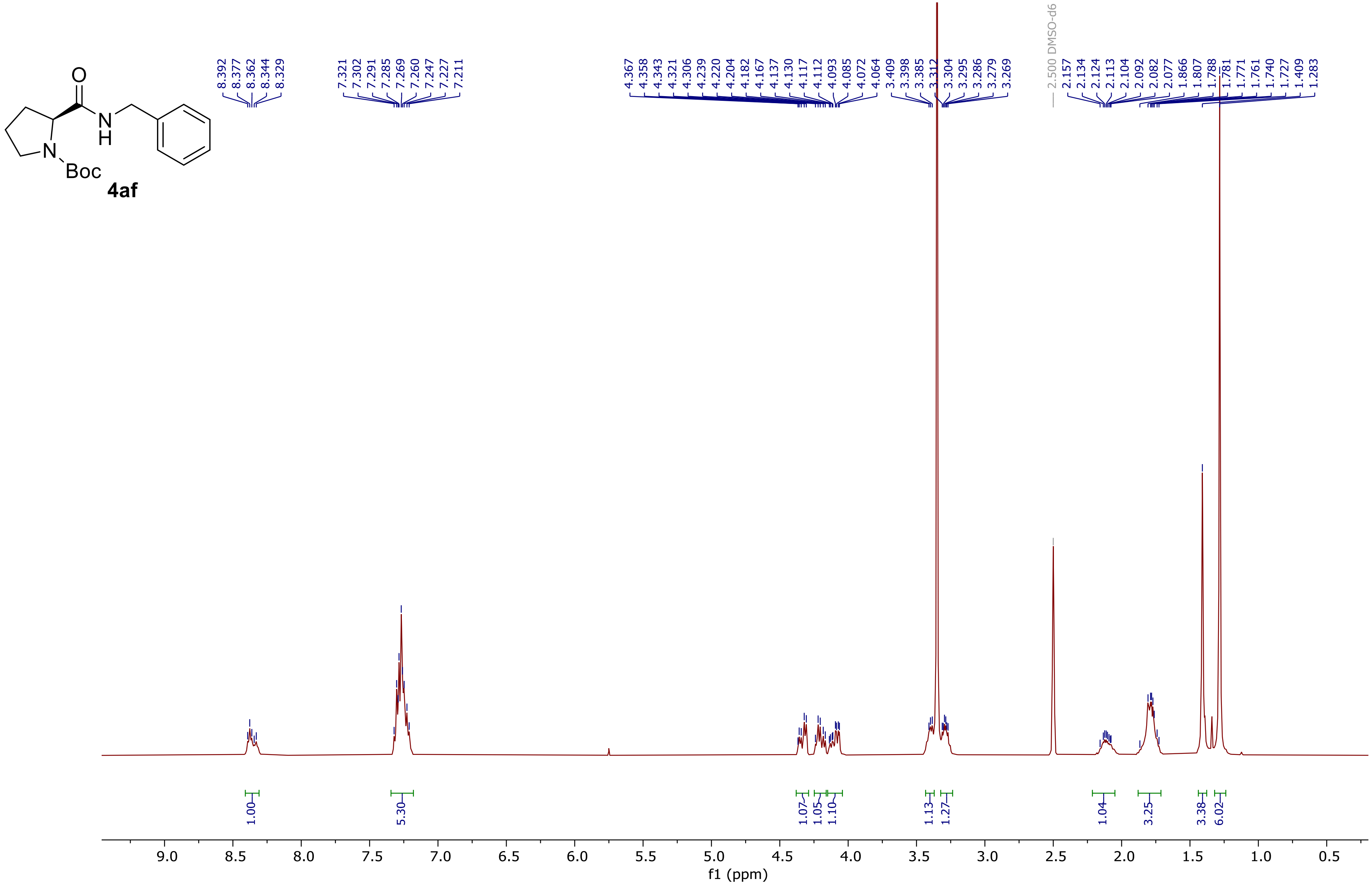

5.64 $^{13}C\{^1H\}$ NMR spectrum 4af (101 MHz, DMSO-d$_6$)

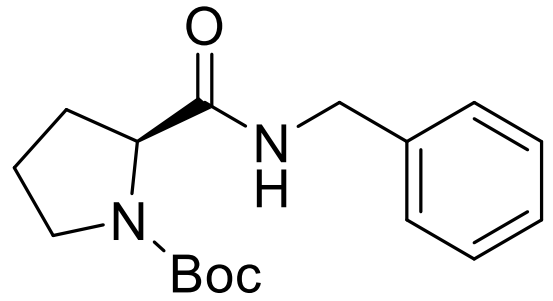

4af

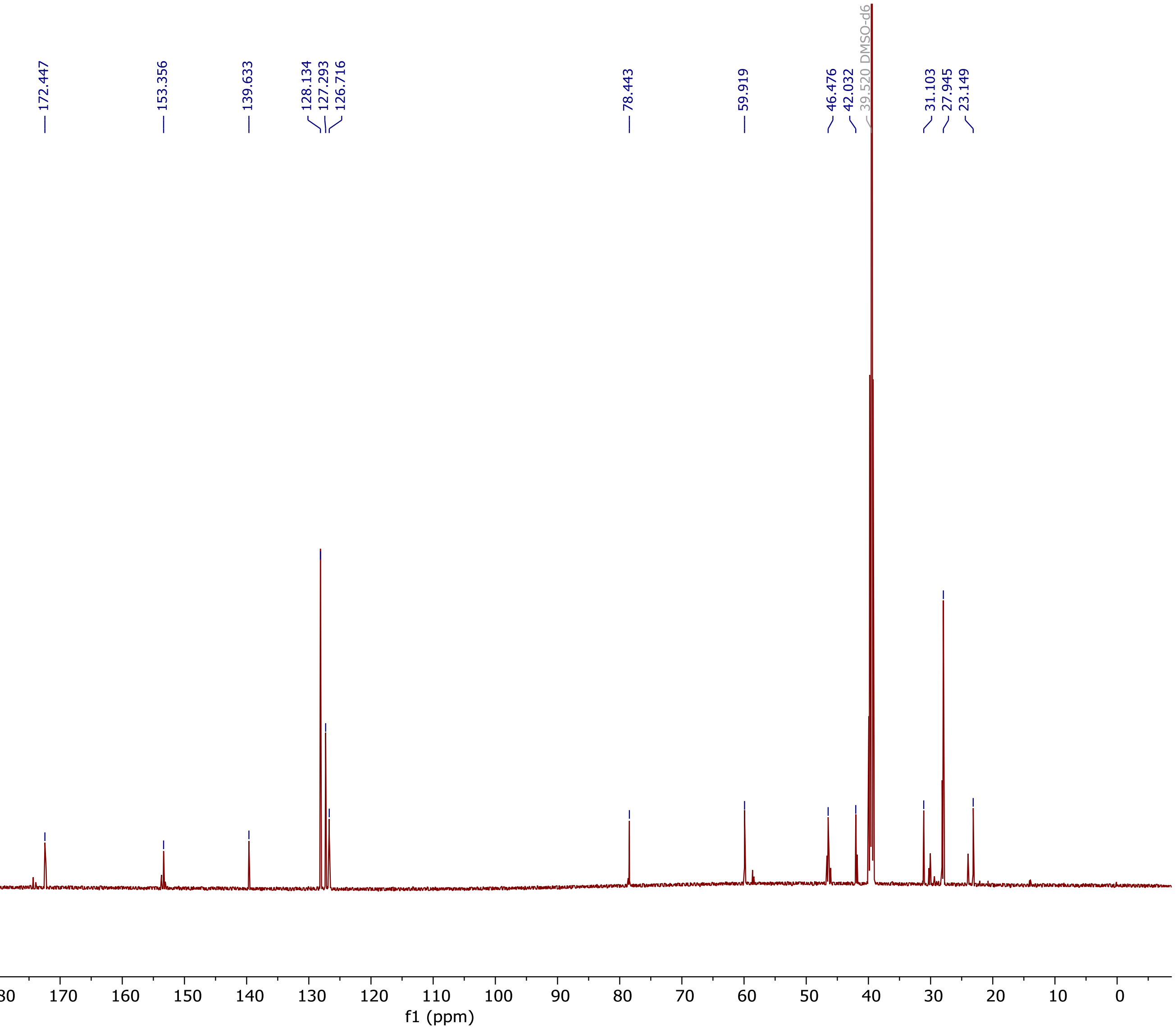

5.65 ^{1}H NMR spectrum 4ag (400 MHz, $CDCl_3$)

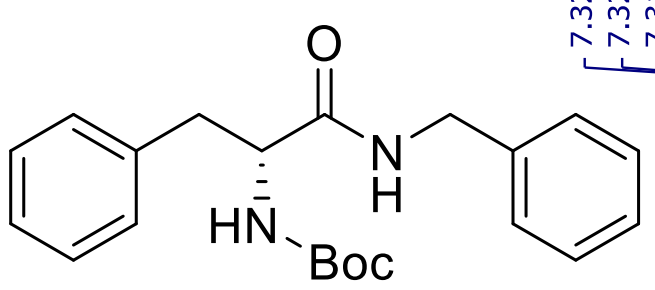

4ag

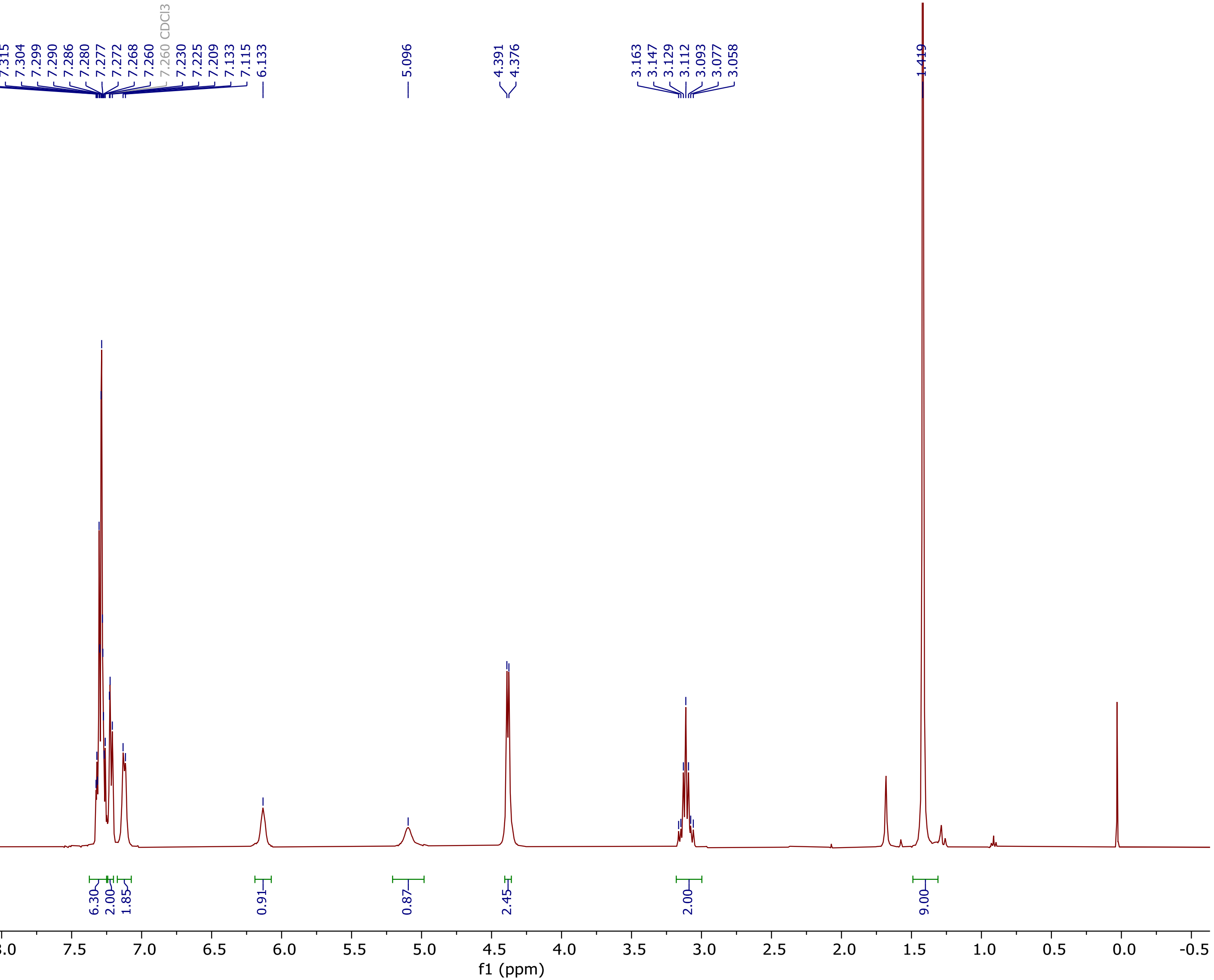

5.66 $^{13}C\{^{1}H\}$ NMR spectrum 4ag (101 MHz, $CDCl_3$)

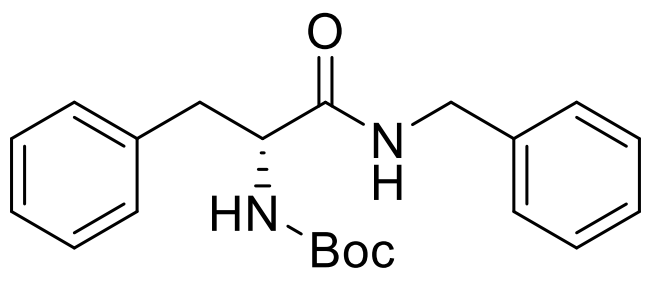

4ag

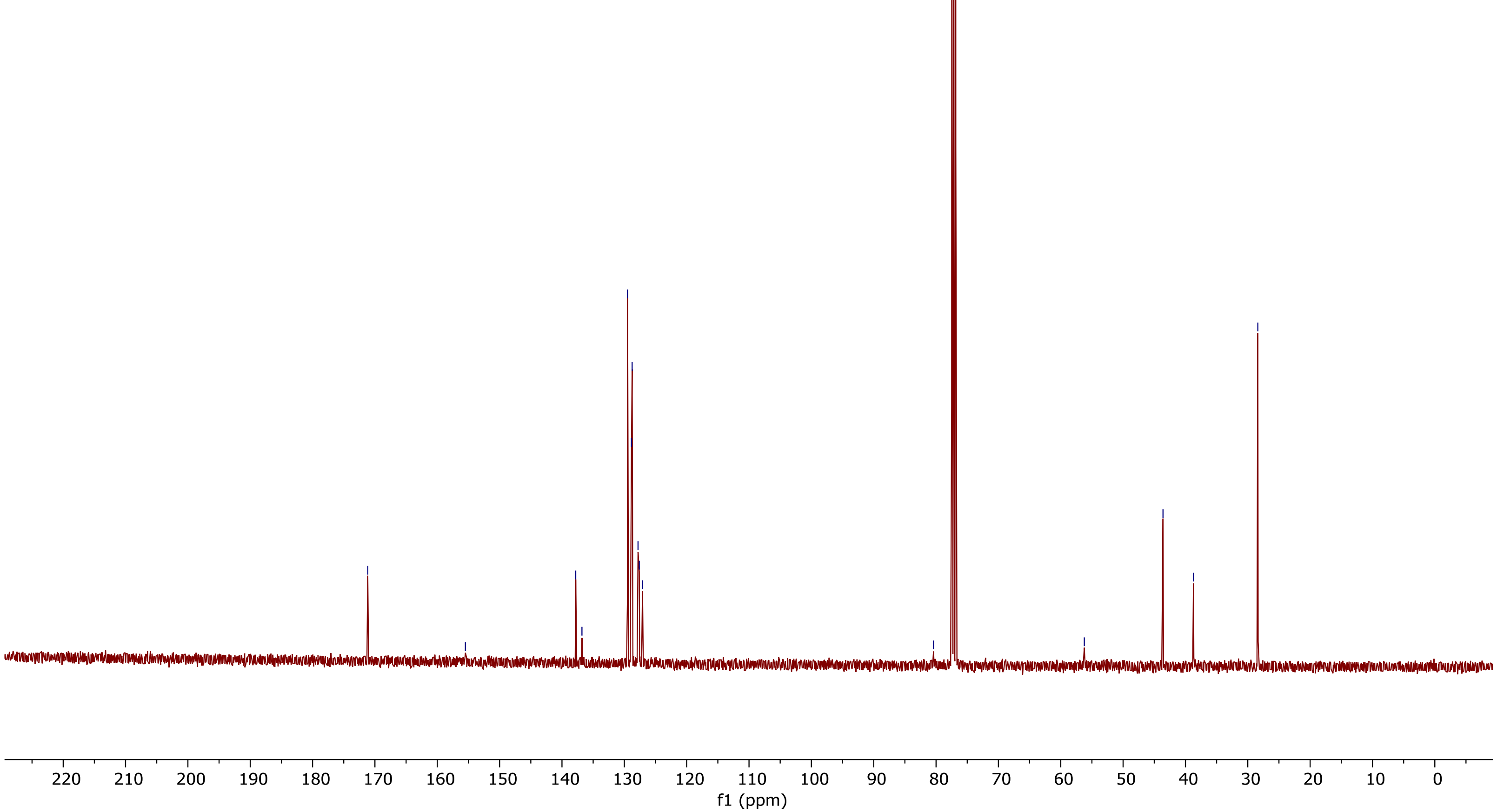

5.67 ^{1}H NMR spectrum 4ah (600 MHz, DMSO-d_6)

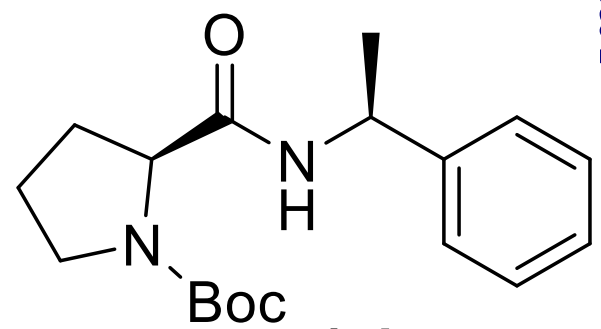

4ah

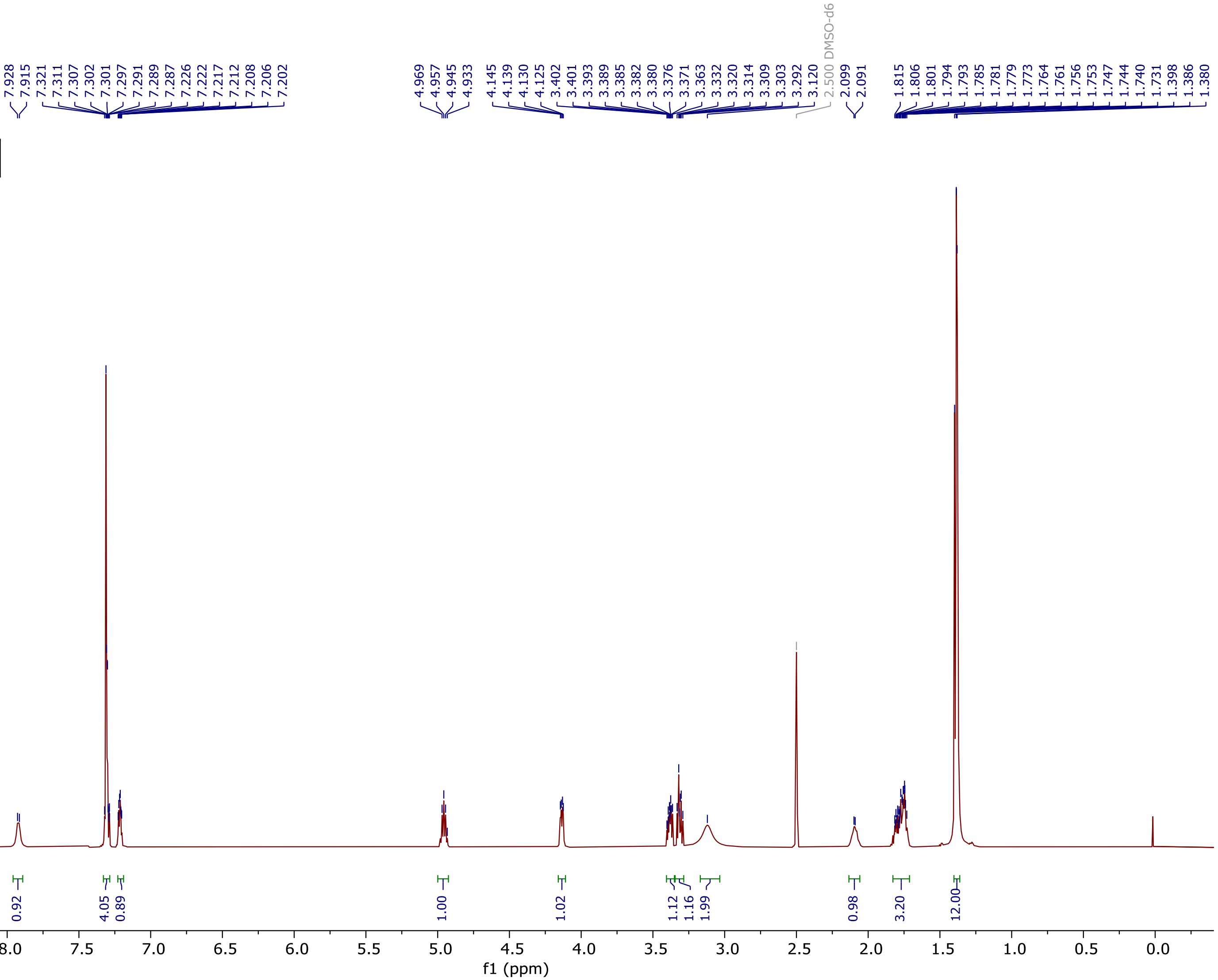

5.68 $^{13}C\{^{1}H\}$ NMR spectrum 4ah (151 MHz, DMSO-d$_6$)

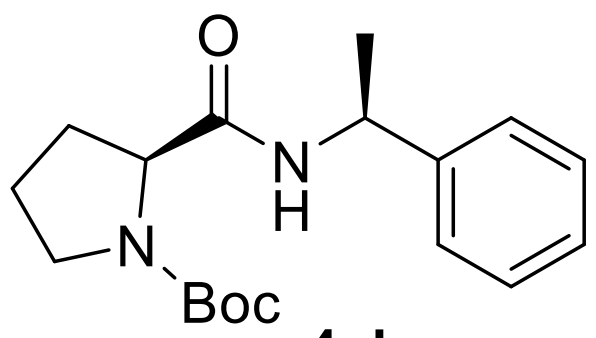
4ah

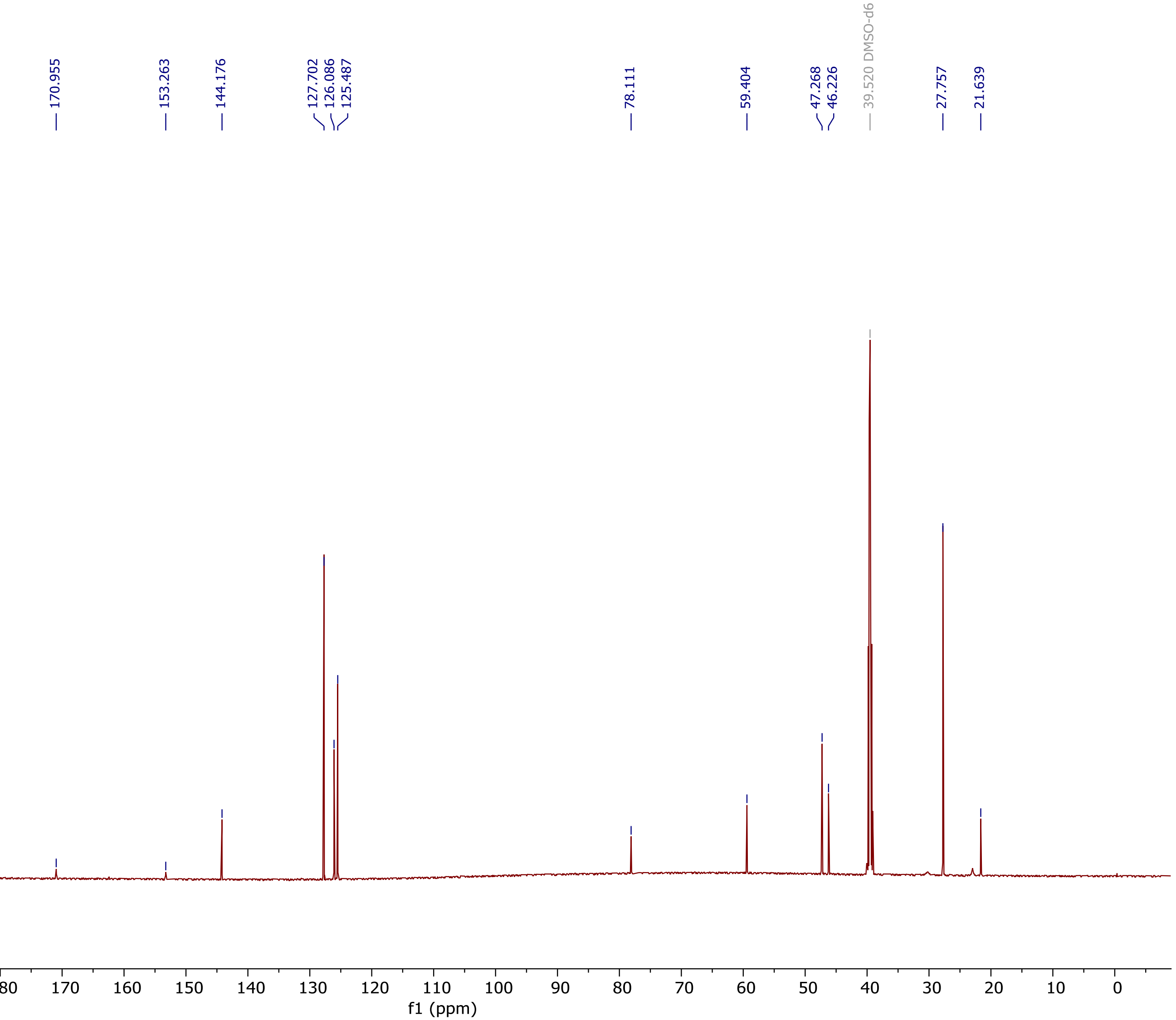

5.69 ^{1}H NMR spectrum 4ai (400 MHz, DMSO-d$_6$)

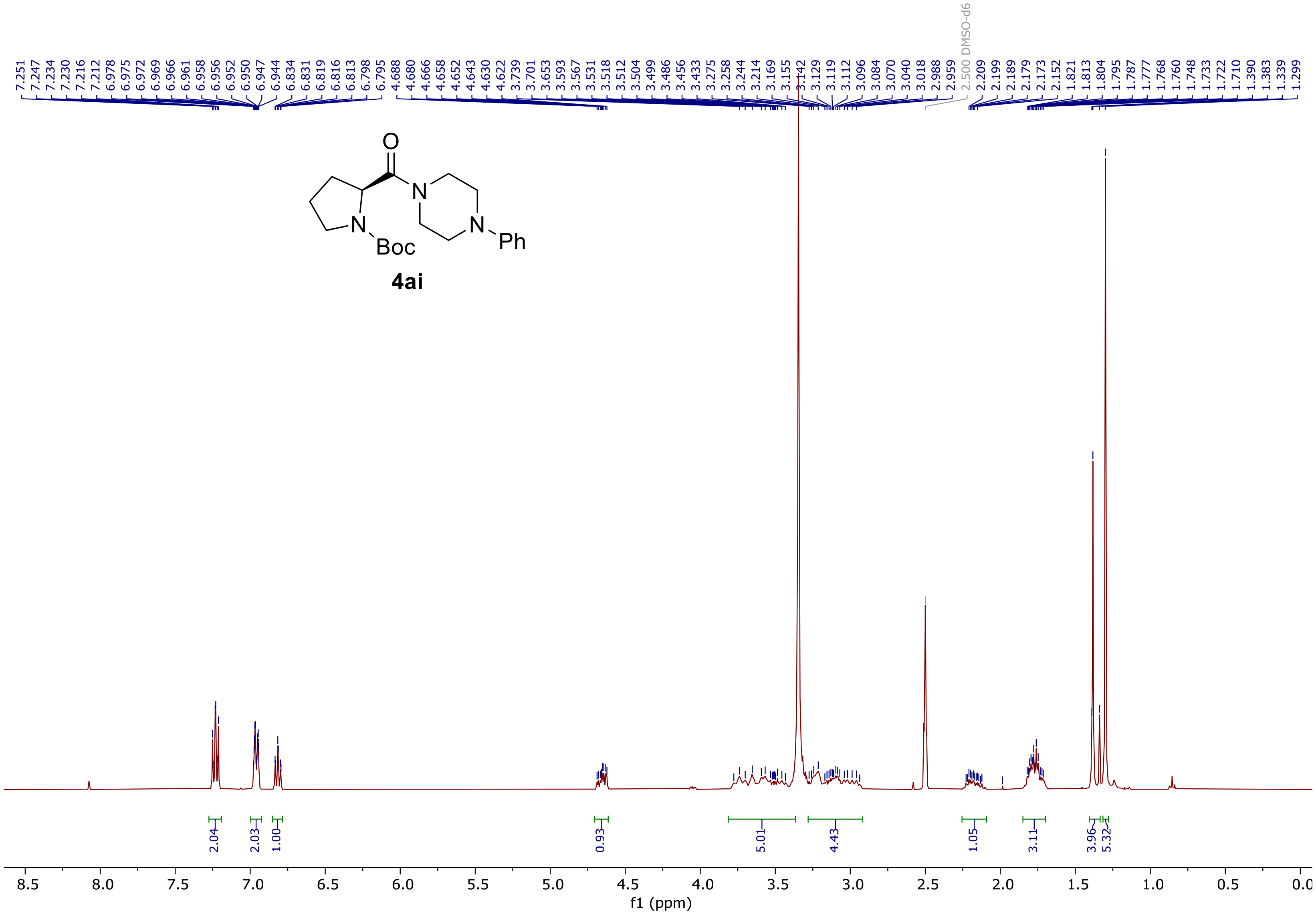

5.70 $^{13}C\{^{1}H\}$ NMR spectrum 4ai (101 MHz, DMSO-d_6)

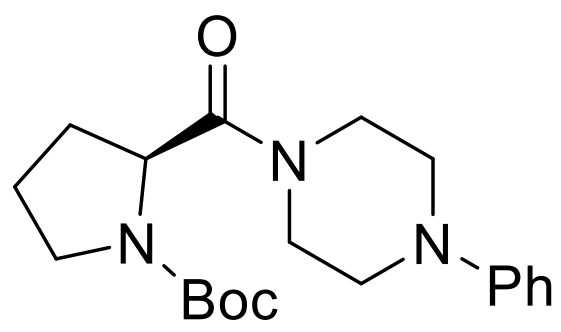

4ai

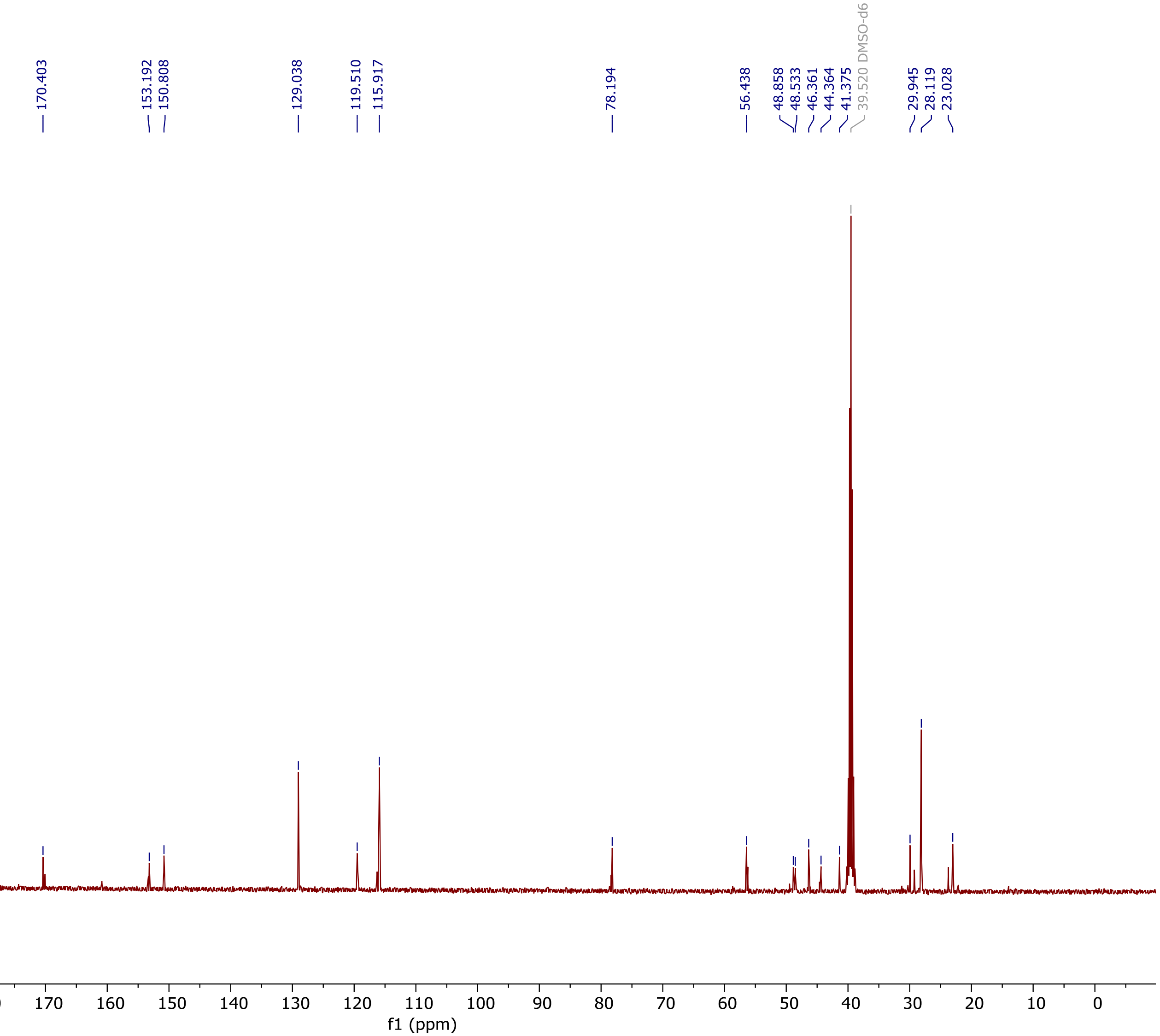

5.71 ^{1}H NMR spectrum 4aj (400 MHz, $CDCl_3$)

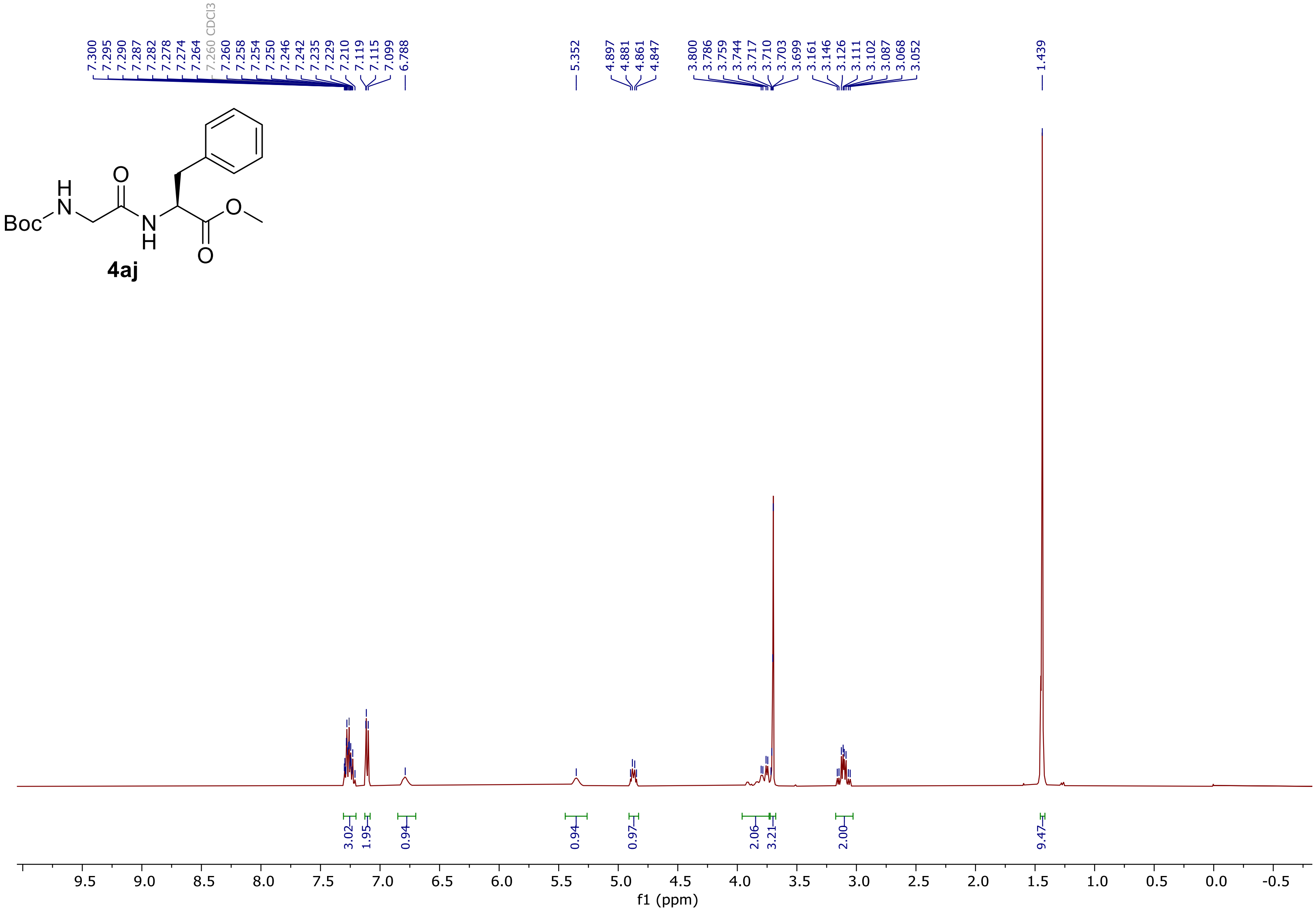

5.72 $^{13}C\{^{1}H\}$ NMR spectrum 4aj (101 MHz, $CDCl_3$)

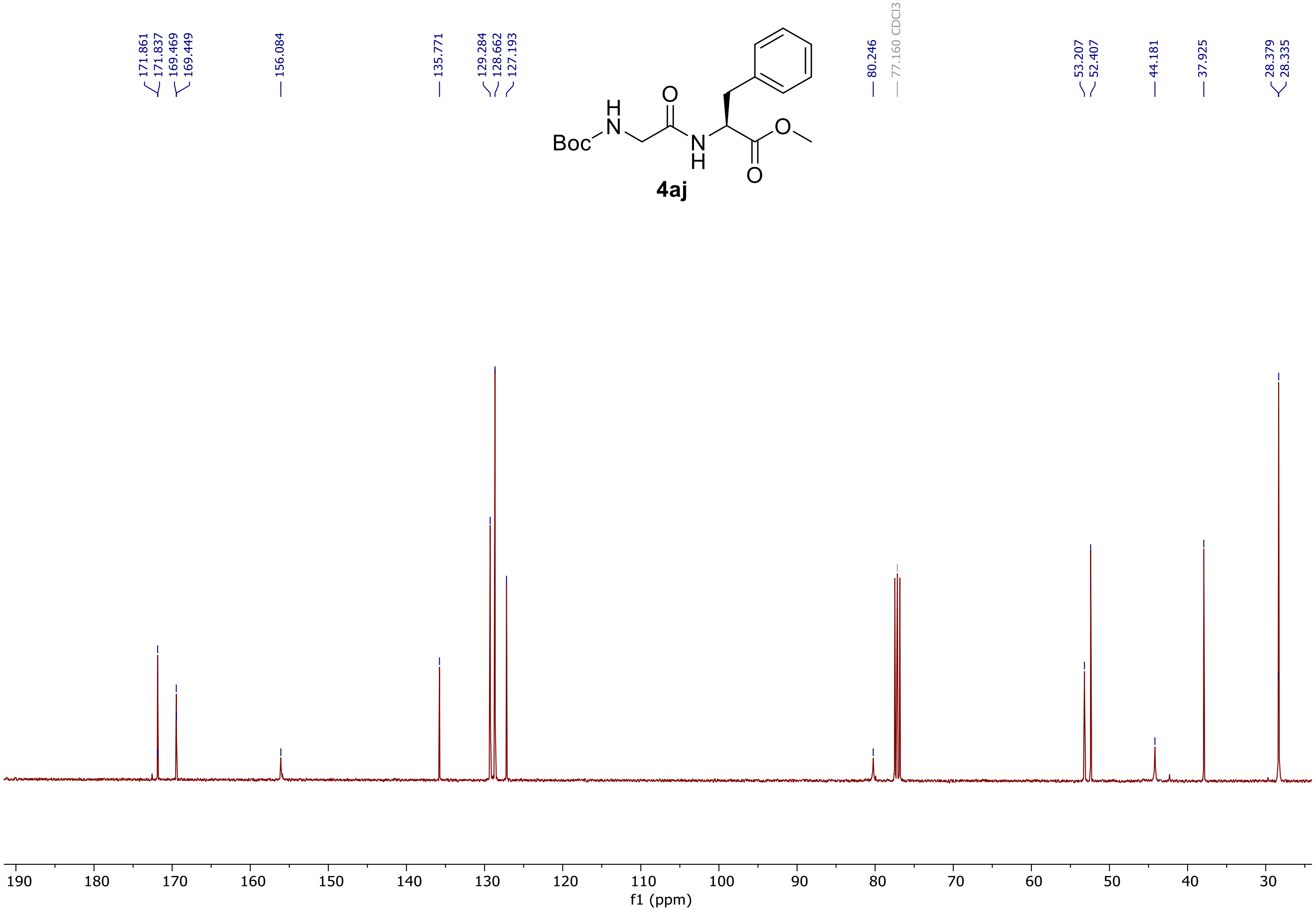

5.73 ^{1}H NMR spectrum 4ak (400 MHz, $CDCl_3$)

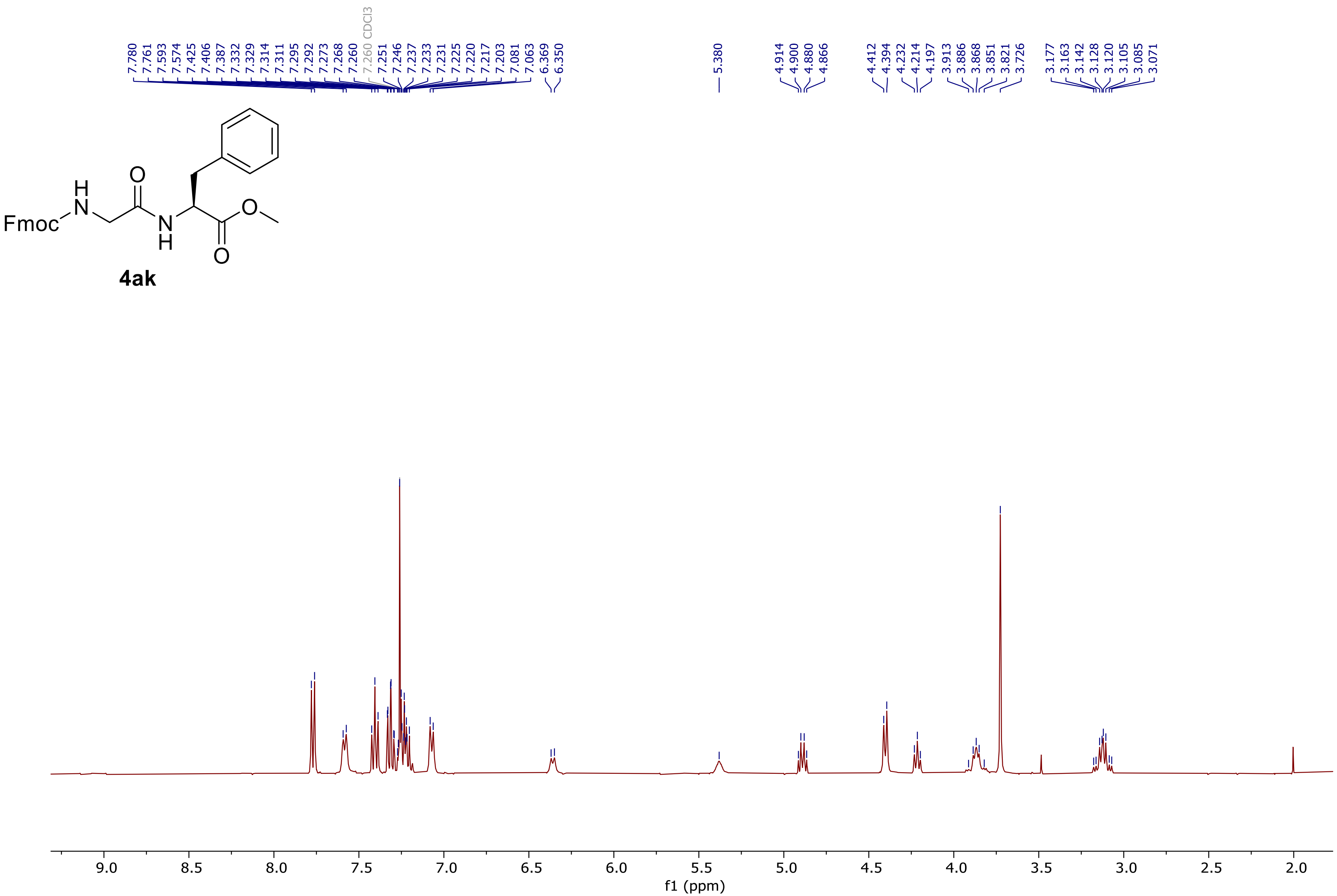

4ak

5.74 $^{13}C\{^1H\}$ NMR spectrum 4ak (101 MHz, $CDCl_3$)

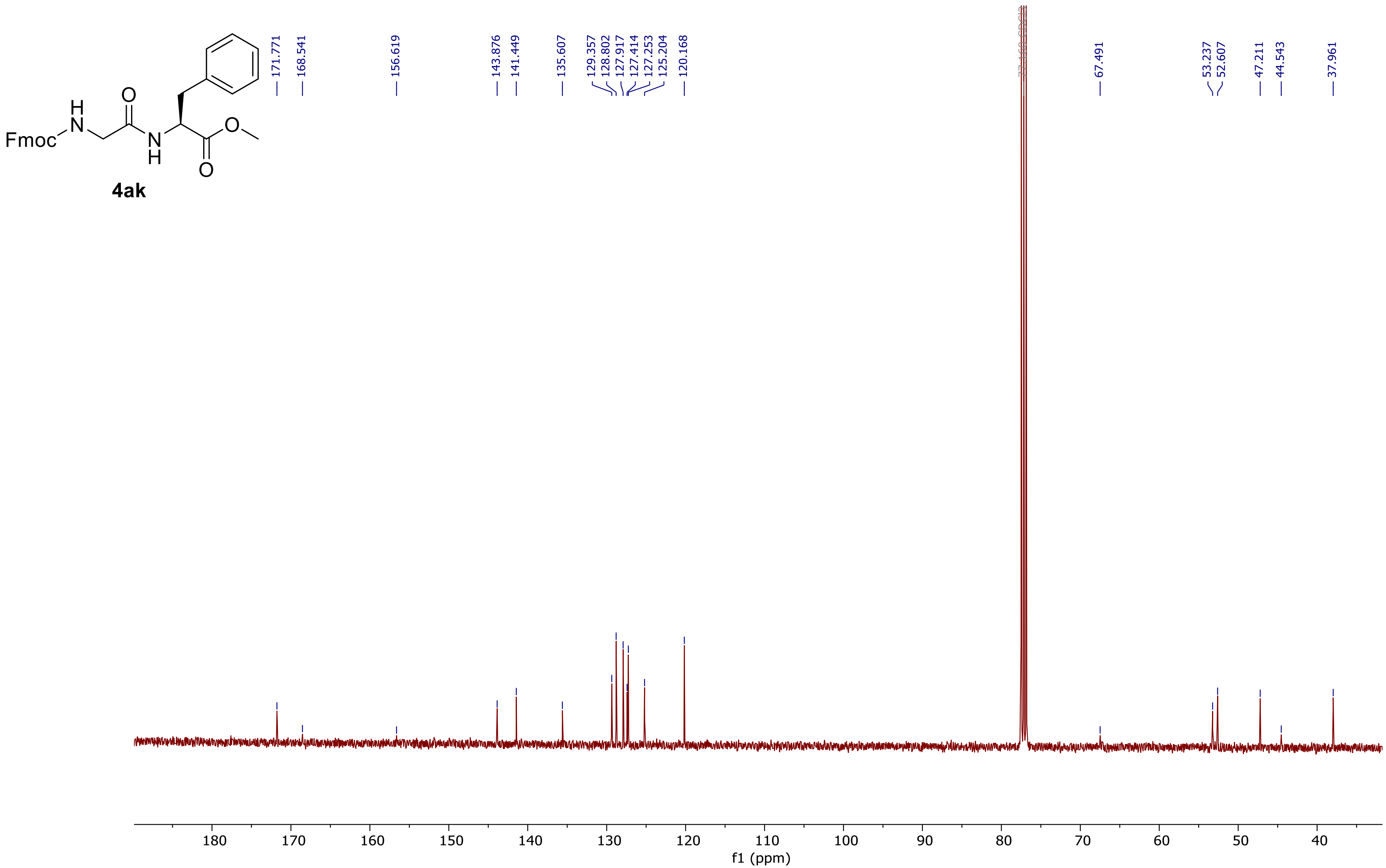

5.75 ^{1}H NMR spectrum 4al (400 MHz, DMSO-d$_6$)

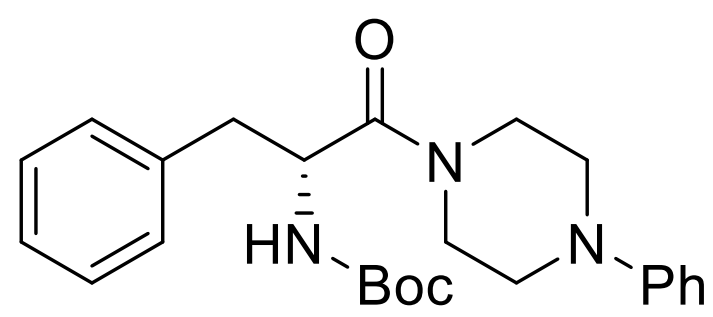

4al

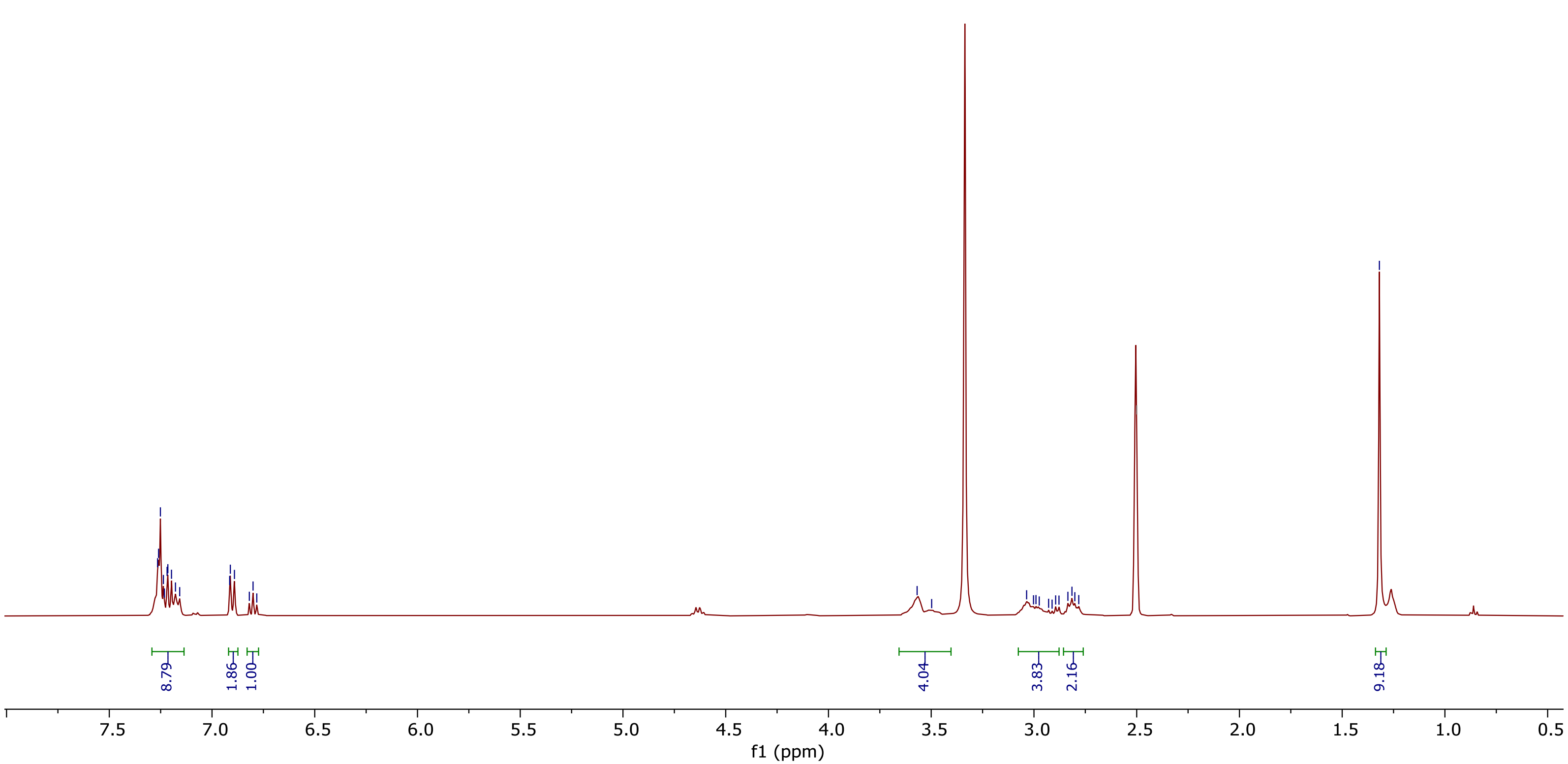

5.76 $^{13}C\{^{1}H\}$ NMR spectrum 4al (101 MHz, DMSO-d$_6$)

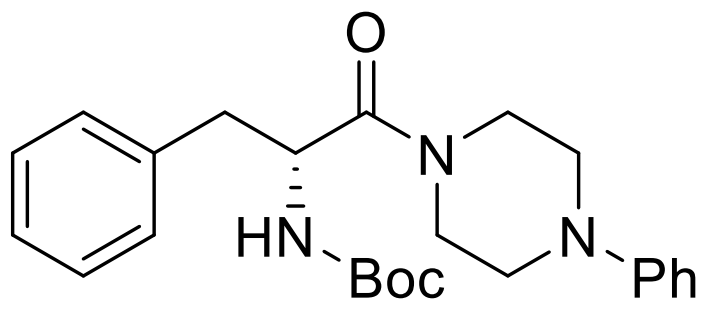

4al

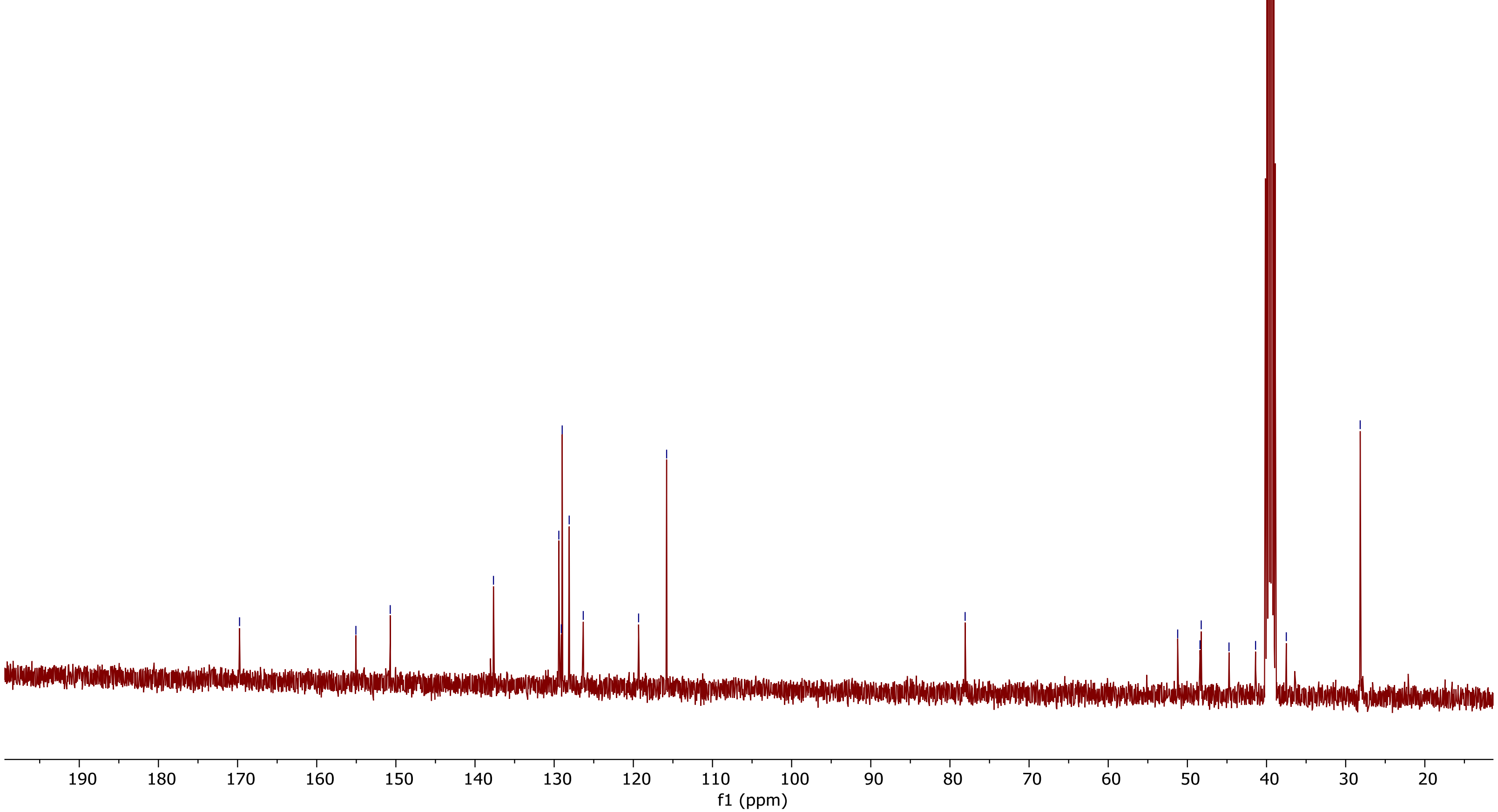

5.77 ^{1}H NMR spectrum 4am (400 MHz, $CDCl_3$)

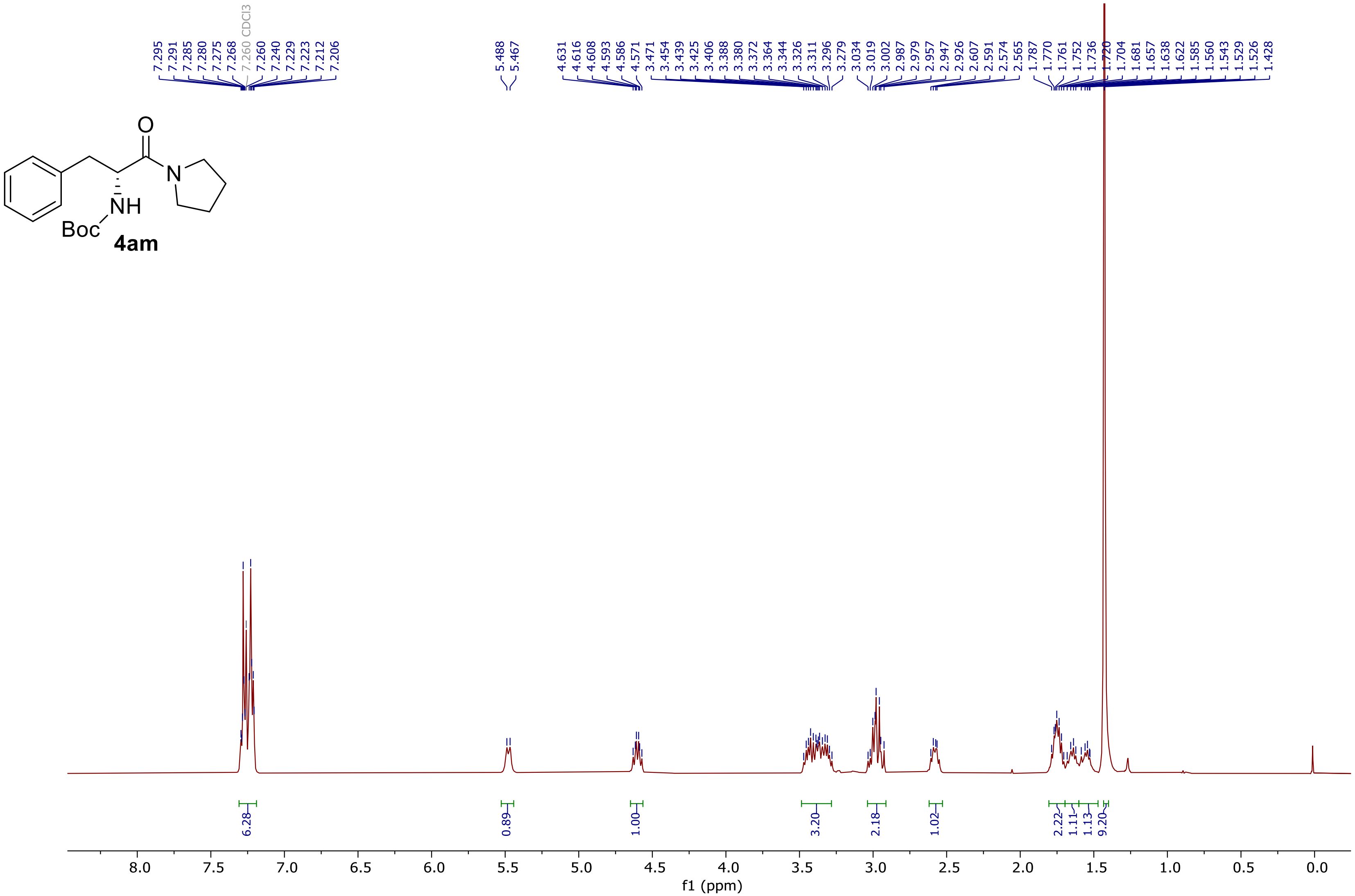

4am

5.78 $^{13}C\{^{1}H\}$ NMR spectrum 4am (101 MHz, $CDCl_3$)

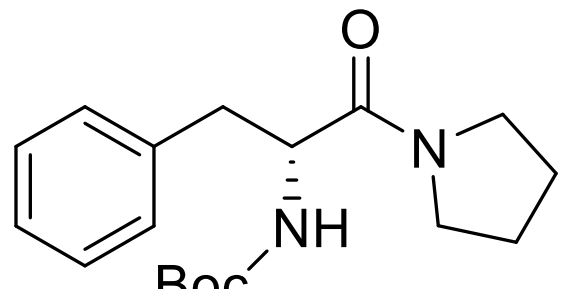

4am

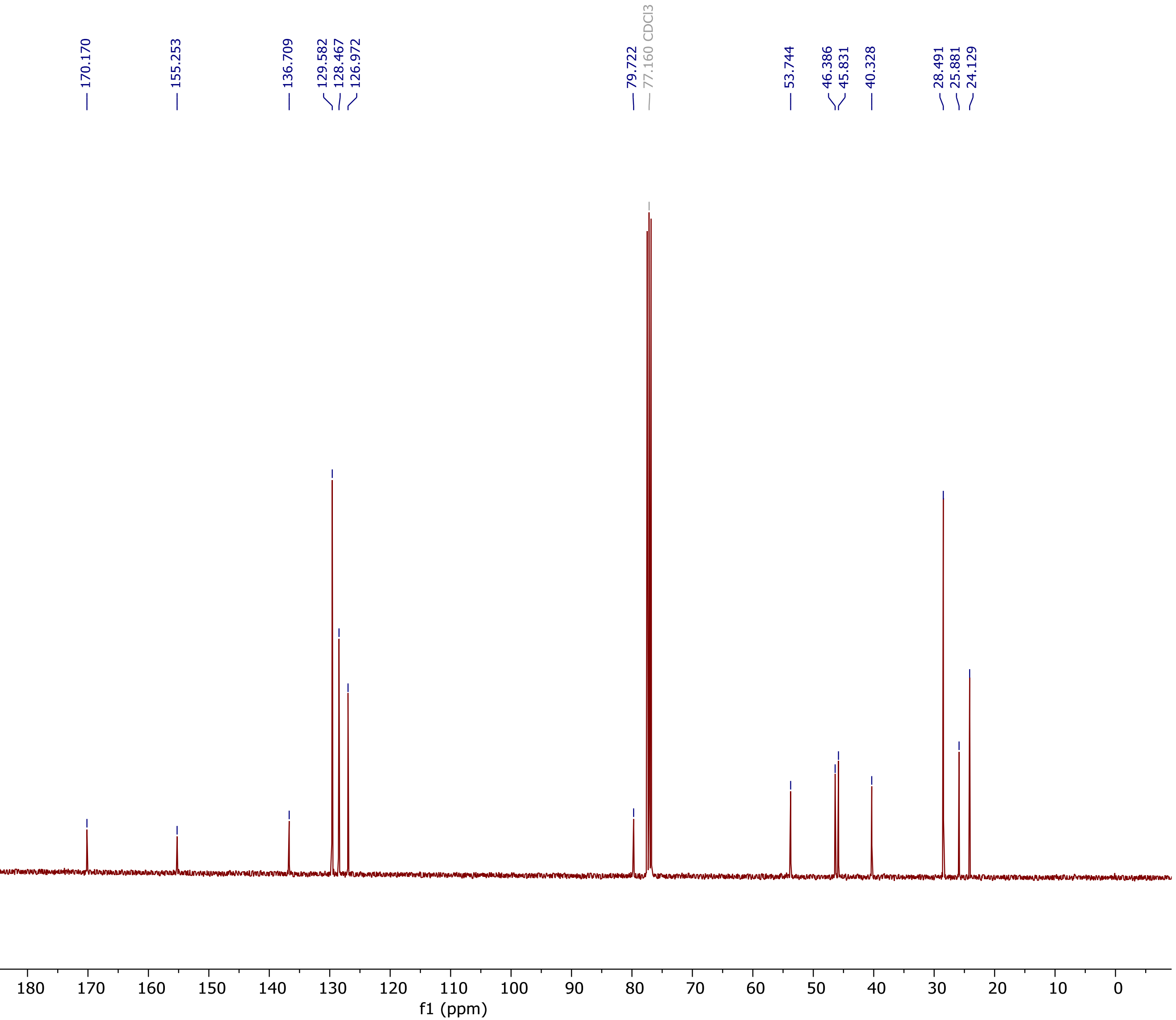

5.79 ^{1}H NMR spectrum 4an (400 MHz, DMSO-d$_6$)

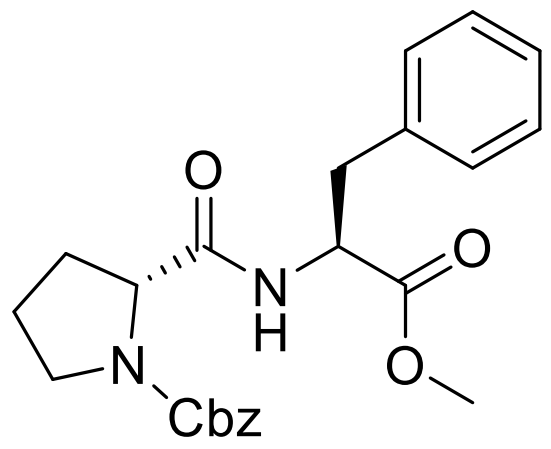

4an

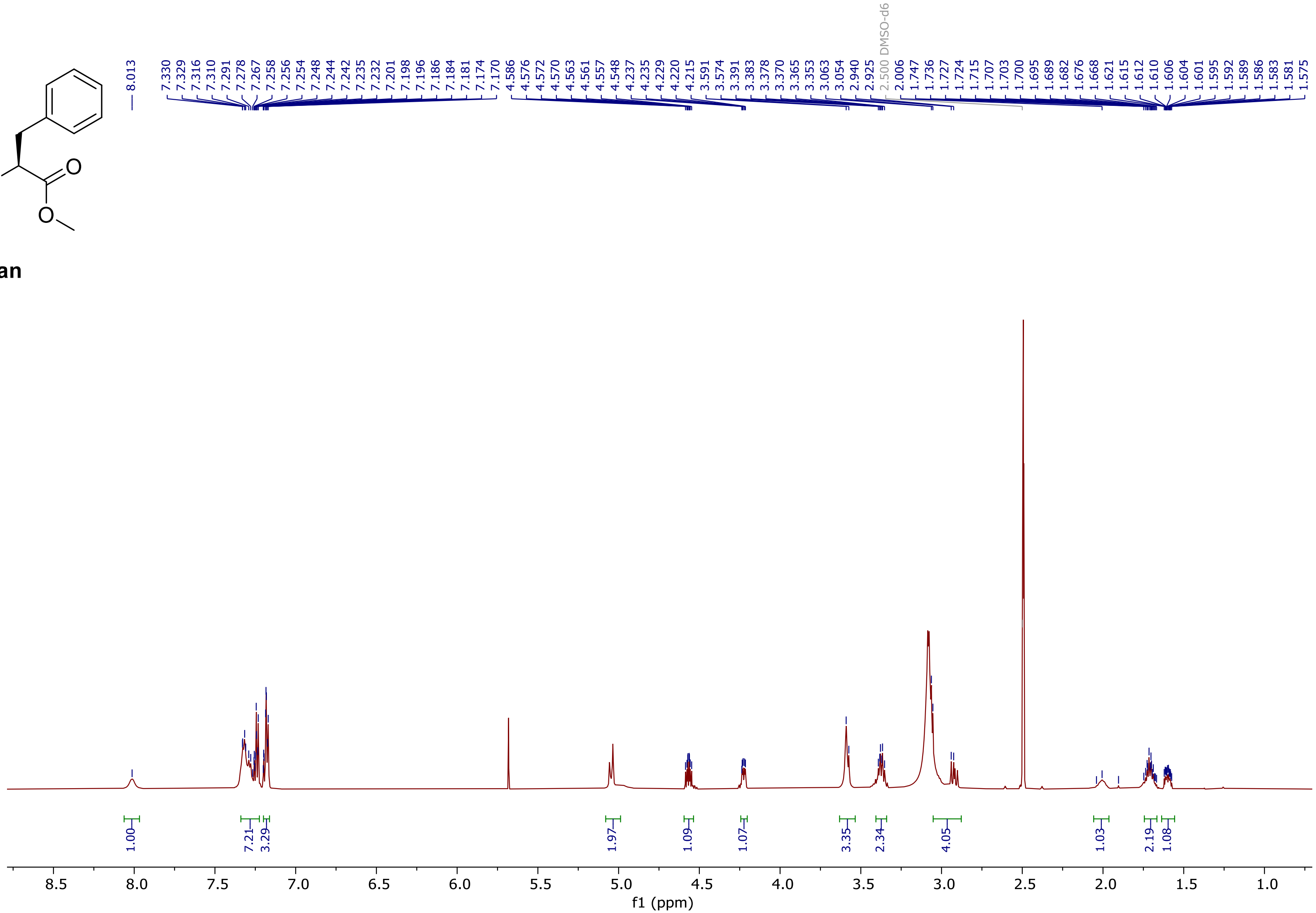

5.80 $^{13}C\{^1H\}$ NMR spectrum 4an (101 MHz, DMSO-d$_6$)

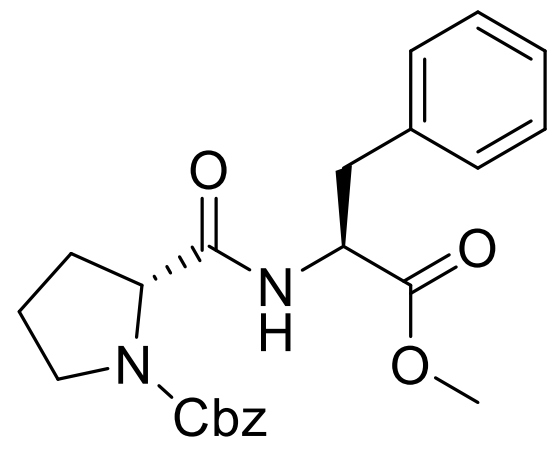

4an

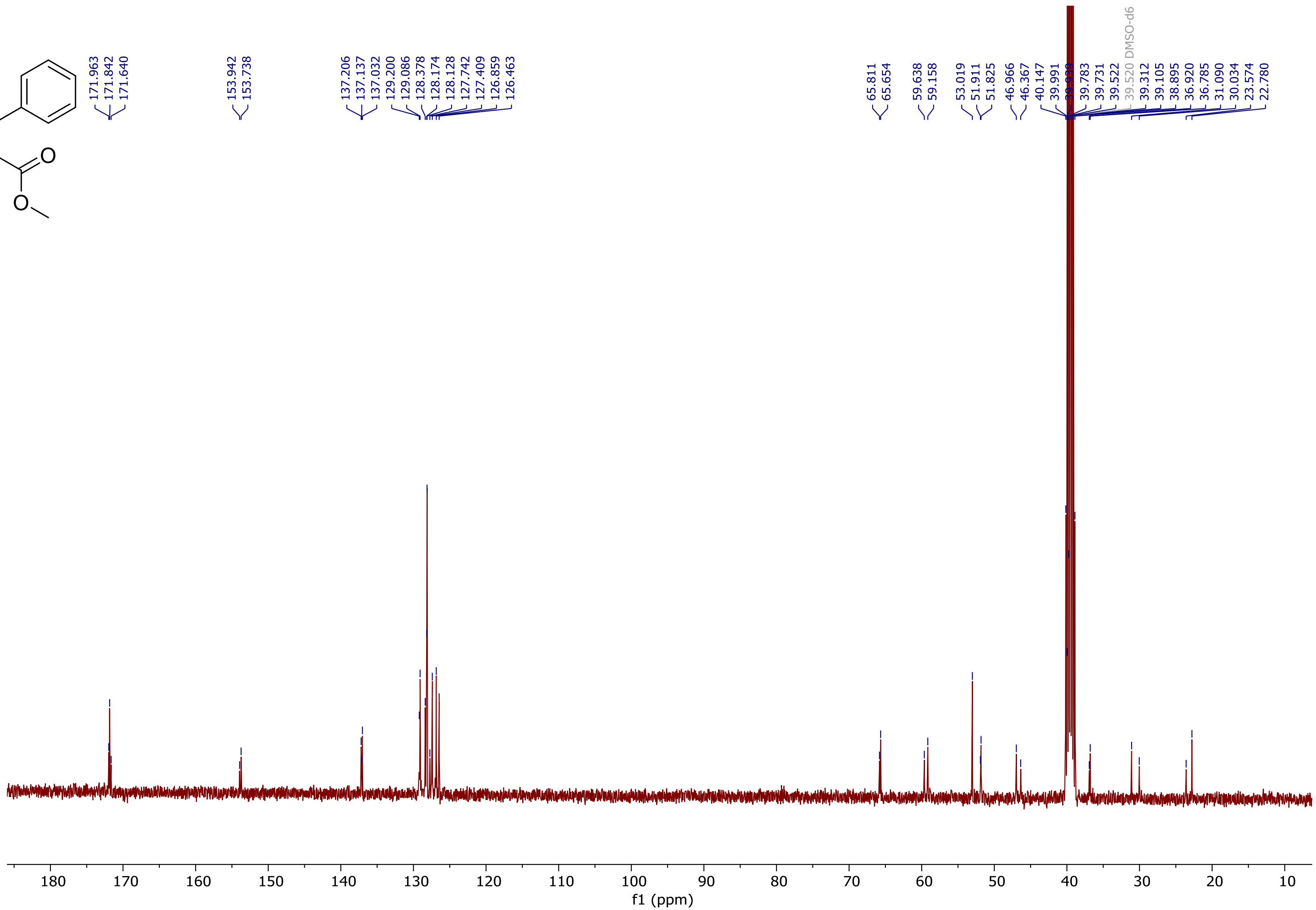

5.81 ^{1}H NMR spectrum 4ao (400 MHz, $CDCl_3$)

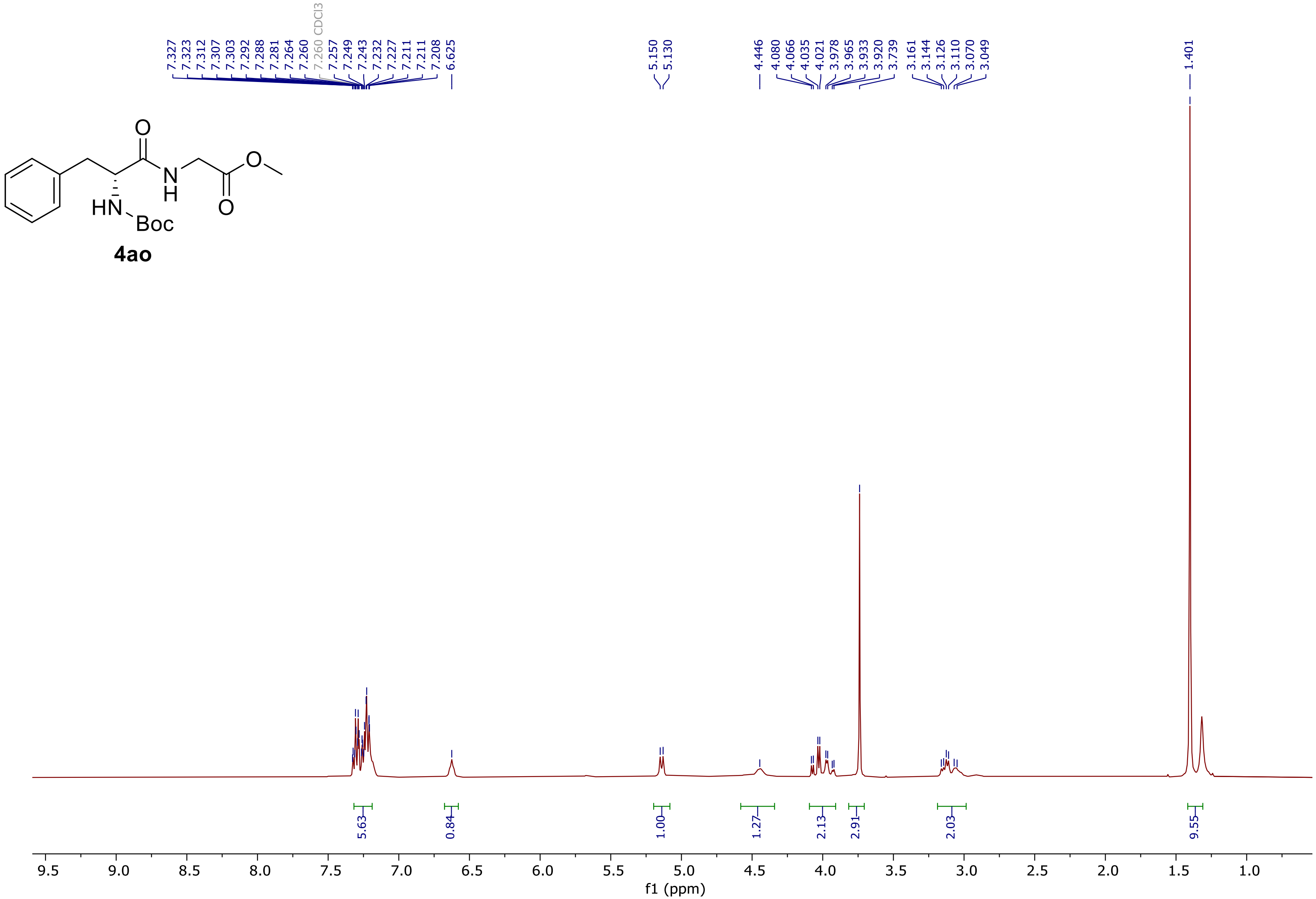

5.82 $^{13}C\{^1H\}$ NMR spectrum 4ao (101 MHz, $CDCl_3$)

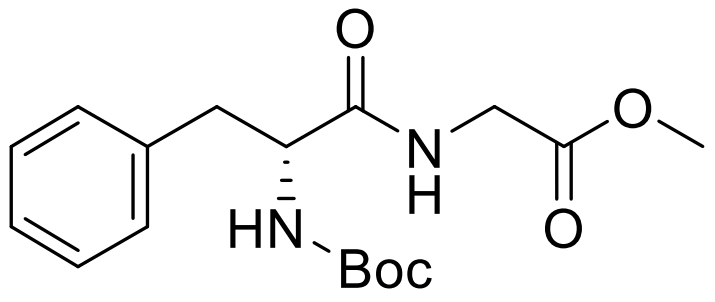

4ao

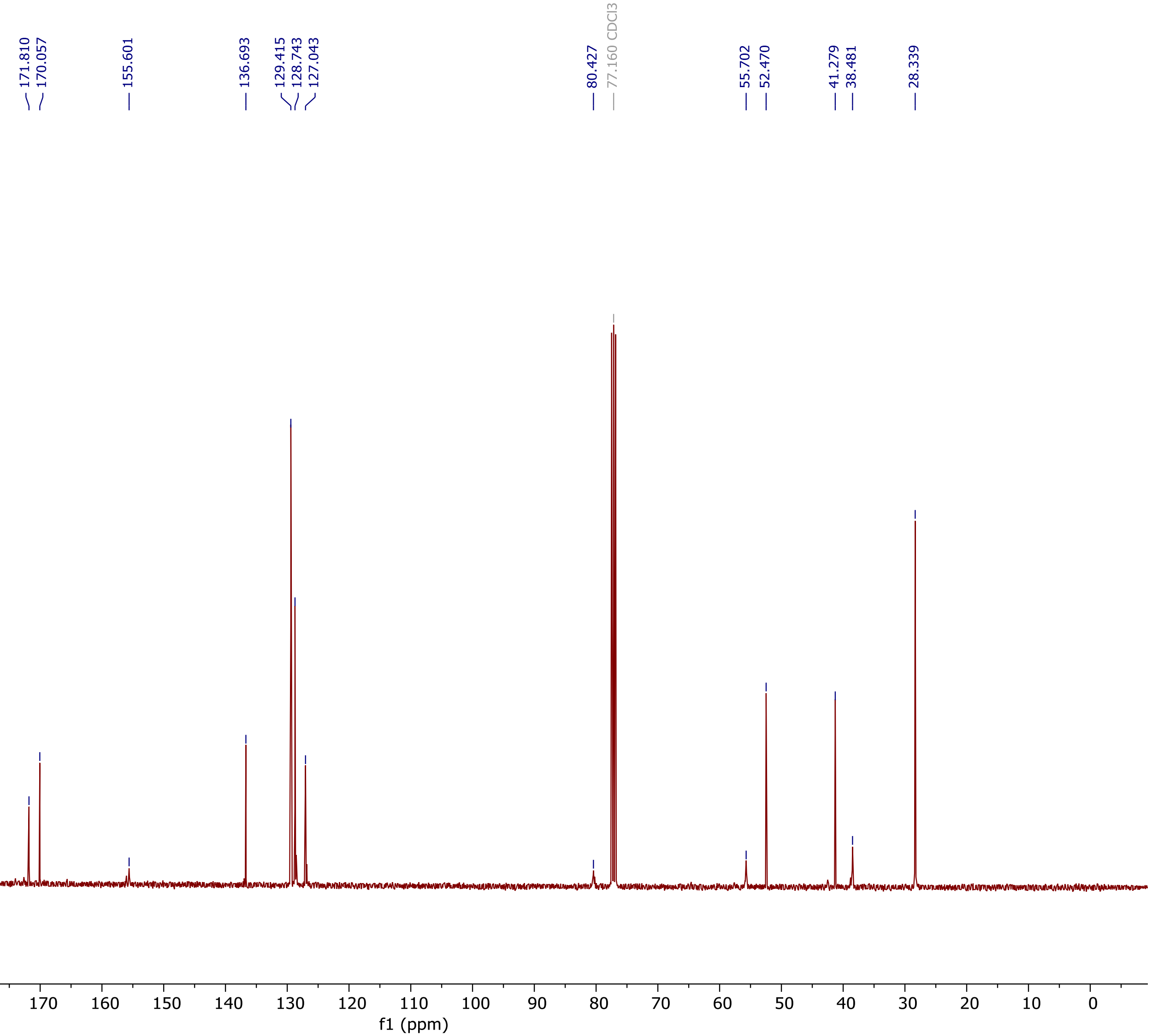

5.83 ^{1}H NMR spectrum 4ap (600 MHz, DMSO-d$_6$)

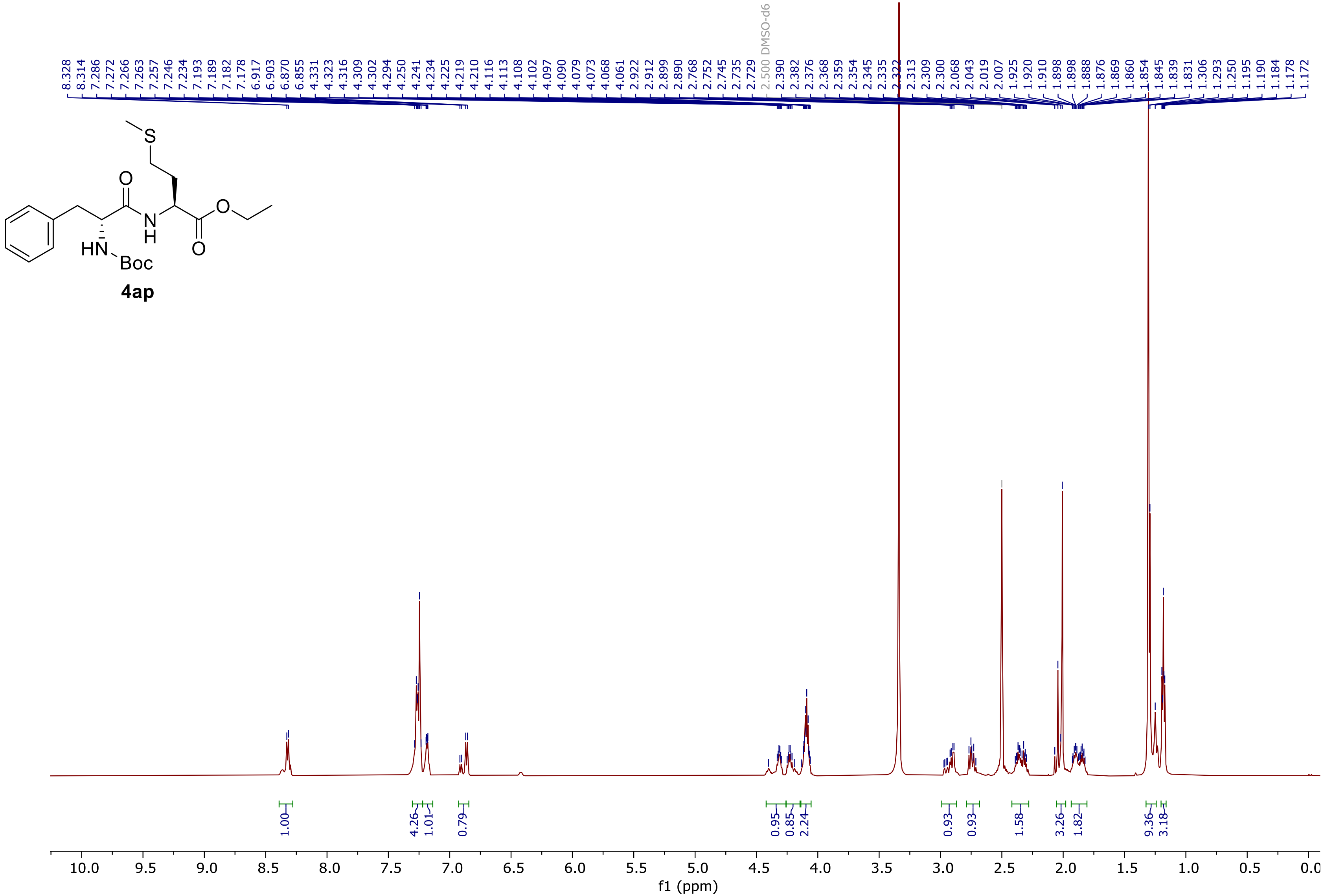

5.84 $^{13}C\{^1H\}$ NMR spectrum 4ap (151 MHz, DMSO-d$_6$)

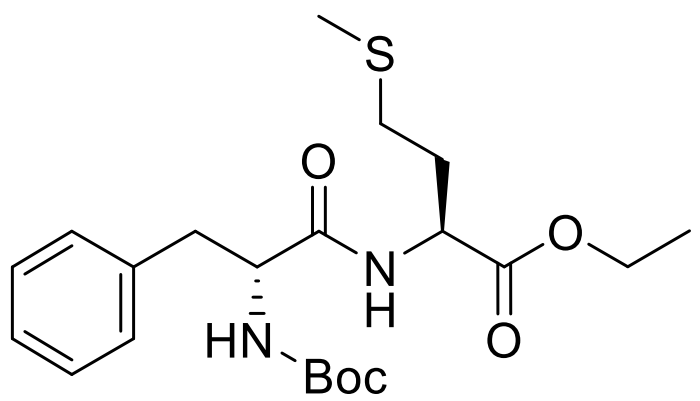

4ap

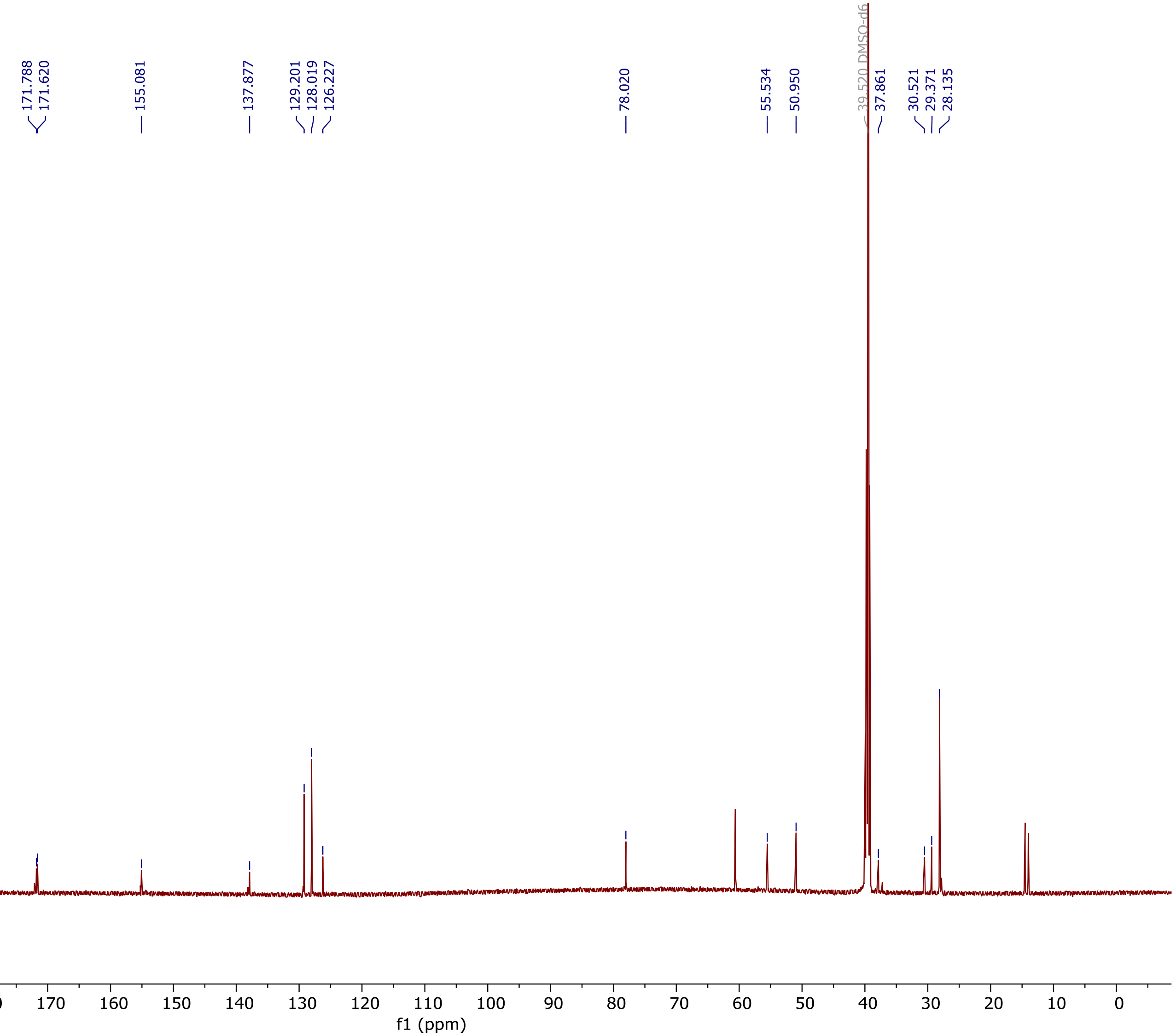

5.85 ^{1}H NMR spectrum S1a (400 MHz, $CDCl_3$)

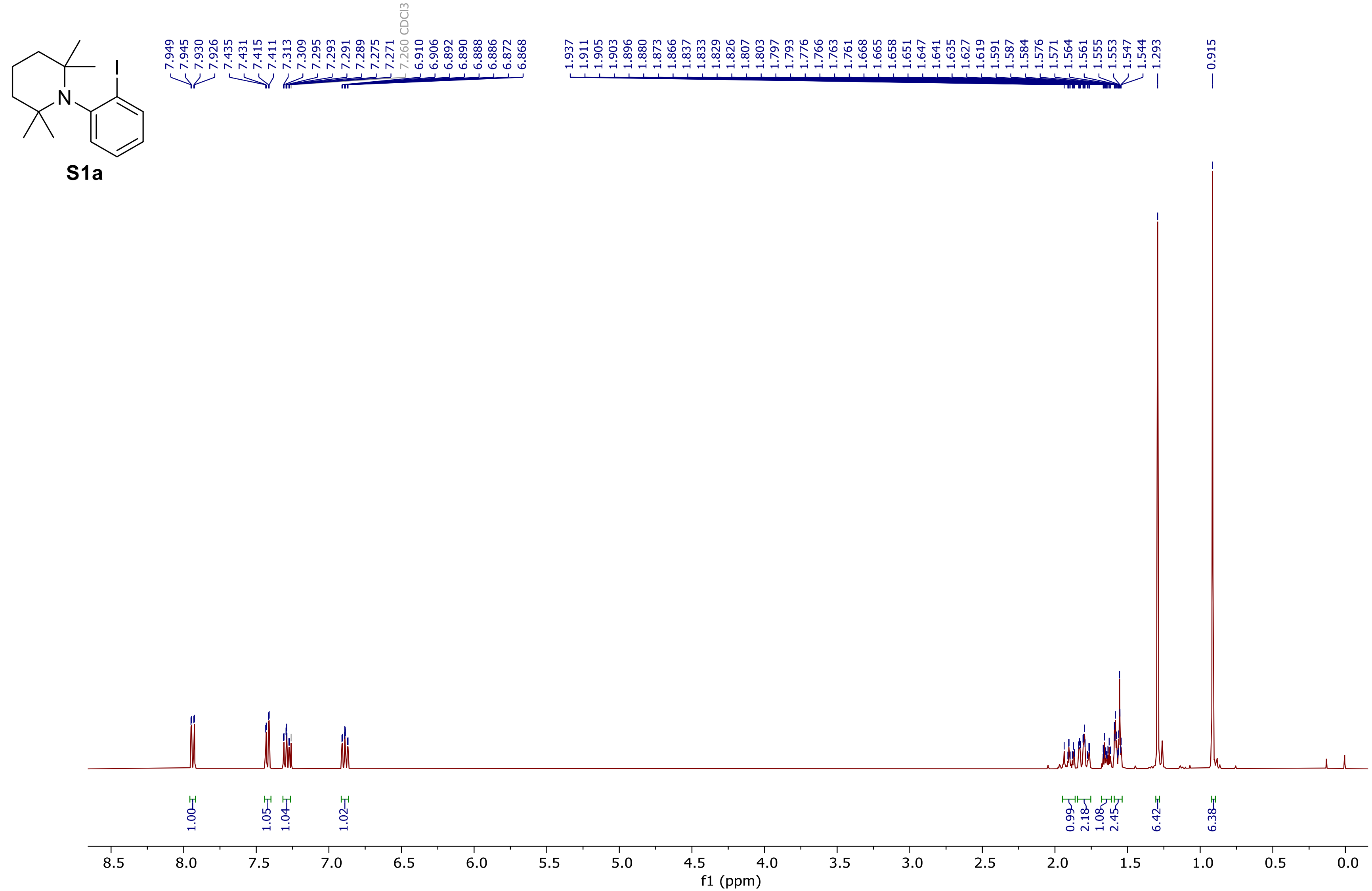

5.86 $^{13}C\{^{1}H\}$ NMR spectrum S1a (101 MHz, $CDCl_3$)

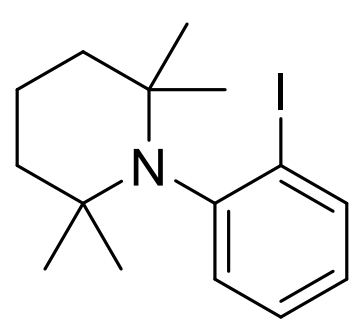

S1a

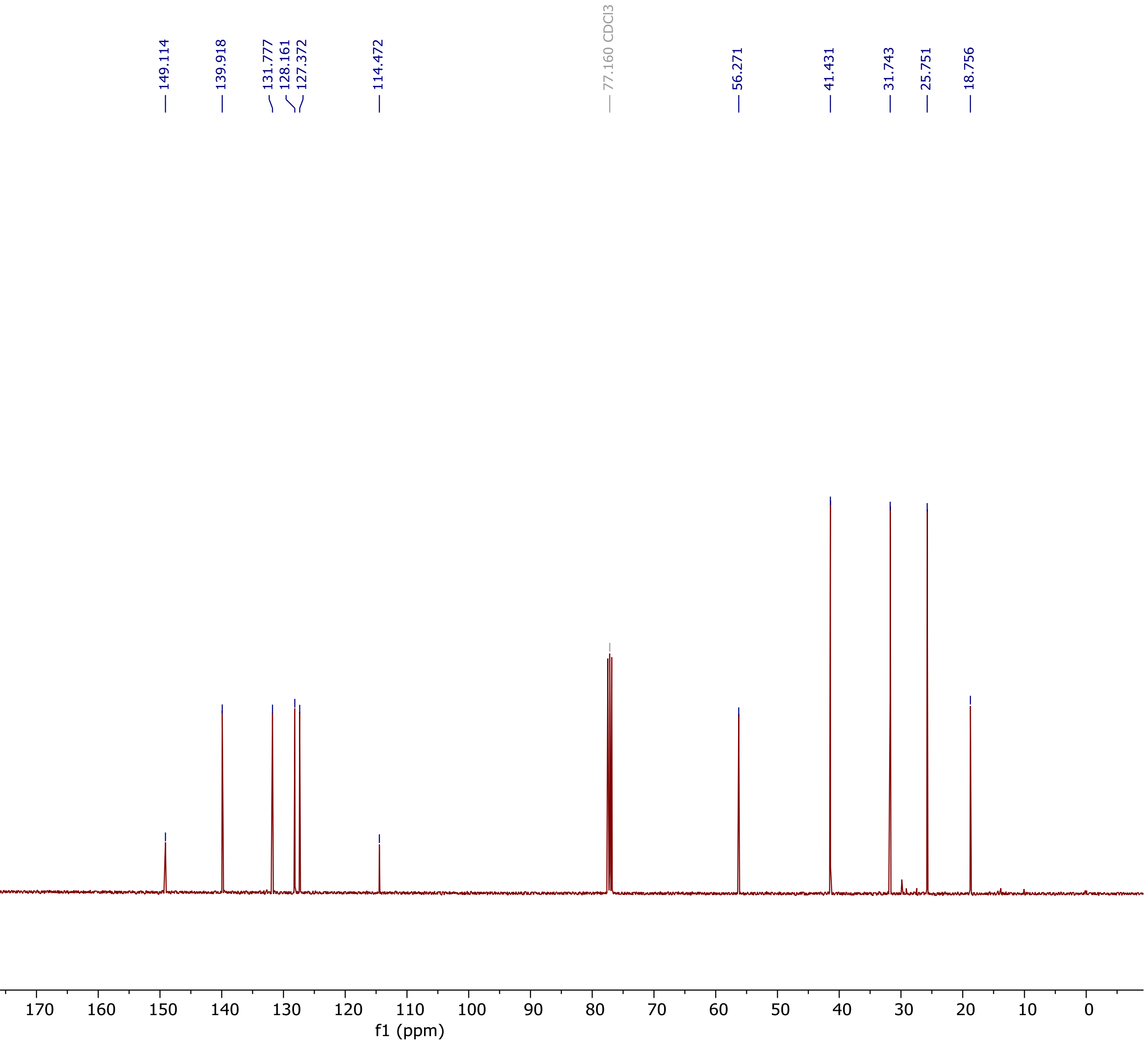

5.87 ^{1}H NMR spectrum S1b (600 MHz, DMSO-d$_6$)

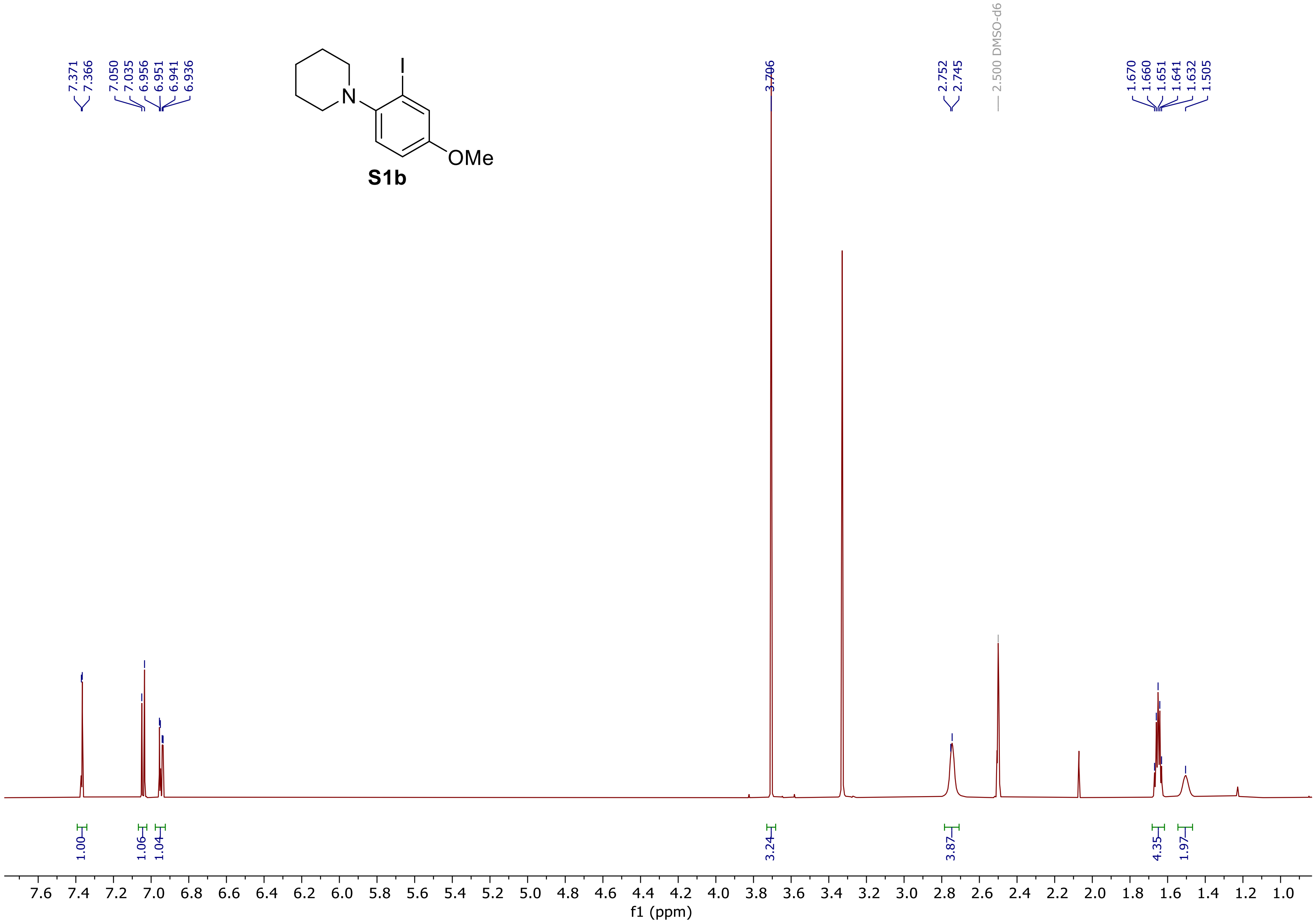

5.88 $^{13}C\{^{1}H\}$ NMR spectrum S1b (151 MHz, DMSO-d$_6$)

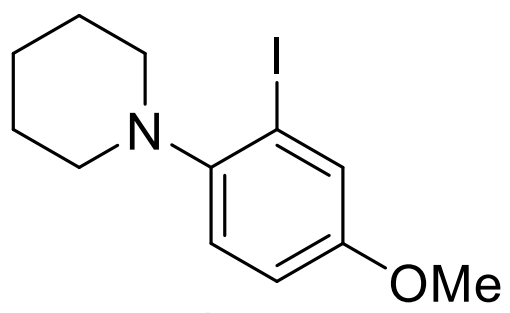

S1b

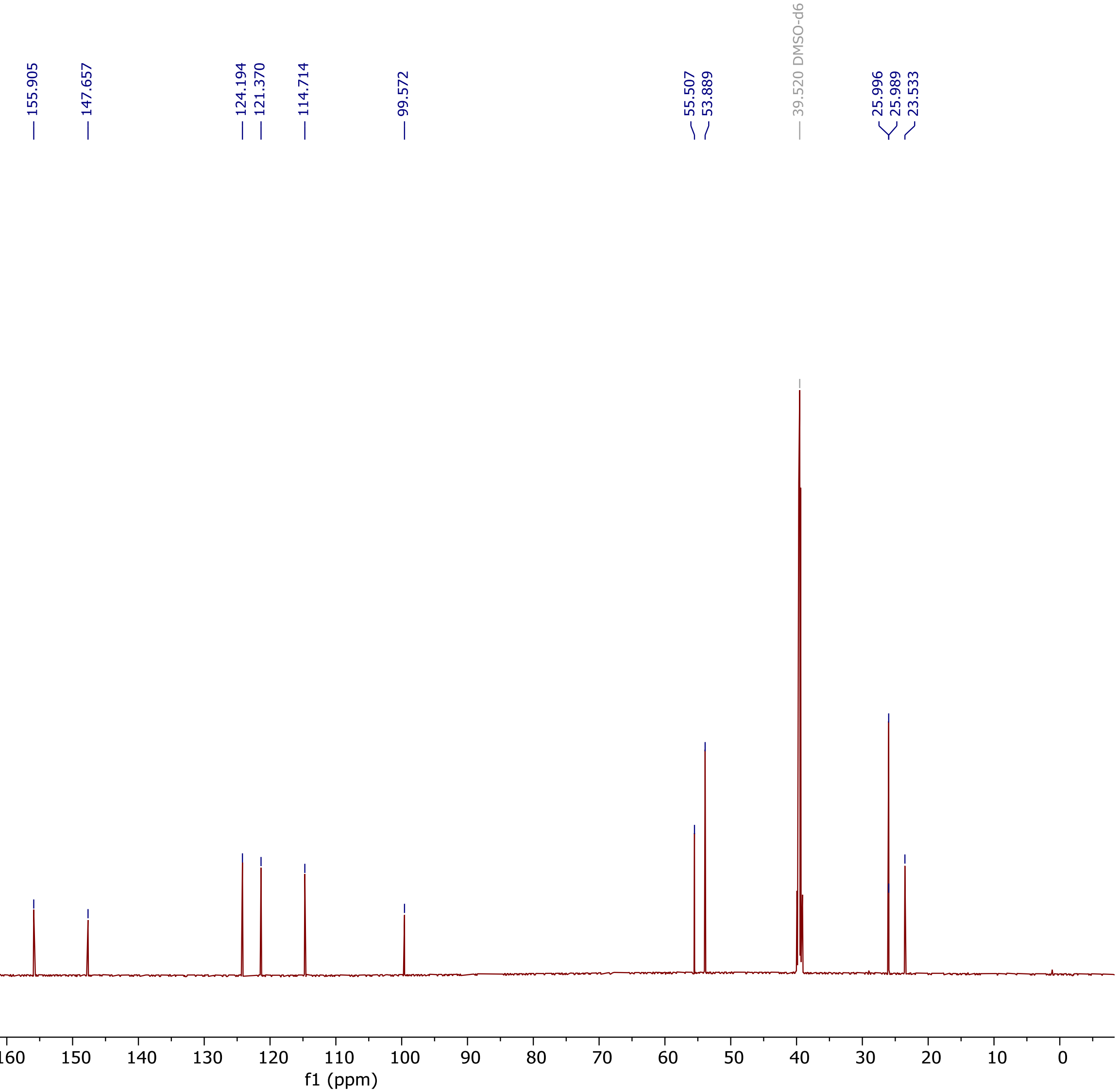

5.89 ^{1}H NMR spectrum 3s (400 MHz, $CDCl_3$)

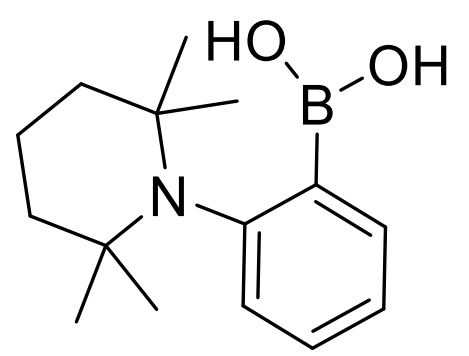

3s

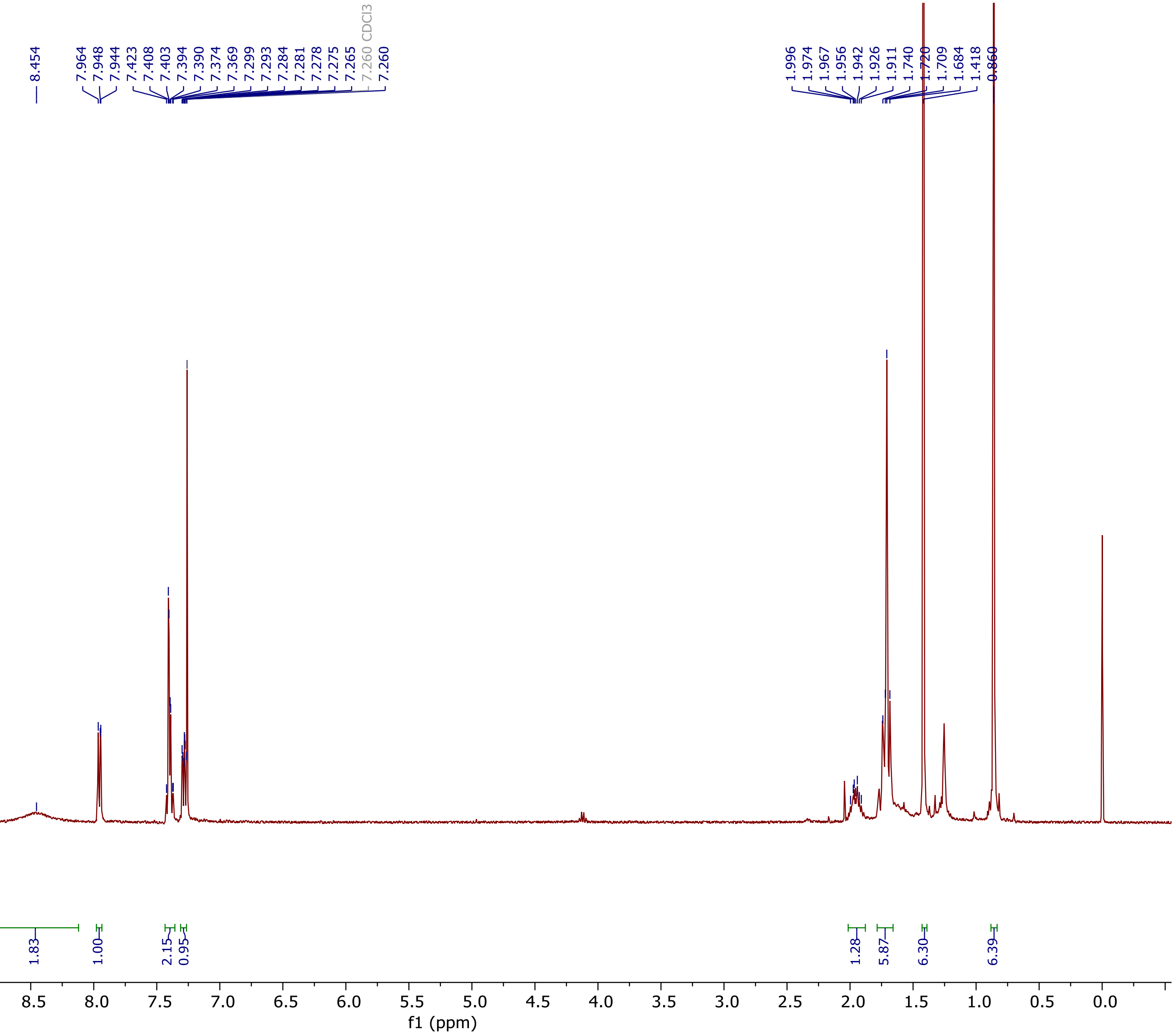

5.90 $^{13}C\{^1H\}$ NMR spectrum 3s (101 MHz, $CDCl_3$)

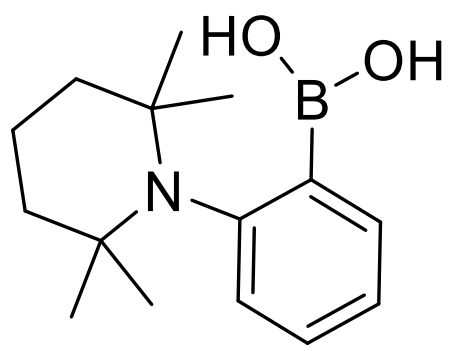

3s

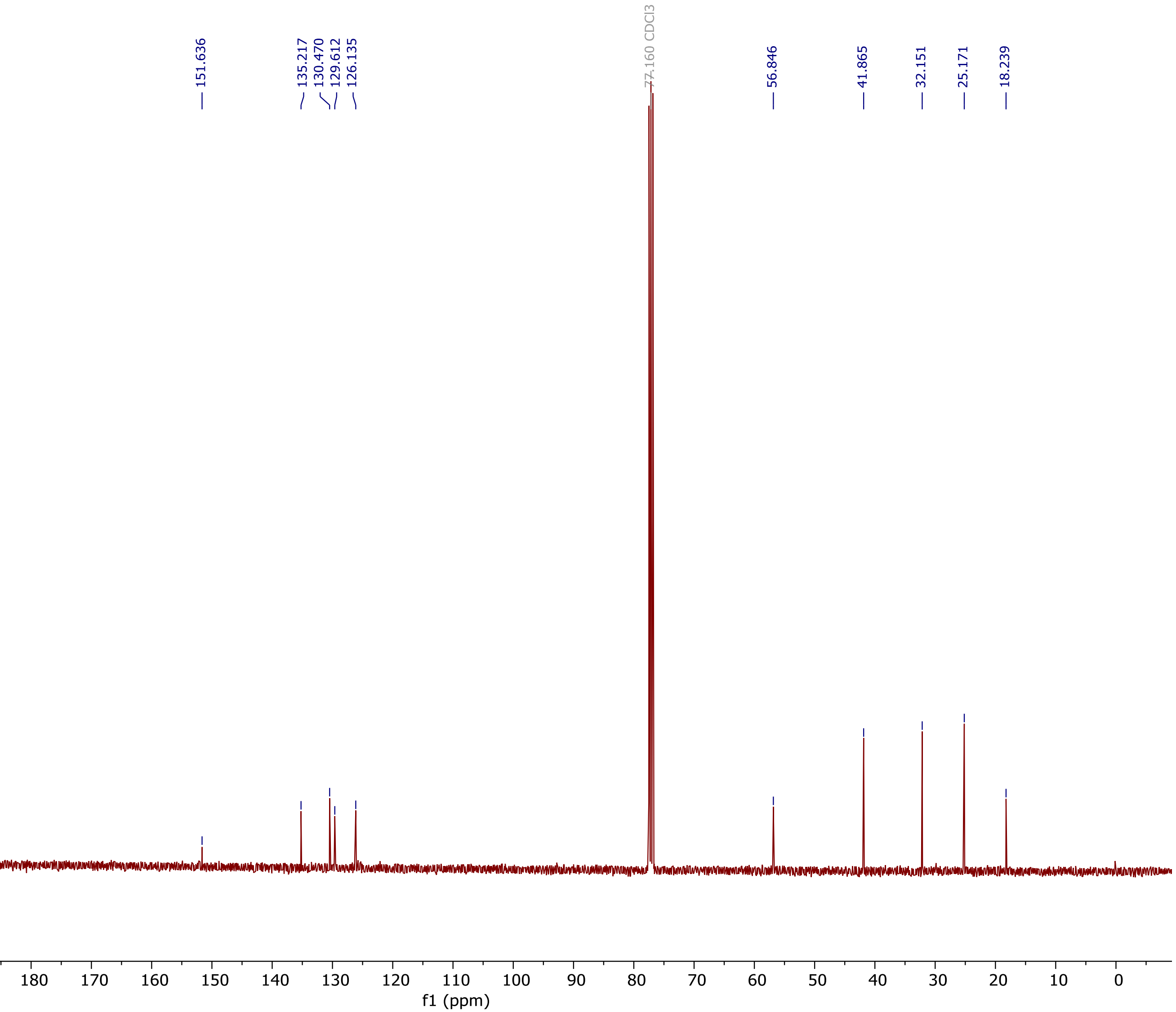

5.91 ^{1}H NMR spectrum 3t (400 MHz, DMSO-d$_6$)

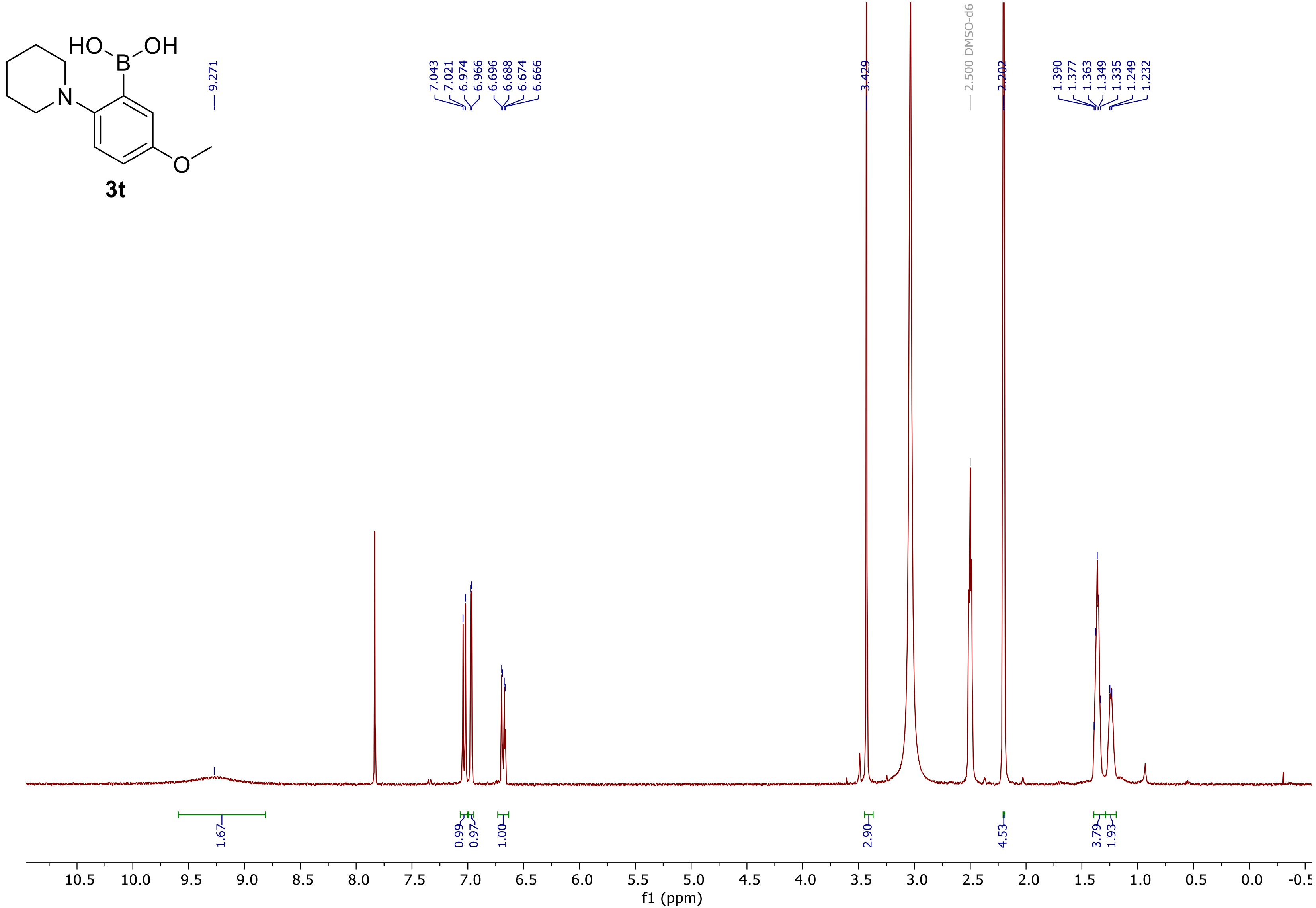

5.92 $^{13}C\{^{1}H\}$ NMR spectrum 3t (101 MHz, DMSO-d$_6$)

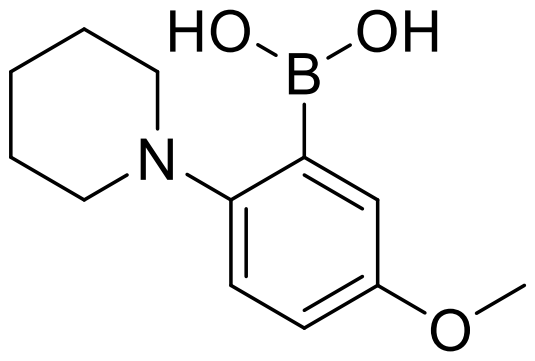

3t

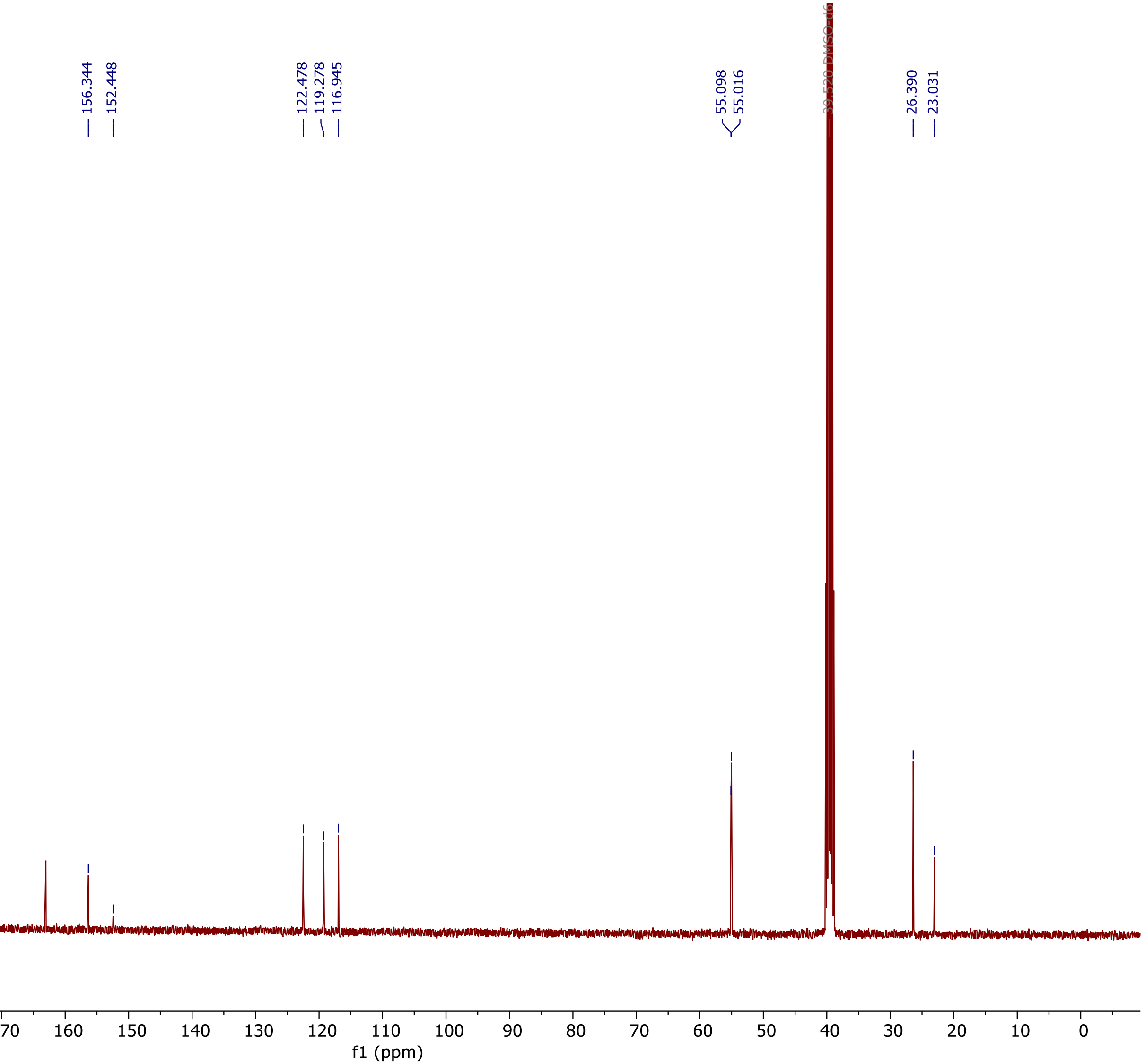

Sample_info n.2mg/ml ACN:MeOH 1:1

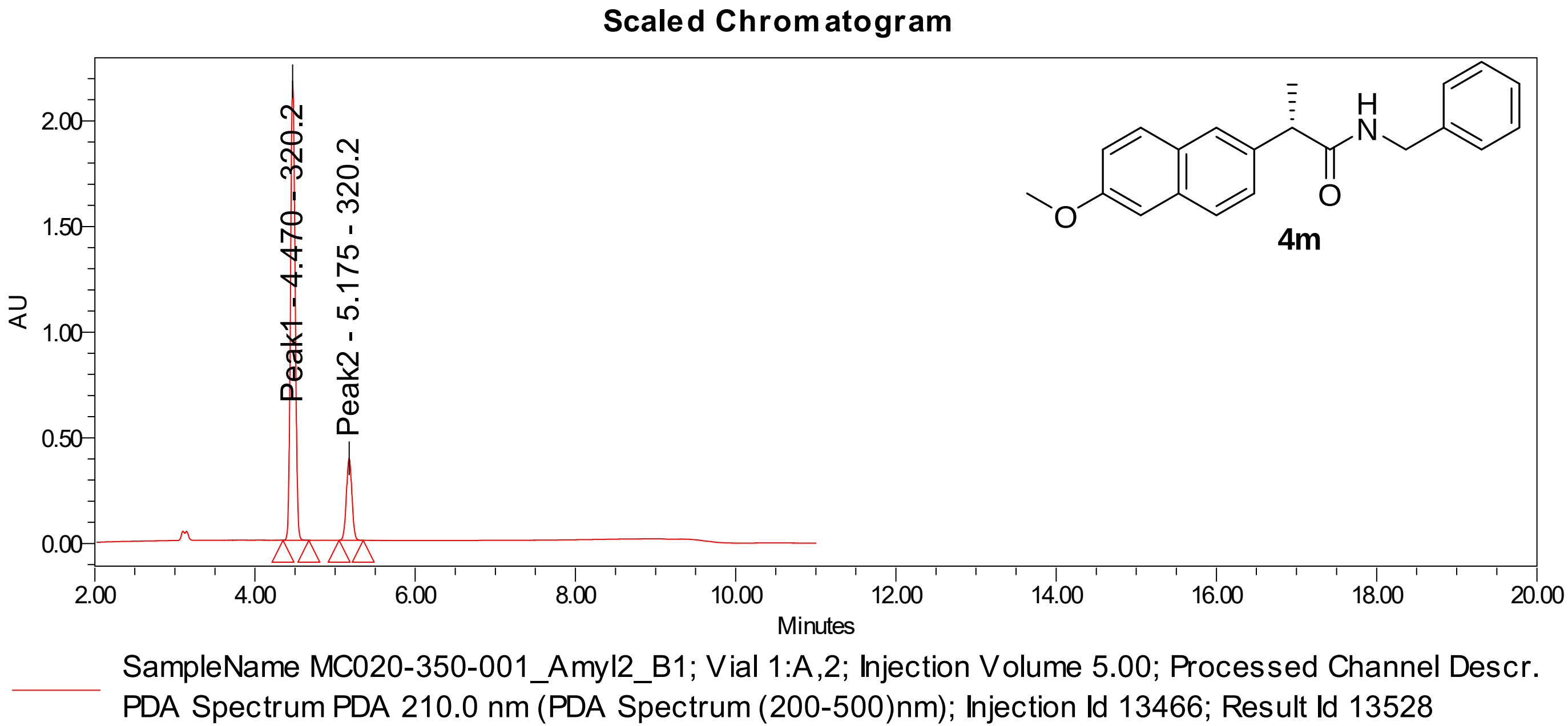

Peak Results

	SampleName	Peak Name	Retention Time (min)	Height (µV)	Area (µV*sec)	% Area
1	MC020-350-001_Amyl2_B1	Peak1	4.470	2172875	8658089	82.24
2	MC020-350-001_Amyl2_B1	Peak2	5.175	387679	1869261	17.76

Sample_info n.2mg/ml ACN:MeOH 1:1

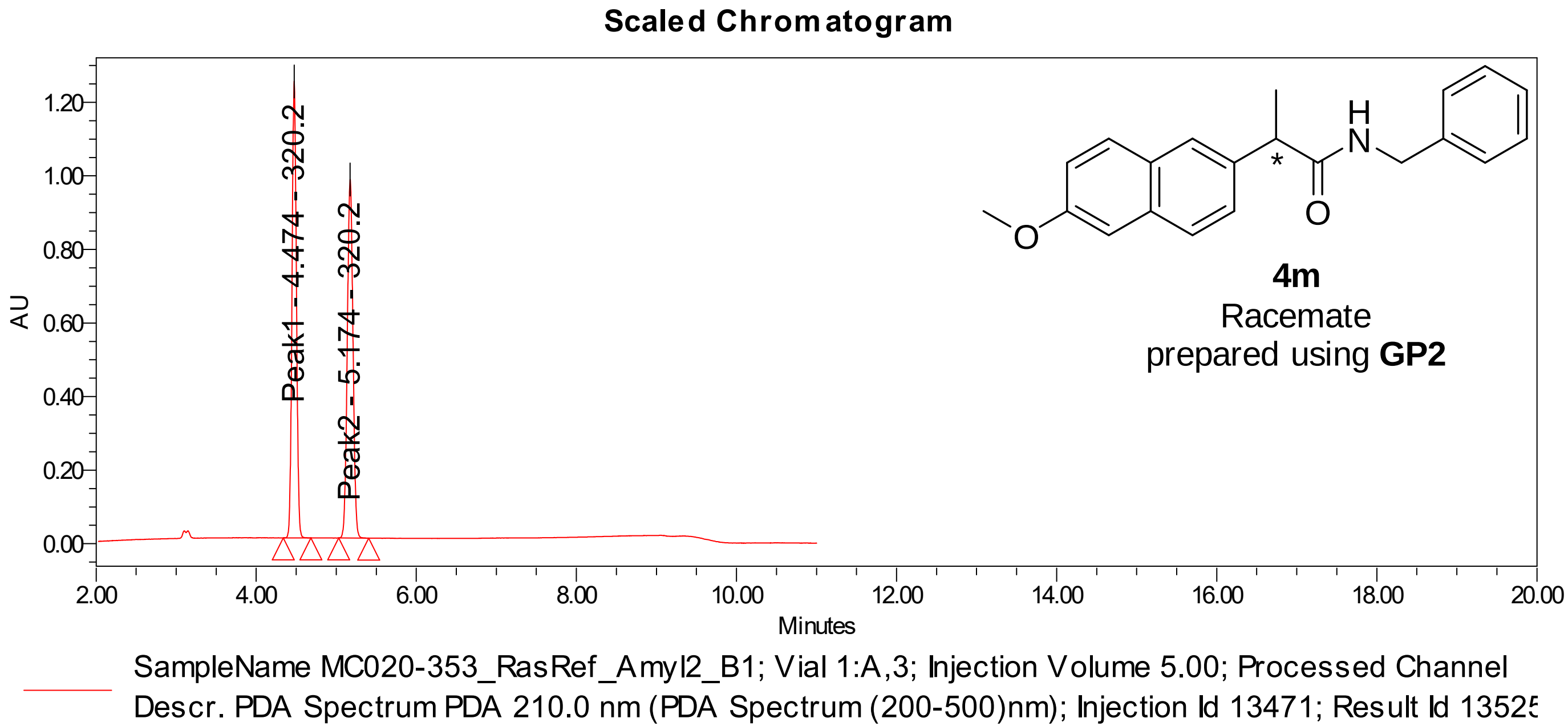

Peak Results

	SampleName	Peak Name	Retention Time (min)	Height (µV)	Area (µV*sec)	% Area
1	MC020-353_RasRef_Amyl2_B1	Peak1	4.474	1242154	4752391	49.74
2	MC020-353_RasRef_Amyl2_B1	Peak2	5.174	974729	4801190	50.26

Sample_info n.2mg/ml ACN:MeOH 1:1

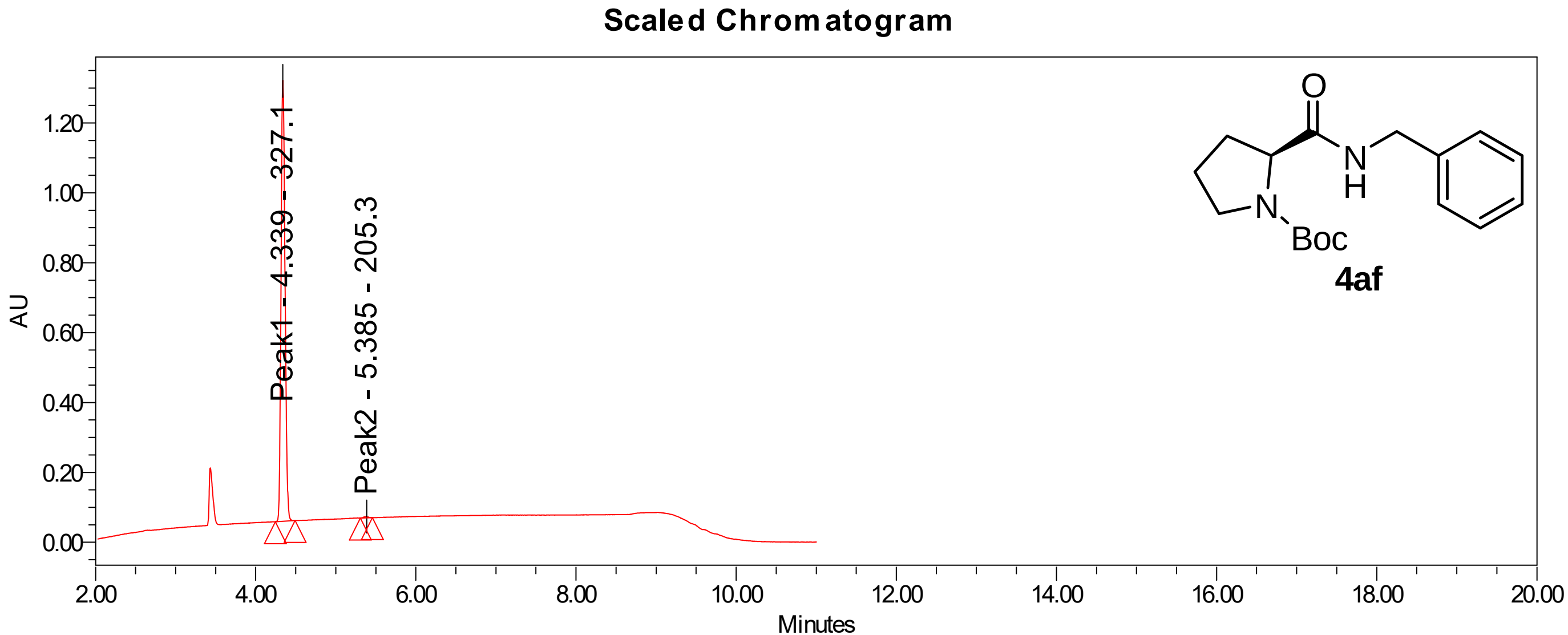

SampleName MC020-338-001_Cel5_B4; Vial 1:A,2; Injection Volume 5.00; Processed Channel Descr. PDA Spectrum PDA 210.0 nm (PDA Spectrum (200-500)nm); Injection Id 11565; Result Id 11581

Peak Results

	SampleName	Peak Name	Retention Time (min)	Height (µV)	Area (µV*sec)	% Area
1	MC020-338-001_Cel5_B4	Peak1	4.339	1262235	4399916	99.69
2	MC020-338-001_Cel5_B4	Peak2	5.385	3380	13667	0.31

Sample_info n.2mg/ml ACN:MeOH 1:1

Scaled Chromatogram

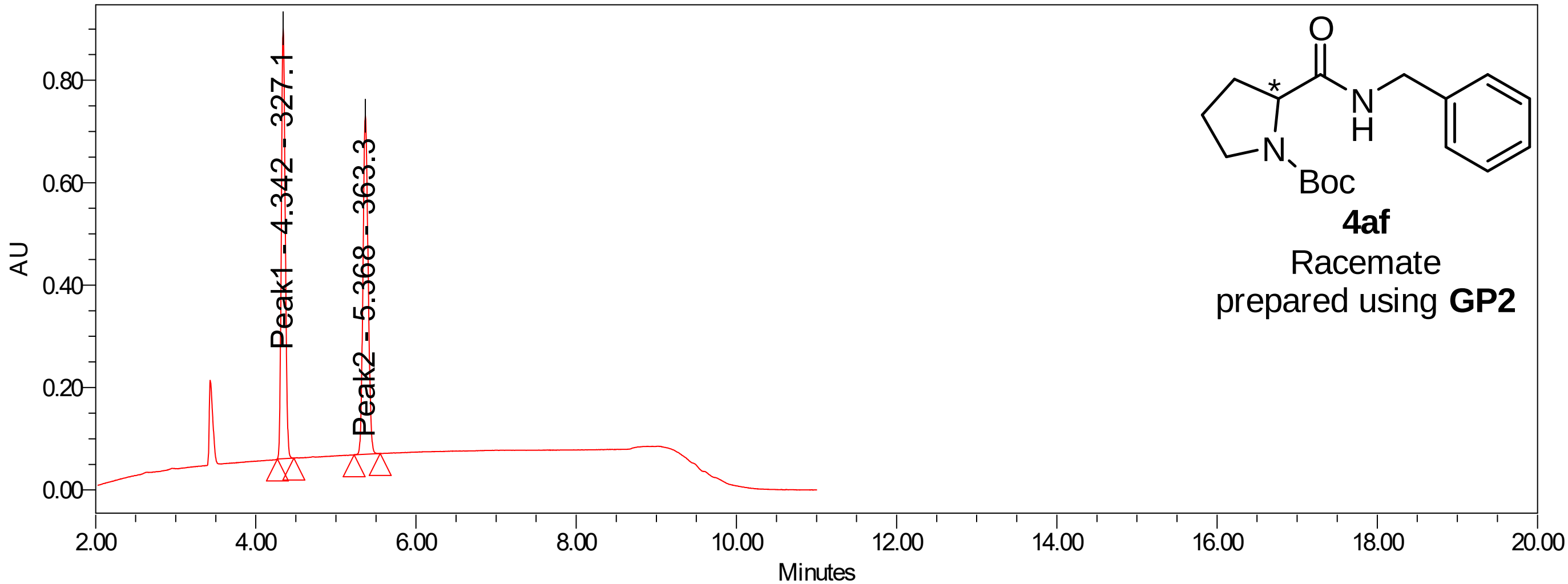

SampleName MC020-339_RasRef_Cel5_B4; Vial 1:A,3; Injection Volume 5.00; Processed Channel Descr. PDA Spectrum PDA 210.0 nm (PDA Spectrum (200-500)nm); Injection Id 11570; Result Id 1157

Peak Results

	SampleName	Peak Name	Retention Time (min)	Height (µV)	Area (µV*sec)	% Area
1	MC020-339_RasRef_Cel5_B4	Peak1	4.342	840955	2842530	49.09
2	MC020-339_RasRef_Cel5_B4	Peak2	5.368	661759	2947814	50.91

Sample_info n.2mg/ml ACN:MeOH 1:1

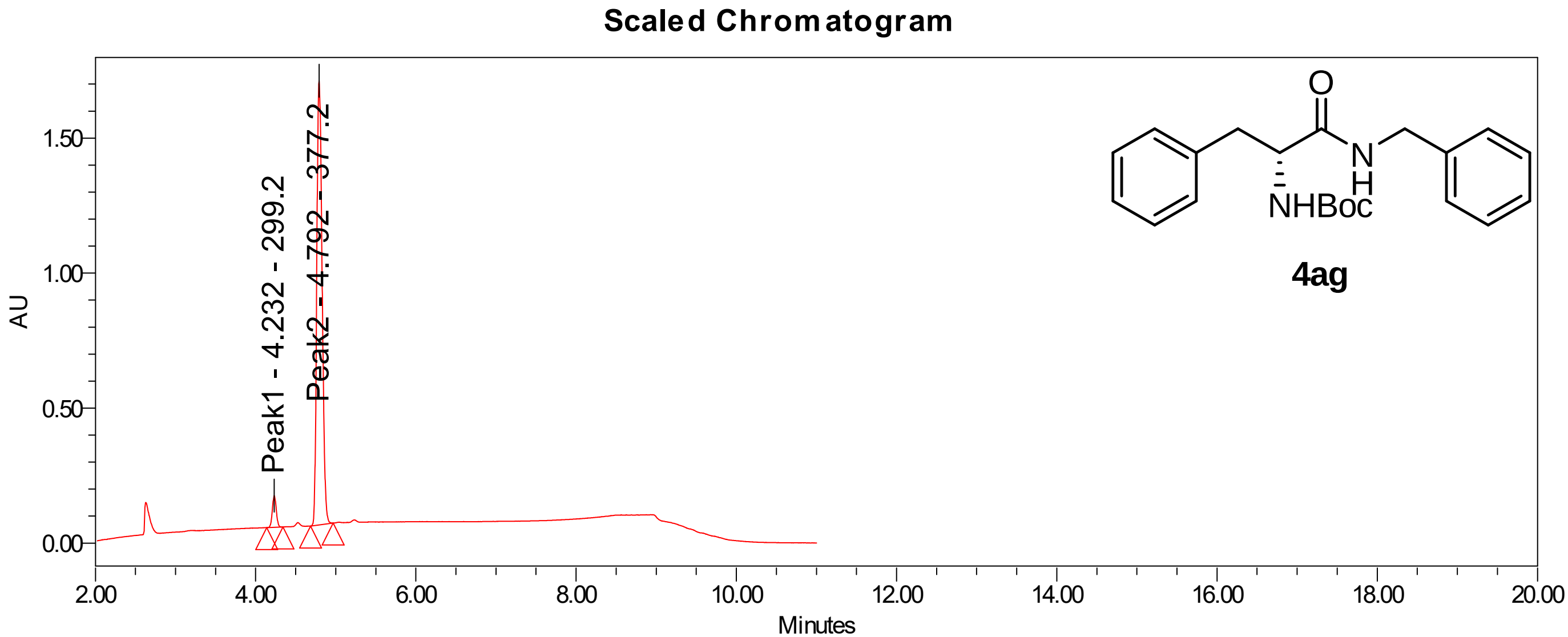

4ag

SampleName MC020-317-001_Amyl1_B4; Vial 1:A,2; Injection Volume 5.00; Processed Channel Descr. PDA Spectrum PDA 210.0 nm (PDA Spectrum (200-500)nm); Injection Id 12533; Result Id 12553

Peak Results

	SampleName	Peak Name	Retention Time (min)	Height (µV)	Area (µV*sec)	% Area
1	MC020-317-001_Amyl1_B4	Peak1	4.232	117095	396414	4.91
2	MC020-317-001_Amyl1_B4	Peak2	4.792	1645348	7676752	95.09

Sample_info n.2mg/ml ACN:MeOH 1:1

Scaled Chromatogram

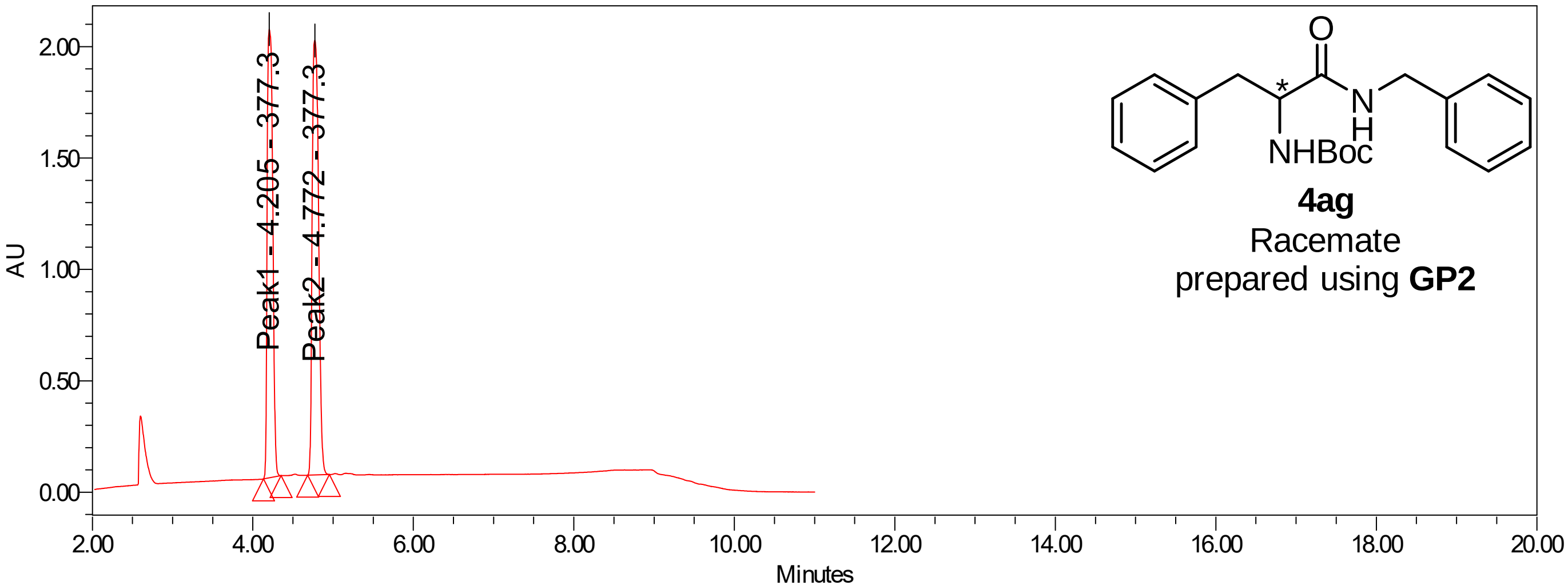

SampleName MC020-348_RasRef_Amyl1_B4; Vial 1:A,3; Injection Volume 5.00; Processed Channel Descr. PDA Spectrum PDA 210.0 nm (PDA Spectrum (200-500)nm); Injection Id 13781; Result Id 1378

Peak Results

	SampleName	Peak Name	Retention Time (min)	Height (µV)	Area (µV*sec)	% Area
1	MC020-348_RasRef_Amyl1_B4	Peak1	4.205	2013715	9636857	47.09
2	MC020-348_RasRef_Amyl1_B4	Peak2	4.772	1950449	10826195	52.91

Sample_info n.2mg/ml ACN:MeOH 1:1

Scaled Chromatogram

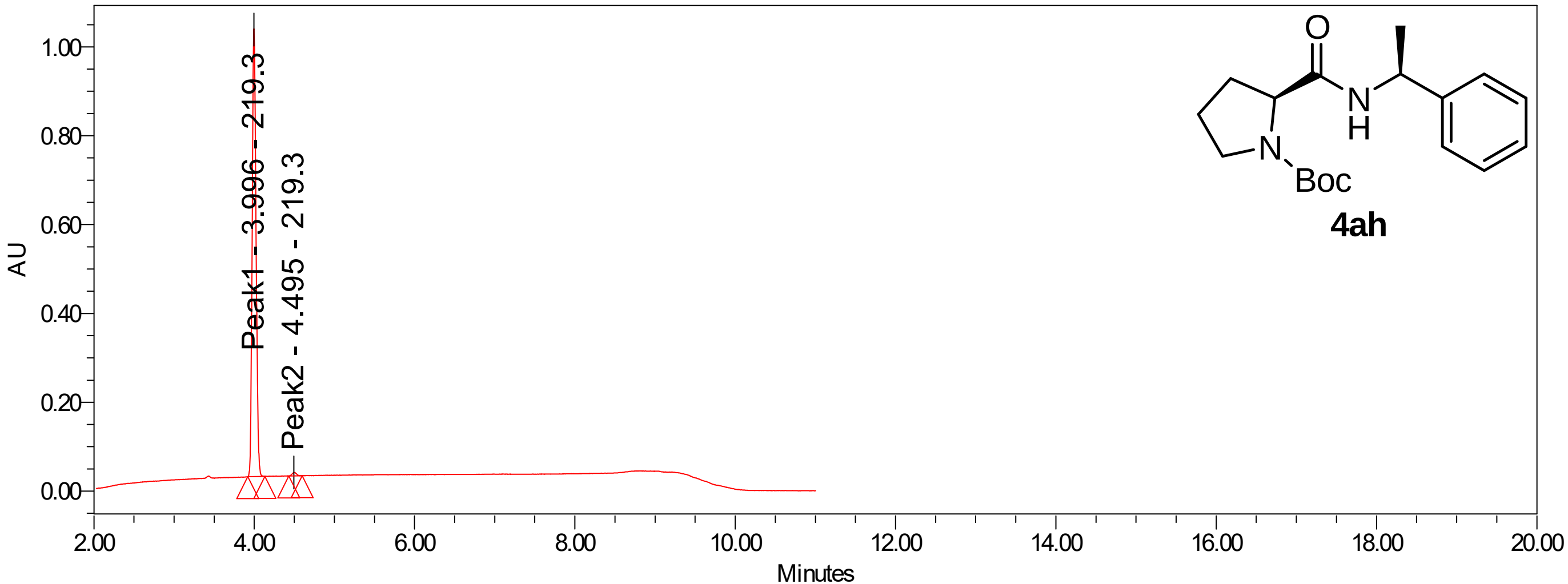

SampleName MC020-370-001_Cel5_B2; Vial 1:A,2; Injection Volume 5.00; Processed Channel Descr. PDA Spectrum PDA 210.0 nm (PDA Spectrum (200-500)nm); Injection Id 15273; Result Id 15289

Peak Results

	SampleName	Peak Name	Retention Time (min)	Height (µV)	Area (µV*sec)	% Area
1	MC020-370-001_Cel5_B2	Peak1	3.996	1008313	3392628	99.16
2	MC020-370-001_Cel5_B2	Peak2	4.495	7703	28684	0.84

Sample_info n.2mg/ml ACN:MeOH 1:1

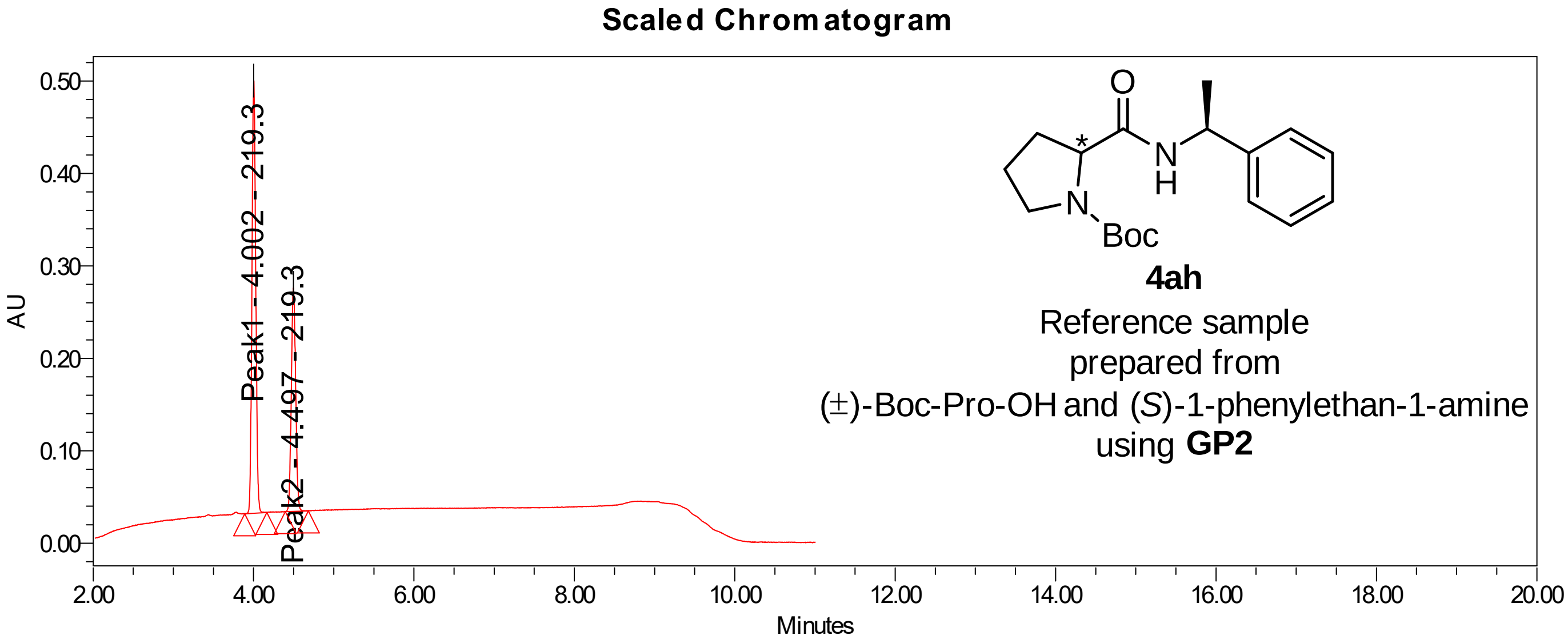

4ah
Reference sample
prepared from
(±)-Boc-Pro-OH and (*S*)-1-phenylethan-1-amine
using **GP2**

SampleName MC020-369_RasRef_Cel5_B2; Vial 1:A,3; Injection Volume 5.00; Processed Channel Descr. PDA Spectrum PDA 210.0 nm (PDA Spectrum (200-500)nm); Injection Id 15278; Result Id 1528€

Peak Results

	SampleName	Peak Name	Retention Time (min)	Height (µV)	Area (µV*sec)	% Area
1	MC020-369_RasRef_Cel5_B2	Peak1	4.002	468814	1543019	62.90
2	MC020-369_RasRef_Cel5_B2	Peak2	4.497	241865	910017	37.10

Sample_info n.2mg/ml ACN:MeOH 1:1

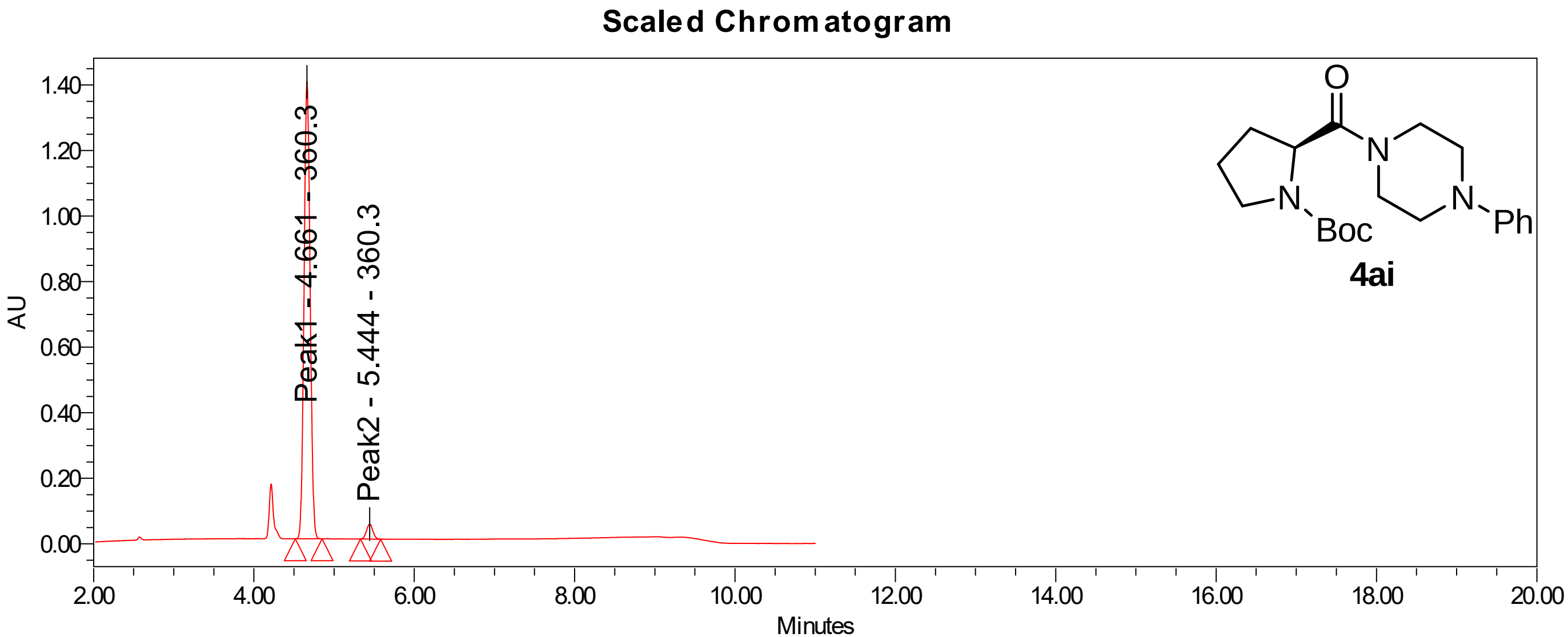

SampleName MC020-366-001_Cel2_B1; Vial 1:A,2; Injection Volume 5.00; Processed Channel Descr. PDA Spectrum PDA 210.0 nm (PDA Spectrum (200-500)nm); Injection Id 14329; Result Id 14347

Peak Results

	SampleName	Peak Name	Retention Time (min)	Height (µV)	Area (µV*sec)	% Area
1	MC020-366-001_Cel2_B1	Peak1	4.661	1395406	7365054	96.72
2	MC020-366-001_Cel2_B1	Peak2	5.444	45694	250109	3.28

Sample_info n.2mg/ml ACN:MeOH 1:1

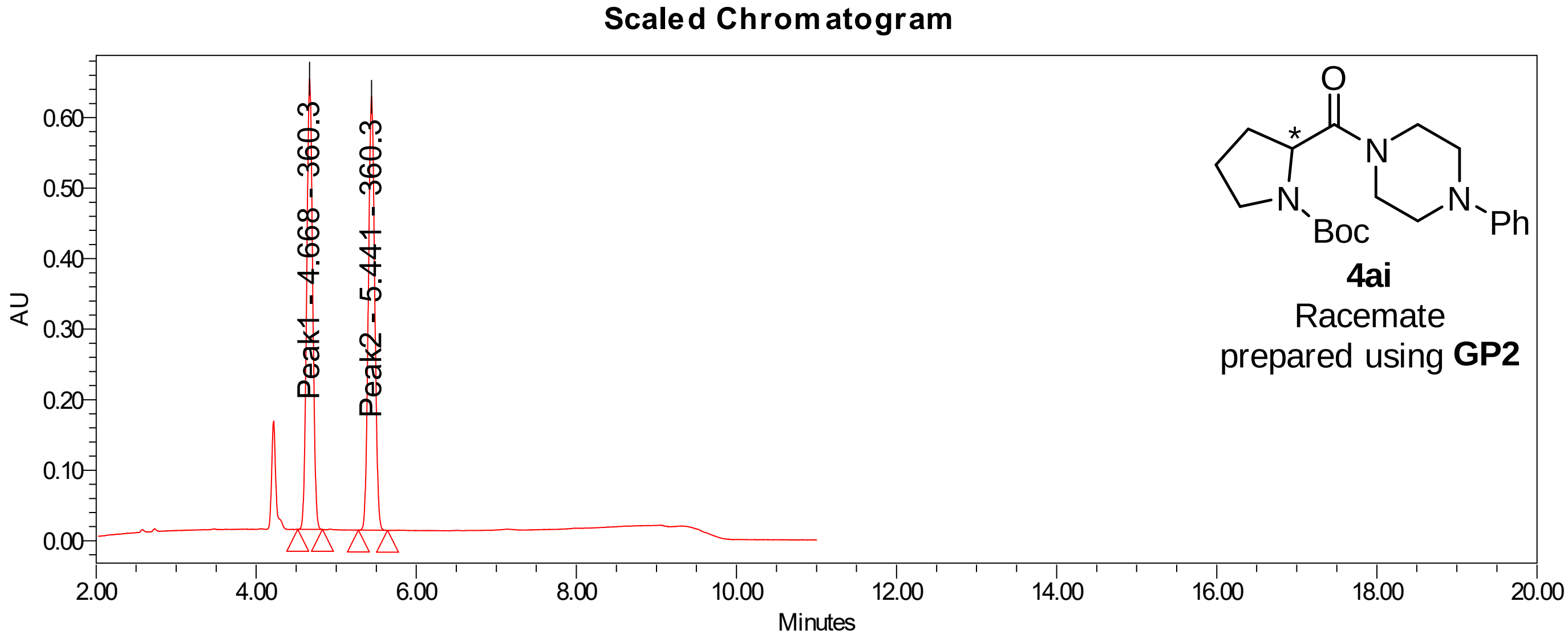

4ai
Racemate
prepared using **GP2**

SampleName MC020-365_RasRef_Cel2_B1; Vial 1:A,3; Injection Volume 5.00; Processed Channel Descr. PDA Spectrum PDA 210.0 nm (PDA Spectrum (200-500)nm); Injection Id 14334; Result Id 1434

Peak Results

	SampleName	Peak Name	Retention Time (min)	Height (µV)	Area (µV*sec)	% Area
1	MC020-365_RasRef_Cel2_B1	Peak1	4.668	639217	3320116	49.42
2	MC020-365_RasRef_Cel2_B1	Peak2	5.441	614779	3398517	50.58

Sample_info n.1mg/ml ACN:MeOH 1:1

Scaled Chromatogram

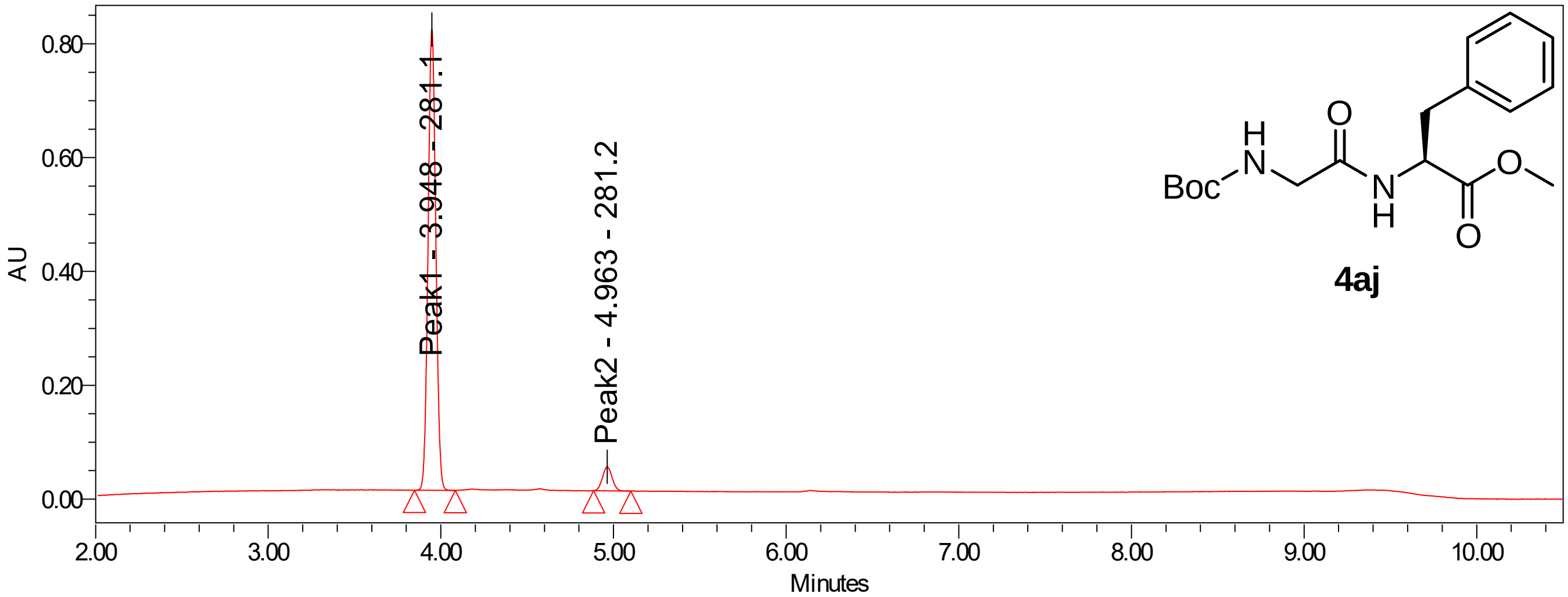

SampleName MC020-410-001_Cel2_B3; Vial 1:A,8; Injection Volume 5.00; Processed Channel Descr. PDA Spectrum PDA 210.0 nm (PDA Spectrum (200-500)nm); Injection Id 19271; Result Id 19293

Peak Results

	SampleName	Peak Name	Retention Time (min)	Height (µV)	Area (µV*sec)	% Area
1	MC020-410-001_Cel2_B3	Peak1	3.948	810592	2531434	93.95
2	MC020-410-001_Cel2_B3	Peak2	4.963	42140	163070	6.05

Sample_info n.1mg/ml ACN:MeOH 1:1

Scaled Chromatogram

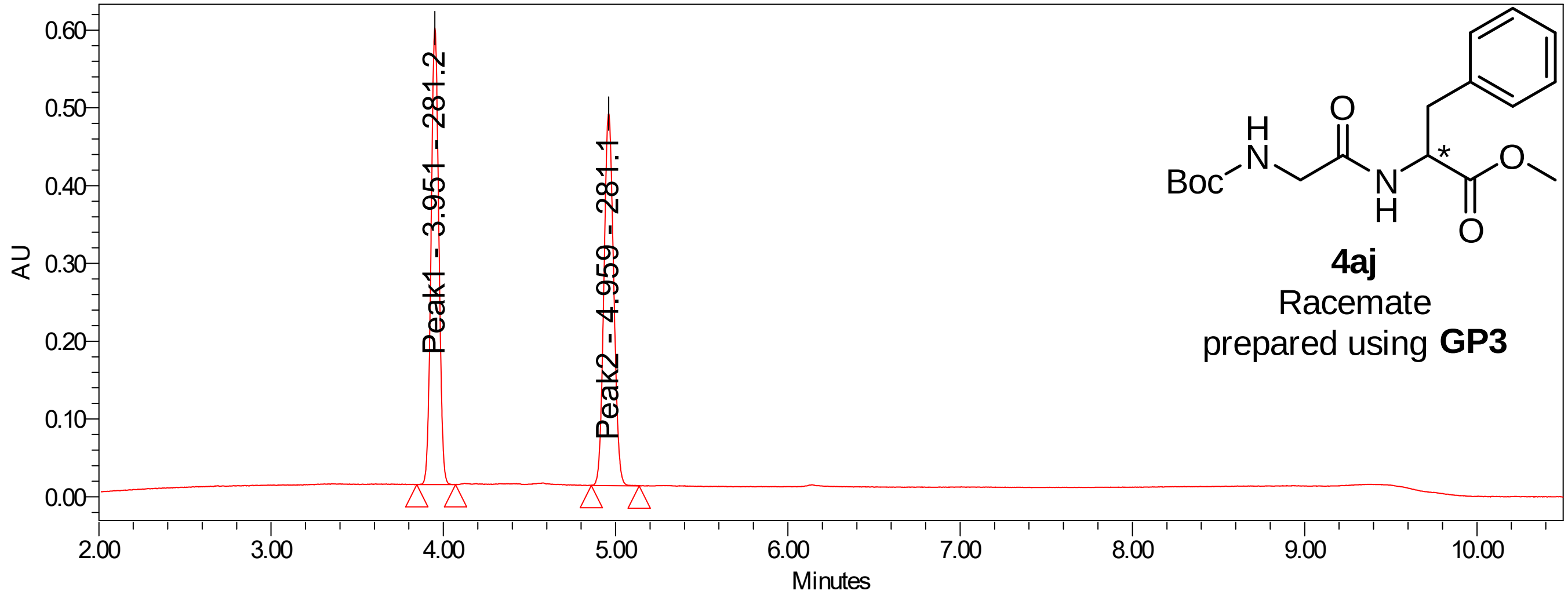

SampleName MC020-393_RasRef_Cel2_B3; Vial 1:A,7; Injection Volume 5.00; Processed Channel Descr. PDA Spectrum PDA 210.0 nm (PDA Spectrum (200-500)nm); Injection Id 19276; Result Id 1929(

Peak Results

	SampleName	Peak Name	Retention Time (min)	Height (µV)	Area (µV*sec)	% Area
1	MC020-393_RasRef_Cel2_B3	Peak1	3.951	587220	1835004	49.35
2	MC020-393_RasRef_Cel2_B3	Peak2	4.959	478417	1883246	50.65

Sample_info n.2mg/ml ACN:MeOH 1:1

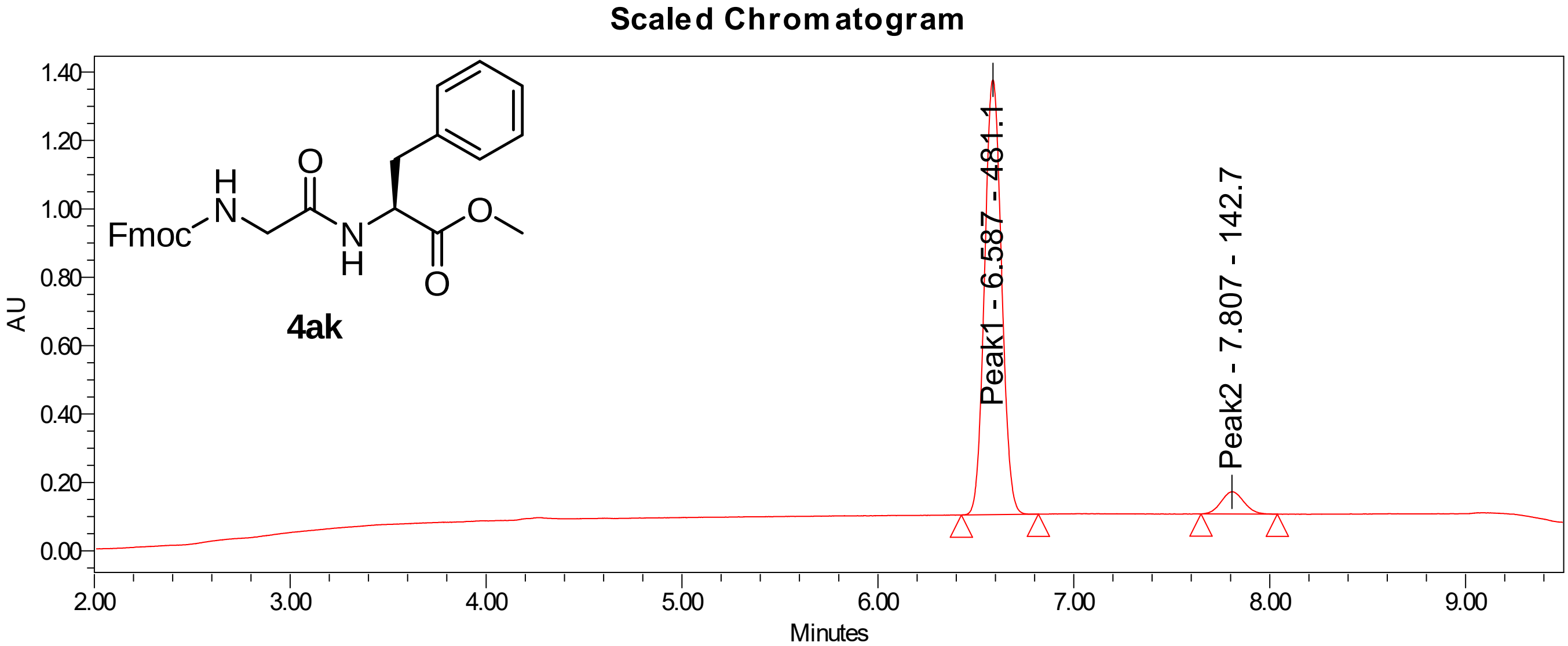

SampleName MC020-388-001_Op_Cel4; Vial 1:A,4; Injection Volume 2.00; Processed Channel Descr. PDA Spectrum PDA 210.0 nm (PDA Spectrum (200-500)nm); Injection Id 18898; Result Id 19013

Peak Results

	SampleName	Peak Name	Retention Time (min)	Height (µV)	Area (µV*sec)	% Area
1	MC020-388-001_Op_Cel4	Peak1	6.587	1271756	7679073	93.60
2	MC020-388-001_Op_Cel4	Peak2	7.807	65160	524793	6.40

Sample_info n.1mg/ml ACN:MeOH 1:1

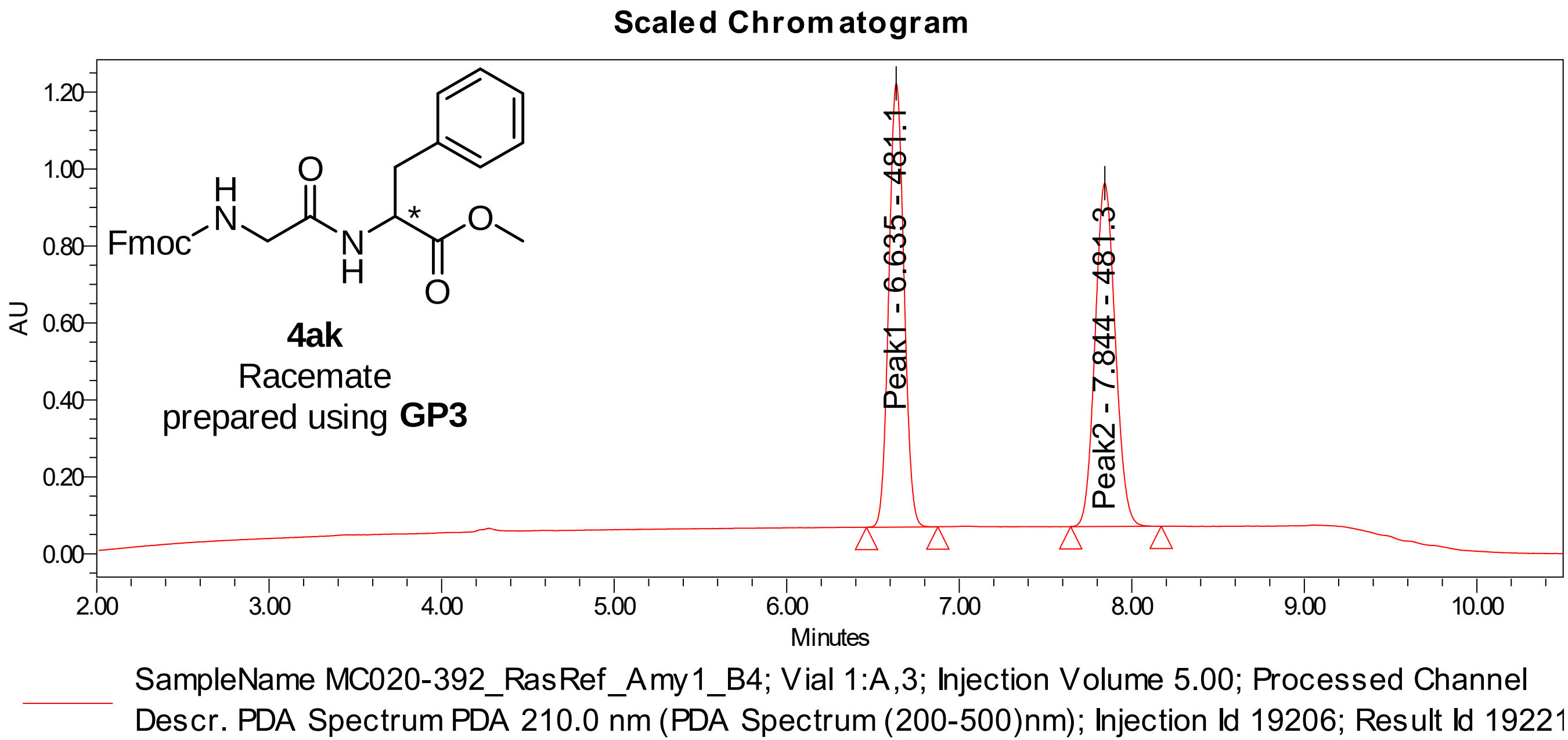

Peak Results

	SampleName	Peak Name	Retention Time (min)	Height (µV)	Area (µV*sec)	% Area
1	MC020-392_RasRef_Amy1_B4	Peak1	6.635	1153132	7029904	49.24
2	MC020-392_RasRef_Amy1_B4	Peak2	7.844	892423	7246130	50.76

Sample_info n.1mg/ml ACN:MeOH 1:1

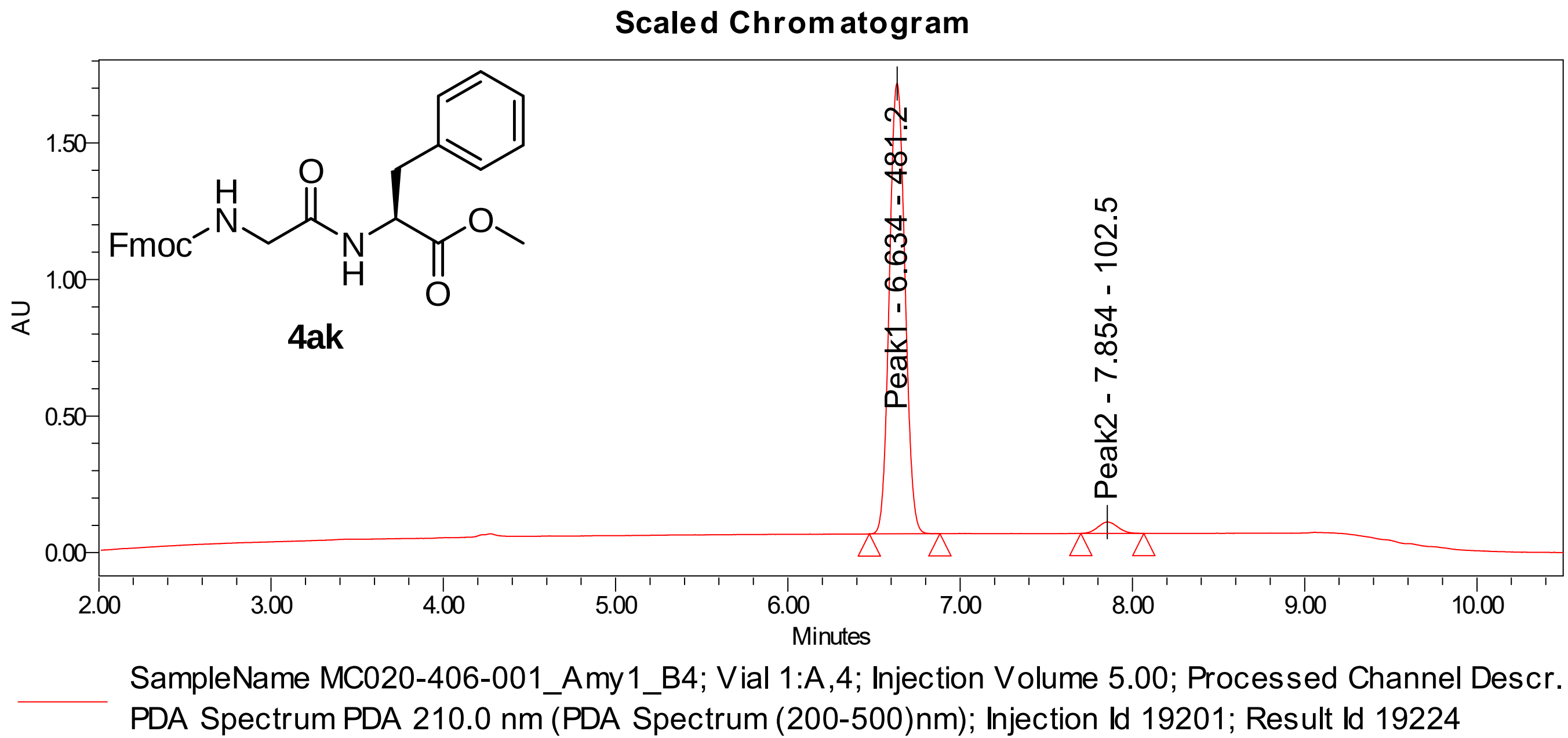

Peak Results

	SampleName	Peak Name	Retention Time (min)	Height (µV)	Area (µV*sec)	% Area
1	MC020-406-001_Amy1_B4	Peak1	6.634	1648814	10376494	96.86
2	MC020-406-001_Amy1_B4	Peak2	7.854	41893	336289	3.14

Sample_info n.2mg/ml ACN:MeOH 1:1

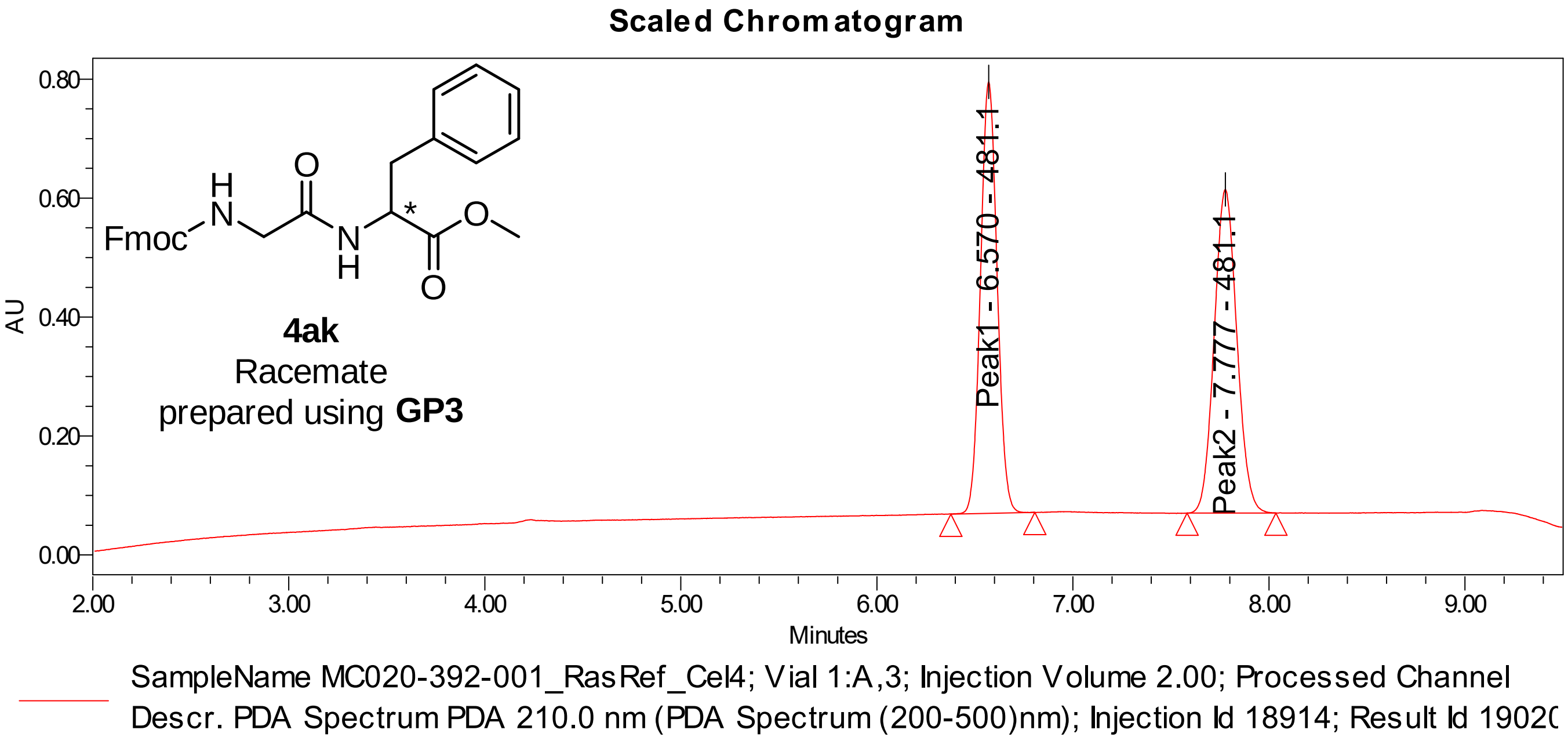

Peak Results

	SampleName	Peak Name	Retention Time (min)	Height (µV)	Area (µV*sec)	% Area
1	MC020-392-001_RasRef_Cel4	Peak1	6.570	725471	4276143	49.46
2	MC020-392-001_RasRef_Cel4	Peak2	7.777	544207	4370145	50.54

Sample_info n.2mg/ml ACN:MeOH 1:1

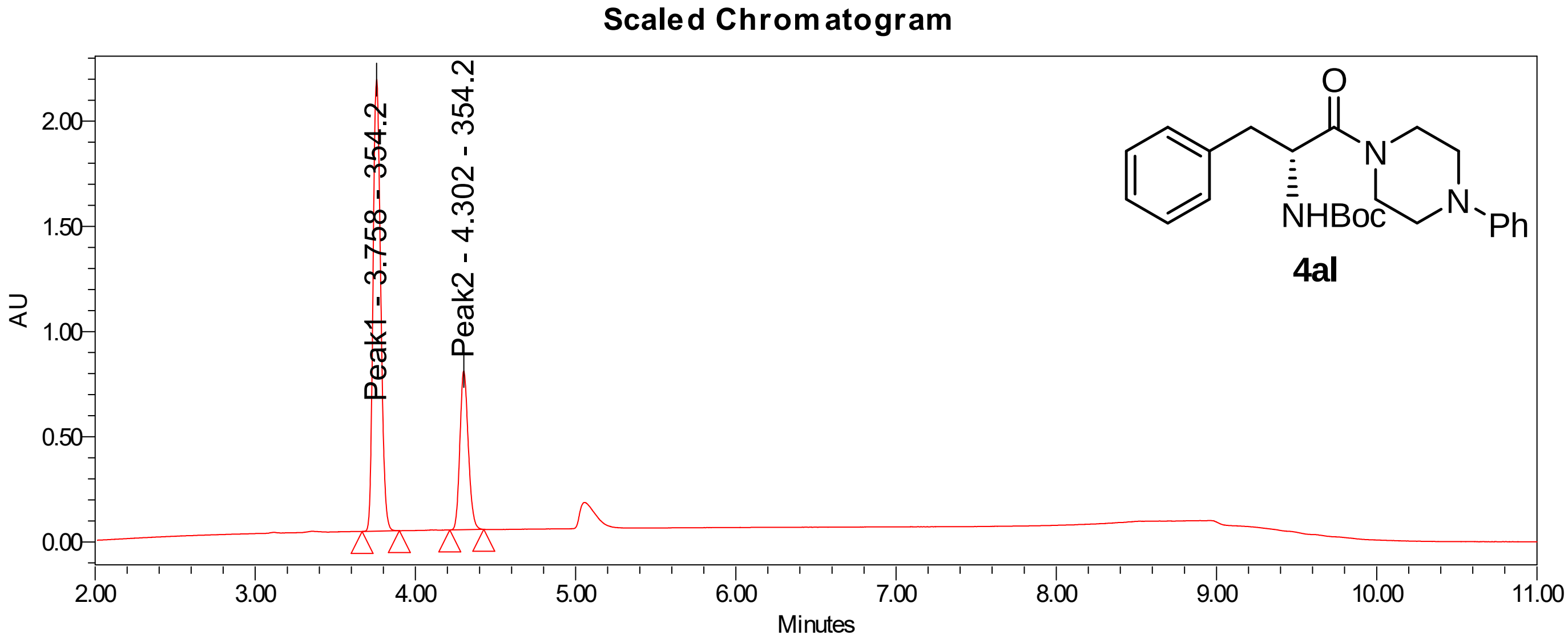

4al

SampleName MC020-374-001_Cel1_B4; Vial 1:A,2; Injection Volume 5.00; Processed Channel Descr. PDA Spectrum PDA 210.0 nm (PDA Spectrum (200-500)nm); Injection Id 16586; Result Id 16602

Peak Results

	SampleName	Peak Name	Retention Time (min)	Height (µV)	Area (µV*sec)	% Area
1	MC020-374-001_Cel1_B4	Peak1	3.758	2146876	7110121	72.14
2	MC020-374-001_Cel1_B4	Peak2	4.302	755317	2746399	27.86

Sample_info n.2mg/ml ACN:MeOH 1:1

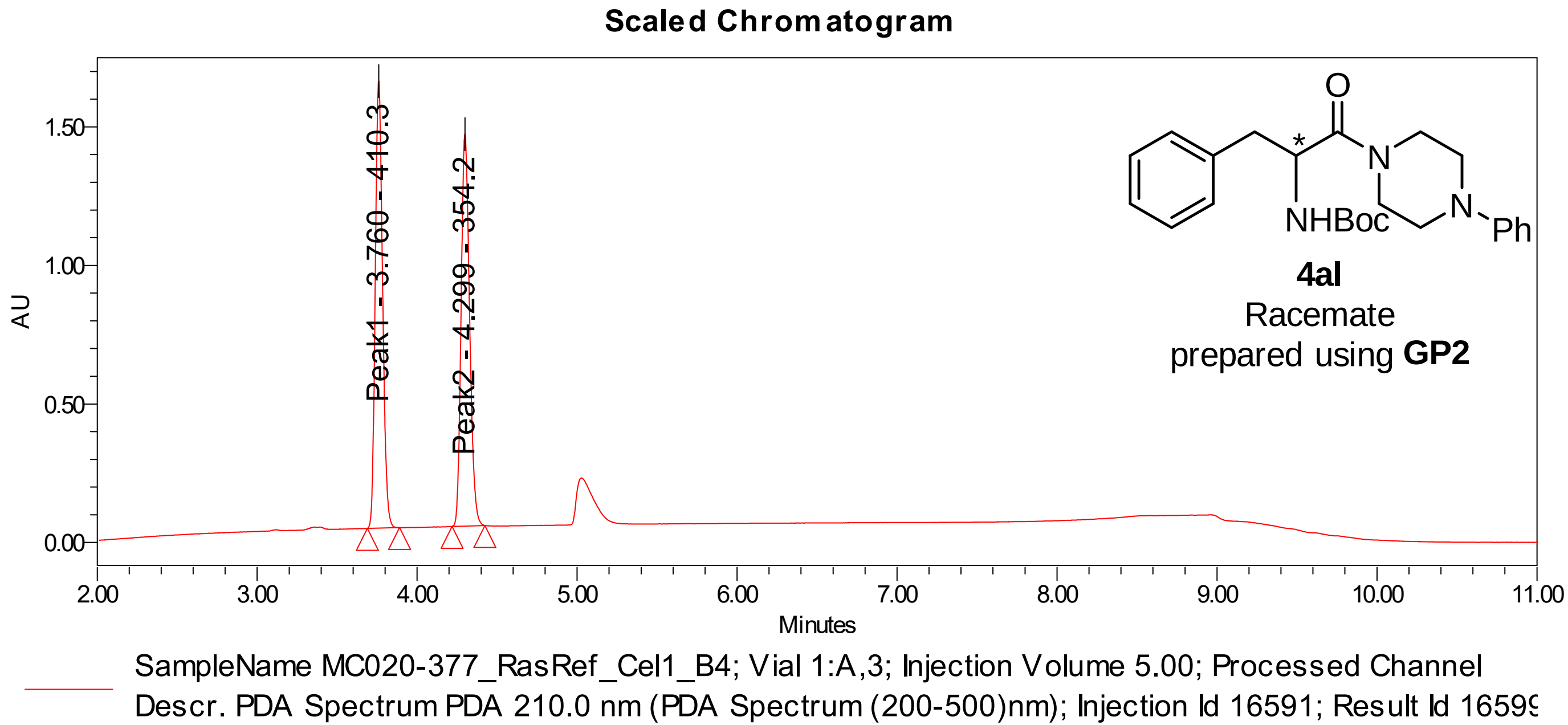

Peak Results

	SampleName	Peak Name	Retention Time (min)	Height (µV)	Area (µV*sec)	% Area
1	MC020-377_RasRef_Cel1_B4	Peak1	3.760	1613988	5133300	49.63
2	MC020-377_RasRef_Cel1_B4	Peak2	4.299	1414586	5209069	50.37

Sample_info n.2mg/ml ACN:MeOH 1:1

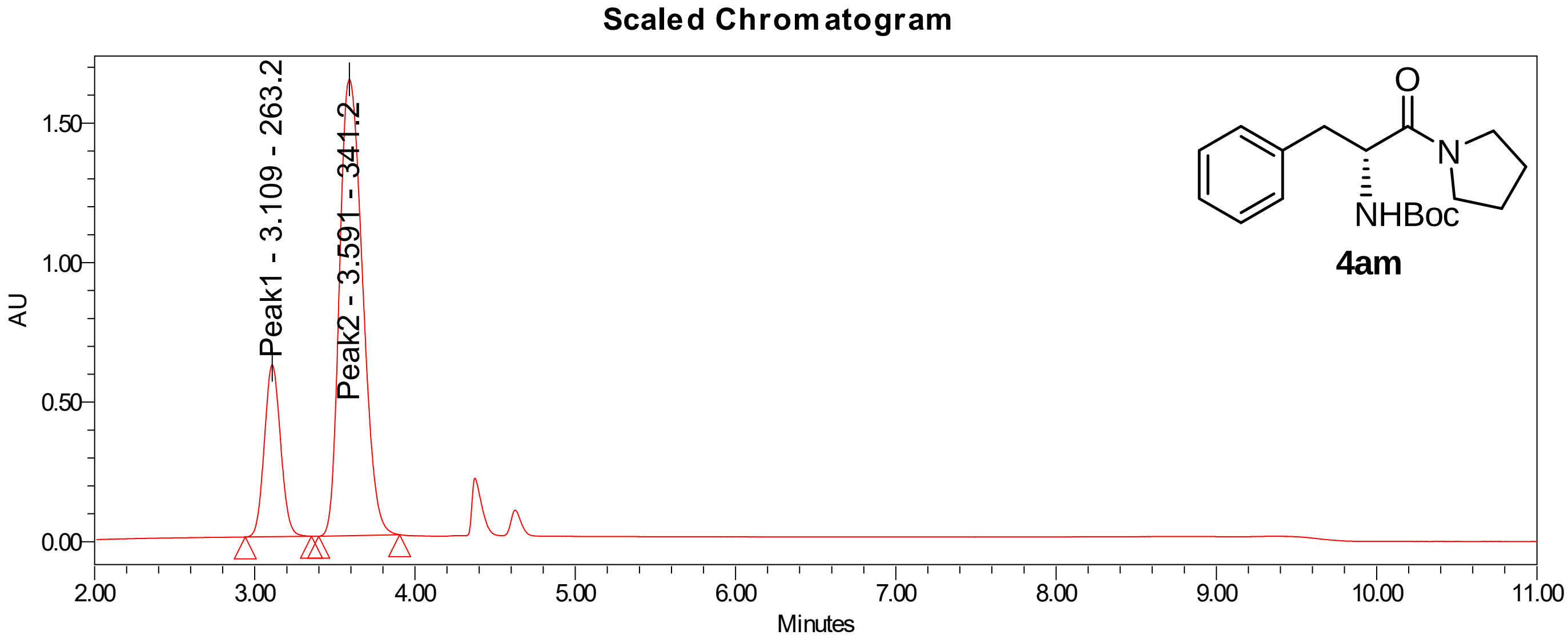

SampleName MC020-375-001_Amyl2_B3; Vial 1:A,2; Injection Volume 5.00; Processed Channel Descr. PDA Spectrum PDA 210.0 nm (PDA Spectrum (200-500)nm); Injection Id 16676; Result Id 16707

Peak Results

	SampleName	Peak Name	Retention Time (min)	Height (μV)	Area (μV*sec)	% Area
1	MC020-375-001_Amyl2_B3	Peak1	3.109	616241	4338013	21.04
2	MC020-375-001_Amyl2_B3	Peak2	3.591	1635275	16277267	78.96

Sample_info n.2mg/ml ACN:MeOH 1:1

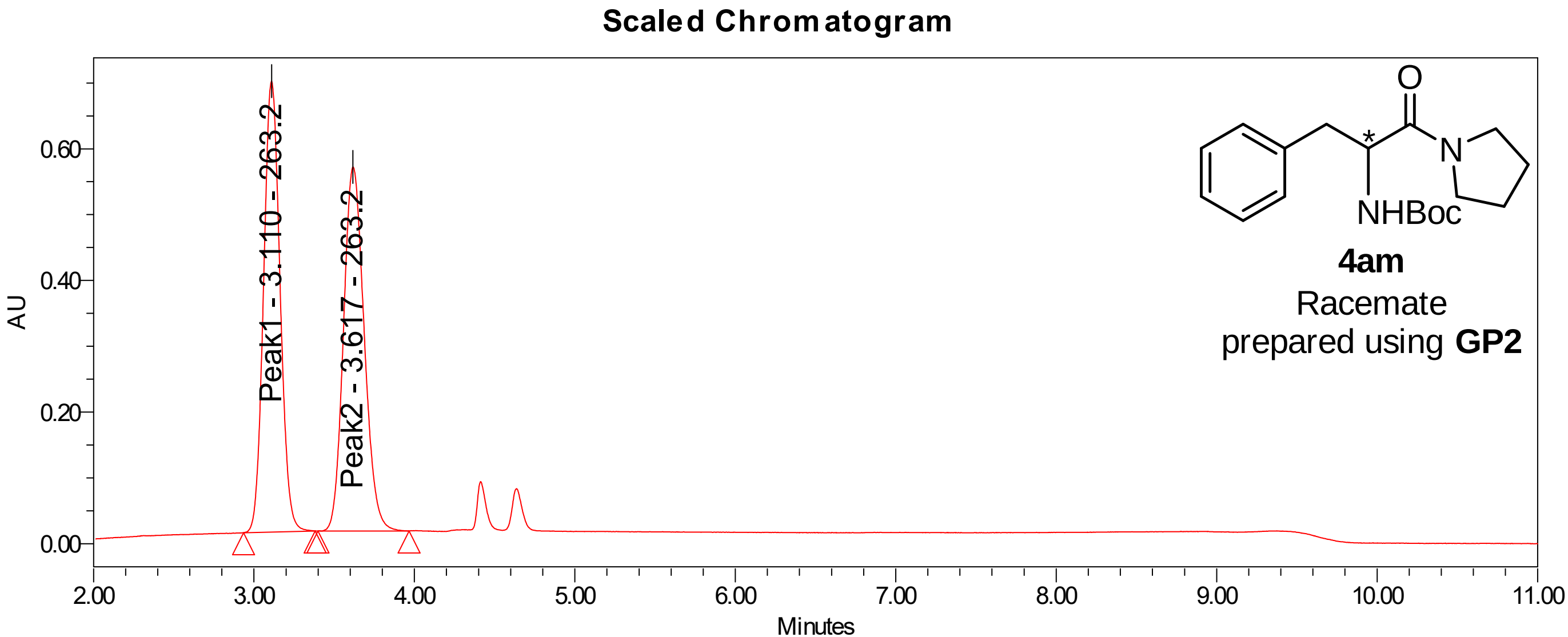

SampleName MC020-371_RasRef_Amyl2_B3; Vial 1:A,3; Injection Volume 5.00; Processed Channel Descr. PDA Spectrum PDA 210.0 nm (PDA Spectrum (200-500)nm); Injection Id 16681; Result Id 16704

Peak Results

	SampleName	Peak Name	Retention Time (min)	Height (µV)	Area (µV*sec)	% Area
1	MC020-371_RasRef_Amyl2_B3	Peak1	3.110	685307	4881216	49.60
2	MC020-371_RasRef_Amyl2_B3	Peak2	3.617	553268	4960196	50.40

Sample_info n.2mg/ml ACN:MeOH 1:1

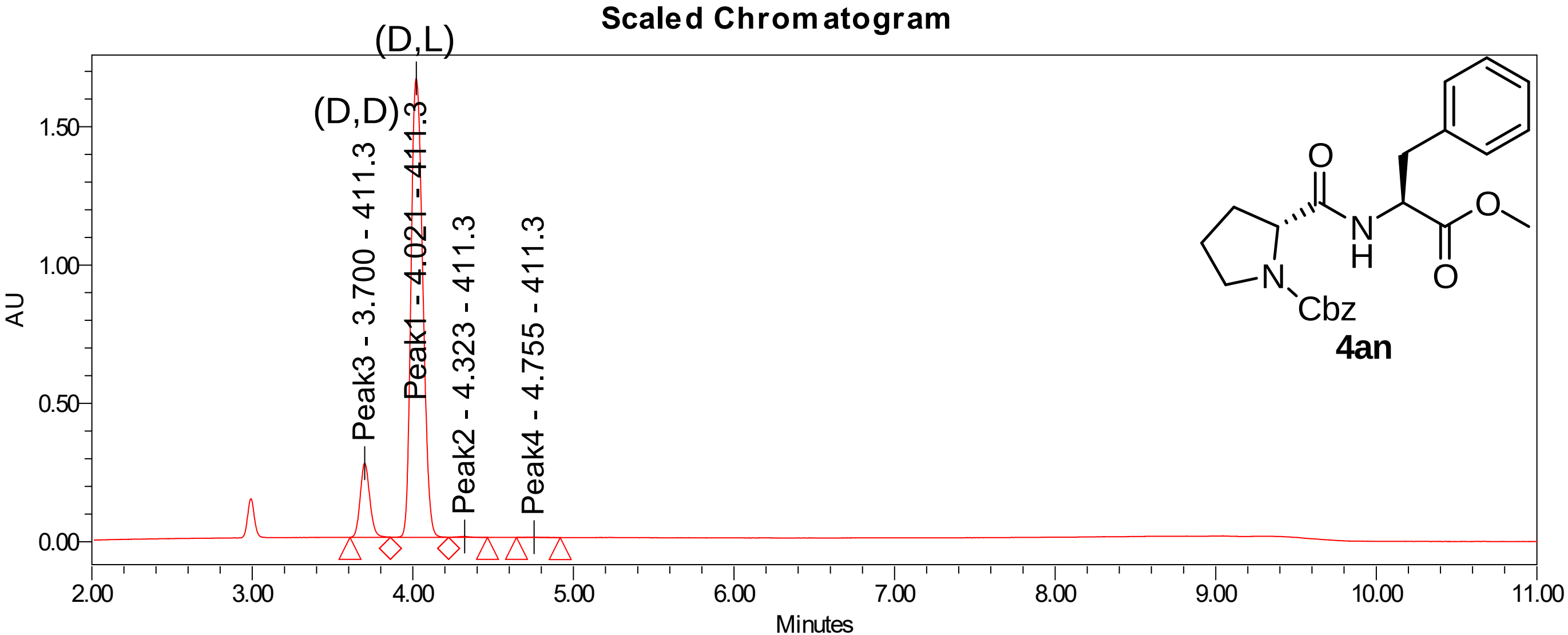

SampleName MC020-384-001_Amy1_B1; Vial 1:A,2; Injection Volume 5.00; Processed Channel Descr. PDA Spectrum PDA 210.0 nm (PDA Spectrum (200-500)nm); Injection Id 17728; Result Id 17753

Peak Results

	SampleName	Peak Name	Retention Time (min)	Height (µV)	Area (µV*sec)	% Area
1	MC020-384-001_Amy1_B1	Peak3	3.700	268320	1115693	11.66
2	MC020-384-001_Amy1_B1	Peak1	4.021	1659209	8419756	88.03
3	MC020-384-001_Amy1_B1	Peak2	4.323	3561	21436	0.22
4	MC020-384-001_Amy1_B1	Peak4	4.755	1217	8160	0.09

Sample_info n.2mg/ml ACN:MeOH 1:1

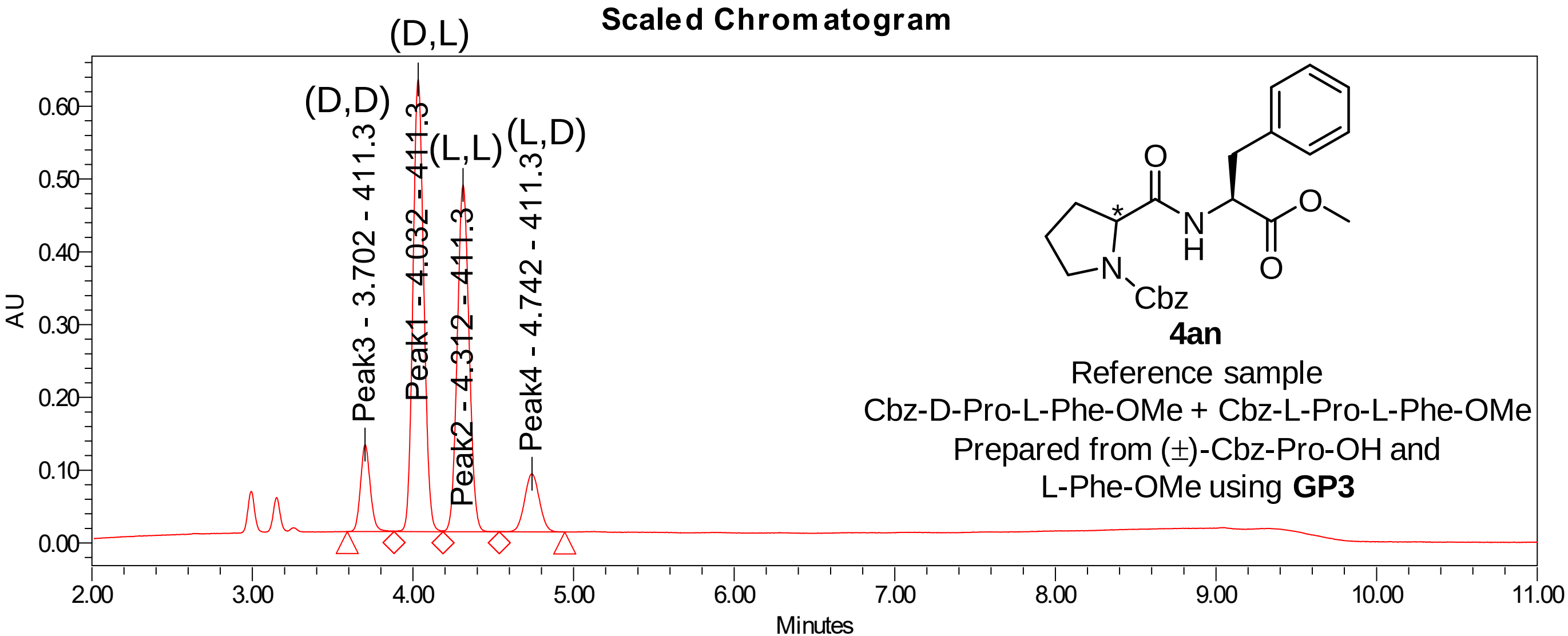

SampleName MC020-394_RasRef_Amy1_B1; Vial 1:A,3; Injection Volume 5.00; Processed Channel Descr. PDA Spectrum PDA 210.0 nm (PDA Spectrum (200-500)nm); Injection Id 17733; Result Id 1775(

Peak Results

	SampleName	Peak Name	Retention Time (min)	Height (µV)	Area (µV*sec)	% Area
1	MC020-394_RasRef_Amy1_B1	Peak3	3.702	119494	503975	7.94
2	MC020-394_RasRef_Amy1_B1	Peak1	4.032	621334	2899254	45.67
3	MC020-394_RasRef_Amy1_B1	Peak2	4.312	476406	2433538	38.34
4	MC020-394_RasRef_Amy1_B1	Peak4	4.742	79744	511262	8.05

Sample_info n.2mg/ml ACN:MeOH 1:1

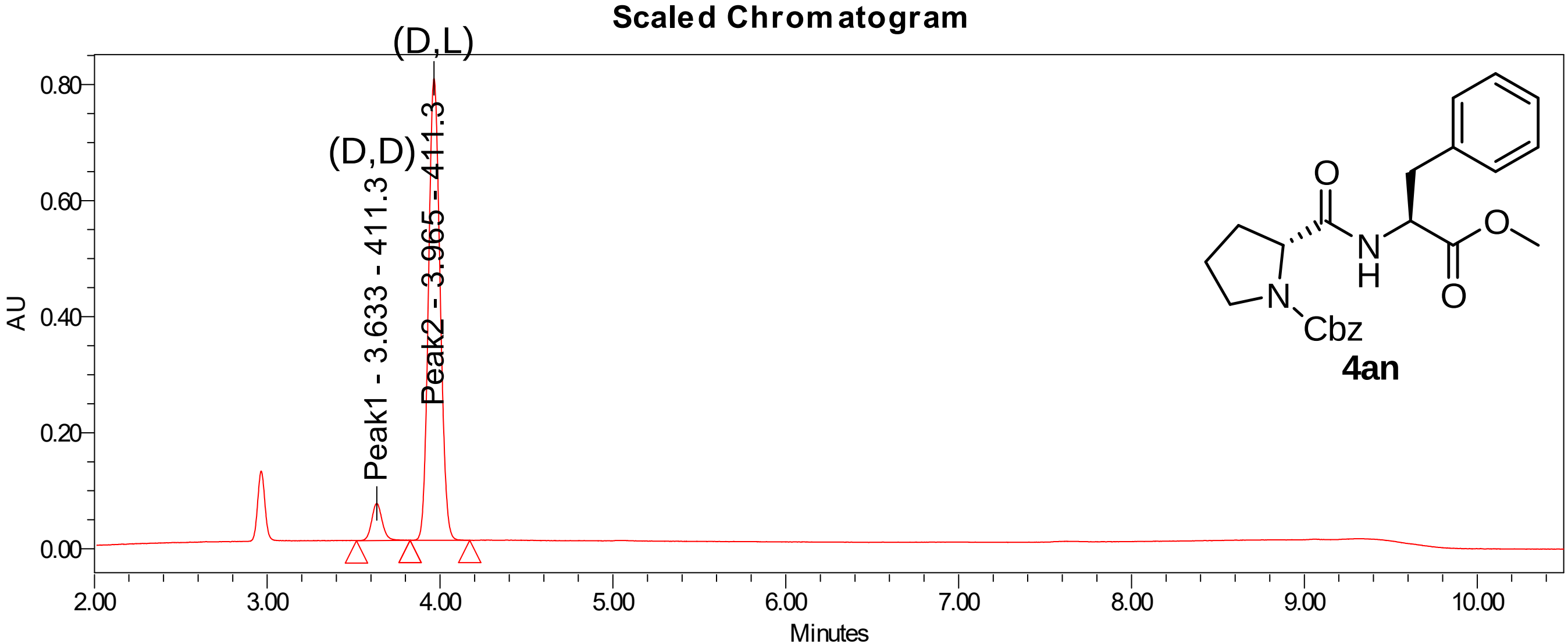

SampleName MC020-405-001_Amy1_B1; Vial 1:A,2; Injection Volume 5.00; Processed Channel Descr. PDA Spectrum PDA 210.0 nm (PDA Spectrum (200-500)nm); Injection Id 19116; Result Id 19159

Peak Results

	SampleName	Peak Name	Retention Time (min)	Height (µV)	Area (µV*sec)	% Area
1	MC020-405-001_Amy1_B1	Peak1	3.633	64006	273329	6.86
2	MC020-405-001_Amy1_B1	Peak2	3.965	796228	3712912	93.14
3	MC020-405-001_Amy1_B1	Peak3	4.366			
4	MC020-405-001_Amy1_B1	Peak4	4.832			

Sample_info n.2mg/ml ACN:MeOH 1:1

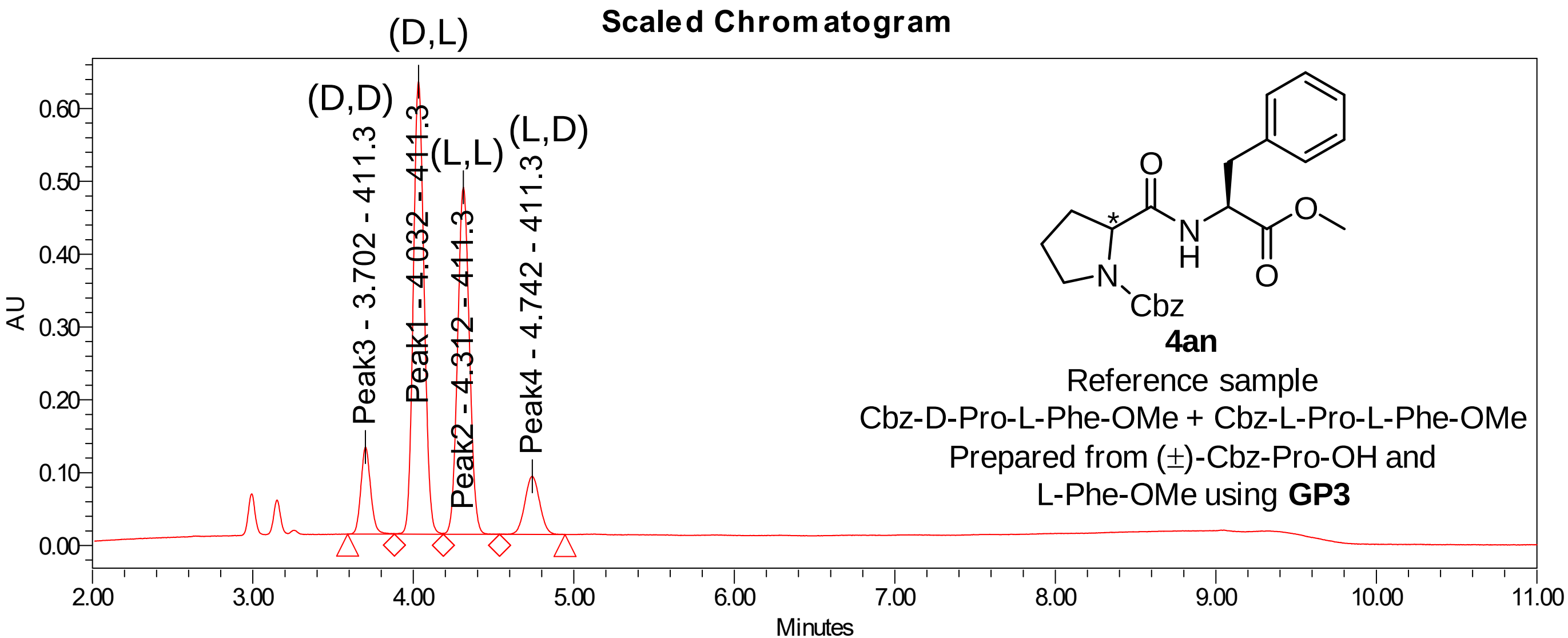

SampleName MC020-394_RasRef_Amy1_B1; Vial 1:A,3; Injection Volume 5.00; Processed Channel Descr. PDA Spectrum PDA 210.0 nm (PDA Spectrum (200-500)nm); Injection Id 17733; Result Id 1775(

Peak Results

	SampleName	Peak Name	Retention Time (min)	Height (µV)	Area (µV*sec)	% Area
1	MC020-394_RasRef_Amy1_B1	Peak3	3.702	119494	503975	7.94
2	MC020-394_RasRef_Amy1_B1	Peak1	4.032	621334	2899254	45.67
3	MC020-394_RasRef_Amy1_B1	Peak2	4.312	476406	2433538	38.34
4	MC020-394_RasRef_Amy1_B1	Peak4	4.742	79744	511262	8.05

Sample_info n.2mg/ml ACN:MeOH 1:1

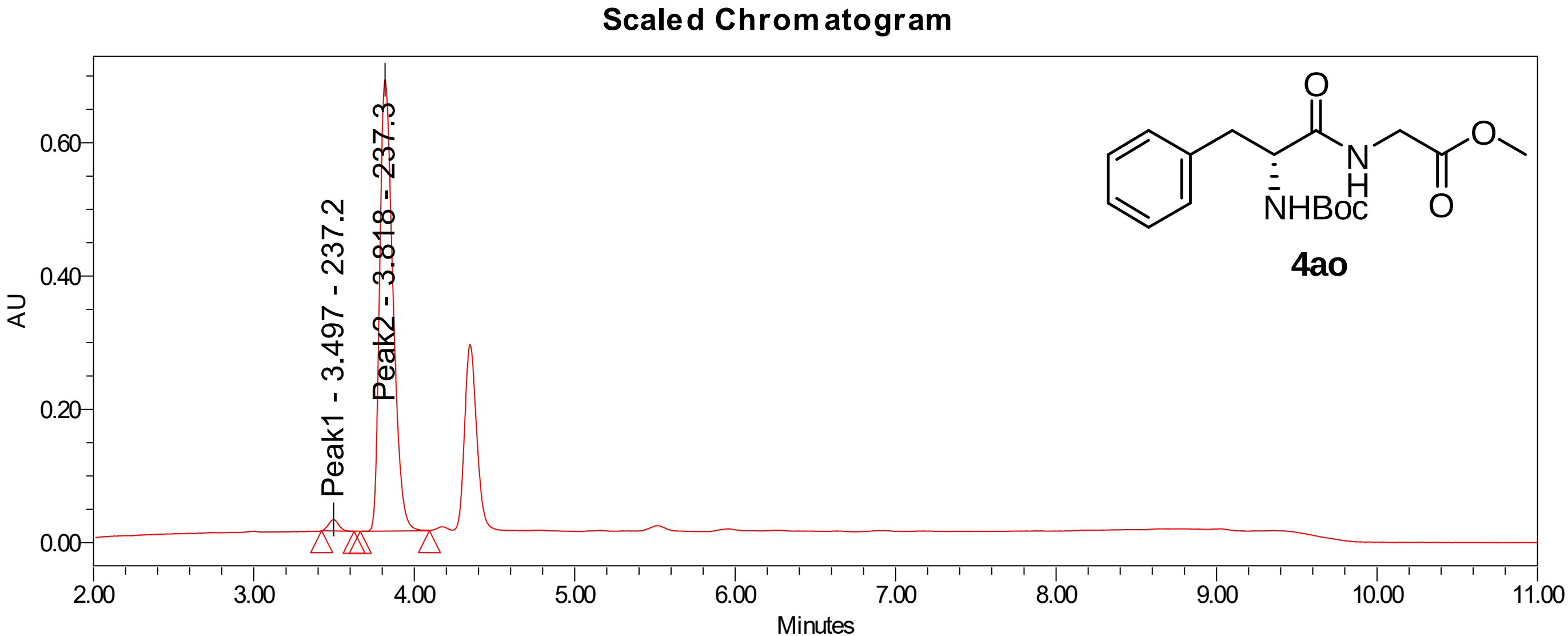

SampleName MC020-383-001_Amy1_B3; Vial 1:A,2; Injection Volume 5.00; Processed Channel Descr. PDA Spectrum PDA 210.0 nm (PDA Spectrum (200-500)nm); Injection Id 18216; Result Id 18259

Peak Results

	SampleName	Peak Name	Retention Time (min)	Height (µV)	Area (µV*sec)	% Area
1	MC020-383-001_Amy1_B3	Peak1	3.497	17127	76189	1.92
2	MC020-383-001_Amy1_B3	Peak2	3.818	677127	3900678	98.08

Sample_info n.2mg/ml ACN:MeOH 1:1

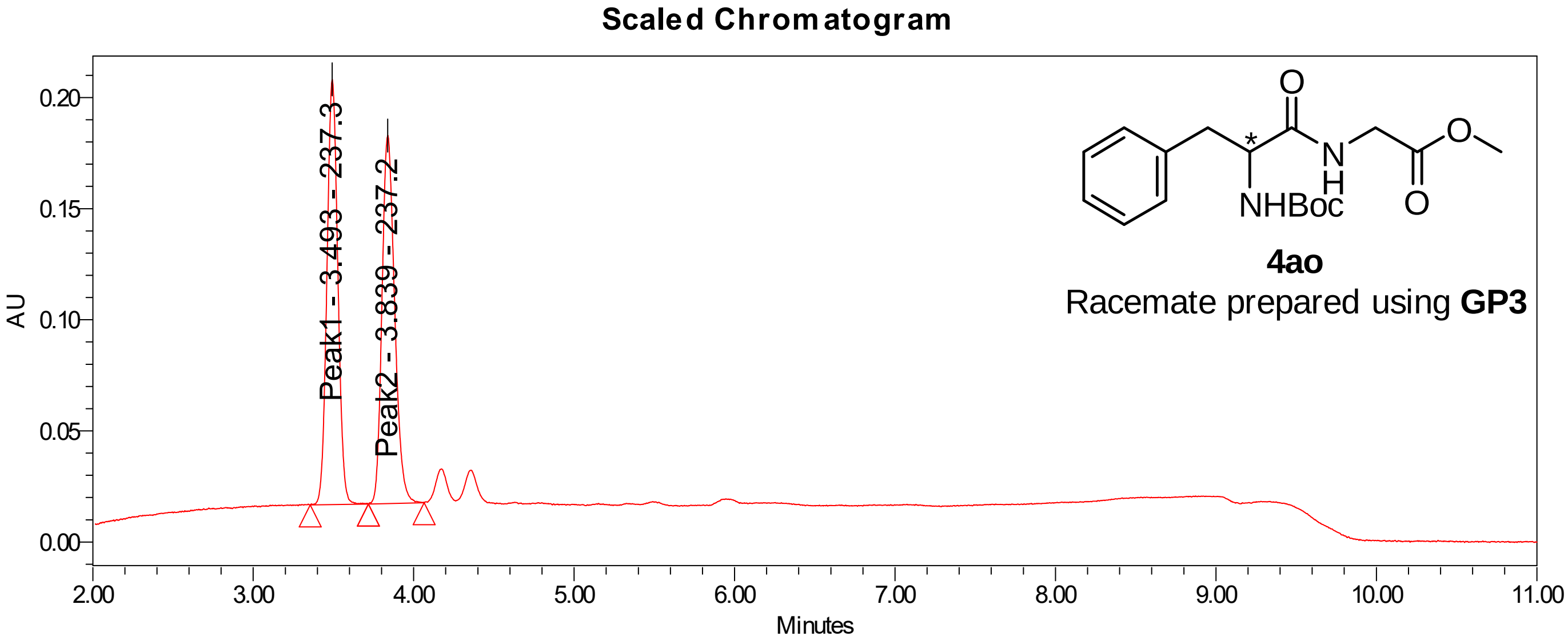

SampleName MC020-390_RasRef_Amy1_B3; Vial 1:A,3; Injection Volume 5.00; Processed Channel Descr. PDA Spectrum PDA 210.0 nm (PDA Spectrum (200-500)nm); Injection Id 18221; Result Id 1825

Peak Results

	SampleName	Peak Name	Retention Time (min)	Height (µV)	Area (µV*sec)	% Area
1	MC020-390_RasRef_Amy1_B3	Peak1	3.493	191365	901812	49.85
2	MC020-390_RasRef_Amy1_B3	Peak2	3.839	165718	907395	50.15

Sample_info n.2mg/ml ACN:MeOH 1:1

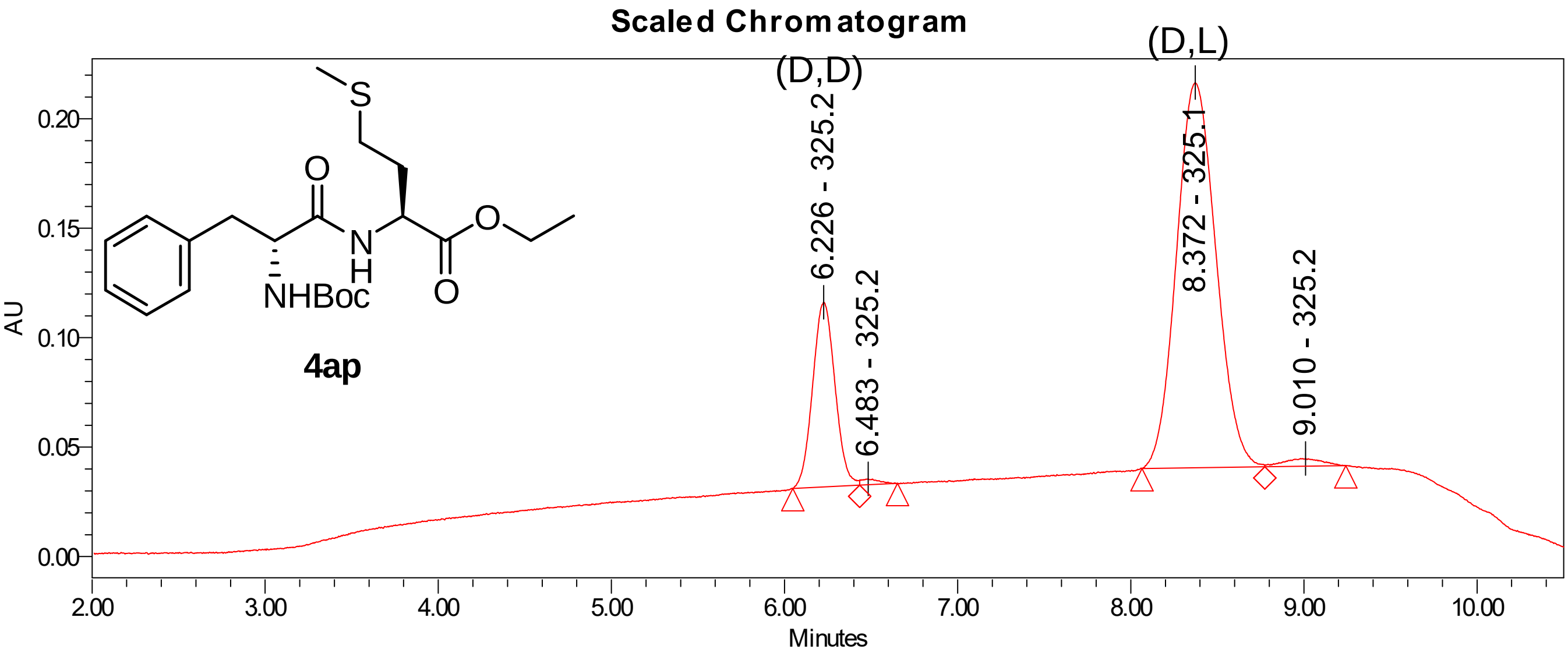

SampleName MC020-389-001_Op_Amyl1; Vial 1:A,4; Injection Volume 5.00; Processed Channel Descr. PDA Spectrum PDA 210.0 nm (PDA Spectrum (200-500)nm); Injection Id 19073; Result Id 19082

Peak Results

	SampleName	Peak Name	Retention Time (min)	Height (µV)	Area (µV*sec)	% Area
1	MC020-389-001_Op_Amyl1		6.226	84393	756189	20.91
2	MC020-389-001_Op_Amyl1		6.483	2764	18886	0.52
3	MC020-389-001_Op_Amyl1		8.372	176029	2786957	77.06
4	MC020-389-001_Op_Amyl1		9.010	3570	54736	1.51

Sample_info n.2mg/ml ACN:MeOH 1:1

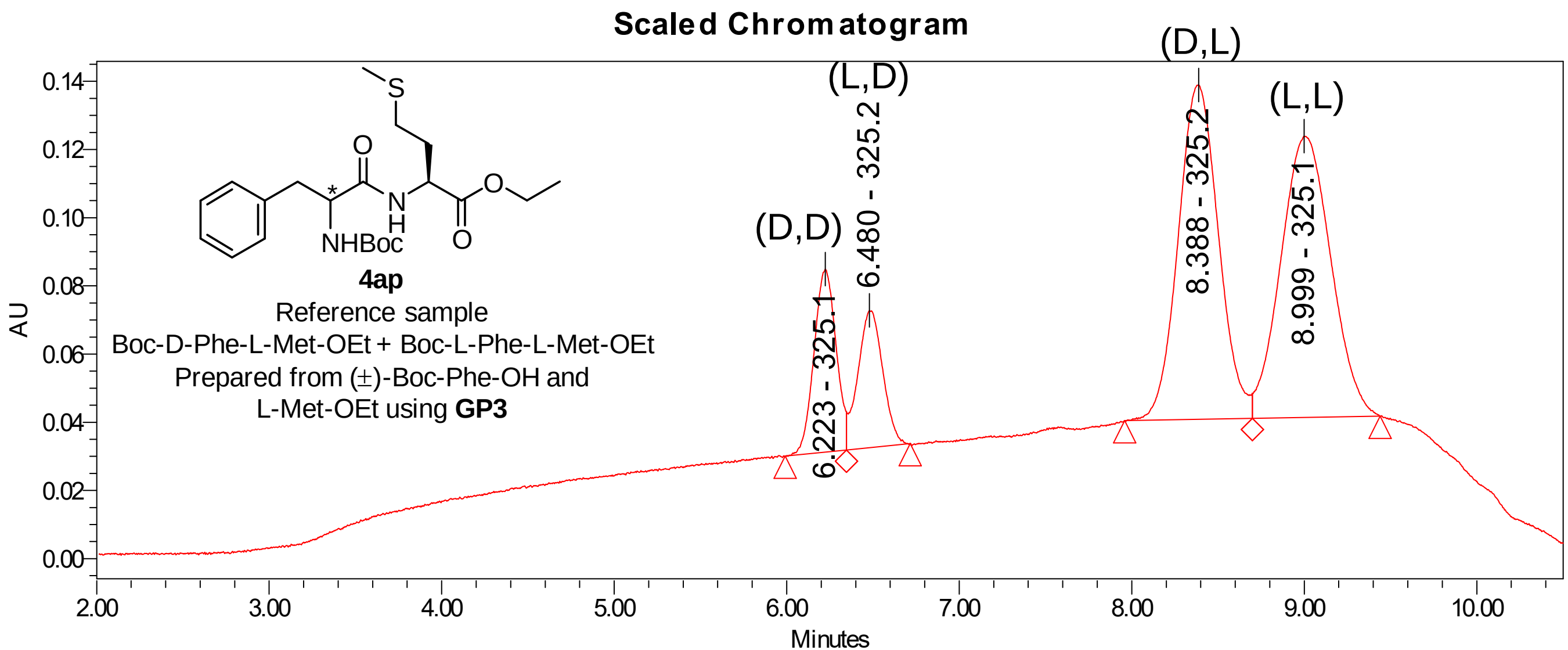

SampleName MC020-391-001_RasRef_Amyl1; Vial 1:A,3; Injection Volume 5.00; Processed Channel Descr. PDA Spectrum PDA 210.0 nm (PDA Spectrum (200-500)nm); Injection Id 19078; Result Id 1909(

Peak Results

	SampleName	Peak Name	Retention Time (min)	Height (µV)	Area (µV*sec)	% Area
1	MC020-391-001_RasRef_Amyl1		6.223	53677	476588	11.52
2	MC020-391-001_RasRef_Amyl1		6.480	40246	403901	9.76
3	MC020-391-001_RasRef_Amyl1		8.388	98033	1592742	38.50
4	MC020-391-001_RasRef_Amyl1		8.999	82508	1664015	40.22

Sample_info n.1mg/ml ACN:MeOH 1:1

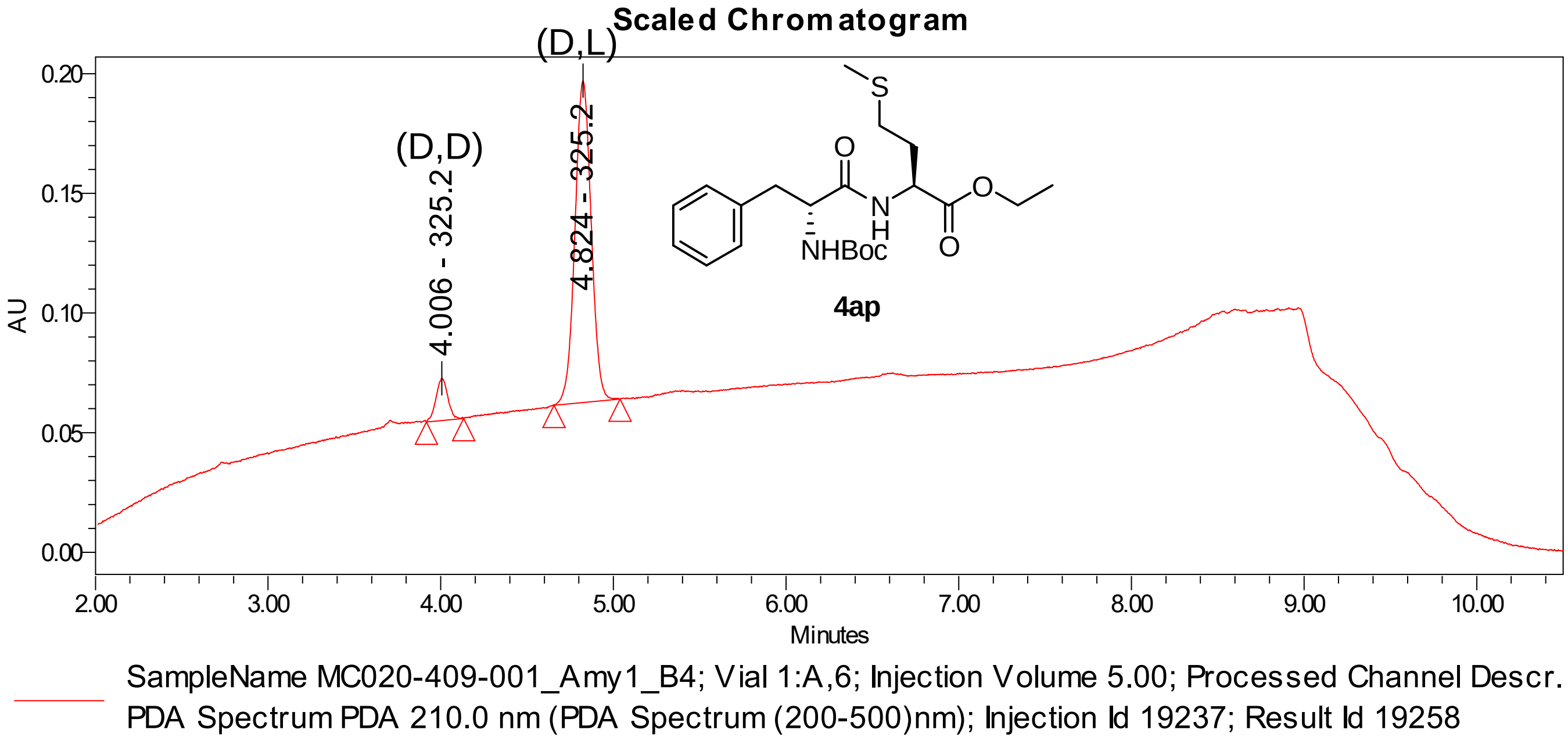

Peak Results

	SampleName	Peak Name	Retention Time (min)	Height (µV)	Area (µV*sec)	% Area
1	MC020-409-001_Amy1_B4		4.006	17691	83924	8.69
2	MC020-409-001_Amy1_B4		4.824	134501	882098	91.31

Sample_info n.1mg/ml ACN:MeOH 1:1

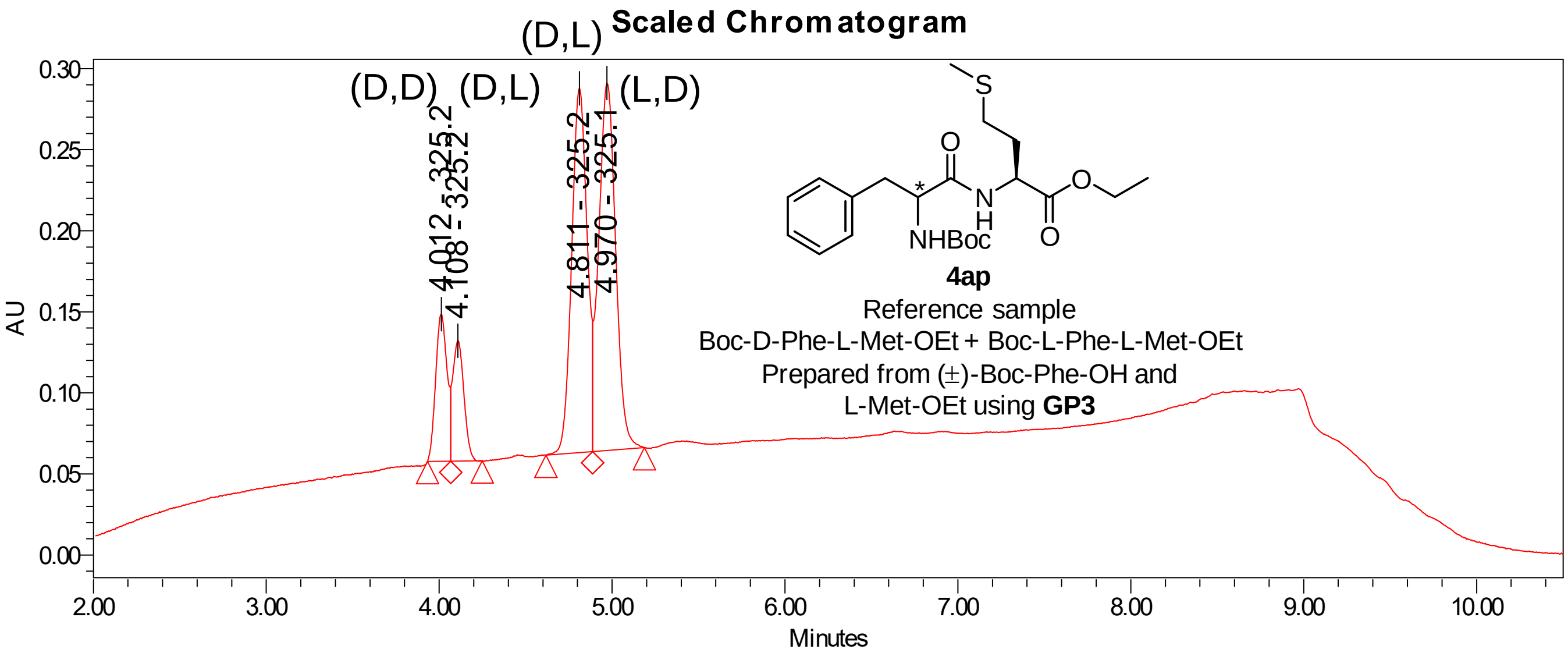

SampleName MC020-391_RasRef_Amy1_B4; Vial 1:A,5; Injection Volume 5.00; Processed Channel Descr. PDA Spectrum PDA 210.0 nm (PDA Spectrum (200-500)nm); Injection Id 19242; Result Id 1925

Peak Results

	SampleName	Peak Name	Retention Time (min)	Height (μV)	Area (μV*sec)	% Area
1	MC020-391_RasRef_Amy1_B4		4.012	90749	417215	11.30
2	MC020-391_RasRef_Amy1_B4		4.108	74248	334620	9.06
3	MC020-391_RasRef_Amy1_B4		4.811	224639	1389718	37.64
4	MC020-391_RasRef_Amy1_B4		4.970	226662	1550955	42.00
