# Supplementary material for: Catalytic Amidation of Carboxylic Acids Using ortho-Aminophenylboronic Acids
Source: ACS Org Inorg Au. 2026 May 12;6(4):422–9. doi: 10.1021/acsorginorgau.6c00021 (PMC13449423; doi:10.1021/acsorginorgau.6c00021)
Supplement: Supplementary file 2 [file gg6c00021_si_002.zip › Metadata for NMR.docx]

**Name of the manufacturer of the spectrometer used to collect the data:**

The ^1^H NMR and ^13^C{^1^H} NMR spectra were recorded in CDCl_3_ or (CD_3_)_2_SO on Bruker Avance 400 or 600 spectrometer. The chemical shifts are reported in ppm relative to CHCl_3_ (δ 7.26) or DMSO-d_6_ (δ 2.50) for ^1^H NMR. For the ^13^C NMR spectra, CDCl_3_ (δ 77.16) or (CD_3_)_2_SO (39.52) were used as the internal standards.

**Acquisition software:** Bruker TopSpin 3.6.4 on 400 MHz or 600 MHz

**Processing programs used to analyse the data:** MestReNova 14.3.0

**The field strength used to measure each nucleus:**

**4a**: **^1^H NMR** 400 MHz, **^13^C NMR** 101 MHz

**4b**: **^1^H NMR** 400 MHz, **^13^C NMR** 101 MHz

**4c**: **^1^H NMR** 400 MHz, **^13^C NMR** 101 MHz

**4d**: **^1^H NMR** 400 MHz, **^13^C NMR** 101 MHz

**4e**: **^1^H NMR** 400 MHz, **^13^C NMR** 101 MHz

**4f**: **^1^H NMR** 400 MHz, **^13^C NMR** 101 MHz

**5f**: **^1^H NMR** 400 MHz, **^13^C NMR** 101 MHz

**4g**: **^1^H NMR** 400 MHz, **^13^C NMR** 101 MHz

**4h**: **^1^H NMR** 400 MHz, **^13^C NMR** 101 MHz

**4i**: **^1^H NMR** 400 MHz, **^13^C NMR** 101 MHz

**4j**: **^1^H NMR** 600 MHz, **^13^C NMR** 151 MHz

**4k**: **^1^H NMR** 400 MHz, **^13^C NMR** 101 MHz

**4l**: **^1^H NMR** 400 MHz, **^13^C NMR** 101 MHz

**4m**: **^1^H NMR** 400 MHz, **^13^C NMR** 101 MHz

**4n:** **^1^H NMR** 500 MHz, **^13^C NMR** 126 MHz

**4o:** **^1^H NMR** 400 MHz, **^13^C NMR** 101 MHz

**4p**: **^1^H NMR** 400 MHz, **^13^C NMR** 101 MHz

**4q**: **^1^H NMR** 400 MHz, **^13^C NMR** 101 MHz

**4r**: **^1^H NMR** 400 MHz, **^13^C NMR** 101 MHz

**4s**: **^1^H NMR** 400 MHz, **^13^C NMR** 101 MHz

**4t**: **^1^H NMR** 400 MHz, **^13^C NMR** 101 MHz

**4u**: **^1^H NMR** 400 MHz, **^13^C NMR** 101 MHz

**4v**: **^1^H NMR** 400 MHz, **^13^C NMR** 101 MHz

**4w**: **^1^H NMR** 400 MHz, **^13^C NMR** 101 MHz

**4x**: **^1^H NMR** 400 MHz, **^13^C NMR** 101 MHz

**4y**: **^1^H NMR** 400 MHz, **^13^C NMR** 101 MHz

**4z**: **^1^H NMR** 400 MHz, **^13^C NMR** 101 MHz

**4aa**: **^1^H NMR** 400 MHz, **^13^C NMR** 101 MHz

**4ab**: **^1^H NMR** 400 MHz, **^13^C NMR** 101 MHz

**4ac**: **^1^H NMR** 400 MHz, **^13^C NMR** 101 MHz

**4ad**: **^1^H NMR** 400 MHz, **^13^C NMR** 101 MHz

**4ae**: **^1^H NMR** 500 MHz, **^13^C NMR** 126 MHz

**4af**: **^1^H NMR** 400 MHz, **^13^C NMR** 101 MHz

**4ag**: **^1^H NMR** 400 MHz, **^13^C NMR** 101 MHz

**4ah**: **^1^H NMR** 600 MHz, **^13^C NMR** 151 MHz

**4ai**: **^1^H NMR** 400 MHz, **^13^C NMR** 101 MHz

**4aj**: **^1^H NMR** 400 MHz, **^13^C NMR** 101 MHz

**4ak**: **^1^H NMR** 400 MHz, **^13^C NMR** 101 MHz

**4al**: **^1^H NMR** 400 MHz, **^13^C NMR** 101 MHz

**4am**: **^1^H NMR** 400 MHz, **^13^C NMR** 101 MHz

**4an**: **^1^H NMR** 400 MHz, **^13^C NMR** 101 MHz

**4ao**: **^1^H NMR** 400 MHz, **^13^C NMR** 101 MHz

**4ap**: **^1^H NMR** 600 MHz, **^13^C NMR** 151 MHz

**S1a**: **^1^H NMR** 400 MHz, **^13^C NMR** 101 MHz

**S1b**: **^1^H NMR** 600 MHz, **^13^C NMR** 151 MHz

**3s**: **^1^H NMR** 400 MHz, **^13^C NMR** 101 MHz

**3t**: **^1^H NMR** 400 MHz, **^13^C NMR** 101 MHz
